# A transdisciplinary framework for chronic illness and chronic pain: a sequential qualitative meta-synthesis

**DOI:** 10.3389/fpubh.2026.1914023

**Published:** 2026-08-12

**Authors:** Sam Safavi-Abbasi, Rory Tasker, Phillip M. Reyes, Brenden Doyle, Payam Dibaj, Elisa A. Venezia

**Affiliations:** 1 Department of Neurological Surgery & Neurosciences, Creighton University, Phoenix Campus, Yavapai Regional Medical Group, Yavapai Regional Medical Center, Prescott, AZ, United States; 2 Department of Curriculum, Teaching and Learning (CTL) Ontario Institute for Studies in Education (OISE), University of Toronto, Toronto, ON, Canada; 3 Barrow Neurological Institute, St. Joseph’s Hospital and Medical Center, Phoenix, AZ, United States; 4 Department of Pediatrics, Center for Rare Diseases Göttingen (ZSEG), University Medical Center Göttingen, Göttingen, Germany

**Keywords:** affordance theory, chronic pain, hermeneutics, heuristics, mindfulness, phenomenology, process ontology, transdisciplinary science

## Abstract

**Introduction:**

Chronic pain is a common, disabling feature of many long-term conditions, yet its clinical management remains fragmented across disciplines, diagnoses, and organizations. Although a substantial qualitative literature describes patients’ lived experience of chronic illness and chronic pain, these findings have rarely been synthesized into a transdisciplinary framework explicitly oriented toward coordinated clinical care. We therefore aimed to develop such a framework grounded in lived experience and relevant to clinical application.

**Methods:**

We conducted a sequential qualitative meta-synthesis in two phases. Phase 1 comprised a systematic review of 104 primary qualitative studies and qualitative evidence syntheses published between 2009 and 2025 examining lived experiences of chronic illness and chronic pain. Searches across eight databases were screened using explicit eligibility criteria. Study quality was appraised using the Critical Appraisal Skills Programme and the Mixed Methods Appraisal Tool, and confidence in synthesized findings was assessed using Grading of Recommendations Assessment, Development and Evaluation–Confidence in the Evidence from Reviews of Qualitative Research. Through iterative hermeneutic reading, thematic coding, and meta-ethnographic translation, we identified recurrent experiential themes, grouped them into higher-order domains, and visualized their relationships using node graphs. Phase 2 reviewed 52 additional studies on transdisciplinary principles, collaboration, implementation, and systems practice. These principles were mapped onto the experiential domains and integrated into a clinically oriented transdisciplinary framework. In total, 156 studies were included across both phases.

**Results:**

Phase 1 identified recurrent and interconnected experiential domains characterizing chronic conditions as lived processes, including social isolation, identity disruption, physical debilitation, emotional distress, cognitive burden, uncertainty, and struggles for legitimacy within healthcare and social systems. These domains were not discrete but dynamically interrelated. Phase 2 identified core transdisciplinary principles, particularly communication, collaboration, participation, reflexivity, and systems thinking, that aligned closely with these experiential domains. Their integration produced a novel transdisciplinary framework that conceptualizes chronic illness and chronic pain as dynamic, relational, and temporally unfolding processes and links lived-experience domains to clinically relevant collaborative pathways.

**Conclusion:**

This study advances a transdisciplinary framework for chronic illness and chronic pain that is grounded in qualitative evidence and oriented toward clinical application. The framework offers a structured basis for person-centered assessment, interdisciplinary communication, longitudinal care planning, and coordinated management of complex chronic conditions.

## 1 Introduction

Chronic illnesses are increasingly widespread and are major contributors to global mortality, disability, morbidity, and health disparities (1–8). Chronic pain is a common and disabling feature of many long-term conditions and is closely linked to multimorbidity, cognitive decline, sleep disturbance, depression, and anxiety (1, 3–5, 9). Although chronic illness and chronic pain are often managed as separate clinical problems, patients frequently experience them as overlapping and interdependent processes that unfold across time and social context (1, 3–5, 8, 9).

Current approaches to chronic illness and chronic pain remain fragmented across specialties, diagnoses, and healthcare systems (10–13). Increasing specialization within medical and psychological subspecialties, together with the organization of healthcare delivery into discrete operational units, has reduced interdisciplinary communication and collaboration (10–13). These structural limitations are especially problematic in conditions characterized by etiological heterogeneity, fluctuating symptom burden, and weak correspondence between biomarkers and subjective reports of suffering (9, 11, 12, 14, 15). Recognition of medically unexplained syndromes is also increasing, as more individuals present with conditions that elude diagnosis through conventional clinical and technological methods (14, 15). As a result, patients commonly encounter delays in diagnosis, inefficient care pathways, and treatments that do not adequately reflect the complexity of lived illness (10–15).

Pain is one of the most burdensome manifestations of chronic disease (1, 3–5, 9). Many chronic conditions, including arthritis, rheumatoid arthritis (RA), fibromyalgia, and gastrointestinal disorders, involve persistent or recurrent somatic pain (1, 3). These bodily symptoms reflect complex interactions among physiological, psychological, somatic, and neurological processes (14, 15). Although some pain-related conditions have identifiable biomarkers, these do not consistently correspond to the severity, distribution, or impact of pain as experienced by patients (9). Psychosocial and behavioral factors, along with broader determinants such as socioeconomic deprivation, cultural influences, and social norms, also shape the development, progression, and experience of chronic illness and pain (6–10). Chronic pain and depression frequently co-occur, further complicating efforts to understand disease trajectories and treatment needs (4, 9).

A substantial body of qualitative research has examined how people live with chronic illness and chronic pain, including experiences of suffering, coping, uncertainty, identity disruption, stigma, and relationships with caregivers and clinicians (8, 16–28). Qualitative evidence syntheses (QES), including thematic syntheses and meta-ethnographies, have made important contributions by integrating findings across studies to deepen understanding of patient and caregiver perspectives (8, 18, 19, 21, 22, 24–28). Thematic synthesis provides a structured approach for combining findings from diverse qualitative studies through line-by-line coding, development of descriptive themes, and generation of analytical themes (29). Interpretive syntheses, including meta-ethnography and grounded theory approaches, further allow concepts and explanatory frameworks to emerge inductively through cross-study analysis (16, 29, 30). Meta-ethnography has been particularly influential in synthesizing lived-experience research in healthcare, both in accordance with Noblit and Hare’s original framework and as part of broader meta-study approaches (16, 30).

However, an important gap remains. Although existing qualitative studies and syntheses have richly described lived experience, they have rarely been integrated into a transdisciplinary framework that is explicitly oriented toward clinical implementation. In other words, while prior work has helped clarify what chronic illness and chronic pain feel like and how they affect everyday life, it has less often shown how these experiential findings can be systematically translated into coordinated, person-centered, and clinically meaningful care pathways. This gap is especially important in chronic conditions in which biological, psychological, social, cultural, and systems-level factors interact in nonlinear and evolving ways (11, 12, 31–33).

Transdisciplinary methodologies offer one way to address this complexity by integrating disciplinary expertise, stakeholder perspectives, and multiple forms of knowledge production (10–12, 31–33). Such approaches have advanced understanding of disease mechanisms, causation, and intervention development by supporting collaboration across disciplinary and institutional boundaries (10–12). In the context of chronic illness and chronic pain, transdisciplinarity provides a way to connect clinical knowledge with patient experience rather than treating them as separate domains. Yet, for this approach to inform practice, structured pathways are needed to translate lived-experience evidence into clinically relevant conceptual and organizational models.

Accordingly, this study aimed to develop a transdisciplinary framework for chronic illness and chronic pain that is grounded in lived experience and oriented toward clinical application. Specifically, we sought to: (1) synthesize qualitative evidence on the lived experience of chronic illness and chronic pain; (2) identify higher-order experiential domains and organize them into an integrative conceptual structure; and (3) align these domains with transdisciplinary principles relevant to coordinated clinical care. To do so, we used a sequential qualitative meta-synthesis that combined hermeneutic phenomenology, thematic synthesis, meta-ethnographic translation, and heuristic organization of findings to support clinically actionable understanding.

To guide formulation of the review question and scope of the synthesis, we used the PICo framework, comprising Population, Phenomena of Interest, and Context (34). In qualitative evidence synthesis, PICo helps define the focus of inquiry by specifying whose experiences are being examined, what phenomena are of interest, and in what context those experiences are situated.

- P (Population): adult patients >18 years old with chronic pain and chronic illness. A comprehensive analysis of qualitative research was conducted to identify key factors associated with chronic pain and chronic illness.
- I (Phenomena of Interest): patients’ lived experiences, caregivers’ and clinicians’ perspectives, contributors to illness chronicity. Major themes concerning the impact of chronic pain and illness, including physical, psychological, social, and economic burdens experienced by individuals and their families, were examined.
- Co (Context): challenges associated with comprehending, diagnosing, and managing chronic pain and chronic illness processes. This context informs healthcare policy and practice by supporting transdisciplinary approaches to improve the effectiveness of treatment and diagnostic pathways for chronic pain and chronic illness.

## 2 Methods

### 2.1 Study design

This study used a sequential transdisciplinary qualitative meta-synthesis to investigate the phenomenology of chronic illness and chronic pain in clinical medicine. The design integrated hermeneutic phenomenology, thematic synthesis, meta-ethnographic translation, and heuristic organization of findings to support clinically actionable interpretation (20, 29, 30, 35–41). Qualitative methodology was selected because it is well suited to examining lived experience, human interaction, interprofessional relationships, and the complex dynamics of chronic illness and chronic pain that are not readily captured by quantitative methods alone.

The analysis was informed by Thomas and Harden’s approach to thematic synthesis, including line-by-line coding, development of descriptive themes, and generation of analytical themes (29). An iterative mixed inductive-deductive strategy supported both descriptive and interpretive synthesis. Included studies were double-coded by an interdisciplinary team, and the evolving codebook was developed from first-order line-by-line codes to higher-order constructs and meta-themes. Judgment-informed weighting was then applied to reflect both thematic frequency and clinical or theoretical centrality. No dedicated qualitative data analysis software was used for coding. Coding and synthesis were conducted manually using structured extraction forms, annotated coding tables, and iterative team-based comparison. d3.js was used only for visualization of thematic relationships. We also report researcher reflexivity (team backgrounds and roles), triangulation of analysts, and procedures for resolving coding disagreements; de-identified extraction data, coding tables, and audit trails are available in the Supplementary materials to support transparency.

For clarity, the methodology was structured at two levels. First, five pre-synthesis steps established the conceptual and procedural foundation of the review: (1) identification of the interdisciplinary team and transdisciplinary orientation; (2) development of the study aim, scope, and required methods and tools; (3) formulation of the review questions and initial coding protocol; (4) systematic search planning, including medical subject headings (MeSH), controlled vocabulary, and screening procedures; and (5) planning for quality appraisal, PRISMA reporting, and the CERQual evidence profile (34, 42, 43). Second, the analytic review and synthesis proceeded through seven synthesis steps derived from meta-ethnographic and qualitative synthesis traditions (20, 29, 30, 36–38, 44). These seven steps described the progression from study identification to translation, interpretive synthesis, and expression of the final conceptual framework. Although presented sequentially, the process was iterative and involved ongoing reflexivity, peer debriefing, and collaborative reinterpretation throughout (35–41). The review was conducted and reported in accordance with PRISMA 2020 guidance (42). Supplementary materials include the full search strings, PRISMA flow diagram, extraction tables, appraisal outputs, codebook, coding records, audit trail, and additional methodological details.

The PICo framework was used to guide the review question and scope (34). The population included adults aged >18 years living with chronic illness and chronic pain. The phenomena of interest included patients’ lived experiences, relevant caregiver and clinician perspectives, and perceived contributors to chronicity. The context concerned challenges related to understanding, diagnosing, and managing chronic illness and chronic pain and their implications for healthcare policy and transdisciplinary practice.

A combined hermeneutic and heuristic design was used to support a refined understanding of chronic illness-related pain (29, 35, 39). Here, heuristics functioned as structured interpretive tools for organizing themes, identifying conceptual relationships, and translating qualitative findings into clinically relevant conceptual models. The Supplementary materials provide an overview of phases 0–3 and the seven-step sequential synthesis protocol developed during the research process.

The research team included members from medicine, nursing, social sciences, and engineering. This transdisciplinary composition strengthened interpretive rigor by incorporating diverse disciplinary perspectives into screening, coding, peer debriefing, thematic comparison, and synthesis development (35–38). SSA and EAV conducted the primary title, abstract, full-text, and line-by-line coding procedures. All team members contributed to codebook development, thematic discussion, interpretive comparison, and final consensus coding (38, 39).

### 2.2 Search strategy and eligibility criteria

A comprehensive literature search was conducted across Medline, SCOPUS, Google Scholar, Cochrane Library, PsycINFO, AMED, ProQuest, and Web of Science. Searches were designed to identify qualitative evidence syntheses and primary qualitative studies examining experiences of chronic illness and/or chronic pain. Search strings combined keywords, MeSH terms, and controlled vocabulary related to chronic illness, chronic pain, phenomenology, qualitative research, meta-ethnography, and lived experience. Searches were limited to January 2009 through December 2025. Full search strings are provided in Supplementary Table S3.

Forward and backward citation tracking was also performed. The initial search yielded 4,512 records. After removal of duplicates and clearly irrelevant titles, 770 records remained. Following manual deduplication, 350 studies were screened by title and abstract by two reviewers (SSA and EAV). Twelve additional studies were excluded, and 338 full-text articles were assessed for eligibility (PRISMA flow diagram, Supplementary Figure S1). Studies were included if they: (1) reported qualitative findings or qualitative evidence synthesis relevant to chronic illness and/or chronic pain; (2) focused on adults aged >18 years; (3) addressed patients’ lived experiences and/or relevant caregiver or clinician perspectives; (4) were published between January 2009 and December 2025; and (5) were available as peer-reviewed full-text publications. Studies were excluded if they: (1) focused exclusively on participants aged <18 years; (2) were dissertations, theses, book chapters, editorials, or other non-peer-reviewed sources; (3) lacked relevant qualitative data or interpretive findings; or (4) did not address chronic illness and/or chronic pain in relation to the review aims. A total of 104 studies met inclusion criteria for phase 1 of the sequential synthesis (for PRISMA flow diagram please see Supplementary Figures S1, S2). These comprised 42 phenomenological studies, 23 interpretive phenomenological analyses, 16 qualitative interview studies, 15 meta-ethnographies, and 8 qualitative evidence reviews (see Supplementary references), representing 162,175 participants across 28 countries (Supplementary Figure S2). In phase 2, 52 additional studies were reviewed to identify foundational transdisciplinary principles corresponding to the domains established in phase 1 and phenomenological hermeneutics. In total, 156 studies were included across both phases of the sequential synthesis.

### 2.3 Quality appraisal

Included studies were appraised using the Mixed Methods Appraisal Tool (43) and the Critical Appraisal Skills Programme checklist, CASP (2020)^1^ (Supplementary Figure S2; Supplementary Table S3). Quality appraisal informed confidence in the evidence but did not replace interpretive assessment of conceptual relevance. Confidence in individual review findings was further assessed using GRADE-CERQual at the finding level, considering methodological limitations, coherence, adequacy, and relevance of contributing data.

### 2.4 Synthesis procedure

The analytic process followed seven synthesis steps informed by meta-ethnography and thematic synthesis (20, 29, 30, 36–38, 44).

#### 2.4.1 Stage 1: getting started

This stage established the rationale, aims, and protocol for the review and initiated the literature search, screening, and quality appraisal processes (20, 30, 36–38, 44).

#### 2.4.2 Stage 2: identification of relevant content

Qualitative evidence syntheses and primary qualitative studies examining experiences of chronic illness and/or chronic pain were identified through systematic screening of titles, abstracts, and full texts by SSA and EAV.

#### 2.4.3 Stage 3: study selection process

Selected studies were read in full to identify key interpretive metaphors, concepts, participant accounts, and contextual details. Demographic and methodological information was systematically extracted (Supplementary Table S2). Screening followed PRISMA guidance (42). Disagreements during title/abstract or full-text screening were first discussed between SSA and EAV. If disagreement remained, the study was reviewed by a third reviewer and, when necessary, discussed with the full team until consensus was reached.

#### 2.4.4 Stage 4: determining relationships among studies

Studies were compared to identify similarities, differences, recurring metaphors, and conceptual relationships. Significant terms, frameworks, metaphors, and experiential patterns were catalogued and examined across studies using reciprocal translation, refutational analysis, and line-of-argument reasoning (20, 29, 30, 36–39).

#### 2.4.5 Stage 5: translating studies into one another

Two reviewers (SSA and EAV) conducted line-by-line coding of included data segments. First-order participant constructs and second-order author interpretations were compared and translated across studies through a collaborative hermeneutic process (20, 30, 36–39). Group discussions were used to refine codes, metaphors, themes, and the evolving codebook. Inter-rater reliability was assessed using raw percent agreement and Cohen’s kappa; across 115 coded themes derived from 104 studies, the two reviewers achieved ≥80% agreement and a Cohen’s kappa of 0.80 (95% CI 0.75–0.85), indicating substantial agreement beyond chance. Details of the coding procedures, code development, discrepancy resolution, and audit trail are provided in Supplementary Tables S2, S4, S8–S15.

#### 2.4.6 Stage 6: synthesizing translations

Translated concepts were integrated into higher-order categories, themes, and meta-themes using constant comparison and hermeneutic interpretation (36–40). Overarching social, cognitive, physical, and psychological domains and their subthemes were synthesized and mapped into a networked interpretive structure.

#### 2.4.7 Stage 7: expressing the synthesis

The final synthesis was organized into a hierarchical interpretive framework suitable for conceptual and clinical application. Heuristic methods supported structured representation of relationships among chronic illness, chronic pain, and social, cultural, somatic, cognitive, and psychological domains (35, 39, 41). This second level of synthesis generated interpretations extending beyond the findings of individual studies (35, 38, 39, 41).

### 2.5 Weighting and sensitivity analysis

Because the evidence base included both small primary qualitative studies and large aggregated evidence reviews, weighting procedures were used to minimize distortion based on participant volume alone. Each included source contributed one set of codes at the study level, irrespective of sample size. Smaller phenomenological studies and larger aggregated reviews were therefore coded on an equal per-study basis rather than weighted by participant number.

Secondary evidence sources (19, 24, 26, 27, 45) were included to broaden the thematic landscape of chronic pain and were analytically classified as aggregated studies. Sensitivity analyses were conducted with and without these large, aggregated reviews. The major domains and higher-order themes remained stable across weighting scenarios, indicating that the final interpretive structure was not driven solely by large-sample evidence reviews. Full procedures and alternative weighting scenarios are reported in the Supplementary materials. Two reviewers (SSA, EAV) independently coded the included studies, and disagreements were reviewed by a third reviewer (PD) and resolved through discussion and team consensus. The evidence base consisted primarily of primary qualitative studies of lived experience, supplemented by a smaller number of qualitative evidence syntheses. Larger evidence syntheses and meta-ethnographies were consulted but were not given extra weight in theme generation; all themes were retained and interpreted contextually across sources. We performed sensitivity analyses (reported in Supplementary Tables) to confirm that inclusion of broader qualitative reviews did not produce redundant or spurious themes, and to ensure themes derived from primary studies remained robust. De-identified extraction data, coding tables, audit trails, and the results of sensitivity testing are provided in the Supplementary materials to support transparency and reproducibility.

### 2.6 Reflexivity and team contribution to analysis

Reflexivity was treated as an ongoing collaborative analytic practice rather than a discrete final step (38, 39). The disciplinary diversity of the team was used intentionally to challenge assumptions, test interpretations, and strengthen conceptual validity (35–38). SSA and EAV led screening, extraction, and initial coding, while the broader team contributed to peer debriefing, codebook refinement, conceptual mapping, discrepancy adjudication, and final synthesis interpretation.

### 2.7 Ethical considerations

As a systematic review, this study did not involve direct human participation or collection of personal data. The review followed ethical standards for evidence synthesis, including transparency, accuracy, appropriate citation, and best practices in data extraction, appraisal, and interpretation. Potential bias was minimized through use of a predefined protocol, dual screening, structured appraisal tools, team-based adjudication, and maintenance of an audit trail.

## 3 Results

### 3.1 Sequential synthesis overview

The sequential synthesis is presented in the order in which the analytic framework was developed. In phase 1, hermeneutic phenomenological synthesis of the included qualitative literature generated 115 phenomenological subthemes, which were consolidated into 11 hermeneutic domains. In phase 2A, these hermeneutic domains were re-examined through ontological heuristic analysis, resulting in 56 ontological heuristic domains for chronic pain and 54 ontological heuristic domains for chronic illness. These were then organized into 10 overarching ontological domains for chronic pain and eight overarching ontological domains for chronic illness. In phase 2B, a separate review of the transdisciplinary literature identified nine primary principles of transdisciplinarity, which were mapped onto the ontological framework to support clinically relevant pathway development. Detailed coding procedures, appraisal materials, weighting procedures, and supplementary analytic tables are provided in the (Supplementary material).

### 3.2 Phase 1: hermeneutic phenomenology

Phase 1 synthesized the lived experiences of chronic illness and chronic pain reported across the included phenomenological, qualitative, and qualitative evidence synthesis studies. Through iterative coding, comparative analysis, and hermeneutic interpretation, 115 phenomenological subthemes were identified and grouped into 11 hermeneutic domains representing the principal experiential dimensions described in the literature.

These findings showed that chronic illness and chronic pain were not experienced as isolated symptoms or discrete biomedical entities, but as multidimensional lived processes shaped by bodily, psychological, social, cultural, and temporal conditions. Multiple subthemes frequently co-occurred within individual studies, indicating that the experience of chronicity was consistently relational and interconnected across the evidence base.

**Figure 1:**
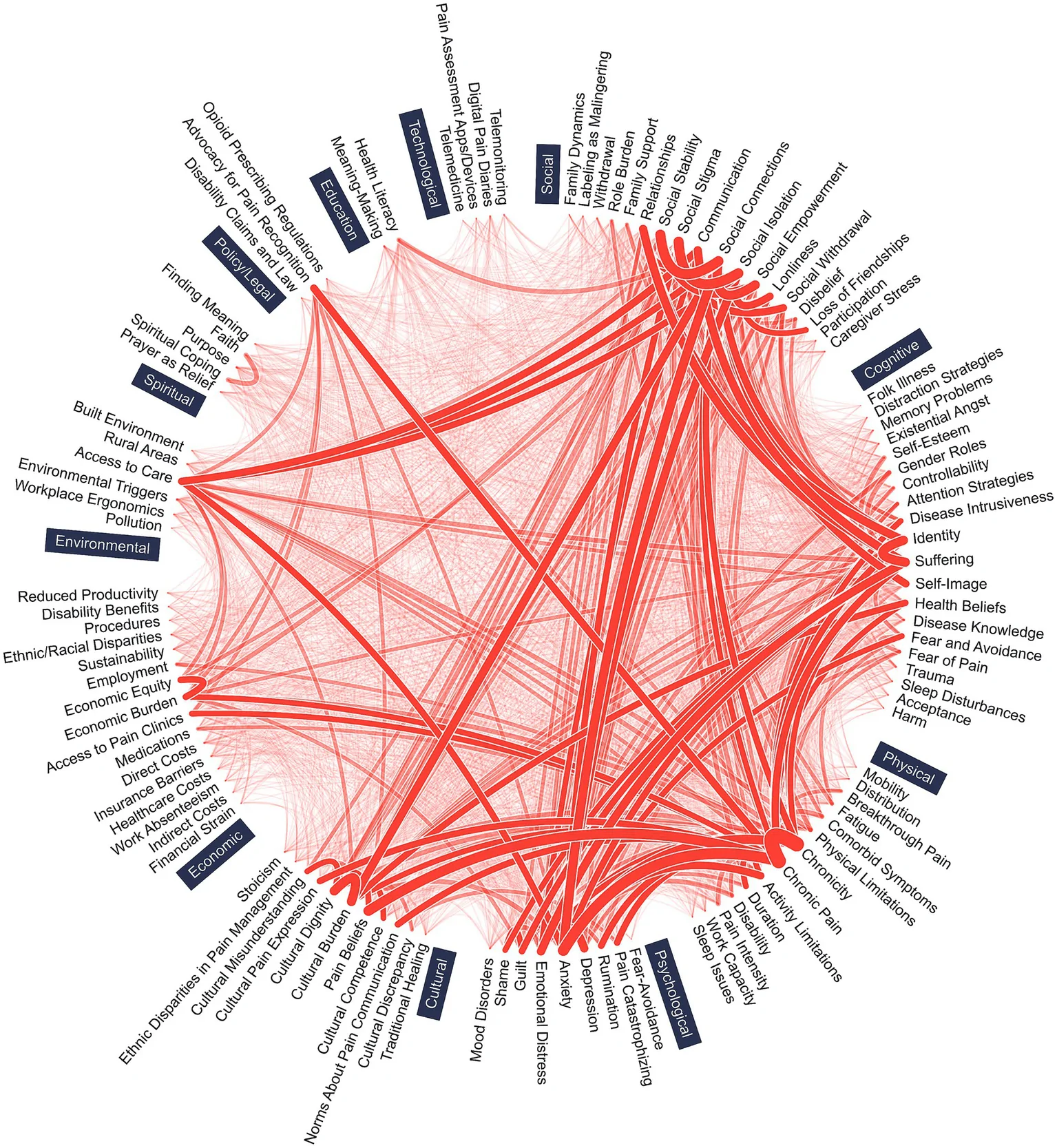
Chronic illness hermeneutic relationship circle. This circular plot visualizes the phase 1 hermeneutic phenomenological synthesis. The outer layer displays 115 phenomenological subthemes grouped into 11 hermeneutic domains derived from 104 included phenomenological studies and qualitative evidence syntheses. Inner links indicate co-occurrence between pairs of subthemes within individual studies, with thicker links representing more frequent co-occurrence across the literature. The figure illustrates the dense interconnections among experiential dimensions of chronic pain and chronic illness, particularly across social, physical, psychological, and temporal aspects of lived experience. Additional coding details and full subtheme lists are provided in Supplementary Tables S3, S4 and Supplementary Figure S2.

Figure 1 presents the network structure of these phenomenological findings. Its contribution lies not only in displaying the 115 subthemes and their organization within the 11 hermeneutic domains, but also in illustrating how these experiential dimensions clustered across the literature. Strong relationships were identified among pain, illness temporality, suffering, physical limitation, anxiety, identity-related disruption, and social experience. Social subthemes such as communication, social isolation, and social stability were especially interconnected, suggesting that social relationships were closely tied to vulnerability, altered identity, and disruption of social roles. The co-occurrence of multiple subthemes within individual studies therefore reflected the strength and consistency of these associations across the literature.

Taken together, phase 1 established that chronic pain and chronic illness are best understood, at the phenomenological level, as densely interconnected experiential processes rather than as separate or static clinical categories. These findings provided the interpretive basis for the second phase of the sequential synthesis (see Table 1).

**Table 1:** Sequential synthesis process and protocol.

Phase	Step	Aim/focus	Methods/tools	Key actions	Outputs
Phase 0	0.1	Interdisciplinary team formation and conceptual orientation	Team expertise across nursing, qualitative methods, pain medicine, neurosurgery, psychology, sociology, data visualization, and engineering	Assemble the team and establish shared conceptual orientations, including biopsychosocial, phenomenological, disability, and systems perspectives	Agreed conceptual framework
Phase 0	0.2	Define review aim, scope, and synthesis approach	Meta-ethnography, hermeneutic analysis, heuristic simplification, graph-based visualization	Specify the review focus, populations, and analytic approach	Review design and protocol structure
Phase 0	0.3	Formulate review questions and coding strategy	Structured protocol development	Develop primary questions on lived experience, meaning, identity, and coping; establish the initial coding approach	Review questions and preliminary coding framework
Phase 0	0.4	Systematic search and study selection	Database searching using MeSH terms, controlled vocabulary, and keywords	Conduct searches, apply inclusion and exclusion criteria, and document the selection process	Final study set and PRISMA flow diagram
Phase 0	0.5	Quality appraisal	CASP; GRADE-CERQual evidence profiling	Assess methodological quality and confidence in evidence	Appraisal tables and evidence profiles
Phase 1	1.1	Data extraction and familiarization	Structured extraction tables; qualitative coding	Extract study characteristics, methods, and findings; begin analytic familiarization	Data extraction tables and initial code set
Phase 1	1.2	Hermeneutic interpretation	Iterative reading; reflexive memoing	Re-read studies, compare interpretations, and document analytic reflections	Interpretive memos
Phase 1	1.3	Meta-ethnographic translation	Noblit and Hare’s meta-ethnographic approach	Conduct reciprocal and refutational translation across studies	Preliminary conceptual categories
Phase 1	1.4	Hermeneutic domain development	Comparative coding and clustering	Consolidate phenomenological subthemes into higher-order hermeneutic domains	115 phenomenological subthemes and 11 hermeneutic domains
Phase 1	1.5	Network visualization of phase 1 findings	d3.js graphing tools	Construct the subtheme co-occurrence network	Hermeneutic relationship circle
Phase 1	1.6	Third-order interpretation	Hermeneutic synthesis; visual analytic review	Develop integrative interpretations and conceptual narrative	Narrative synthesis and conceptual diagrams
Phase 1	1.7	Confidence assessment	GRADE-CERQual	Assess confidence in synthesized findings	Evidence profiles
Phase 2A	2.1	Ontological heuristic re-coding	Heuristic framework; comparative coding	Re-code hermeneutic findings into causal, contextual, relational, and process-based structures	Initial ontological heuristic coding set
Phase 2A	2.2	Ontological domain refinement	Iterative clustering; collaborative discussion	Refine and group ontological heuristic domains by chronic pain and chronic illness	56 ontological heuristic domains for chronic pain and 54 for chronic illness
Phase 2A	2.3	Overarching ontological structuring	Heuristic simplification	Group heuristic domains into higher-order ontological categories	10 overarching ontological domains for chronic pain and eight for chronic illness
Phase 2A	2.4	Ontological framework visualization	d3.js radial network graph	Visualize relations among overarching and heuristic ontological domains	Radial ontological network graph
Phase 2B	2.5	Identification of transdisciplinary principles	Separate transdisciplinary literature review	Identify recurrent principles relevant to chronic pain and chronic illness	Nine primary transdisciplinary principles
Phase 2B	2.6	Mapping principles to the ontological framework	Matrix construction; co-occurrence analysis	Map transdisciplinary principles to ontological domains and assess patterns of representation	Domain–principle co-occurrence matrix
Phase 2B	2.7	Integrative synthesis and reporting	Hermeneutic integration; transparency and reproducibility procedures	Integrate ontological and transdisciplinary findings and prepare the final framework and reporting materials	Final integrated framework, figures, and audit trail

*Supplementary materials provide detailed coding procedures, full subtheme and domain lists, study-by-code matrices, search strategies, PRISMA documentation, quality appraisal tables, GRADE-CERQual evidence profiles, reproducibility files, and visualization scripts, including formulas and sensitivity analyses for frequency (F), relevance weighting (R), and combined visualization weights (W).

### 3.3 Phase 2A: identification of heuristic ontology

The transition from hermeneutic domains to ontological heuristic domains constituted the principal interpretive step in the sequential synthesis. Whereas the 11 hermeneutic domains remained closely anchored in lived-experience description, phase 2A re-examined these findings to identify recurring causal, contextual, relational, and process-based structures that could support a more clinically transferable conceptual framework.

To achieve this, two researchers independently (SSA, EAV) re-coded the hermeneutic findings using a heuristic framework. Through iterative comparison, clustering, and collaborative discussion among the authors, the phenomenological material was reorganized into ontological heuristic domains. This process yielded 56 ontological heuristic domains for chronic pain and 54 ontological heuristic domains for chronic illness. These were then further grouped into 10 overarching ontological domains for chronic pain and eight overarching ontological domains for chronic illness (See Supplementary Tables for coding process). This stage of the synthesis represented a second-order interpretive reclassification rather than a direct restatement of the original phenomenological findings. The ontological heuristic domains were developed to capture dynamic patterns, underlying conditions, and clinically meaningful structures within the data. Although several domains overlapped across chronic pain and chronic illness, some remained condition-specific. For example, opioid prescription and regulation emerged as a distinct domain relevant to chronic pain, whereas most other domains were broadly congruent across both conditions.

**Figure 2:**
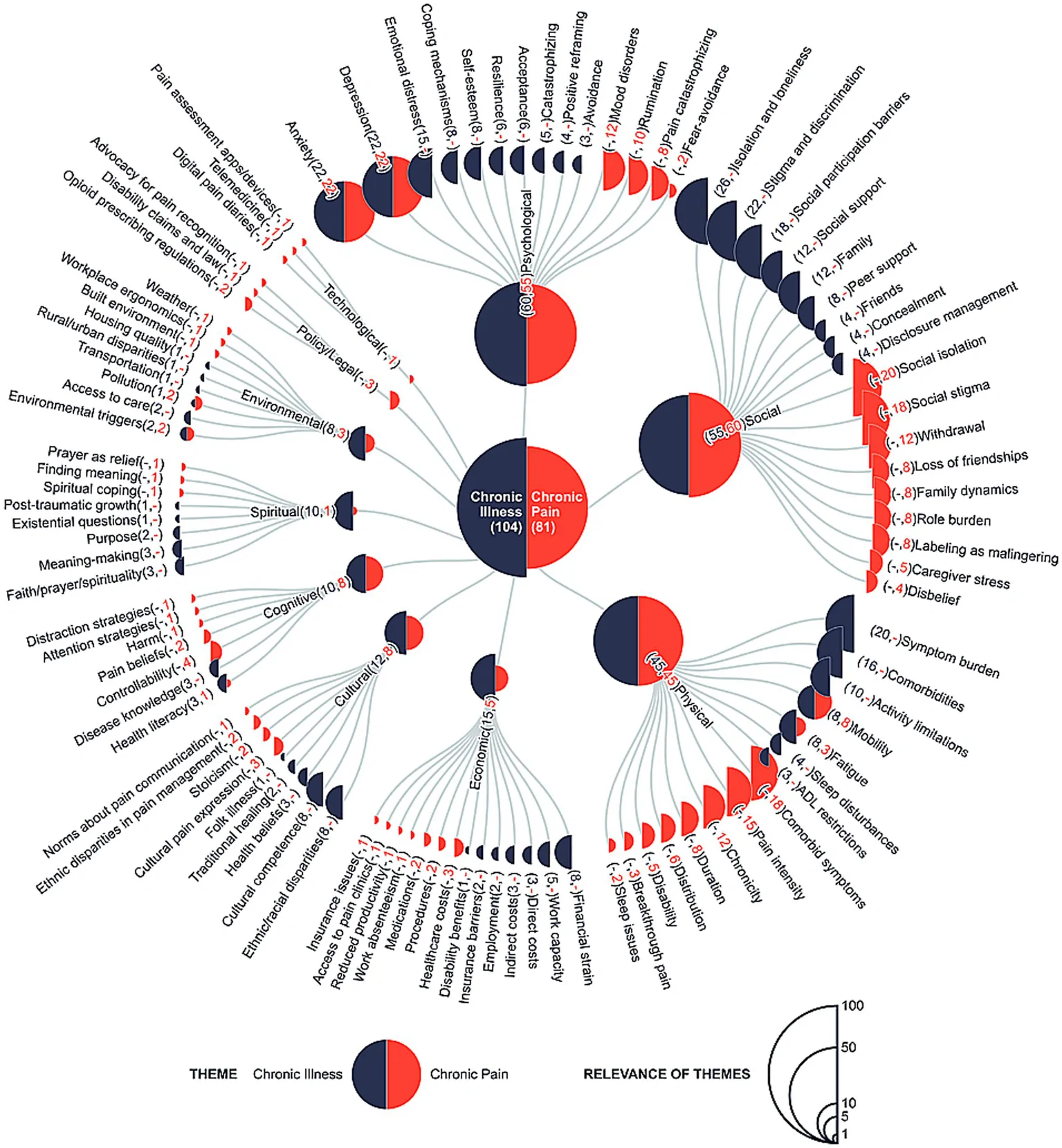
Radial network graph of ontological heuristic domains. This radial network graph presents the phase 2A ontological heuristic reorganization of the phenomenological findings. Inner nodes represent overarching ontological domains, and outer nodes represent the associated ontological heuristic domains derived from the phase 1 synthesis. Node size reflects coded relevance based on the synthesis and collaborative author review. The analysis identified 56 ontological heuristic domains for chronic pain and 54 for chronic illness, which were grouped into 10 overarching ontological domains for chronic pain and eight for chronic illness. Connecting arcs indicate relational links among domains and illustrate the process-oriented view that chronic pain and chronic illness emerge through dynamic interactions among multiple social, physical, psychological, cognitive, cultural, economic, environmental, and contextual conditions. This figure provides a structured overview of the ontological framework developed from the synthesis.

Figure 2 presents this ontological heuristic reorganization. More specifically, it illustrates how the phenomenological findings were translated into a process-oriented ontological structure for chronic pain and chronic illness. The figure shows that these conditions arise through dynamic interrelations among multiple domains rather than through fixed or isolated mechanisms. Social, physical, and psychological domains showed the greatest prominence, while cognitive, cultural, economic, environmental, and spiritual domains also contributed substantively to the final structure. The significance of Figure 2 lies in demonstrating that chronic pain and chronic illness are best understood as variable ontological processes shaped by interacting causes and conditions.

This ontological heuristic structure simplified the organization of the phenomenological findings while preserving their complexity, thereby creating a conceptual bridge between lived-experience synthesis and transdisciplinary clinical application.

### 3.4 Phase 2B: identification of clinical transdisciplinary pathways

In phase 2B, the ontological heuristic framework was mapped against principles identified from the transdisciplinary literature in order to support clinically relevant pathway development. A separate review was conducted to identify principles of transdisciplinarity most relevant to chronic illness and chronic pain within the domain structure established in phase 2A.

This analysis identified nine primary principles of transdisciplinarity. These principles were selected because they recurred consistently across the transdisciplinary literature, aligned conceptually with chronic illness and chronic pain as complex multidomain phenomena, and demonstrated the strongest interpretive fit with the ontological heuristic domains established in the preceding phase. They were therefore included as the most analytically salient principles for the framework developed in this study, rather than as an exhaustive representation of all transdisciplinary principles described in the broader literature.

**Figure 3:**
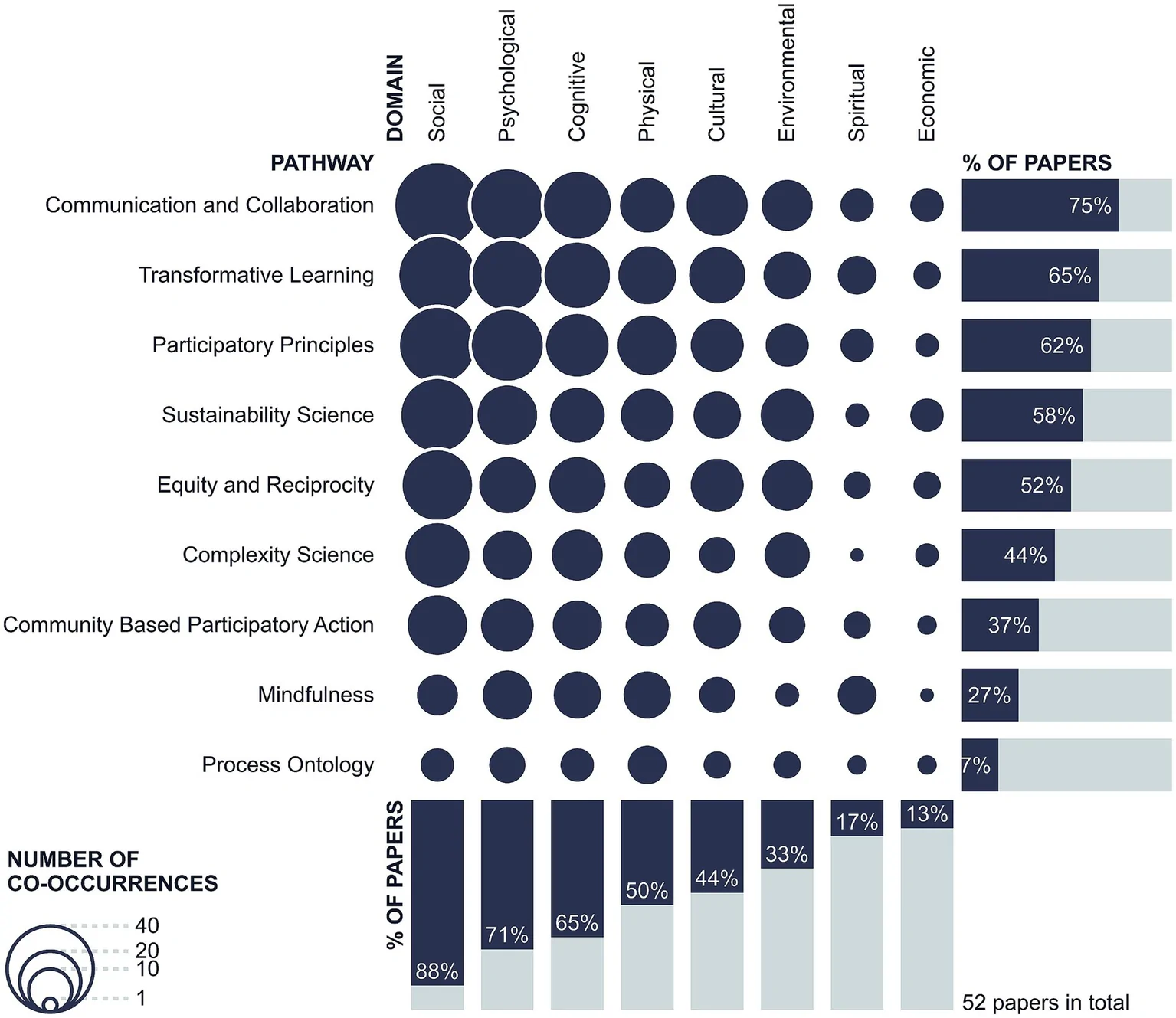
Matrix of ontological domains and transdisciplinary principles. This matrix presents the phase 2B mapping of transdisciplinary principles onto the ontological framework. Columns represent the ontological domains identified in phase 2A, and rows represent the nine primary transdisciplinary principles identified in the separate transdisciplinary review. Each cell contains a node whose size reflects the co-occurrence of a given principle and ontological domain within the included transdisciplinary literature. The matrix shows where transdisciplinary principles were most strongly represented across the ontological structure, with communication, collaboration, and participatory practice emerging as especially prominent. This visualization supports practical interpretation by identifying areas of stronger and weaker integration relevant to clinical pathway development.

Figure 3 presents the mapping of these nine principles onto the ontological framework. Its significance lies in showing that the final framework is not only descriptive, but also clinically and analytically applicable. Communication, collaboration, and participatory practice emerged as especially central principles, highlighting the importance of shared engagement across disciplines, stakeholders, and communities. The review also identified reflexivity, mindful reflection, complexity, and sustainability as important principles for addressing chronic illness and chronic pain within dynamic and interdependent systems of care. These findings indicate that transdisciplinary implementation depends not only on the integration of knowledge across fields, but also on sustained attention to relationships, shared learning, and adaptive coordination across clinical and social contexts.

Taken together, phase 2B translated the ontological framework into a transdisciplinary structure for clinical interpretation and pathway development.

### 3.5 Interpretive summary

Across the sequential synthesis, the analytic progression moved from 115 phenomenological subthemes to 11 hermeneutic domains, then to 56 ontological heuristic domains for chronic pain and 54 ontological heuristic domains for chronic illness, which were further grouped into 10 overarching ontological domains for chronic pain and eight overarching ontological domains for chronic illness. These final ontological structures were then mapped against nine primary transdisciplinary principles.

Overall, the findings indicate that chronic illness and chronic pain are not adequately represented as static biomedical entities. Rather, they are more accurately understood as dynamic, relational, and multidimensional processes shaped by interacting ontological conditions. The final framework therefore provides a phenomenologically grounded, ontologically structured, and transdisciplinarily oriented basis for clinical interpretation and application.

## 4 Discussion

This study develops a heuristic ontology of chronic illness, chronic pain, and multimorbidity grounded in a sequential synthesis of phenomenological, ontological, and transdisciplinary literatures. By heuristic ontology, we mean a practical interpretive framework for organizing and making sense of a complex phenomenon rather than a formal ontology intended for taxonomic standardization or computational representation. This distinction is important because the framework proposed here is not designed to replace biomedical classifications or disease-specific diagnostic models, but to support interpretation, communication, and application in relation to chronic illness and chronic pain as they are lived and managed across time and context.

The central argument of the study is that chronic illness, chronic pain, and multimorbidity are not best understood as isolated disease entities or static symptom aggregates, but as temporospatial, relational, and processual patterns. Process ontology is relevant because it understands reality in terms of dynamic, unfolding, and interacting processes rather than fixed substances with stable properties (46–49). Relational ontology similarly shifts attention from self-contained entities to phenomena constituted through relations, interactions, and dependencies (48, 50, 51). The temporospatial dimension adds that chronic illness unfolds through time and is expressed through embodied, interpersonal, and environmental contexts rather than at a single static point. Taken together, these concepts provide the ontological basis for the framework developed in this review.

Bringing these strands together is not simply an exercise in theoretical expansion. The findings of the synthesis indicate that chronicity is consistently organized through interdependence. In phase 1, phenomenological synthesis identified 115 subthemes grouped into 11 hermeneutic domains, with strong co-occurrence among bodily suffering, altered temporality, identity disruption, anxiety, social isolation, communication difficulties, physical limitation, and changing social participation. These patterns suggest that chronic illness and chronic pain are not lived as separable clinical components, but as interwoven modes of existence that shape how individuals move, relate, interpret, anticipate, and function. Phenomenology is particularly important here because it renders visible illness as lived and embodied rather than merely observed or measured (52). It also clarifies that pain is not simply a symptom attached to disease categories, but an experience that reorganizes time, embodiment, agency, and social relation.

The ontological reorganization developed in phase 2A reinforces this interpretation by showing that the phenomenological structure of chronicity can be translated into a process-oriented framework. The resulting ontological domains do not merely redescribe experiential findings; they identify recurring conditions, relations, and patterns through which chronic illness and chronic pain emerge and persist. This is where process ontology is especially useful. If chronic illness is understood as unfolding rather than static, then clinical interpretation must attend to change, recurrence, interaction, accumulation, and adaptation rather than relying only on cross-sectional symptom description or disease counts (46–49).

Temporospatiality is especially important in this regard. Chronic illness is not defined only by persistence, but by trajectory. It evolves through duration, fluctuation, recurrence, progression, remission, exacerbation, and cumulative treatment burden. Symptoms such as pain are also spatially expressed, not only within the anatomical body, but through posture, movement, psychomotor change, interpersonal interaction, environmental fit, and everyday functioning (46–48, 53–57). This is particularly important in multimorbidity, where conditions interact with one another, with treatment regimens, and with wider determinants over time (53–55, 58, 59). A temporospatial perspective therefore shifts clinical attention from disease count alone to illness trajectory, symptom patterning, and the interaction of conditions across lived contexts.

The clinical implications of this shift are direct. If chronic illness is temporospatial and processual, then assessment should extend beyond diagnosis to include symptom fluctuation, treatment burden, functional change, relational impact, and relevant social and environmental context. Care planning should be longitudinal and adaptive rather than based exclusively on condition-specific protocols. Symptom interpretation should account for how pain, fatigue, mood, cognition, and function cluster and change over time rather than treating each in isolation. Multimorbidity should likewise be approached as an interacting configuration rather than a simple sum of conditions. These implications are not added externally to the framework; they follow from the ontology that the synthesis supports.

This orientation is consistent with the broader evidence base. Psychosocial factors remain among the strongest predictors of pain experience and pain distribution, indicating that chronic pain cannot be reduced to biomarker profiles alone (9). Research on multimorbidity increasingly relies on temporal patterning to identify clinically meaningful trajectories that cannot be captured by diagnosis count in isolation (53, 54). Phenomenological work further supports this emphasis by showing that pain is lived as embodied and temporal rather than as a detached symptom attached to disease categories (52). Mechanistic explanations therefore remain necessary, but they should be understood as partial abstractions from broader ongoing processes rather than exhaustive accounts of illness reality (48–50).

Relationality is equally central to the framework. At the embodied level, illness is experienced through changing relations among pain, mood, movement, cognition, sleep, fatigue, and function. At the interpersonal level, chronic illness frequently reorganizes family life, intimate relationships, and everyday social participation, especially when symptoms are invisible, difficult to articulate, or inconsistently recognized by others (60, 61). At the systems level, illness is shaped by encounters with clinicians, organizational pathways, service fragmentation, and structural inequalities that affect recognition, access, continuity, and ongoing management (1, 3, 60, 61). The framework therefore treats illness as constituted through relations across biological, experiential, interpersonal, sociocultural, and institutional domains rather than through any one level alone.

This emphasis is especially important in chronic pain and multimorbidity because these conditions rarely remain confined to the individual body. Pain may intensify psychological distress, constrain activity, alter identity, and increase risk of depression; while coexisting pain and depression are associated with poorer outcomes, higher healthcare use, and lower health-related status than either condition alone (1, 3, 4). These interactions are also shaped by socioeconomic conditions, showing that chronic illness is produced through both psychophysical and structural processes (1, 3, 60, 61). A heuristic ontology that does not account for these relations risks reproducing the fragmentation that complex chronicity resists.

An important contribution of the present framework is that it does not stop at phenomenological or ontological description, but links ontological domains to transdisciplinary principles that make implementation more explicit. The principles identified in phase 2B, particularly communication, collaboration, participation, reflexivity, and systems thinking, should not be understood as supplementary values added after the analytic work was complete. Rather, they follow directly from the ontological structure of the framework itself.

If illness unfolds over time, then communication becomes essential for interpreting trajectories across settings, professions, and episodes of care. If illness is embodied and situated, then participation becomes indispensable because bodily experience, symptom meaning, function, and environmental fit cannot be adequately understood without patient involvement. If illness is relational and socially distributed, then collaboration is necessary because management involves patients, families, clinicians, and multiple professional perspectives that must be coordinated rather than merely collocated. If illness is shaped by norms, expectations, institutions, and inequalities, then reflexivity becomes essential because interpretation and intervention are never neutral, but are shaped by assumptions, power relations, and structural conditions. Systems thinking cuts across all these domains because chronic illness care is characterized by nonlinearity, feedback, context dependence, and adaptive change (54, 55, 58, 59, 62–68).

The cross-sectional matrix developed in phase 2B is intended to make these relationships explicit. Its purpose is not merely illustrative. Rather, it operationalizes the framework by showing how ontological commitments imply practical requirements for inquiry and care. The temporal domain implies longitudinal communication and repeated reinterpretation over time. The embodied and spatial domain implies participatory attention to lived experience, function, and environment. The interpersonal domain implies collaboration among patients, families, and professions. The sociocultural domain implies reflexivity regarding norms, institutions, and inequalities. Systems thinking links all domains by foregrounding interaction, adaptation, and implementation across changing contexts (54, 55, 58, 59, 62–68). In this sense, the matrix translates conceptual claims into practical orientations without reducing complexity to a checklist.

The contribution of the proposed heuristic ontology becomes clearer when situated alongside existing models. It does not replace biomedical approaches, which remain essential for diagnosis, pathophysiology, risk stratification, and treatment selection. Nor does it reject biopsychosocial thinking, which has been important in broadening attention beyond purely biological explanation. Rather, the present framework complements these models by providing a more explicit ontological organization of chronic illness as temporospatial, relational, and processual.

Compared with condition-specific biomedical models, the framework better captures interacting trajectories, cumulative treatment burden, and context-sensitive variation in multimorbidity (53–55, 58, 59). Compared with general biopsychosocial formulations, it offers a more structured account of how embodiment, temporality, interpersonal relations, and sociocultural conditions are linked within a single interpretive model. Its contribution therefore lies less in introducing entirely new domains than in integrating them more coherently and translating them more directly into clinically relevant and transdisciplinary terms. The theoretical breadth of the framework also requires clear discipline so that range does not become diffuseness. In this review, phenomenology is used specifically to clarify lived embodiment and temporality (52). Process ontology is used to explain why chronic illness can be understood as unfolding, emergent, and dynamic rather than static (46–49). Relational ontology supports the view that illness is constituted through dependencies and interactions rather than existing as a self-contained object (48, 50, 51). Neuro-ecological, ecological, and enactive perspectives are included only insofar as they reinforce the context-sensitive and relational character of perception, action, and experience (17, 47, 57, 69–77). Their role is not to create parallel theoretical tracks, but to converge on the same interpretive claim: chronic pain and chronic illness are not fully captured by static disease categories because they emerge through ongoing organism-environment interactions. Systems and transdisciplinary literatures then connect this ontological account to collaboration, reflexivity, implementation, and adaptive care structures (58, 59, 62–68). This framing is important because it keeps the theoretical framework aligned with the central purpose of the study for clinical applicability and clarifies the distinct function each strand.

The practical consequence of this integrated model is that it supports clinical reasoning in multimorbidity and chronic pain by encouraging trajectory-based assessment, attention to relational burden, participatory interpretation of lived experience, interdisciplinary communication, and context-sensitive planning. These implications are consistent with the proposed heuristic ontology and help show why a temporospatial and relational account is not only conceptually coherent but clinically relevant.

## 5 Future directions

Future work should focus on empirical evaluation of the proposed heuristic ontology in clinical and service contexts. Studies are needed to assess whether the framework can usefully inform assessment, care planning, interdisciplinary communication, and longitudinal management in chronic illness, chronic pain, and multimorbidity. Initial efforts may be most productive in smaller, context-specific studies designed to examine feasibility, usability, and implementation conditions across care environments.

Further work is also needed to develop and evaluate clinician-facing educational approaches. If the framework is to have practical value, clinicians will require support in applying temporally sensitive, relational, and systems-oriented modes of reasoning in routine care. Relevant areas for development include longitudinal clinical reasoning, interpretation of treatment burden and symptom interaction, interdisciplinary communication, engagement with patient-reported experience, and reflexive awareness of sociocultural and structural influences on care.

Future transdisciplinary research would benefit from more explicit use of participatory co-design methods. Patients, caregivers, clinicians, and community stakeholders could be involved not only in contributing experiential knowledge, but also in refining conceptual domains, identifying implementation priorities, and interpreting findings. Potential approaches include stakeholder workshops, advisory panels, consensus-oriented methods, and iterative development of tools or pathways for use in practice. Such work would help assess the framework’s relevance, acceptability, and adaptability beyond the present synthesis.

Longitudinal qualitative research may also clarify how meaning, identity, symptom interpretation, and relational burden shift over the course of illness and treatment. In addition, further study is needed to examine the transferability of the framework across healthcare systems and socioeconomic settings, including environments where transdisciplinary implementation is constrained by limited resources or fragmented service organization. Closer engagement with recent implementation science and integrated care literature may help guide this next phase of development.

### 5.1 Limitations

Several limitations should be considered when interpreting this study. First, the synthesis is based on published literature and may therefore be affected by publication bias. Studies offering clearer conceptual convergence or more readily translatable findings may be more likely to enter the published record than studies reporting ambiguity, inconsistency, or less easily synthesized accounts. The framework presented here should therefore be understood as a structured interpretive synthesis rather than a comprehensive or definitive account of the field.

Second, the proposed heuristic ontology is inherently interpretive. This is consistent with the study’s hermeneutic and transdisciplinary orientation, but it also means that the framework is shaped by analytic judgment and may reflect the assumptions and positioning of the research team. We sought to strengthen the trustworthiness of the analysis through collaborative coding, reflexive discussion, and triangulation across sources. Even so, interpretive variation remains possible. In this context, the value of the framework rests less on formal reproducibility than on conceptual clarity, transparency, and usefulness for further inquiry and application. Its stability and applicability will require further examination through case-based use, stakeholder appraisal, and iterative refinement.

Third, the study relies on secondary qualitative material and does not incorporate primary interviews or observational data. This may limit contextual depth, particularly in relation to underrepresented populations, local care practices, and institutional settings not well captured in the available literature. Relatedly, the conceptual foundations of the framework draw primarily on Western philosophical traditions, including phenomenology and process-oriented thought. Although these traditions offer important analytic resources, they may not fully capture culturally specific understandings of embodiment, suffering, relationality, illness, and care. Further work is needed to examine how the framework aligns with, or requires modification in relation to, other epistemic and cultural contexts.

Fourth, the transferability of the framework across healthcare systems and socioeconomic settings remains uncertain. The organization of care, availability of interdisciplinary resources, funding structures, and broader social determinants of health vary considerably across settings. These differences are likely to shape both the salience of ontological domains and the feasibility of transdisciplinary implementation.

Finally, implementation of the proposed approach is likely to be challenging in routine practice. Transdisciplinary models depend on coordination across professional boundaries, institutional support, stakeholder engagement, and sufficient time and material resources. These conditions may be difficult to secure, particularly in resource-constrained environments characterized by workforce pressures, fragmented care pathways, reimbursement limitations, and competing service demands. The present study should therefore be read as offering a conceptual foundation for further development rather than an immediately deployable model of care.

## 6 Conclusion

This study synthesizes qualitative literature on chronic illness and chronic pain and proposes a heuristic ontology for interpreting these conditions as temporally unfolding, relational, and processual phenomena. Through a sequential transdisciplinary approach informed by hermeneutic phenomenology, the analysis highlights the interdependence of somatic, psychological, social, and cognitive dimensions of illness experience.

The synthesis suggests that domains such as social isolation, identity disruption, physical limitation, and cognitive distress do not occur in isolation, but interact over time and within broader sociocultural and institutional contexts. The heuristic ontology was developed to render these interdependencies more conceptually explicit and to support a more integrated understanding of chronic illness complexity, particularly in relation to long-term care and multimorbidity.

The study’s contribution lies primarily in offering a structured interpretive framework that may complement existing biomedical and biopsychosocial models by making temporality, relationality, embodiment, and emergence more analytically visible. The accompanying cross-sectional matrix extends this contribution by linking ontological domains with transdisciplinary principles, including communication, collaboration, participation, reflexivity, and systems thinking.

The potential implications are practical as well as conceptual. For clinicians, the framework may support broader attention to illness trajectories, treatment burden, lived experience, and relational context. For policymakers and service planners, it may help clarify why chronic illness care often requires models capable of coordination, adaptation, and contextual responsiveness. For researchers, it offers a basis for further empirical refinement, participatory development, and implementation-focused study across diverse care settings.

## Data Availability

The original contributions presented in the study are included in the article/Supplementary material, further inquiries can be directed to the corresponding author.

## Footnotes

1
https://www.casp-uk.net
