# Supplementary material for: A transdisciplinary framework for chronic illness and chronic pain: a sequential qualitative meta-synthesis
Source: Front Public Health. 2026 Aug 12;14:1914023. doi: 10.3389/fpubh.2026.1914023 (PMC13506920; doi:10.3389/fpubh.2026.1914023)
Supplement: Supplementary file 1 [file Data_Sheet_1.docx]

Supplementary Material

# 1 Supplementary Figures

**Supplementary Figure S1.** **PRISMA Flow Diagram of the Study Selection Process**

**Studies Selection Process**

**Identification**

Records identified through database search:

PubMed (n=588), Google scholar (n=157), Scopus (n=25), AMED (n=15), Cochrane (13), Web of Science (n=87), PsycINFO (n=277), ProQuest (3,350)

**3,742** duplicates and irrelevant titles were identified and excluded

**Screening**

**432** irrelevant studies were excluded

Records screened by titles and abstract (**n=770**)

**182** studies excluded

- Inappropriate population: Participant’s age (adults (<18 years)
- Irrelevant focus/ topic: Studies not addressing the lived experiences, perceptions, or personal narratives of individuals with chronic pain or chronic illness
- Studies not related to transdisciplinary science
- Not published between 2010 and 2025
- Conference abstracts, dissertations, commentaries, book chapters
- Irrelevant design/ methodology
- Inadequate reporting
- Inappropriate setting/ context: The study was not conducted within the defined context or population parameters; acute setting, acute pain.
- Lack of reflexivity
- Disciplinary siloing
- Conceptual mismatch

(**n=338**) full-text studies assessed for eligibility

**Eligibility**

**Included**

**(n=104)** studies included in hermeneutic phenomenology

**(n=52)** studies included in transdisciplinary analysis

**Supplementary Figure S2. Studies Selected for Inclusion in Hermeneutic Phenomenology: Stage 1 of the Synthesis**

Of the 104 studies, 42 were phenomenological, 23 were interpretive phenomenological analyses, 16 were qualitative interview studies, 15 were meta-ethnographic, and 8 were qualitative evidence reviews. These studies included 517, 230, 482, 16,239, and 144,707 patients, respectively, totaling 162,175 patients across 28 countries.

# 2 Supplementary Tables

**Supplementary Table S1. Detailed Protocol for the Sequential Synthesis and Coding Workflow/Audit Information**

| Phase | Activity | Tools/Methods | Reviewers/Authors | Criteria/Protocol | Domains/Themes | Outcome |
| --- | --- | --- | --- | --- | --- | --- |
| Phase 0 | Pre-synthesis Stages | Five-step protocol | SSA, PD, RT | Enhance reproducibility, methodological rigor | Key themes/ domains | Informed subsequent synthesis phases |
| Phase 0 | Keywords and Search Strings | MeSH, controlled vocabulary, free-text terms | SSA, EAV, PD | Adapted for PsycINFO, PubMed, Scopus, Google Scholar, ProQuest, Web of Science | - | Literature published Jan 2009-Dec 2025 |
| Phase 0 | Initial Data Collection | Structured data extraction form | SSA, PD, RT | Independently tested, oversight for consistency | Study characteristics, participant details | Extracted data: authorship, year, country, methodology, framework, findings |
| Phase 0 | Certainty of Evidence and Critical Appraisal | MMAT, CASP | EAV, SSA, PD | CASP score 0–10, minimum threshold 70% | Risk bias, methodological rigor, clarity, relevance | Studies included if the threshold is met |
| Phase 0 | Subjective Experiences Analysis | GRADE-CERQual | EAV, SSA | Four components: methodological limitations, coherence, adequacy, relevance | Confidence graded: high, moderate, low, very low | Discrepancies resolved by consensus |
| Phase 0 | Ethical Considerations | Ethical guidelines, IRB approval, participant confidentiality | EAV, SSA | Transparency, accuracy, best practices | Review all studies for IRB approval, participatory principles, interview styles | Bias minimized, citation observed |
| Phase 0 | Initial Data Analysis | CERQual Evidence Profile | EAV, PD, RT | Summary of findings, confidence levels, references | Review-finding groups, initial themes | Themes defined, named, assigned to domains |
| Stage 1 | Collaborative Identification and Definition of Problems | Qualitative Evidence Synthesis | SSA, EAV, RT | Research objectives, academic and societal questions | Conceptual and methodological framework | Collaborative research team assembled |
| Stage 1 | Stakeholder Integration | Phenomenological approach, qualitative literature review | SSA, EAV, RT | Systematic analysis, inclusion and quality criteria | Perspectives: patients, families, clinicians | Findings relevant to sociocultural contexts |
| Stages 1 & 2 | Analytic Rigor and Reproducibility | Thematic synthesis, meta-ethnographic frameworks | SSA, EAV, RT | Structured, multi-stage coding and synthesis | Iterative process | Promote transparency and reproducibility |
| Stages 1 & 2 | Coding Procedure | Seven sequential stages across three phases (0-3) | SSA, EAV, RT, BD, PD, PMR | Familiarization, line-by-line coding, descriptive themes, cross-study translation, analytical themes, domain classification | Social, psychological, physical, cognitive | Final thematic structures by consensus, graph development and visualization |
| Stages 1 & 2 | Resolution of Discrepancies | Iterative discussion, consensus | SSA, EAV, RT, BD, PD, PMR | Agreement estimated 85–90% conceptual alignment | Iterative process | Full-team consensus |
| Stage 2 | Audit Trail and Data Management | Annotated coding tables, version-controlled maps, summaries | SSA, EAV | Document coding decisions, theme development | Circling back to reviewing the coding procedures | Iterative revisions recorded |
| Stage 2 | Reflexivity | Group discussions, mindfulness | Interdisciplinary team | Ongoing reflective discussions | Reflexivity is iterative and continuous | Examined the influence of perspectives and experience |

**Supplementary Table S2. Study Corpus, Search Methodology, Coding Procedures, Reliability Assessment**

| Section | Description |
| --- | --- |
| Source corpus | Qualitative and qualitative-synthesis studies on the lived experience of patients with chronic pain and chronic illness; identified via database searches (PubMed, Scopus, Web of Science, PsycINFO, ProQuest, AMED, Cochrane, Google Scholar). |
| Search strategy | Exhaustive controlled-vocabulary and free-text terms for pain and chronic illness and pain; exact strings and hit counts in Supplementary Table. |
| Inclusion criteria | Primary qualitative studies and qualitative evidence syntheses reporting patient/caregiver lived experiences |
| Exclusion criteria | Purely quantitative studies; non-qualitative empirical studies, protocols, studies without any primary participant accounts. |
| Data management | Exported to reference manager, duplicates removed, full texts saved in version-controlled folder, summary/coding/theme development completed. |
| Coding team | Multidisciplinary analysts: qualitative methodologists, nursing, sociology (RT, EAV), clinician-researchers (SSA, PD, EAV), content experts, engineering (SSA, PD, PR, EAV). |
| Data Visualization | This study sought to elucidate the lived experiences identified in the studies identified in the comprehensive literature review. Accordingly, after establishing the hermeneutic phenomenological framework, we applied heuristic methods to organize the data. The domains and themes were systematically arranged heuristically to facilitate the simplification, organization, and visualization of the findings. |
| Reviewers | Two reviewers (EAV, SSA) independently coded each study; a third reviewer (PD) resolved any disagreements. |
| Consensus | Final coding of themes/subthemes reached through group discussion |
| Training | Pilot coding exercise on 10 diverse papers; reflexivity briefings; written reflexivity log maintained. |
| Codebook development | Iterative, mixed inductive-deductive approach; a priori thematic framework; open coding of 23 papers; codes grouped and mapped to meta-themes; codebook lists label, definition, notes, example quotes, domain/meta-theme mapping; hermeneutic followed by heuristic analytic codes were developed by mapping subthemes from the Chronic Illness and Chronic Pain codebooks into higher‑level (heuristic) themes, recording exact subtheme k‑counts (F) and assigning an ordinal relevance weight (R; 3 = core, 2 = contextual) by independent coders with consensus reconciliation. Primary prominence scores were computed as Worig = F × R, and sensitivity‑adjusted scores as WA = ln(F+1) × R to temper high‑count dominance. To test the robustness, we ran alternate coding scenarios (conservative, balanced, liberal), including leave‑one‑theme and leave‑one‑study analyses, and selectively excluded low‑k subthemes or whole studies to assess how mappings and R assignments affected rankings; all changes, reconciliation notes, and scenario outputs are archived in the project repository and reported in the Supplementary Materials. |
| Coding procedure | Familiarization, line-by-line first-order coding, second-order coding, cross-study translation/synthesis, domain assignment and weighting. Inter-rater reliability: the coding reliability was assessed using raw percent agreement and Cohen’s kappa. Across the coded dataset (n = 111 themes, from 104 studies), reviewers achieved ≥80% raw agreement and Cohen’s kappa ≈0.80 (95% CI [CI0.75-0.85]), indicating substantial agreement beyond chance. Because kappa can be influenced by category prevalence and marginal distributions, we report both the statistics and inspected category frequencies to confirm no undue bias; prevalence‑adjusted checks produced consistent results. Discrepancies were resolved via blinded third‑coder adjudication and consensus team meetings, with all resolution decisions recorded in the audit trail. Full inter‑rater tables, calculation scripts, and item‑level agreement data are provided in supplementary tables. |
| Metadata extraction | Author names, publication year, country, design, sample entered structured extraction form |
| Quality appraisal | CASP (and MMAT for mixed-methods); appraisal items and total score recorded; sensitivity analysis for low-scoring studies (threshold 70%). |
| Confidence assessment | GRADE-CERQual applied to high-order findings; confidence level (high, moderate, low, very low) documented in evidence profile. |
| Reliability & audit trail | Double coding, inter-rater agreement monitored (≥85%), disagreements resolved by consensus, audit logs retained |
| Outputs | Full codebook, study × code matrix, CERQual evidence profiles, weighted domain/subtheme frequency tables, narrative meta-synthesis. |
| Reporting standards | PRISMA-S guidance: search strings, dates, limits, hits, screening flowchart, appraisal results, supplementary files. |
| Ethics/data availability | Published, anonymized data; no new human subject research; extraction sheets and codebooks shared in project repository |
| Notes | The coding procedure integrates empirical frequency with judgement-based weighting. Sensitivity analyses and alternative weighting scenarios are provided, along with the search strings, screening flowchart, comprehensive codebook (including definitions and exemplar quotations), extraction tables, appraisal scores, audit trail, and all relevant materials, which are available in the Supplementary Materials. |

**Supplementary Table S3. Keywords and Search Strings***

| Data base | Search string |
| --- | --- |
| PubMed | PubMed ("Chronic Pain"[MeSH] OR "chronic pain"[tiab] OR "persistent pain"[tiab] OR "long-term pain"[tiab] OR "recurrent pain"[tiab] OR "chronic pain syndrome"[tiab] OR "musculoskeletal pain"[MeSH] OR "musculoskeletal pain"[tiab] OR "low back pain"[MeSH] OR "low back pain"[tiab] OR "neck pain"[tiab] OR neuralgia[tiab] OR neuropath*[tiab] OR "widespread pain"[tiab] OR fibromyalgia[tiab] OR "headache disorders"[MeSH] OR headache[tiab] OR "orofacial pain"[tiab] OR "temporomandibular"[tiab] OR "post-stroke pain"[tiab] OR "chronic pelvic pain"[tiab] OR "chronic postoperative pain"[tiab] OR nocicept*[tiab]) AND ("Chronic Disease"[MeSH] OR "chronic disease"[tiab] OR "chronic illness"[tiab] OR chronic*[tiab] OR "long term illness"[tiab] OR "long-term illness"[tiab] OR longterm[tiab] OR persistent[tiab] OR recurrent[tiab] OR multimorbid*[tiab] OR comorbid*[tiab] OR "long term condition"[tiab] OR "non-communicable disease"[tiab] OR NCD[tiab]) AND (qualitative[tiab] OR "qualitative research"[MeSH] OR "qualitative research"[tiab] OR interview*[tiab] OR "focus group*"[tiab] OR ethnograph*[tiab] OR "thematic analys*"[tiab] OR "content analys*"[tiab] OR "grounded theory"[tiab] OR phenomenolog*[tiab] OR "meta-ethnograph*"[tiab] OR "meta-synth*"[tiab] OR "meta-summar*"[tiab] OR "mega-ethnograph*"[tiab] OR "meta narrative"[tiab] OR "critical interpretive synthesis"[tiab]) AND ("lived experience"[tiab] OR "patient experience"[tiab] OR "illness narrative*"[tiab] OR stigma[MeSH] OR stigma[tiab] OR shame[tiab] OR "self-blame"[tiab] OR guilt[tiab] OR "body image"[tiab] OR "social isolation"[tiab]) AND (phenomenolog*[tiab] OR "Affordance theory"[tiab] OR "cultural affordances"[tiab] OR ontology[tiab] OR "systems thinking"[tiab] OR "systems science"[tiab] OR "complexity science"[tiab] OR "social determinants of health"[tiab] OR participatory[tiab]) |
| Scopus | TITLE-ABS-KEY(("chronic pain" OR "persistent pain" OR "long-term pain" OR "chronic pain syndrome" OR "musculoskeletal pain" OR "low back pain" OR "neck pain" OR neuralgia OR neuropath* OR "widespread pain" OR fibromyalgia OR headache OR "orofacial pain" OR "temporomandibular" OR "chronic pelvic pain" OR nocicept*) AND ("chronic disease" OR "chronic illness" OR chronic* OR "long-term illness" OR longterm OR persistent OR recurrent OR multimorbid* OR comorbid* OR "long term condition" OR "non-communicable disease" OR NCD) AND (qualitative OR "qualitative research" OR interview* OR "focus group*" OR ethnograph* OR "thematic analys*" OR "content analys*" OR "grounded theory" OR phenomenolog* OR "meta-ethnograph*" OR "meta-synth*" OR "meta-summary" OR "meta narrative" OR "critical interpretive synthesis" OR "mega-ethnograph*") AND ("lived experience" OR "patient experience" OR "illness narrative*" OR stigma OR shame OR "self-blame" OR guilt OR "body image" OR "social isolation") AND (phenomenolog* OR "Affordance theory" OR "cultural affordances" OR ontology OR "systems thinking" OR "systems science" OR "complexity science" OR "social determinants of health" OR participatory)) |
| Web of Science | TS=(("chronic pain" OR "persistent pain" OR "long-term pain" OR "recurrent pain" OR "chronic pain syndrome" OR "musculoskeletal pain" OR "low back pain" OR "neck pain" OR neuralgia OR neuropath* OR fibromyalgia OR "widespread pain" OR headache OR "temporomandibular" OR "orofacial pain" OR "chronic pelvic pain" OR nocicept*) AND ("chronic disease" OR "chronic illness" OR chronic* OR "long-term illness" OR longterm OR persistent OR recurrent OR "long-standing" OR multimorbid* OR comorbid* OR "long term condition" OR "non-communicable disease" OR NCD) AND (qualitative OR "qualitative research" OR interview* OR "focus group*" OR ethnograph* OR "thematic analys*" OR "content analys*" OR "grounded theory" OR phenomenolog* OR "meta-ethnograph*" OR "meta-synth*" OR "meta narrative" OR "critical interpretive synthesis" OR "mega-ethnograph*") AND ("lived experience" OR "patient experience" OR "illness narrative*" OR stigma OR shame OR "self-blame" OR guilt OR "body image" OR "social isolation") AND (phenomenolog* OR "Affordance theory" OR "cultural affordances" OR ontology OR "systems thinking" OR "systems science" OR "complexity science" OR "social determinants of health" OR participatory)) |
| PsychINFO | ((TI "chronic pain" OR AB "chronic pain" OR TI "persistent pain" OR AB "persistent pain" OR TI "musculoskeletal pain" OR AB "musculoskeletal pain" OR neuralgia OR neuropath*) AND (TI "chronic illness" OR AB "chronic illness" OR TI "chronic disease" OR AB "chronic disease" OR chronic* OR "long-term" OR longterm OR persistent OR recurrent) AND (AB qualitative OR AB interview* OR AB "focus group*" OR AB ethnograph* OR AB "thematic analys*" OR AB "content analys*" OR AB "grounded theory" OR AB phenomenolog* OR AB "meta-ethnograph*" OR AB "meta-synth*" OR AB "critical interpretive synthesis") AND (AB "lived experience" OR AB "patient experience" OR AB "illness narrative*" OR stigma OR shame OR "self-blame" OR guilt OR "body image" OR "social isolation") AND (AB phenomenolog* OR AB "affordance theory" OR AB "cultural affordances" OR AB ontology OR AB "systems thinking" OR AB "systems science" OR AB "complexity science" OR AB "social determinants of health" OR AB participatory)) |
| ProQuest | ("chronic pain" OR "persistent pain" OR "long-term pain" OR "chronic pain syndrome" OR "musculoskeletal pain" OR "low back pain" OR "neck pain" OR neuralgia OR neuropath* OR "widespread pain" OR fibromyalgia OR headache OR "orofacial pain" OR "temporomandibular" OR "chronic pelvic pain" OR nocicept*) AND ("chronic disease" OR "chronic illness" OR chronic* OR "long-term illness" OR longterm OR persistent OR recurrent OR multimorbid* OR comorbid* OR "long term condition" OR "non-communicable disease" OR NCD) AND (qualitative OR "qualitative research" OR interview* OR "focus group*" OR ethnograph* OR "thematic analys*" OR "content analys*" OR "grounded theory" OR phenomenolog* OR "meta-ethnograph*" OR "meta-synth*" OR "meta narrative" OR "critical interpretive synthesis") AND ("lived experience" OR "patient experience" OR "illness narrative*" OR stigma OR shame OR "self-blame" OR guilt OR "body image" OR "social isolation") AND (phenomenolog* OR "affordance theory" OR "cultural affordances" OR ontology OR "systems thinking" OR "systems science" OR "complexity science" OR "social determinants of health" OR participatory) |
| AMED (Ovid) | (exp Pain/ OR chronic pain.tw. OR persistent pain.tw. OR "chronic pain.»tw. OR "musculoskeletal pain.»tw. OR "low back pain.»tw. OR neck pain.tw. OR neuralgia.tw. OR neuropath*.tw. OR fibromyalgia.tw. OR headache.tw. OR "orofacial pain.»tw. OR "temporomandibular.»tw.) AND (exp Chronic Disease/ OR chronic illness.tw. OR chronic disease.tw. OR chronic*.tw. OR "long term illness.»tw. OR longterm.tw. OR persistent.tw. OR recurrent.tw. OR multimorbid*.tw. OR comorbid*.tw.) AND (qualitative.tw. OR "qualitative research.»tw. OR interview*.tw. OR "focus group*.»tw. OR ethnograph*.tw. OR "thematic analys*.»tw. OR phenomenolog*.tw. OR "meta-ethnograph*.»tw. OR "meta-synth*.»tw.) |
| Cochrane Library | ("chronic pain" OR "persistent pain" OR "long-term pain" OR "chronic pain syndrome" OR "musculoskeletal pain" OR "low back pain" OR neuralgia OR neuropath* OR fibromyalgia OR headache OR "temporomandibular" OR "orofacial pain" OR "chronic pelvic pain" OR nocicept*) AND ("chronic disease" OR "chronic illness" OR chronic* OR "long-term illness" OR persistent OR recurrent OR "long term condition" OR multimorbid* OR comorbid*) AND (qualitative OR "qualitative research" OR interview* OR "focus group*" OR ethnograph* OR "thematic analys*" OR "grounded theory" OR phenomenolog* OR "meta-ethnograph*" OR "meta-synth*") |
| Google Scholar | "chronic pain" OR "persistent pain" OR "long-term pain" OR "musculoskeletal pain" OR "low back pain" OR neuralgia OR neuropath* OR fibromyalgia OR headache AND "chronic illness" OR "chronic disease" OR chronic* OR "long-term illness" OR persistent OR recurrent AND (qualitative OR "qualitative research" OR interview OR "focus group" OR ethnograph OR "thematic analysis" OR "meta-ethnograph" OR "meta-synth") AND ("lived experience" OR "patient experience" OR "illness narrative" OR stigma OR shame OR "self-blame" OR guilt OR "body image" OR "social isolation") |

***** A combination of Medical Subject Heading (MeSH) terms, controlled vocabulary, and free-text terms were used to capture relevant studies.

**Supplementary Table S4. Sequential Synthesis Details: Phases, Methods, and Key Outputs**

| Phase | Aim/Focus | Key methods / tools | Reviewers / roles | Key outputs / notes |
| --- | --- | --- | --- | --- |
| Phase 0 (Pre-synthesis) | Define scope, team, search, quality thresholds, and coding protocol | Databases: MEDLINE/ PubMed, Scopus, Web of Science, PsycINFO, ProQuest, AMED, Cochrane, Google Scholar; MeSH + free text; PRISMA; CASP/MMAT; GRADE-CERQual | SSA (lead), EAV, PD, RT (qual methods), PMR, BD (content experts) | Search period, inclusion/exclusion, PRISMA flow; selected studies for Phase 1; appraisal tables (see Supp.) |
| Phase 1 (Hermeneutic phenomenology) | Extract lived-experience themes; iterative coding and translation | Line-by-line coding; meta-ethnography (Noblit & Hare); hermeneutic circle; qualitative software; d3.js for node graph | Two independent coders per study: EAV, SSA; PD adjudicator; team consensus meetings | First- and second-order codes; 110 initial themes → 11 overarching domains; node graph and interpretive memos |
| Phase 2A (Heuristic ontology) | Map themes to ontological heuristic domains and weight relevance | Heuristic ontology framework; deductive re-coding; weighting rules (frequency + author judgment) | SSA, EAV (re-coding); group review (all authors) | 56 pain heuristics / 54 illness heuristics; chord diagram / radial network (node sizes reflect weighted relevance) |
| Phase 2B (Transdisciplinary pathways) | Align domains with transdisciplinary principles and identify implementation pathways | Literature mapping across 52 selected papers; thematic co-occurrence matrix; heat map (d3.js) | SSA, RT, PD, BD (pathway synthesis); external stakeholder input noted | Mapping matrix of domains × transdisciplinary principles; nine core principles identified; pathway documentation |
| Quality & confidence assessment | Assess methodological rigor and confidence in synthesized findings | CASP/MMAT; GRADE-CERQual evidence profiles; sensitivity analyses | Double coding, inter-rater agreement monitoring (target ≥85%); consensus resolution | CERQual profiles for main findings (high/moderate/low/very low); sensitivity analysis results in Supp. |
| Reliability, audit trail & reflexivity | Ensure reproducibility and document interpretive decisions | Version-controlled extraction sheets; audit logs; reflexivity memos; pilot coding (23 papers) | Coding team-maintained reflexivity logs; group reflexive sessions; analyst workshops (EAV, SSA + rotating reviewers) reviewed subtheme tables line-by-line, mapped subthemes to higher‑level heuristic codes, and recorded exact k‑counts (F) | Sensitivity testing and alternate coding procedures (R values: 3=core, 2=contextual, 1= lower relevance); resolved disagreements by consensus using exemplar quotes, and logged reconciliation notes. Sessions included sensitivity checks (ln(F+1), alternate R, leave‑one tests) and entries to the project audit trail; coding protocol, inter-rater stats, and reflexivity tables available in supplementary materials |
| Main outputs for manuscript | Concise, reproducible summary for readers | One main methods/results table in manuscript; figures: hermeneutic circle, radial network, co-occurrence matrix; brief codebook excerpt in main text | Lead authors prepare manuscript tables and reference supplements | Main table in-text; full codebook, study×code matrix, PRISMA, GRADE-CERQual evidence profiles, full figures, and audit trail, full protocol, codebook, PRISMA flowchart, and CERQual profiles available in Supplementary Materials |

**Supplementary Table S5. Hermeneutic Study Characteristics and Theme Development**

| Study Number | Study ID | Study Design | Population | Setting/ Country | Methodology | Theoretical Framework | Key Findings/ Initial Theme Development |
| --- | --- | --- | --- | --- | --- | --- | --- |
| 1 | Furnes & Dysvik,  2010 | Qualitative Evidence Review | Thematic Analysis of the Experiences of Patients with Chronic, Non-malignant Pain, Grief, and Loss | Studies from Multiple Countries | Open Qualitative Systematic Methodological Approach | Review of the literature | The study identified two main themes related to chronic non-malignant pain and associated grief: “relearning the world” and “adaptation.” It also identifies subthemes to those including “despair and hope,” “lack of understanding and insight,” and “bodily discomfort and reintegrated body” |
| 2 | Krohne & Ihlebaek, 2010 | Qualitative interview study | Experiences of 14 Norwegian adults with chronic whiplash associated disorder | Norway | Qualitative study using focus groups interviews, thematic analysis | Giorgi's phenomenological approach | The study identified three main themes related to the fluctuating, variable nature of the symptoms: (1) activity, (2) rest, (3) social withdrawal |
| 3 | Skjutar & Müllersdorf, 2010 | Qualitative interview study | Experiences of 10 Swedish adults with musculoskeletal chronic pain | Sweden | Semi-structured interviews, thematic content analysis | Occupational perspective on pain | The study identified three themes: (1) The need to handle the painful body and eager mind, (2) The need for transformation of self-image, and (3) The need to achieve affirmation through social engagement and enjoyment. Participants described experiencing inner conflict between rest and activity, the challenge of adapting to a new self-identity, and the importance of meaningful engagement for well-being. |
| 4 | Vallerand & Nowak, 2010 | Phenomenological study | Experiences of 22 patients with chronic pain and opioid medication treatment | USA | Semi-structured interviews, thematic analysis | Phenomenological study | The study established patient’s lived experiences before opioid treatment characterized by: (1) desperation, (2) inability to function. Life after treatment with opioids was characterized by: (1) living a secret life, (2) fear of losing the pain management regimen, and (3) gratefulness. The themes developed were: the (1) incapacitation of chronic pain (2) stigmatization, (3) secrecy to regain life and (4) improvement of functional capacity and ability to perform self-care. |
| 5 | Hansson et. al  2011 | Phenomenological Study | Experiences of 8 Patients with Chronic Pain on their encounters 6with the healthcare system | Sweden | Semi-structured interviews with thematic synthesis | Qualitative descriptive analysis | The study identified that negative approaches to chronic pain among healthcare providers contributed to increased feelings of negativity in chronic pain patients, and that patients often felt discredited and that their own personal experience of the situation was being called into question. |
| 6 | Skuladottir & Halldorsdottir, 2011 | Phenomenological study | Experiences of 5 women with chronic pain | Iceland | 12 basic steps of the research process of the Vancouver School | Vancouver School of Phenomenology | The study identified the following overriding themes: (1) the quest to find a diagnosis, (2) the quest for treatment, (3) the quest to learn to live with pain, (4) the quest for support and connection, (5) the quest for normalcy, (6) the quest for dignity, (7) avoid isolation and (8) loneliness, (9) the need for family support, (10) the need for financial support |
| 7 | Tollefson et. al  2011 | Phenomenological Study | Experiences of Rural Patients living with chronic pain | Australia | Open-ended interviews with thematic synthesis | Phenomenological analysis | The study identified several core themes including pain as silence, privacy as a way of protection, no place out here to get support, and dealing with healthcare professionals that do not understand. |
| 8 | Lavie-Ajayi et al., 2012 | Interpretative phenomenological analysis (IPA) | Experiences of 6 individuals with chronic pain | Israel | Semi-structured interviews, thematic analysis | Phenomenological analysis | The study identified the “double faced phenomenon”: a vivid and total experience on one hand, elusive and deceptive on the other. The nature of this phenomenon - together with the medical and public d delegitimization of chronic pain prompts patients to question their own experiences and to face what the authors call:” narratological distress.” |
| 9 | Nay & Fetherstonhaugh  2012 | Phenomenological Study | Experiences of four patients with chronic pain | Australia | Semi-structured interviews with thematic synthesis guided by the van Manen methodology | Phenomenological analysis | The study identified several major themes including that pain is what the patient says it is, how staff respond is integral to the pain process, pain involves shock, loss of control, unpleasant through to unbearable sensations. |
| 10 | West et al., 2012 | Qualitative interview study | Experiences of 10 individuals with chronic pain | Australia | Semi-structured interviews, thematic analysis | van Manen hermeneutic process | The study demonstrates the ability of individuals to express (1) resilience, (2) demonstrate positivity despite the challenges of illness, and (3) transcend the difficulties of chronic pain. |
| 11 | Snelgrove et. al,  2013 | Interpretative Phenomenological Analysis (IPA) | Experiences of 8 Patients with chronic low back pain | UK | Semi-structured interviews with thematic synthesis | Smith & Osborn (2003) guided data analysis | The study identified several core themes of the constant, intrusive, painful body; unchanging painful lives and fragmentation of self; changed and problematic relationship with the environment; loss of valued roles and changed social relationships; loss of social life; and managing the pain. |
| 12 | Taylor et. al,  2013 | Interpretative Phenomenological Analysis (IPA) | Experiences of 9 patients with a history of torture and persistent pain | UK | Semi-structured interviews, Post-Traumatic Stress Diagnostic Scale | Phenomenological analysis | The study identified four main themes, including “pain is the enemy,” “pain and intrusive memories are connected,” “changed identity,” and “resilience and resources” |
| 13 | Daker-White et al., 2014 | Meta-ethnography | Meta-ethnography of qualitative studies on the patients’ experience of  rheumatoid arthritis symptoms and socio-environmental factors | Studies from multiple countries | Meta-ethnography, synthesis of 52 studies use of Critical Appraisal Skills Program (CASP) tool, comparison to their previously published synthesis of 24 studies | Meta-ethnography, synthesis of qualitative evidence syntheses | Symptoms of rheumatoid arthritis were described as:  (1) an assault on self-identity, (2) biographical disruption, (3) role incompetence, (4) dread of dependency on others and a need for new ways to live. |
| 14 | Dysvik et. al,  2014 | Qualitative Interview Study | Experiences of 34 patients with chronic pain undergoing pain management | Norway | Semi-structured interviews | Descriptive qualitative analysis | Written qualitative reports indicated the importance of group processes, increased self-understanding, and other aspects meaningful to the participants that overall improved and alleviated their suffering. |
| 15 | Furnes et. al,  2014 | Phenomenological Analysis | Experiences of 34 participants with chronic non-malignant pain who participated in group-based pain management programs. | UK | Open interviews, thematic synthesis | Phenomenological Analysis | The study identified two main subthemes: “the significance of active involvement in gaining new insight” and “the significance of community and group support” |
| 16 | Juuso et. al,  2014 | Phenomenological analysis | Experiences of 9 women with a history of fibromyalgia | Sweden | Semi-structured interviews with thematic synthesis | Phenomenological hermeneutic analysis | The study identified two major themes from the women’s experiences: “being seen as a malingerer” and “being acknowledged” |
| 17 | Lima et. al,  2014 | Qualitative Evidence Review | Review of the evidence for the current model of understanding of chronic pain and inclusion of phenomenological perspectives on that understanding | Studies from multiple countries | Descriptive qualitative evidence reviews | Descriptive synthesis of the perspective of phenomenological and nonphenomenological definitions of chronic pain | The study identified and discussed the differences between the classical approach to understanding chronic pain, and the phenomenological approach to understanding chronic pain and argues for the benefits of the phenomenological model, stating that it could improve adherence and treatment efficacy. |
| 18 | Mahon et al.,  2014 | Qualitative Interview Study | Experiences of 19 patients with chronic illness | Ireland | Semi-structured interviews with thematic synthesis | Followed the qualitative descriptive design described by Sandelowski (2000) | The study identified several common themes among its participants including a sense of loss, feeling of anger and frustration, uncertainty and stress, and adapting to new life. |
| 19 | Mathias et. al,  2014 | Interpretative Phenomenological Analysis (IPA) | Experiences of 6 participants with chronic pain going through acceptance therapy | UK | Semi-structured interviews, thematic synthesis | Phenomenological Analysis | Five major themes emerged: “I’m not alone,” “others understand my pain,” “freedom from pain taking over,” “a new self, one with pain,” and “facilitated change with exercise is possible.” |
| 20 | Merolli et al., 2014 | Qualitative interview study | 218 individuals with chronic pain using social media for self-management | Global (participants recruited online) | Online survey with open-text responses, thematic content analysis | Theory of Affordances | Identified five therapeutic affordances of social media: (1) Exploration (information seeking), (2) Connection (mitigating isolation), (3) Narration (shared experiences), (4) Adaptation (adjusting self-management), (5) Self-Presentation (controlling identity). Social media facilitates support, information exchange, and emotional validation, but also presents concerns about misinformation and privacy. |
| 21 | Nilsen & Anderssen,  2014 | Phenomenological Study | Experiences of 20 patients with chronic, non-malignant pain | Norway | Open-ended interviews | Descriptive Phenomenological analysis | The study found that participants experienced loneliness, fear of stigmatization, and despair because of their condition and were motivated to come back to a normal daily life and especially to get back to paid work. |
| 22 | Ojala, et. al  2014 | Phenomenological Analysis | Experiences of 34 participants with chronic non-malignant pain, most of which had degenerative spinal pain | Finland | Semi-structured interviews with thematic synthesis | Giorgi’s four-phase phenomenological method | The study identified five major subthemes: “the dominance of chronic pain,” “pain is the master,” “life is not worth living,” “contextual pain,” and “waiting and hoping” |
| 23 | Brooks et. al, 2015 | Interpretative Phenomenological analysis (IPA) | Experiences of 9 patients with chronic non-cancer pain that were managing their pain with prescription opioids |  | Semi-structured interviews with thematic synthesis and subthemes | Phenomenological analysis | The study identified six major themes relating to the use of opioids in the management of chronic pain: “pharmacological side effects,” “feeling stigmatized,” “guilt,” “fear,” “ambivalence,” and “self-protection/acceptance” |
| 24 | De Villiers et. al,  2015 | Phenomenological Study | Experiences of 9 underprivileged participants with chronic pain | South Africa | Semi-structured interviews, Tesch coding process, thematic synthesis | Phenomenological analysis | The study identified four themes among their participants: “pain is a multidimensional experience,” “influence of pain on physical activities,” “the psychosocial influence of pain,” and “influence of pain on spirituality” |
| 25 | Hearn et. al,  2015 | Interpretative Phenomenological Analysis (IPA) | Experiences of 8 participants with chronic neuropathic pain with a history of spinal cord injury | UK | Semi-structured interviews | Phenomenological Analysis | The study identified three supra-ordinate themes: “a perceived gap between treatments received and participants' views of what they wanted and needed”; “a fight for life control and acceptance”; and “feeling understood by others with SCI, but isolated from the non-understanding able-bodied.” |
| 26 | Kirkham et. al,  2015 | Interpretative Phenomenological Analysis (IPA) | Experiences of seven women with chronic pain | UK | Patients painted what they viewed their pain as and were subsequently given semi-structured interviews about their paintings and what they meant to them, thematic synthesis | Phenomenological Analysis | The study described the paintings themselves as strong, vivid, and abstract, often objectifying the pain as punitive and sinister. Some paintings also depicted the transition from the self before pain to the self with pain. |
| 27 | MacNeela et al., 2015 | Meta-ethnography | Patient experiences with chronic low back pain | Global (various countries and cultural settings) | Meta-ethnographic synthesis of the findings of 38 qualitative studies, multiple themes were synthesized | Meta-ethnography based on Noblit &  Hare | Chronic low back pain creates significant constraints on self and social interactions. Chronic pain creates: (1) an oppressive intrusion on self-identity, (2) creates family strains, (3) loss of income and job opportunities, (4) creates social withdrawal and creates (5) dissatisfaction with healthcare and clinicians. Patients can also engage in (1) self-management activities and learn to (2) live with the pain. |
| 28 | Ojala et al., 2015  (This is a separate study done with the same population of participants as Ojala et. al 2014 and the study below by the same) | Phenomenological Study | Experiences of 34 patients with chronic pain, most of whom had degenerative spinal pain | Finland | Semi-structured interviews, open coding, thematic synthesis with themes and subthemes identified | Giorgi’s phenomenological method | This study focused on the theme that “pain affects the whole person” identified by the overarching study and further delves in to the subthemes of the same which include “distress,” “loneliness,” “lost identity,” and “low quality of life” |
| 29 | Ojala et al., 2015 | Phenomenological study | Experiences of 34 patients with chronic pain | Finland | Semi-structured interviews, open coding, thematic analysis, themes and subthemes were identified | Giorgi’s phenomenological method | The study identified themes around rejection by the health care and social security systems, contributing to additional unnecessary mental health problems for the participants. Subthemes included: (1) being disbelieved, (2) denying pain, (3) underrating symptoms, (4) pain is in your head, (4) second-class citizen, (5) lazy pain patient, (6) false beliefs demand passivity. |
| 30 | Ojala et. al, 2015 | Phenomenological Study | Experiences of 34 patients with chronic pain, most of which had degenerative spinal pain | Finland | Semi-structured interviews, open coding, thematic synthesis | Giorgi’s phenomenological method | The study focuses on the patients’ own views of what their pain means to them, identifying themes of “state of reflection,” “reconsidering values,” “acceptance of pain,” “support network,” “altered self,” “joys in life,” and “pain dissociation” |
| 31 | Röing & Sanner 2015 | Meta-ethnograph | Patients’ lived experiences with chronic illness | Scandinavian Countries | Meta-ethnographic synthesis of phenomenographic studies on chronic illness | Synthesis and  phenomenographic interpretation of 12 selected articles | Four categories describing patients’ experiences of  chronic illness: (1) a different lived body,  (2) a struggle with threat to identity and self-esteem,  a (3) diminished lifeworld, and (4) a challenging reality. |
| 32 | Svensson et. al,  2015 | Phenomenological Study | Experiences of ten women with chronic pain secondary to vertebral compression fractures | Sweden | Open interviews, open coding, thematic synthesis | Phenomenological hermeneutic analysis | The study identified themes of “struggling to understand a deceiving body,” “breakthrough pain fueling fear,” “fearing a trajectory into isolation,” “concerns of dependency,” and “fearing an uncertain future” |
| 33 | Coleman et. al,  2016 | Interpretative Phenomenological Analysis (IPA) | Experiences of 7 patients with a history of sickle cell disease and chronic pain | UK | Semi-structured interviews with open coding and thematic synthesis | Phenomenological analysis | The study identified that the patients felt that their pain experiences were unimaginable, agonizing, continuous, inescapable, and limitless, all while being almost impossible to describe, with participants often resorting to analogies and personification. They felt obliged to accept their illness as it would nearly be cured, but were nevertheless able to appreciate life and recognize positive life lessons. |
| 34 | Dueñas et al. 2016 | Qualitative evidence review | Effects of chronic pain and chronic illness such as Fibromyalgia on individuals, their families and social environment | Studies from multiple countries | STROBE (Strengthening the Reporting of Observational Studies in Epidemiology) checklist, without any synthesis of the data: 78 studies, themes were not identified methodologically | Review of literature without theoretical foundation | Identification of associated problems, such as depression and anxiety in caregivers who support patients with chronic pain and chronic illness |
| 35 | Rambod et. al,  2016 | Phenomenological Study | Effects of chronic pain in 14 hemophilia patients | Iran | Semi-structured interviews with thematic synthesis and van Manen six-step analysis | Phenomenological analysis | The study identified three main themes: “alteration in physical health,” “engagement in psychological problems,” and “impairment in social relationships” |
| 36 | Angel et. al,  2017 | Phenomenological Study | Experiences of 25 individuals with chronic low back pain after biopsychosocial counseling intervention | Scandinavia | Semi-structured interviews, Ricoeur’s three-stem interpretation | Phenomenological analysis | The study identified several narratives in getting on with life without pain, including recovering and returning to prior lifestyle, minimizing pain with strict regimes, and developing strategies for when pain recurs. For those with continual pain they identified narratives of finding a way to a functioning everyday life with pain and being stuck and finding no way out. |
| 37 | Hassani et al., 2017 | Phenomenological study | Experiences of 22 individuals with chronic pain | Iran | Colaizzi nine steps for directing a descriptive  phenomenological study,  themes and subthemes were developed | Descriptive phenomenology | The study identified the following themes for resilience: (1) the art of overcoming suffering, (2) purpose and (3) spirituality, (4) support and connection: |
| 38 | Morton et al., 2017 | Meta-ethnography | Patients with chronic conditions and chronic pain | Studies from multiple countries | Studies were appraised with the multi-dimensional National Institute for Health and  Clinical Excellence (NICE) quality appraisal checklist for qualitative  studies | Meta-ethnography based on Noblit &  Hare | Patients using self-management digital interventions often feel well cared for and take a more active role. Tele-monitoring physiological data can encourage complex behavioral changes. |
| 39 | Palacios-Ceña et. al,  2017 | Phenomenological study | Experiences of 20 women with a history of chronic migraines that presented to a headache clinic | Spain | Semi-structured interviews with open coding and thematic synthesis | Phenomenological analysis | The study identified five main themes regarding the significance of the patients’ suffering: shame of suffering from an invisible condition, treatment need/skepticism/fear, looking for physician support and sincerity/fighting misconceptions, limiting the impact on daily life through self-control, and understanding/disbelief from family and work |
| 40 | Schütze et. al,  2017 | Qualitative Interview Study | Experiences of 15 adults with chronic low back pain that scored highly on the pain catastrophizing scale | Australia | Semi structured interviews with independent analysis | Analysis of metacognition based on the interview data | The study identified several characteristic metacognitions in patients with pain catastrophizing including thinking helps me to cope, rumination is uncontrollable, and worry is pointless. Often, these were held at the same time, creating significant internal conflict. |
| 41 | Toye et al., 2017 | Mega-ethnography | Lived experiences of patients with chronic pain | Studies from multiple countries | Authors did not formally appraise the quality of included 11 qualitative studies | Meta-ethnography, synthesis of qualitative evidence syntheses, the authors coin mega-ethnography | Development of seven themes: (1) my life is impoverished and confined; (2) struggling against my body to be me; (3) the quest for the diagnostic ‘holy grail’; (4) lost personal credibility; (5) trying to keep up appearances; (6) need to be treated with dignity; and (7) deciding to end the quest for the grail is not easy. |
| 42 | Twiddy et. al,  2017 | Qualitative Interview Study | Experiences of 18 adults aged 18-30 in focus groups at a pain clinic in the UK | UK | Semi-structured interviews | Descriptive phenomenology | This study identified four main themes from the interviews: thwarted opportunities, peer separation, illness validity in the context of age, and dependency/parental enmeshment. |
| 43 | Al Kalaldeh et al., 2018 | Qualitative evidence review | Patients with chronic illnesses also experiencing suffering. | Studies from multiple countries | Studies were appraised for their quality and validation using the Critical Appraisal Skill Program (CASP) | Review of 26 studies focusing on lived experiences of suffering. The study draws on philosophical perspectives from Husserl | Understanding of how chronic illness affects self-perception, social interactions, and overall well-being, thematic synthesis is not performed |
| 44 | Cooper et al.,  2018 | Qualitative interview study | Experiences of 18 adults BMI >=25-46 with chronic musculoskeletal pain | UK | Semi-structured interviews with thematic synthesis and open coding | Phenomenological analysis | Three superordinate themes: pain as a motivator and barrier to weight loss, fear of weight causing more damage, and activity is positive. This study highlights the dichotomy of pain being both a motivator and a barrier to healthy weight loss. |
| 45 | Gutke et al.,  2018 | Phenomenological Study | Experiences of nine women with chronic pelvic girdle pain after childbirth | Sweden | Open-ended interviews and thematic synthesis | The Empirical Phenomenological Psychological Method | The study identified three constituent themes as central to the experience: importance of the body for identity, the understanding of pain, and the stages of change. |
| 46 | Hearn et. al,  2018 | Interpretative Phenomenological Analysis (IPA) | Experiences of 8 patients with history of spinal cord injury and chronic neuropathic pain | UK | Semi-structured interviews, open coding, and thematic synthesis | Phenomenological analysis | The study identified three supraordinate themes mediating pain and adherence: the dichotomy of safety perceptions, adherence despite adversity, and fighting the future. |
| 47 | Leder, 2018 | Qualitative Evidence Review | Review of multiple studies documenting the experiences of patients with chronic pain/illness and long-term incarceration | US | Studies were appraised independently for quality | Qualitative descriptive analysis | The study identified some experiential commonalities of patients including constriction of lived space and range of possible action, disruption of lived time such that one is trapped in an aversive “now,” isolation and rapid removal of meaningful social contact, and disempowerment/depersonalization. |
| 48 | Lee et al, 2018 | Interpretative Phenomenological Analysis (IPA) | Experiences of 8 patients diagnosed with COPD experiencing moderate to severe COPD pain | Canada | Semi-structured interviews with thematic synthesis | Phenomenological analysis | This study identified five core themes: the pain complicates the clinical profile of COPD, uncertainty of the pain experience: frustrations related to healthcare professionals’ explanation of the pain and need to legitimize, language and the behavior of pain, psychological reactions towards pain including depression and fear-avoidance behavior and altered identity perception and guilt. |
| 49 | Hazaveh and Hovey,  2018 | Qualitative Interview Study | Experiences of 6 patients living with chronic orofacial pain who were undergoing treatment at a pain clinic | Montreal | Open interviews with qualitative analysis | Descriptive phenomenological analysis as defined by van Manen and Laverty (2003) | The study identified consistency in the experiences of these patients including dissatisfaction with their journey through the healthcare system and the loss of self-identity, ability to enjoy eating or engaging in social activities due to their chronic pain. |
| 50 | McGeechan et al.,  2018 | Interpretative Phenomenological Analysis | Experiences of 6 participants attending oncology follow-up for colonic cancer | England | Semi-structured interviews with thematic synthesis | Phenomenological Analysis | The study identified two superordinate themes including physical and psychological consequences of cancer and adapting to life after treatment. |
| 51 | Mengshoel et al., 2018 | Meta-ethnography | Patient’s experiences with the process and consequences of  receiving the diagnosis of Fibromyalgia | Global (various countries and cultural settings) | Synthesis of 27 studies using criteria to appraise the quality of primary studies by the Joanna Briggs Institute instrument (JBI-QUARI). | Meta-ethnography based on Noblit &  Hare | Two broad concepts relating to the role of the Fibromyalgia diagnosis in  validating and making sense of bodily experiences, and its role in legitimating sickness personally and socially |
| 52 | Stensland & Sanders,  2018 | Phenomenological Study | Experiences of 21 clinic patients with chronic low back pain | US | Semi-structured interviews, line-by-line thematic coding | Phenomenological analysis | The sub-study focused on one main theme of “it changed my whole life,” with subthemes including pain damages the sense of self, trapped in a body that doesn’t work anymore; me, my partner, and my pain; pain complicates family relationships, painfully employed, and socially and recreationally repressed. |
| 53 | Stensland & Sanders, 2018 | Phenomenological Study | Experiences of 21 patients with chronic lower back pain | US | Semi-structured interviews with thematic synthesis | Phenomenological analysis | This sub-study focused on the theme of living a life full of pain, with subthemes of corporeality, temporality, relationality, and spatiality |
| 54 | Stensland & Sanders, 2018 | Phenomenological Study | Experiences of 21 patients with chronic lower back pain | US | Semi-structured interviews with thematic synthesis | Phenomenological analysis | The sub-study focused on the theme “not so golden after all” relating each patient’s pain and their experience with aging, including subthemes such as falling apart, pain stigmatizes aging, hurting slowly but aging quickly, pain threatens independence, and the reality of unrealized futures. |
| 55 | Bath et al.,  2019 | Phenomenological Study | Experiences of 12 farmers living with chronic low back pain | Canada | Semi-structured interviews with thematic synthesis | Phenomenological analysis | The study identified two overarching themes related: seasonality and isolation with subthemes including pushing through, doing less, barriers to healthcare, and self-management. |
| 56 | Cheng et al, 2019 | Meta-ethnography | Patients experiences with multiple chronic conditions | Studies from multiple countries and various cultural settings | A seven-step analytic method was used after a  quality evaluation based on Joanna Briggs Institute Qualitative Assessment and Review  Instrument (JBI-QUARI). | Meta-ethnography based on Noblit &  Hare | Experiences of coping with multiple chronic conditions, including: (1) appraisals, (2) strategies to maintain a normal life, (3) strategies to  keep the spirits up, and (4) coping in the social context. |
| 57 | Hughes et al.,  2019 | Interpretative Phenomenological Analysis (IPA) | The experiences of wheelchair-using individuals with paraplegia and chronic pain relating to quality of life | UK | Participants photographed things that they thought described their daily QoL and were then interviewed over telephone regarding those pictures | Phenomenological analysis | The study identified three superordinate themes: experiencing quality of life through the perception of self and identity, interpersonal relationships as facilitators and barriers to quality of life, and pain and experience management. |
| 58 | Rodríguez et al., 2019 | Qualitative interview study | 10 adults with chronic pain due to spine deformity | Chile | Semi-structured interviews, open coding, thematic analysis | Grounded Theory, Cultural Perspective on Pain | Chronic pain affects daily activities, requiring adaptations in home environments and physical positions. Social and emotional well-being is impacted, but the strong family and community ties in Chile offer support. Public services need improvements for accessibility. |
| 59 | Schultz et al.,  2019 | Interpretative Phenomenological Analysis (IPA) | Experiences of 10 patients with chronic widespread pain | Denmark | Semi-structured interviews with open coding and thematic synthesis | Phenomenological analysis | The study identified four superordinate themes: meeting different attitudes, fragmentation of treatment, the importance of time, and feeling trapped. |
| 60 | Toye et al., 2019 | Mega-ethnography | patients’ experience of living with rheumatoid arthritis symptoms and socio-environmental factors | Studies from multiple countries | mega-ethnography utilized to use conceptual findings from qualitative evidence syntheses of 9 studies, development of 10 themes | Mega-ethnography, | Ten conceptual categories: (1) rheumatoid arthritis is in control of my body, (2) rheumatoid arthritis alters reciprocity, (3) rheumatoid arthritis is an emotional challenge, (4) rheumatoid arthritis disrupts my present and future self, (5) the challenge of balancing personal and work life, (6) I am trying to make sense of what is happening, (7) rheumatoid arthritis is variable and unpredictable, (8) rheumatoid arthritis is invisible, (9) I need a positive experience of healthcare, and (10) I need to reframe the situation. |
| 61 | Mustafa et al.,  2020 | Phenomenological Study | Experiences of 13 Indian Canadian women living with chronic pain | Canada | Semi-structured interviews, van Manen thematic analysis | Phenomenological analysis | The study identified four themes: the body in pain, pain in the context of lived and felt space, pain and relationships, and pain and time. The women in the study attributed this to a “dual-gender role” adopted after immigration where they had to balance traditional household tasks and employment outside the home, which exacerbated their pain. |
| 62 | Roenne et al.,  2020 | Phenomenological Study | Experiences of 10 patients with chronic non-malignant pain from three different multidisciplinary pain centers. | Denmark | Interpretation of the documentation of those pain centers as guided by Paul Ricouer’s philosophy | Phenomenological analysis | This study focused predominantly on the roles of relatives in the patient’s experience of their chronic pain, finding themes of relative involvement being arbitrary, loss of relationships and risk of social isolation, and a profound need for being understood. |
| 63 | Skúladóttir et al.,  2020 | Phenomenological Study | Experiences of eleven participants with chronic pain | Vancouver | The Vancouver School of Doing Phenomenology | Phenomenological Analysis | This study identified an overarching theme of “the journey of breaking the vicious circle of chronic pain” and describes their journey from a position of being in that cycle while simultaneously trying to hide the pain to reconstructing their daily lives after the pain rehabilitation program. |
| 64 | Yildirim Duman  2020 | Phenomenological Study | Experiences of 10 patients with chronic disease who attended two family healthcare centers | Turkey | Semi-structured interviews and thematic synthesis | Phenomenological analysis | This study identified three main themes of social support, disease management, and self-awareness/empowerment |
| 65 | Andersen et al., 2021 | Interpretative phenomenological analysis (IPA) | 23 adult patients with chronic illness and chronic pain | Denmark | Semi-structured interviews, thematic analysis | Analysis of Kierkegaardian concepts,  double hermeneutical-Phenomenological analysis, | Experiences of illness are perceived as an unwelcome and significant alteration to individuals' lives. Esthetic, ethical, and religious dimensions, along with adaptive strategies, have been developed in response. |
| 66 | Fracso et al.,  2021 | Phenomenological study | Experiences of 20 patients with chronic diseases before and after The Chronic Disease Self-Management Program. | France | Semi-structured interviews with thematic synthesis | Phenomenological analysis | This study identified areas of thematic improvement for the patients from the program including in terms of self-empowerment, belonging in a community of peers, and becoming involved in their health education. |
| 67 | Honoré Grauslund et al.,  2021 | Phenomenological Study | Experiences of 11 Turkish participants with chronic low back pain | Denmark | Semi-structured interviews and Giorgi’s phenomenological analysis | Phenomenological analysis | This study identified three superordinate themes from their analysis: overwhelming and prevailing pain, being distressed in community life, and looking for a way to escape pain. |
| 68 | Keen et al., 2021 | Qualitative interview study | 12 UK adults with chronic pain | United Kingdom | Semi-structured interviews, Grid Elaboration Method (GEM), Pain Neurophysiology Education (PNE) article review | Social Representations Theory (SRT), Pain conceptualization models | Chronic pain is understood through inconsistent experiential models; PNE was intellectually engaging, but perceived as practically inadequate. Participants found PNE validating, but questioned its impact on behavior change. |
| 69 | Niño de Guzmán Quispe et al. 2021 | Qualitative evidence review | Patients with various chronic illnesses | Studies from multiple countries | Thematic synthesis of self-management themes and sub-themes in chronic illness of 148 studies | Thematic synthesis, mixed methods | Twelve main themes were identified: Eight described the process of illness self-management: disease progression, self-management behaviors, social support, interaction with healthcare professionals, access to healthcare, costs for patients, culturally defined roles and perceptions, and health knowledge |
| 70 | Stensland  2021 | Phenomenological Study | Experiences of 21 patients with treatment-resistant chronic low back pain | US | Semi-structured interviews and van Manen guided analysis | Phenomenological analysis | The main theme was managing the incurable with four subthemes including a quest for relief, spinal procedures offering only temporary relief yet ongoing disappointment, the apprehension of medication, and making things work for me. |
| 71 | Stensland  2021  (independent of previously listed) | Phenomenological Study | Experiences of 21 patients with treatment-resistant chronic low back pain | US | Semi-structured interviews and van Manen guided analysis | Phenomenological analysis | The findings focused on the overarching theme of helplessness and perseverance, with subthemes including feeling helpless because nothing works, feeling down and depressed, distantly wishing for an end, accepting the reality of my pain, and the pain stays, I keep going |
| 72 | Toye et al., 2021 | Meta-ethnography | Healing and recovering from chronic pain | Studies from multiple countries | Utilization of eMERGe meta-ethnography reporting guidance, synthesis of 195 studies, development of 10 themes | Meta-ethnography | The study showed that health interventions for chronic pain should focus on: 1) validating pain through clear and rational explanations; 2) validating patients by listening to and considering their experiences; 3) encouraging patients to connect with a sense of self, practice self-kindness, and explore future possibilities; and 4) facilitating safe reconnection with the social world. |
| 73 | Chala et al.,  2022 | Interpretive Phenomenological Analysis (IPA) | Experiences of 15 participants with chronic low back pain | Ethiopia | Semi-structured interviews with open coding and thematic analysis | Phenomenological analysis | The main themes included chronic low back pain affects life on a day-to-day basis, the invisibility of pain results in misunderstanding, misjudgment, and loneliness, the cause of pain is a mystery, the search for a cure is a quest, and each person has their own ways of managing, coping, and living with the pain. |
| 74 | Kahere et al,  2022 | Phenomenological study | Experiences of 15 participants on the barriers of accessing low back pain health services | South Africa | Semi-structured interviews with barrier-focused analysis | Phenomenological analysis | Barriers identified included: travel, long waiting periods, shortage of personnel, poor infrastructural development, inadequate healthcare personnel, communication barrier, social influence, beliefs around cause and effect, misdiagnosis, and inappropriate and/or ineffective treatment approaches. |
| 75 | Altun et al.,  2023 | Phenomenological Study | Experiences of 10 Assyrian women of a refugee background living with chronic pain | Australia | Semi-structured interviews and review of field notes with thematic synthesis | Phenomenological analysis | This study identified five major themes: the story of pain, the experience of help seeking in Australia and their home country, factors shaping the ability to access appropriate care, support seeking systems, and influence of culture and gender roles. |
| 76 | Chimezie, 2023 | Qualitative evidence review | Individuals at risk of or living with chronic diseases | Global (focus on Africa and low-income countries) | Narrative synthesis of existing research and policy analysis | Health Literacy Framework | This study highlights the role of health awareness in chronic disease prevention and access to care. Emphasizes the impact of health literacy, socioeconomic status, cultural beliefs, and healthcare system weaknesses on chronic disease management. Recommends improving community education, access to care, and preventive health strategies. |
| 77 | Joyce et al., 2023 | Qualitative interview study | 12 underserved adults with chronic low back pain | United States | Semi-structured interviews, thematic analysis | Bandura’s Self-Efficacy Theory, identification of key themes | This study identified three major themes: 1) Empowerment through education and exercise, 2) Interconnectedness with providers and other patients, and 3) Improvements in pain, body mechanics, and mood. Some participants experienced limited improvement due to therapist disengagement. |
| 78 | Kjærulff et al., 2023 | Qualitative evidence review | Individuals with chronic conditions, particularly diabetes, cardiovascular diseases, obesity, hypertension, and dyslipidemia | Studies from multiple countries | Narrative synthesis of 17 studies, review and synthesis of qualitative, quantitative, and mixed methods approaches, Mixed Methods Appraisal Tool (MMAT) | Narrative Synthesis | This study assessed peer-to-peer interactions on social media influence relationships with healthcare professionals (HCPs) in three keyways: (1) impacting communication with HCPs, (2) shaping discussions about medical advice, and (3) influencing perceptions of HCPs. Findings highlight that social media provides empowerment and support but also introduces concerns about misinformation and conflicting views with HCPs. |
| 79 | Peppard et al.,  2023 | Phenomenological Study | Experiences of 13 active duty, retired, and veteran women dealing with chronic pain | US | Semi-structured interviews with van Manen’s approach | Phenomenological analysis | This study identified eight themes: (1) chronic pain is a frustrating, persistent, daily, and an hourly struggle; (2) resilience in living with chronic pain is the new normal; (3) mission first and the impact of invisible pain; (4) self-care management and internal locus of control with nonpharmacological therapies; (5) pain accepted and managed to improve quality of life; (6) coronavirus disease 2019 (COVID-19) diminished social interactions; (7) pain of sexual trauma is not reported; and (8) disparities in health care due to self-perception of provider bias as pain is not understood. |
| 80 | Sun et al. 2023 | Meta-ethnography | Individuals with chronic illnesses and their caregivers regarding hospital-to-home transitions | Studies from multiple countries | Thematic synthesis of seventeen qualitative studies; (CASP) qualitative research appraisal tool. | A narrative synthesis was conducted informed by Meleis's Theory of Transition. | This study identified three barriers to transitional care: (1) communication barriers, (2) location and rural areas, (3) psychological stress; and facilitators: (1) family caregiver support and (2) nurse-provided patient-centered care and care coordination |
| 81 | van Rysewyk et al., 2023 | Meta-ethnography | Individuals with chronic non-cancer pain (CNCP) | United Kingdom | thematic qualitative analysis, lived experience, pain perception, and patient-centered perspectives | Mega-ethnography, | This study identified eight key themes related to the experience of chronic pain: emotional burden, identity changes, social implications, and struggles with pain management. A survey validated these themes among UK pain community members. |
| 82 | Albán-Reyes et al.,  2024 | Qualitative interview study | Experiences of 17 professional ballet dancers from Colombia with chronic pain and their coping strategies | Colombia | Semi-structured interviews | Interview analysis | The dancers reported frequent injuries resulting in chronic sprains, back pain, disc herniation, and other pains often attributed to poor strengthening and imposition of high physical loads. To cope with the pain the dancers would often seek out strategies for self-affirmation and motivation to continue their profession. |
| 83 | Bruger et al.,  2024 | Interpretative Phenomenological Analysis (IPA) | Experiences of 8 working age UK stroke survivors with chronic post-stroke pain | UK | Semi-structured interviews with thematic synthesis | Phenomenological Analysis | This study identified three group experiential themes: the solitude of the pain experience, unsatisfactory healthcare and the need for self-care, and development of pain acceptance. |
| 84 | Bullo et al.,  2024 | Interpretive Phenomenological Analysis (IPA) | Experiences of 10 women in two focus groups experiencing chronic pain secondary to endometriosis | UK | Semi-structured interviews with conceptual metaphor and metonymy analysis to examine visual representations of endometriosis pain | Phenomenological Analysis | This study identified Pain as physical violence as the overarching theme, with subthemes including color as emotional representation, texture as sensory qualities, and materials as sensation. |
| 85 | Gaber et al., 2024 | Meta-ethnography | Older adults living in long-  term care facilities | Studies from multiple countries | Studies were appraised for their quality and validation using the Critical Appraisal Skill Program (CASP), followed eMERGe guidelines | Meta-ethnography based on Noblit &  Hare | The conceptualization of participatory research approaches in long-term care facilities for older adults provides practical insights into: (1) empowerment, (2) growth, (3) cultural change, and (4) social change, which have emerged from these methodologies. The epistemology of participatory research is aimed at enhancing everyday life rather than just studying it. |
| 86 | Lilja et al.,  2024 | Phenomenological Study | Experiences of ten individuals with chronic musculoskeletal pain | Sweden | Semi-structured interviews guide derived from Brinkmann and Kvale focusing on support in the management of chronic pain | Phenomenological analysis | The predominant two themes identified by this study were: balancing being the most valuable player and accepting the support required to be that capable person. The study stressed the importance of both maintaining independence and personal care as well as being able to defer some decision-making and independence to healthcare systems when appropriate. |
| 87 | Lo Monaco et al., 2024 | Phenomenological study | Experiences of 10 female patients with Fibromyalgia | Italy | In-depth, qualitative interviews, thematic and interpretative phenomenological analysis. Themes and subthemes were created; Colaizzi's seven‐step method for analysis utilized | Phenomenological inquiry,  philosophical foundations of Husserl and Heidegger. | The study established 5 themes: (1) trauma preceding diagnosis, (2) pervasive feelings of misunderstanding, (3) struggle to maintain strength, (4) challenges in fulfilling maternal roles, (5) persistent sexual discomfort. |
| 88 | Lundin et al., 2024 | Phenomenological study | Experiences of 14 individuals with chronic and their difficulties returning to work | Sweden | Semi-structured interviews, thematic analysis | Phenomenological hermeneutics, themes and subthemes were created | This study developed 6 themes: (1) feeling safe, (2) feeling valuable, (3) being trusted to take command, (4) access to dependable care, (5) flexibility in planning, (6) support for transitioning towards a sustainable future |
| 89 | Macgregor et al., 2024 | Meta-ethnography | Patients’ lived experiences of acceptance of  chronic pain | Studies from multiple countries | Utilization of eMERGe meta-ethnography reporting guidance | Meta-ethnography based on Noblit &  Hare | This study showed that the conceptualization of accepting life with chronic pain can be viewed as an ecosystem, reflecting the fluid and continuous journey that is interdependent with our socio-cultural environment. |
| 90 | Matias-Soto et al., 2024 | Interpretative phenomenological analysis (IPA) | Experiences of 22 patients with chronic low back pain | Spain | Cross-sectional qualitative study based on individual semi-structured interviews, interpretative phenomenology approach through a depth-reflective inquiry. Themes and subthemes were created. Utilization of the Consolidated Criteria for Reporting Qualitative Research (COREQ) | Phenomenological analysis | A variety of emotional factors were defined: (1) fears, (2) frustrations, and (3) worries were the three most expressed. Cognitive factors were identified related to: (1) pain predictability, (2) pain perception, and (3) pain interference/ disability. The theme behaviors were composed of the following subthemes: (1) strategies for managing symptoms, (2) social factors, and (3) strategies for coping with daily tasks. Additionally, cognitive factors related to the (1)"Diagnosis,» (2)"Health system attention,» and (3)"Medical prescriptions" were defined. |
| 91 | Nikoloudi et al., 2024 | Interpretative phenomenological analysis (IPA) | Experiences of 6 individuals who are among the last survivors of the chronic effects of leprosy | Greece | In-depth, qualitative interviews, thematic and interpretative phenomenological analysis. Themes and subthemes were created. | Phenomenological analysis | Navigation of illness through the cultural meaning system surrounding leprosy was illustrated by the themes. The societal meanings and experiences of severe stigmatization, leading to social exclusion and the loss of previous lives and self-identity. The biographical disruption caused by leprosy led to the establishment of new routines and a devalued identity, thus profoundly affecting social identities and overall sense of self. |
| 92 | Okolo et al., 2024 | Meta-ethnography | Individuals from various cultural backgrounds experiencing pain | Global (various countries and cultural settings) | Thematic synthesis of anthropological literature | Cultural Construction of Pain, Sociocultural Model of Pain | This study highlighted how pain perception, expression, and management vary widely across cultures. Some cultures emphasize stoicism, while others encourage open expression. Pain management strategies include: (1) traditional healing, (2) biomedical approaches, and (3) spiritual practices. This study highlights the need for culturally sensitive healthcare approaches to pain management. |
| 93 | Sanabria-Mazo et al.,  2024 | Qualitative Interview Study | Qualitative interview study nestled within a larger RCT on 55 patients with chronic low back pain and depression | Multiple countries | Semi-structured interviews and thematic synthesis | Interview series with thematic synthesis and barrier analysis | This study identified that the patients perceived behavioral, affective, and cognitive improvements after completing group psychotherapy sessions. The main barriers were a lack of human contact and social isolation. |
| 94 | Schwarz B et al.2024 | Meta-ethnography | Mega-synthesis of the findings of qualitative meta-syntheses on return to work of people with various chronic illnesses | Mainly European countries | Meta-synthesis of qualitative studies | Mega-ethnography, Return to work model | This study highlighted how the conceptualization of the return-to-work process with multiple actors and interests contingent on (1) individual’s perception regarding their own limitations and chronic health condition, (2) their coping strategies and (3) motivational structures, the (4) perceived social support, and the (%) working environment’s willingness and potential to adapt work demands |
| 95 | Snell et al., 2024 | Interpretative phenomenological analysis (IPA) | 5 adult male participants with chronic fatigue syndrome | United Kingdom | Semi-structured interviews, thematic analysis | Phenomenological analysis | Hegemonic masculine norms negatively affect illness perception and help-seeking behaviors. |
| 96 | Barber et al., 2025 | Phenomenological study | Experiences of 13 individuals with chronic pain enrolled in Integrative Restoration, a secular Yoga Nidra adaptation within a multidisciplinary pain clinic setting | Australia | Semi-structured interviews, thematic analysis | Phenomenological analysis | This study demonstrated the effects of Yoga, including: (1) trauma reprocessing and integration; (2) improved pain self-management and self-efficacy, (3) dis-identification from persisting pain as personal identity, and (4) confidence to self-taper pain medications. |
| 97 | Bosco et al. 2025 | Interpretative phenomenological analysis (IPA) | Experiences of 17 patients with irritable bowel Disease (IBD) | Italy | Semi-structured interviews, thematic and interpretative phenomenological analysis. | Phenomenological analysis, Cohen's hermeneutic approach | This study highlighted the importance of addressing the unique challenges faced by rural patients with IBD, particularly, the psychological and social dimensions of geographic and social isolation. |
| 98 | Danes-Daetz et al.,  2025 | Qualitative interview study | Study of 14 female patients and their perceptions of their experience with chronic knee pain | Thailand/Malaysia | Semi-structured interviews | Shared experience Interview analysis | This study revealed that the shared experiences in managing chronic knee pain involved struggles with pain, limitations due to pain, and fear for the future. Barriers identified included exercise, stigmatization of osteoarthritis, and dived barriers into four larger categories: individual, interpersonal, organizational, and community. |
| 99 | Jiang et al., 2025 | Interpretative phenomenological analysis (IPA) | Experiences of social isolation of 20 older adults with chronic diseases in rural areas | China | Semi-structured interviews, thematic and interpretative phenomenological analysis. Consolidation of Reports of Qualitative Research (COREQ) standard was also used. Themes and sub-themes developed | Phenomenological analysis | This study found that the psychological factors influencing feeling isolated were: (1) guilt, (2) stigmatization and (3) emotional stress; social factors were: (1) lack of social support, (2) meaningless socialization, (3) deterioration of social environment. Biological contributors included: (1) distress related to chronic illness symptoms and (2) treatment confinement |
| 100 | Najem et al., 2025 | Phenomenological study | Experiences of 12 patients with chronic low back pain | Lebanon | Semi-structured interviews, thematic and interpretative phenomenological analysis. Themes and sub-themes developed | Phenomenological analysis | This study developed themes and sub-themes regarding chronic low back in a cultural context in an Arabic speaking society. Social influences of Lebanese society were explored. |
| 101 | Putri & Amir,  2025 | Phenomenological Study | Experiences of 12 participants with chronic illness | Indonesia | Semi-structured interviews with thematic synthesis | Phenomenological analysis | This study identified key themes of emotional distress, coping mechanisms, and influence of social networks and called for long-term emotional support in patients with chronic illness. |
| 102 | Shehadeh et al., 2025 | Phenomenological study | Experiences of 15 healthcare professionals treating patients with chronic illnesses | Jordan | Semi-structured interviews, thematic and interpretative phenomenological analysis. The Consolidation of Reports of Qualitative Research (COREQ) standard was also used. Themes and sub-themes developed | Phenomenological analysis | This study highlights the perspectives of healthcare professionals responsible for providing care for older people in nursing homes; Three overarching themes were identified: (1) offering dedicated courses and postgraduate programs; (2) improving facilities and resources; and (3) raising awareness and enhancing collaborations. |
| 103 | Stilwell et al., 2025 | Phenomenological study | Experiences of 12 adults living with chronic pain | Canada | In-depth, qualitative interviews, thematic and interpretative phenomenological analysis. | Phenomenological analysis | Three main themes were developed: (1) Pain-related minimal-self disruption involves a disrupted sense of agency, Theme 2: Context surrounding minimal-self disruption, Theme 3: Fluidity of the self and different modes of suffering |
| 104 | Safour et al., 2025 | Interpretative phenomenological analysis (IPA) | Experiences of 6 adult patients with chronic temporomandibular disorders | Canada | Semi-structured interviews, thematic and interpretative phenomenological analysis. | Phenomenological analysis, constructivist ontology | Four themes were developed: (1) fatigue, frustration and physical limitations, (2) communication challenges affecting self-expression in the social and professional setting, (3) adjustments and accommodations in social and professional life, (4) frustrations seeking medical help |

**Supplementary Table S6. Quality Assessment using CASP Tool**

| Study ID | Was there a clear statement of the research aims? | Is the qualitative methodology appropriate? | Was the research design appropriate to address the study aims? | Was the recruitment strategy appropriate? | Was the data collection method adequate? | Was the relationship between researchers and participants considered? | Was ethical approval obtained for the study? | Were data analysis methods sufficiently rigorous? | Is there a clear statement of findings? | Was the research valuable? |
| --- | --- | --- | --- | --- | --- | --- | --- | --- | --- | --- |
| Krohne & Ihlebaek, 2010 | Yes | Yes | Yes | Yes | Yes | Yes | Yes | Yes | Yes | Yes |
| Skjutar & Müllersdorf, 2010 | Yes | Yes | Yes | Yes | Yes | Unclear | Yes | Yes | Yes | Yes |
| Furnes & Dysvik, 2010 | Yes | Yes | Yes | Unclear | Unclear | Unclear | No | Yes | Yes | Yes |
| Vallerand & Nowak, 2010 | Yes | Yes | Yes | Yes | Yes | Yes | Yes | Yes | Yes | Yes |
| Hansson et al., 2011 | Yes | Yes | Yes | Yes | Yes | Yes | No | Yes | Yes | Yes |
| Skuladottir & Halldorsdottir, 2011 | Yes | Yes | Yes | Yes | Yes | Yes | Yes | Yes | Yes | Yes |
| Tollefson et al., 2011 | Yes | Yes | Yes | Yes | Yes | Yes | Yes | Yes | Yes | Yes |
| Lavie-Ajayi et al., 2012 | Yes | Yes | Yes | Yes | Yes | Unclear | Yes | Yes | Yes | Yes |
| Nay & Fetherstonhaugh, 2012 | Yes | Yes | Yes | Yes | Yes | Yes | Yes | Yes | Yes | Yes |
| West et al., 2012 | Yes | Yes | Yes | Yes | Yes | Yes | Yes | Yes | Yes | Yes |
| Snelgrove et al., 2013 | Yes | Yes | Yes | Yes | Yes | Yes | Unclear | Yes | Yes | Yes |
| Taylor et al., 2013 | Yes | Yes | Yes | Yes | Yes | Yes | Unclear | Yes | Yes | Yes |
| Daker-White et al., 2014 | Yes | Yes | Yes | Yes | Yes | Yes | Unclear | Yes | Yes | Yes |
| Furnes et al., 2014 | Yes | Yes | Yes | Yes | Yes | Yes | Yes | Yes | Yes | Yes |
| Juuso et al., 2014 | Yes | Yes | Yes | Yes | Yes | Yes | Yes | Yes | Yes | Yes |
| Mahon et al., 2014 | Yes | Yes | Yes | Yes | Yes | Yes | Yes | Yes | Yes | Yes |
| Mathias et al., 2014 | Yes | Yes | Yes | Yes | Yes | Yes | Yes | Yes | Yes | Yes |
| Merolli et al., 2014 | Yes | Yes | Yes | Yes | Yes | Unclear | Yes | Yes | Yes | Yes |
| Nilsen & Andersen, 2014 | Yes | Yes | Yes | Yes | Yes | Unclear | Unclear | Yes | Yes | Yes |
| Ojala et al., 2014 | Yes | Yes | Yes | Yes | Yes | Yes | Yes | Yes | Yes | Yes |
| Lima et al., 2014 | Yes | Yes | Yes | Yes | Yes | Unclear | Unclear | Yes | Yes | Yes |
| Dysvik et al., 2014 | Yes | Yes | Yes | Yes | Yes | Yes | Yes | Yes | Yes | Yes |
| Brooks et al., 2015 | Yes | Yes | Yes | Yes | Yes | Yes | Yes | Yes | Yes | Yes |
| De Villiers et al., 2015 | Yes | Yes | Yes | Yes | Yes | Yes | Yes | Yes | Yes | Yes |
| Hearn et al., 2015 | Yes | Yes | Yes | Yes | Yes | Yes | Yes | Yes | Yes | Yes |
| Kirkham et al., 2015 | Yes | Yes | Yes | Yes | Unclear | Yes | Yes | Yes | Yes | Yes |
| MacNeela et al., 2015 | Yes | Yes | Yes | Yes | Yes | Yes | Unclear | Yes | Yes | Yes |
| Ojala et al., 2015 | Yes | Yes | Yes | Yes | Yes | Yes | Yes | Yes | Yes | Yes |
| Ojala et al., 2015 | Yes | Yes | Yes | Yes | Yes | Yes | Yes | Yes | Yes | Yes |
| Ojala et al., 2015 | Yes | Yes | Yes | Yes | Yes | Yes | Yes | Yes | Yes | Yes |
| Röing & Sanner 2015 | Yes | Yes | Yes | Yes | Yes | Yes | Unclear | Yes | Yes | Yes |
| Coleman et al., 2016 | Yes | Yes | Yes | Yes | Yes | Yes | No | Yes | Yes | Yes |
| Dueñas et al. 2016 | Yes | Yes | Yes | Unclear | Yes | Unclear | Unclear | Yes | yes | Unclear |
| Rambod et al., 2016 | Yes | Yes | Yes | Yes | Yes | Yes | Yes | Yes | Yes | Yes |
| Svensson et al., 2016 | Yes | Yes | Yes | Yes | Yes | Yes | Yes | Yes | Yes | Yes |
| Angel et al., 2017 | Yes | Yes | Yes | Yes | Yes | Yes | Yes | Yes | Yes | Yes |
| Hassani et al., 2017 | Yes | Yes | Yes | Yes | Yes | Yes | Yes | Yes | Yes | Yes |
| Morton et al., 2017 | Yes | Yes | Yes | Yes | Yes | Yes | Yes | Yes | Yes | Yes |
| Palacios-Ceña et al., 2017 | Yes | Yes | Yes | Yes | Yes | Yes | Yes | Yes | Yes | Yes |
| Schütze et. al., 2017 | Yes | Yes | Yes | Yes | Yes | Yes | Yes | Yes | Yes | Yes |
| Toye et al., 2017 | Yes | Yes | Yes | Yes | Yes | Yes | Unclear | Yes | Yes | Yes |
| Toye et al. 2021 | Yes | Yes | Yes | Yes | Yes | Yes | Yes | Yes | Yes | Yes |
| Twiddy et al., 2017 | Yes | Yes | Yes | Yes | Yes | Yes | Yes | Yes | Yes | Yes |
| Hearn et al., 2018 | Yes | Yes | Yes | Yes | Yes | Yes | Yes | Yes | Yes | Yes |
| Al Kalaldeh et al., 2018 | Yes | No | No | Unclear | Yes | Unclear | Unclear | Yes | Yes | Unclear |
| Cooper et al., 2018 | Yes | Yes | Yes | Yes | Yes | Yes | Yes | Yes | Yes | Yes |
| Gutke et al., 2018 | Yes | Yes | Yes | Yes | Yes | Yes | Yes | Yes | Yes | Yes |
| Leder et al., 2018 | Yes | Yes | Yes | Yes | Yes | Yes | No | Yes | Yes | Yes |
| Lee et al. 2018 | Yes | Yes | Yes | Yes | Yes | Yes | Yes | Yes | Yes | Yes |
| Hazaveh and Hovey 2018 | Yes | Yes | Yes | Yes | Yes | Yes | Yes | Yes | Yes | Yes |
| McGeechan et al., 2018 | Yes | Yes | Yes | Ye | Yes | Yes | Yes | Yes | Yes | Yes |
| Mengshoel et al., 2018 | Yes | Yes | Yes | Yes | Yes | Yes | Yes | Yes | Yes | Yes |
| Stensland & Sanders 2018 | Yes | Yes | Yes | Yes | Yes | Yes | Yes | Yes | Yes | Yes |
| Stensland & Sanders 2018 | Yes | Yes | Yes | Yes | Yes | Yes | Yes | Yes | Yes | Yes |
| Stensland & Sanders 2018 | Yes | Yes | Yes | Yes | Yes | Yes | Yes | Yes | Yes | Yes |
| Yildirim Duman, 2018 | Yes | Yes | Yes | Yes | Yes | Yes | Yes | Yes | Yes | Yes |
| Bath et al., 2019 | Yes | Yes | Yes | Yes | Yes | Yes | Yes | Yes | Yes | Yes |
| Cheng et al., 2019 | Yes | Yes | Yes | Yes | Yes | Yes | Yes | Yes | Yes | Yes |
| Hughes et al., 2019 | Yes | Yes | Yes | Yes | Unclear | Yes | Yes | Yes | Yes | Yes |
| Rodríguez et al., 2019 | Yes | Yes | Yes | Yes | Yes | Yes | Yes | Yes | Yes | Yes |
| Schultz et al., 2019 | Yes | Yes | Yes | Yes | Yes | Yes | Yes | Yes | Yes | Yes |
| Toye et al., 2019 | Yes | Yes | Yes | Yes | Yes | Yes | Unclear | Yes | Yes | Yes |
| Mustafa et al., 2020 | Yes | Yes | Yes | Yes | Yes | Yes | Yes | Yes | Yes | Yes |
| Roenne et al., 2020 | Yes | Yes | Yes | Yes | Yes | Yes | Yes | Yes | Yes | Yes |
| Skúladóttir et al., 2020 | Yes | Yes | Yes | Yes | Yes | Yes | Yes | Yes | Yes | Yes |
| Keen et al., 2021 | Yes | Yes | Yes | Yes | Yes | Yes | Yes | Yes | Yes | Yes |
| Andersen et al., 2021 | Yes | Yes | Yes | Yes | Yes | Yes | Unclear | Yes | Yes | Yes |
| Fracso et al., 2021 | Yes | Yes | Yes | Yes | Yes | Yes | Yes | Yes | Yes | Yes |
| Honoré Grauslund et al., 2021 | Yes | Yes | Yes | Yes | Yes | Yes | Unclear | Yes | Yes | Yes |
| Niño de Guzmán Quispe et al. 2021 | Yes | Yes | Yes | Yes | Yes | Yes | Yes | Yes | Yes | Yes |
| Stensland, 2021 | Yes | Yes | Yes | Yes | Yes | Yes | Yes | Yes | Yes | Yes |
| Stensland, 2021 | Yes | Yes | Yes | Yes | Yes | Yes | Yes | Yes | Yes | yes |
| Toye et al., 2021 | Yes | Yes | Yes | Yes | Yes | Yes | Unclear | Yes | Yes | Yes |
| Chala et al., 2022 | Yes | Yes | Yes | Yes | Yes | Yes | Yes | Yes | Yes | Yes |
| Kahere et al., 2022 | Yes | Yes | Yes | Yes | Yes | Yes | Yes | Yes | Yes | Yes |
| Altun et al., 2023 | Yes | Yes | Yes | Yes | Yes | Yes | Yes | Yes | Yes | Yes |
| Chimezie, 2023 | Yes | No | Unclear | Yes | Unclear | Unclear | Yes | Yes | yes | Unclear |
| Joyce et al., 2023 | Yes | Yes | Yes | Yes | Yes | Yes | Yes | Yes | Yes | Yes |
| Kjærulff et al., 2023 | Yes | Yes | Yes | Yes | Yes | Yes | Unclear | Yes | Yes | Yes |
| Peppard et al., 2023 | Yes | Yes | Yes | Yes | Yes | Yes | Unclear | Yes | Yes | yes |
| Sun et al. 2023 | Yes | Yes | Yes | Yes | Yes | Yes | Unclear | Yes | Yes | Yes |
| van Rysewyk et al., 2023 | yes | Yes | Yes | Yes | Yes | Yes | Yes | Yes | Yes | Yes |
| Albán-Reyes et al., 2024 | Yes | Yes | Yes | Yes | Yes | Yes | Yes | Yes | Yes | Yes |
| Bruger et al., 2024 | Yes | Yes | Yes | Yes | Yes | Yes | Yes | Yes | Yes | Yes |
| Bullo et al., 2024 | Yes | Yes | Yes | Yes | Yes | Unclear | Yes | Yes | Yes | Yes |
| Gaber et al., 2024 | Yes | Yes | Yes | Yes | Yes | Yes | Unclear | Yes | Yes | Yes |
| Lilja et al., 2024 | Yes | Yes | Yes | Yes | Yes | Yes | Yes | Yes | Yes | Yes |
| Lo Monaco et al., 2024 | Yes | Yes | Yes | Yes | Yes | Yes | Yes | Yes | Yes | Yes |
| Lundin et al., 2024 | Yes | Yes | Yes | Yes | Yes | Yes | Yes | Yes | Yes | Yes |
| MacGregor et al., 2024 | Yes | Yes | Yes | Yes | Yes | Yes | Yes | Yes | Yes | Yes |
| Matias-Soto et al., 2024 | Yes | Yes | Yes | Yes | Yes | Yes | Yes | Yes | Yes | Yes |
| Nikoloudi et al., 2024 | Yes | Yes | Yes | Yes | Yes | Yes | Yes | Yes | Yes | Yes |
| Okolo et al., 2024 | Yes | Yes | Yes | Yes | Unclear | No | Unclear | Yes | Yes | Unclear |
| Sanabria-Mazo et al., 2024 | Yes | Yes | Yes | Yes | Yes | Yes | Yes | Yes | Yes | Yes |
| Schwarz B et al.2024 | Yes | Yes | Yes | Yeas | Yes | Yes | No | Yes | Yes | Yes |
| Snell et al., 2024 | Yes | Yes | Yes | Yes | Yes | Yes | No | Yes | Yes | Yes |
| Barber et al., 2025 | Yes | Yes | Yes | Yes | Yes | Yes | Yes | Yes | Yes | Yes |
| Bosco et al. 025 | Yes | Yes | Yes | Yes | Yes | Yes | Yes | Yes | Yes | Yes |
| Danes-Daetz et al., 2025 | Yes | Yes | Yes | Yes | Yes | Yes | Yes | Yes | Yes | Yes |
| Jiang et al., 2025 | Yes | Yes | Yes | Yes | Yes | Yes | Yes | Yes | Yes | Yes |
| Najem et al., 2025 | Yes | Yes | Yes | Yes | Yes | Yes | Yes | Yes | Yes | Yes |
| Putri & Amir, 2025 | Yes | Yes | Yes | Yes | Yes | Yes | Yes | Yes | Yes | Yes |
| Shehadeh et al., 2025 | Yes | Yes | Yes | Yes | Yes | Yes | Yes | Yes | Yes | Yes |

**Supplementary Table S7. GRADE-CERQual for Phenomenology Studies**

| Study Number | Study ID | Methodological Limitations | Coherence | Adequacy of Data | Relevance |
| --- | --- | --- | --- | --- | --- |
| 1 | Furnes & Dysvik,  2010 | Moderate: The paper utilizes an “open qualitative review” rather than a systematic review, which may introduce selection bias. However, the authors followed a systematic four-step process to identify subthemes and perform an abstraction to create the final framework  . As a theoretical proposal, it lacks the empirical testing found in clinical trials. | High:  The findings are highly coherent, as the authors logically link established grief theories (e.g., Attig, Parkes) to the specific experiences of chronic pain patients. The resulting framework (Figure 1) provides a consistent and integrated structure where subthemes (like despair vs. hope) feed directly into the main themes of relearning the world and adaptation. | Moderate: The “data” consists of a synthesis of 27 cited works, including classic and recent grief theories, phenomenological philosophy, and the authors' own previous empirical research. While rich in theoretical depth, the source does not provide the primary data sets or a formal assessment of the quality of the studies it reviews. | High: The paper is directly applicable to the specific context of the clinical interventions for chronic pain; it addresses a identified gap in healthcare, the lack of recognition regarding the similarities between grief from death and grief from chronic illness, making it highly relevant for healthcare workers in pain clinics. |
| 2 | Krohne & Ihlebaek, 2010 | Moderate: The study employed standard qualitative methods, including focus groups and Systematic Text Condensation, and followed ethical protocols. However, confidence is tempered by a small sample size (n=14) and recruitment through patient organizations, which the authors admit may limit transferability as these individuals may be more “proactive” than the typical patient. Additionally, the WAD diagnoses were self-reported rather than medically documented for the study. | High: There is a clear, strong link between the underlying data (verbatim participant quotes) and the identified themes of dominant symptoms and coping strategies. Furthermore, the findings, such as the fluctuating nature of pain and the use of specific coping mechanisms, are noted by the authors to be consistent with existing literature on chronic pain and WAD. | High: The researchers reported reaching theme saturation between the groups, suggesting that the data collected was sufficient to cover the relevant concepts. The use of 90-minute focus group sessions allowed for rich, in-depth discussions where participants could express experiences in their own words, providing high-quality qualitative data. | High: The study is directly relevant to its own stated objectives of exploring how individuals with chronic WAD cope on a day-to-day basis. It specifically addresses an identified gap in qualitative insight regarding the “lay health resources” and self-initiated strategies used by this population, making the findings highly applicable to clinical understanding of patient experiences. |
| 3 | Skjutar & Müllersdorf, 2010 | Moderate: The study used a robust framework for trustworthiness, including triangulation, peer debriefing, thick descriptions, and an audit trail. However, limitations exist: the use of a convenience sample of only 10 participants from a single rehabilitation unit, potential researcher bias as both authors are occupational therapists, and the presence of family members as translators for two participants, which may have restricted open communication. | High: The findings are strongly supported by the underlying data, with verbatim quotes illustrating each of the three themes. The study also demonstrates high coherence by showing how the results align with existing theoretical frameworks, such as the Shifting Perspectives Model of Chronic Illness, and other empirical research on chronic pain management. | Moderate: The study provides “thick descriptions” and uses thematic content analysis to move beyond literal descriptions to latent meanings. While the data are rich in detail, the quantity is limited to 10 participants with 30-60 minute interviews each, which is a small sample size, even for qualitative research. | High: The study is directly applicable to the experiences of people living with chronic pain. It specifically addresses the gap in clinical practice regarding the understanding of patient-perceived needs versus provider-led interventions, making it highly relevant to the context of chronic pain rehabilitation. |
| 4 | Vallerand & Nowak, 2010 | Moderate: The researchers employed several methods to enhance rigor, including inter-rater reliability (three researchers verifying codes), serial interviews for validation, and theoretical sampling to saturation. However, the authors acknowledge significant limitations. Specifically, the study is not prospective, leading to potential recall bias, and the sample was limited to two clinical sites with participants who had primarily positive experiences with opioid therapy. | High: The findings are strongly supported by the underlying data, with extensive verbatim quotes illustrating each emerged theme, such as “desperation” before treatment and "balancing" or “secrecy” after. The authors also established credibility by returning to participants for a second interview to validate that the identified themes matched their lived experiences. | High: The study reached data saturation with a sample of 22 participants, which provided a rich, “thick” description of the human experience through in-depth serial tape-recorded interviews. This qualitative approach successfully captured the complexity and multidimensionality of the patients' lives that quantitative scales commonly miss. | High: The study is highly applicable to the context of understanding the patient's perspective on chronic opioid therapy, a controversial and debated treatment modality. The findings provide clinicians and nurses with essential insights into how to act as advocates and improve the delivery of holistic care for those living with chronic non-malignant pain. |
| 5 | Hansson et. al  2011 | High: The study utilized a rigorous phenomenological approach and a clearly defined data analysis process (whole-parts-whole). The researchers actively employed "bracketing" to minimize the influence of their own preconceptions on the results. While they acknowledge a gender imbalance (only two male participants), they maintain the findings are generalizable to similar patient groups. | High: The findings are strongly supported by the data, with a clear logical thread from raw interview material to the identified “essence” and its seven constituents. The results are consistent with, and often confirm, existing literature regarding patients feeling discredited or needing to “fight” for care. | Moderate: The data are rich in quality, consisting of verbatim-transcribed interviews lasting 50-70 minutes that provided deep insights into the participants' “lifeworlds.” However, the quantity of data is limited, with a sample size of only eight participants. While the authors claim data saturation was reached, the small N and lack of male perspective slightly limit the overall adequacy. | High: The study is directly applicable to the experiences of individuals with chronic pain in health service encounters. The participants had a wide range of chronic conditions (e.g., fibromyalgia, RA, back pain) and extensive experience with the healthcare system, ranging from 3 to 45 years of living with pain. |
| 6 | Skuladottir & Halldorsdottir, 2011 | Moderate: The study utilizes a rigorous, 12-step phenomenological framework (the Vancouver School) and employs investigator triangulation and member checking (verification with participants) to enhance trustworthiness. However, the sample size is very small (n=5), and the authors explicitly state that the participants are not representative of the entire population of women in chronic pain. | High: The findings are extensively supported by direct quotes from the participants, clearly linking the raw data to the identified needs. Furthermore, the results are highly consistent with existing literature regarding the “struggle for credibility,” the impact of invisible illness, and the value of social support in chronic pain. | Moderate: The data are exceptionally rich and in-depth, derived from ten tape-recorded dialogues lasting between 60 and 120 minutes each. While the quality and depth are high, the quantity is limited by the small number of participants, which may not capture the full variety of experiences within this demographic. | High: The study is directly applicable to the research goal of identifying the needs of women suffering from chronic pain. The recruitment criteria (women aged 25-55 with chronic non-malignant pain for at least six months) and the qualitative approach ensure the findings specifically address the self-reported perspective required. |
| 7 | Tollefson et. al  2011 | Moderate: Methodological Limitations: The extent to which studies supporting the finding have flaws in design or conduct. Coherence: How well the findings are supported by the underlying data and if the finding is consistent across studies. Adequacy of Data: The richness, quantity, and quality of data support the findings in the study. Relevance: The extent to which the studies supporting the finding are applicable to the specific context of the review question. using the above domains evaluate the confindence of this paper by grading each domain as either the following high, moderate, low, or very low. include a brief justification or rationale. result should be in tabular form. | High: The findings are firmly supported by the underlying data, with specific participant quotes illustrating each of the four identified themes. Furthermore, the study is consistent with existing literature regarding pain-related reticence and the role of family dynamics in chronic pain. | Moderate: While the study provides rich, in-depth qualitative data through conversational interviews (approximately 1 hour each), the quantity of data are limited by the small number of participants. The authors noted that data collection ceased when redundancy was reached, which is standard for the methodology. | High: The study is directly applicable to the specific context of chronic pain in rural areas, addressing a noted gap in the literature regarding geographical isolation and its impact on interpersonal and professional relationships. It offers specific insights relevant for rural nursing practice. |
| 8 | Lavie-Ajayi et al., 2012 | Moderate:  The study utilizes established qualitative frameworks, specifically Interpretative Phenomenological Analysis (IPA) and grounded theory, following a rigorous protocol from the University of Oxford  . Ethical standards were maintained through university approval and informed consent  . However, the sample size is limited to six participants from a single country (Israel) recruited via snowball sampling, which may limit the breadth of the design. | High: The central finding of “narratological distress” is strongly supported by the underlying interview data, which clearly illustrates the tension between the “vivid” physical experience of pain and its “elusive” social perception. Furthermore, the authors demonstrate that their findings are consistent with existing literature in Europe and the USA regarding the marginalization of chronic pain patients. | Moderate: The researchers prioritized the richness and depth of the data, selecting the most “narratologically rich” interviews from a larger dataset for the IPA. The provided excerpts offer detailed and nuanced personal accounts of the “assault on the self.” Nevertheless, the quantity of data are constrained by the small participant pool. | High: The findings are highly applicable to the context of chronic illness, as they directly address the psychosocial impact, emotional distress, and identity struggles (biographical disruption) inherent in chronic pain. The study provides specific insights into how medical and social discourse affects a patient's internal narrative and self-trust. |
| 9 | Nay & Fetherstonhaugh  2012 | Low: The paper is explicitly described as a “case study of one,” focusing entirely on the primary author's (Nay's) own lived experiences. While it follows van Manen’s phenomenological framework, the authors acknowledge this approach is “unorthodox” and relies on personal disclosure rather than a broader study population. | Moderate: The findings, such as the importance of individual control, the impact of staff responses, and the distinction between prereflective pain and reflection, are well-supported by the four detailed personal accounts provided  . However, because it is a single-subject study, there is no cross-study consistency demonstrated within this specific work. | Low: While the data are exceptionally “rich” and provides deep, personal insights into emotional, acute, cognitive, and chronic pain, it is extremely limited in quantity, representing only one person's perspective. | High: The paper is highly applicable to its intended context, as it specifically aims to help nurses understand the "essence of pain" to improve the recognition, assessment, and management of pain in older people, particularly those living with dementia. |
| 10 | West et al., 2012 | High: The study used a robust interpretive qualitative design and phenomenological analysis appropriate for exploring lived experiences  . Rigour was maintained through data saturation, an audit trail, and having two researchers independently code transcripts to reach a consensus  . While the authors acknowledge a lack of cultural diversity in the sample, the internal methodology is sound and transparently reported. | High: The findings are strongly supported by direct participant quotes that align clearly with the identified themes of individual strength, positivity, and acceptance. Furthermore, the study’s findings are consistent with the existing literature regarding adaptive coping and the paradigm shift toward strength-based care. | Moderate: The data are rich and descriptive, providing in-depth insight into the participants' lives. However, the sample is small (10 participants) and lacks diversity, as all participants were Anglo-Saxon Australians, which may limit the breadth of the findings to a specific cultural context. | High: This paper is highly relevant as it addresses a previously identified gap: the lack of qualitative research on the meaning of resilience specifically for those with chronic pain  . It provides actionable insights for clinical nursing practice, emphasizing a shift from a deficit-based to a strength-based model of care. |
| 11 | Snelgrove et. al,  2013 | High: The study employs Interpretative Phenomenological Analysis (IPA), a well-established qualitative method suited for exploring personal meaning. It follows clear procedures, including purposive sampling, semi-structured interviews, and an iterative, idiographic analysis process. A credibility check was performed by an independent specialist to validate thematic representations. | High: The findings are explicitly supported by the data, with extensive verbatim quotes from a variety of participants (e.g., Don, Sara, Eirlys) used to illustrate each sub-theme. The authors specifically prioritized internal coherence as a validation criterion to ensure their arguments remained consistent with the participants' accounts. | Moderate: While a sample size of 10 participants is small by quantitative standards, it is robust for an IPA study, which emphasizes depth over breadth. The data are rich and detailed, derived from interviews lasting 60-90 minutes, providing sufficient material to reach thematic saturation regarding the “crucial nature of the pain” and “managing the pain.” | High: The study is directly applicable to the context of chronic low back pain (CLBP). It specifically targets patients who have lived with the condition for at least four years, and are awaiting specialized clinic treatment, providing a highly relevant perspective on the psychosocial and biomedical frameworks these patients use to understand their illness. |
| 12 | Taylor et. al,  2013 | Moderate: The study utilized established qualitative methods (Interpretative Phenomenological Analysis) and implemented an independent audit of transcripts to ensure themes were grounded in the data. However, the sample size is small (n=9), and the use of interpreters for five of the nine interviews is a noted limitation, as interpretation can change data in “unquantifiable ways.” | High: The findings are strongly supported by the underlying data, with superordinate themes, such as “pain is the enemy” and “pain and intrusive memories are connected,” consistently emerging from detailed participant accounts. The findings also show high coherence with existing theoretical frameworks like dual representation theory. | Moderate: The data are rich and descriptive, capturing complex metaphors and deeply personal experiences of torture survivors. Nevertheless, the small number of participants and the potential for lost nuance through translation mean the overall quantity and depth of data are somewhat limited. | High: This research is exceptionally relevant to its context, as it addresses a significant gap in literature regarding the interaction of pain and PTSD specifically within the torture survivor population. Its clinical implications for integrated treatment are directly applicable to the review's focus on this group. |
| 13 | Daker-White et al., 2014 | Moderate: The first synthesis was rigorous, utilizing exhaustive searches and multiple reviewers, whereas the second synthesis was more limited, relying on a single database and a single reviewer. Furthermore, the authors noted that many of the primary studies included in the synthesis were “poor” and lacked conceptual depth. | High:  Findings displayed high consistency, as the majority of studies in both syntheses were judged to be “about similar things” and were conceptually coherent. The synthesis successfully compared findings from two different time periods (1975–2001 and 2002–2009), revealing consistent themes over 35 years. | Moderate: The review included a substantial quantity of data, totaling 57 papers across both syntheses. However, the richness of the data were inconsistent, as later studies often lacked the descriptive detail and theoretical development found in earlier sociological work. | High:  The findings are highly relevant to the review's goal of understanding the lived experience of rheumatoid arthritis  . However, the relevance to a global context is slightly limited because the source material focused primarily on western, English-speaking, and clinic-based populations. |
| 14 | Dysvik et. al,  2014 | Moderate: The study utilizes a qualitatively driven mixed-method design that allows for the triangulation and validation of findings. However, the authors acknowledge that the sample size (n=34) is too small for generalizability, and they suggest that a power analysis should have been performed to select an optimal sample size. Additionally, while the questionnaires used validated components, there was no published validation for the specific standardized pain questionnaire from the Norwegian Pain Forum. | High: The findings are well-supported by the underlying data, with qualitative reports and quantitative data showing consistent results regarding the program's value in alleviating suffering. The “results narrative” effectively integrates both data streams, showing how shifts in perspective identified in written reports aligned with the improvements noted in health status and pain intensity. | Moderate: The qualitative data provides “thick and nuanced descriptions” of the participants' experiences, offering high richness. Quantitatively, most scales showed satisfactory reliability (Cronbach's alpha scores between 0.61 and 0.90). However, the quantity of data were insufficient to maintain statistical significance for several variables once the Bonferroni correction was applied for multiple testing. | High: The study is highly applicable to the clinical context of chronic pain management and the nursing role in alleviating suffering. It directly addresses the multidimensional nature of chronic pain and evaluates a specific, replicable intervention (a group CBT-based program), making the findings very relevant for healthcare providers. |
| 15 | Furnes et. al,  2014 | Moderate: The study utilizes a clear qualitative design with a phenomenological hermeneutic approach and a 6-month follow-up. While the analytical process is detailed and aims for reliability, the data collection relied solely on written reports rather than interactive interviews, which may have limited the depth of participant descriptions and the ability to clarify responses. | High: The findings are strongly supported by the underlying data, with subthemes and the main theme derived directly from identified meaning units. The authors noted that the empirical material was “mostly homogeneous,” suggesting consistent findings across the participant group. | High: The study includes a sample of 34 participants, which is substantial for a qualitative study. The authors describe the data as providing “detailed and extensive descriptions” and “rich descriptions of the topic,” which allowed for clear patterns to emerge during analysis. | High: The study is highly applicable to the context of chronic pain management, as it specifically explores the experiences of outpatients in a CBT-based group program within a clinical rehabilitation setting. |
| 16 | Juuso et. al,  2014 | High: The study follows a rigorous and well-documented phenomenological hermeneutical interpretation method. The authors clearly describe their narrative approach, ethical approvals, and efforts to restrain “preunderstanding” to ensure the probability of the interpretation. | High: The findings are strongly supported by the data, with the two main themes (“being seen as a malingerer” and “being acknowledged”) being illustrated through extensive verbatim quotes from the participants. These findings are consistently cross-referenced and supported by existing literature and philosophical frameworks. | Moderate: The data are highly rich, as interviews were extensive, lasting between 51 and 147 minutes. However, the quantity is limited to 9 participants recruited from a specific region in northern Sweden, which may not capture the full breadth of experiences across different cultural or clinical settings. | High:  The findings are highly applicable to the lived experiences of women with fibromyalgia, specifically addressing their interactions with family, the workplace, and health care personnel. The study provides direct insights into the social and clinical challenges faced by this specific population. |
| 17 | Lima et. al,  2014 | Moderate: The paper is a theoretical critique and collaboration rather than a primary empirical study. While it includes a search of the PubMed database to assess the prevalence of phenomenological literature, the search was limited to specific terms (“phenomenological and pain”) and identified a relatively small number of relevant theoretical articles. | High: The findings are supported by a consistent and logical argument that traces the historical and philosophical roots of current medical models (Cartesian dualism) to explain their failure to encompass the complex, unified experience of chronic pain. The proposed phenomenological solution follows directly from this critique. | High: The paper provides a rich and extensive quantity of theoretical data, citing 76 different sources spanning history, philosophy (Husserl, Merleau-Ponty, Heidegger), and medical science to support its claims. It offers a deep, multi-layered context for its conclusions. | High: The source is directly applicable to the context of understanding chronic pain. It specifically addresses the limitations of the dominant biomedical and biopsychosocial models in clinical practice and proposes an alternative framework tailored to the subjective experience of the patient. |
| 18 | Mahon et al.,  2014 | Moderate: The study used a convenience sample of 19 participants recruited from only one service, which the authors acknowledge limits the ability to generalize findings. However, methodological rigor was supported by the use of established qualitative descriptive designs and thematic analysis frameworks, as well as credibility checks like reflective diaries and researcher consensus. | High: The findings are strongly supported by the underlying data, with each theme (loss, anger, uncertainty, and adaptation) clearly illustrated by rich participant narratives. Furthermore, the study's results are consistent with international literature and established theoretical models of chronic illness transition, such as Kralik’s stages. | Moderate: While the two focus groups provided rich and detailed narratives regarding the participants' experiences, the authors state that the small sample size (n=19) and limited geographic focus mean a larger, more comprehensive study is required to fully capture the diversity of the Irish experience. | High: The study is highly applicable to the context of chronic illness experiences in Ireland, specifically addressing a gap in existing research. It highlights context-specific issues such as the impact of the Irish economic recession and the fragmented nature of the local healthcare system on patient outcomes. |
| 19 | Mathias et. al,  2014 | Moderate: The study employed Interpretative Phenomenological Analysis (IPA), which is appropriate for exploring individual experiences, and included validity checks such as participant feedback on findings. However, limitations exist due to the small, homogeneous sample (six white British females) and potential social desirability bias, as the researcher was supervised by one of the program facilitators. | High:  The findings are strongly supported by the underlying data, with five clear themes emerging consistently from verbatim participant extracts. Furthermore, the findings are consistent with established theoretical frameworks including Acceptance and Commitment Therapy (ACT), CBT, and mindfulness. | Moderate: The data are qualitatively rich, providing deep idiographic insights into the “double hermeneutic” of participant experiences. However, the quantity of data are limited by the small sample size and recruitment from a single community outpatient site, which the authors acknowledge may limit the wider applicability. | High: The study is highly applicable to the context of chronic pain management, as it specifically identifies which components of an acceptance-based PMP (such as mindful movement, pacing, and group validation) were perceived by patients as facilitating positive change. |
| 20 | Merolli et al., 2014 | Moderate: The researchers employed a rigorous five-phase thematic content analysis (TCA) process involving three independent investigators to ensure consistency. However, the authors acknowledge significant limitations: they were unable to calculate a response rate due to viral dissemination, the sample was subject to self-selection bias, and the qualitative nature of the analysis is open to interpretive bias. | High: The findings are strongly supported by the underlying data. The authors clearly documented how they moved from initial deductive coding to an inductive analysis of 155 quotes to refine their “therapeutic affordances.” The final SCENA model (Self-presentation, Connection, Exploration, Narration, Adaptation) directly reflects the language and themes extracted from participant responses. | Moderate: While the survey had 218 total participants, the qualitative findings are based on a subset of 68 users (31.2%) who provided 117 free-text responses. This provided a rich dataset for themes like “exploration” (52 quotes), but other categories like “self-presentation” were supported by much thinner data (only 7 quotes). | High: The study is highly applicable to the context of chronic disease self-management. It specifically targets people with chronic pain (PWCP) to understand the “how” and “why” behind their use of various social media platforms. The resulting SCENA model is purpose-built to guide clinical decision-making and further health informatics research. |
| 21 | Nilsen & Anderssen,  2014 | Moderate: The study utilizes a well-defined phenomenological-hermeneutical approach and narrative analysis. However, the authors acknowledge limitations, including a sample that is not statistically representative and a lack of method triangulation. The data were also generated in an interactive interview setting, which may influence the results. | High: The findings are strongly supported by underlying data, with clear links between the raw interview narratives and the emerging themes such as the importance of work and the use of nature as a strategy. The results are also framed consistently within Bourdieu’s theoretical concepts of habitus and capital. | High: The study provided rich, high-quality data through in-depth interviews lasting 2-3 hours each with 20 participants. This allowed for “pain stories” to emerge systematically, providing a deep volume of qualitative material for the thematic analysis. | High: The findings are highly applicable to the study's specific aim of exploring how individuals with chronic non-malignant pain manage their condition in their everyday lives  . The context-specific results (e.g., the role of the Northern Norway environment) provide direct insights into individual self-care strategies. |
| 22 | Ojala, et. al  2014 | Moderate: The study used an established phenomenological method (Giorgi) and a multidisciplinary team for data analysis to enhance credibility. However, the authors noted limitations: transcripts were not returned to participants for comment, and results were not checked by participants (member checking), which can be a standard way to ensure accuracy in qualitative research. | High: The findings are highly consistent and logically supported by the underlying data. The subthemes (e.g., “Pain is the master,” “Life is not worth living”) directly emerge from the participants' rich, detailed descriptions of their experiences. The researchers also successfully integrated their findings with existing biopsychosocial literature. | High: The study utilized a robust sample size for qualitative research, consisting of 34 participants from diverse sources. Data collection resulted in 631 pages of transcribed interviews, and the researchers explicitly stated that data saturation was reached by the 34th interview, meaning no new meaning units were being discovered. | High: The study is highly applicable to understanding the lived experience of patients with chronic pain in a clinical context. It specifically addresses how pain impacts the biopsychosocial well-being of patients, offering insights into why multidisciplinary and patient-centered care is necessary for this population. |
| 23 | Brooks et. al, 2015 | Moderate: The study used a rigorous Interpretive Phenomenological Analysis (IPA) framework, involving audio-recorded, verbatim transcriptions and a six-step thematic process. However, a significant limitation was the lack of follow-up; while all nine participants committed to two interviews for deeper clarification, only four completed the second one. | High: The findings are strongly supported by the underlying data, with direct participant quotes clearly illustrating each of the eight identified themes. The results, such as the theme of “finding balance,” are also consistent with previous qualitative research in the field. | Moderate: The data are thematically rich, capturing complex emotional experiences like stigma, guilt, and self-protection. However, the quantity is limited by a small sample size (n=9) and the aforementioned failure to secure follow-up interviews, which restricted further elaboration and clarification of themes. | High: The study is highly applicable to the context of chronic pain management, as it directly explores the lived experiences of adults using prescription opioids for CNCP to understand the psychological and social impacts beyond mere physical pain relief. |
| 24 | De Villiers et. al,  2015 | Moderate: The study employed a rigorous descriptive phenomenological design and utilized Guba’s trustworthiness constructs, including triangulation, member checks, and bracketing to manage researcher bias. However, the sample was small (n=9) and predominantly female, which the authors admit may not represent male perspectives. Additionally, while interviews were in English, the use of a translator for “language issues” and the researcher's dual role as a clinic nurse could introduce subtle biases. | High: The findings are strongly supported by rich, verbatim data and participant quotes that align clearly with the identified themes of physical, psychosocial, and spiritual impact. The results are internally consistent and the authors successfully link their findings to established theoretical frameworks, such as Cecily Saunders' concept of “total pain.” | Moderate: The data are highly rich and qualitative, involving hour-long in-depth interviews that captured non-verbal cues like sighing and crying to ensure depth. While the researchers conducted concurrent analysis to determine data saturation, the total number of participants (n=9) is relatively low, which may limit the breadth of the data. | High:  The study is highly applicable to understanding chronic pain in resource-poor, underprivileged contexts  . It specifically addresses a “knowledge gap” regarding the daily lived experiences of South Africans serving as a direct response to the failures of the public health care system in that region. |
| 25 | Hearn et. al,  2015 | Low: The study employed a rigorous Interpretative Phenomenological Analysis (IPA) framework. To ensure quality, the researchers used “bracketing” via a reflective diary to manage bias and employed independent auditors to check that themes remained grounded in the data. While the sample was small (n=8) and self-selecting, this is consistent with the idiographic requirements of qualitative research to provide depth over breadth. | High: The findings are highly coherent, with super-ordinate themes supported by clear convergences across the accounts. For example, “the chasm” between biomedical perspectives and patient needs was prevalent across all eight participants. The authors successfully mapped the individual sensory experiences (e.g., “pit of fire”) to broader psychological and social consequences. | High: The data are exceptionally rich, derived from verbatim transcripts of semi-structured interviews lasting between 40 and 120 minutes. The authors included sufficient raw data in the results to validate their interpretations and provided a detailed table of participant characteristics to contextualize the findings. | High: The study directly addresses a “dearth of evidence” regarding the psychological and social aspects of pain management specifically for the SCI population  . The findings offer high clinical relevance by suggesting a shift in rehabilitation from purely pharmacological pain relief toward biopsychosocial pain management and acceptance-based therapies. |
| 26 | Kirkham et. al,  2015 | Low: The study follows a rigorous and systematic Interpretative Phenomenological Analysis (IPA) procedure, adhering to established qualitative quality criteria (e.g., Yardley, 2008). While the sample size is small and homogeneous (n=7 White British women), the authors note this is a standard and intentional feature of IPA designed to allow for deep idiographic analysis. | High: The findings are strongly supported by the data, with a “triple hermeneutic” approach that grounds interpretation in the participants' own accounts of their artwork. There is clear consistency across the themes (e.g., the "Pain as Object" theme) as similar visual metaphors and descriptions were found across multiple participants, such as the use of spikes and jagged shapes to represent suffering. | High: The data are exceptionally rich, utilizing both visual (drawings) and verbal (in-depth interviews lasting 45-70 minutes) representations. The authors provide “sufficient density of evidence” for each theme, by including both the original images and verbatim transcripts that capture the nuanced, subjective experience of pain. | High: The study is highly applicable to the understanding of chronic pain, an “invisible” condition that is notoriously difficult for patients to articulate. By using pictorial representations, the research directly addresses clinical needs for better methods to investigate the "hidden, elusive qualities of pain" and offers practical suggestions for therapeutic interventions. |
| 27 | MacNeela et al., 2015 | High: The authors utilized a rigorous, stepwise meta-ethnographic methodology to re-analyze existing findings. They applied Meyrick’s (2006) criteria for quality appraisal to evaluate the transparency and systematicity of each study, specifically excluding those that omitted critical information on interview content or researcher interpretation. | High: The findings are well-supported by reciprocal translation, which identified cross-study similarities, and refutational analysis, which accounted for divergent findings. These were integrated into a final “line of argument” that logically connects the themes of distress, loss, and self-management. | High: The paper synthesizes a substantial volume of data from 38 peer-reviewed articles (representing 28 empirical investigations) published over 17 years. The sample included studies from multiple disciplines (psychology, medicine, physiotherapy, nursing) and various healthcare settings. | High: The studies were selected based on strict inclusion criteria requiring a qualitative research design focused specifically on the subjective experience of chronic low back pain. This ensures that the findings are directly applicable to understanding the personal and social impact of the condition. |
| 28 | Ojala et al., 2015  (a) | High: The study employed a rigorous four-phase phenomenological method (Giorgi’s method) designed to minimize bias through bracketing (epoche). The research process was transparent, utilizing a team-based approach to verify meaning units and perspectives, which enhances the trustworthiness of the results. | High: The findings are logically structured into four essential, overlapping themes, Invisibility, Negativity, Dominance, and Affectivity, that directly emerge from the four-phase analysis. The paper demonstrates a clear, consistent link between the underlying interview data and the final synthesis of the “essence” of chronic pain. | High: The study utilized a relatively large sample for qualitative research, consisting of 34 participants from diverse sources. This resulted in a rich dataset of 631 transcribed pages of in-depth interviews, and the author confirmed that data saturation was reached by the final interview. | High: The findings are highly applicable to the core research aim of understanding the lived experience of chronic pain from a patient's perspective. It successfully addresses the multidimensional nature of the condition, providing insights into the psychological, social, and pedagogical aspects often neglected in traditional medical models. |
| 29 | Ojala et al., 2015 (b) | High: The study follows a rigorous and well-documented qualitative framework using Giorgi’s four-phase phenomenological method. Researchers employed clear inclusion criteria, ethical oversight, and a multidisciplinary team to perform the final synthesis, which enhances the credibility of the analysis. | High: The findings are consistently supported by the underlying data, with subthemes (such as “Being disbelieved” and “Denying pain”) clearly linked to specific participant experiences and direct quotes. Furthermore, the results align with existing literature regarding the psychosocial impact of “unseen” chronic pain. | High: The data are exceptionally rich, comprising 631 transcribed pages from in-depth interviews with 34 participants. The authors explicitly noted that data saturation was reached by the final interview, ensuring a comprehensive exploration of the phenomenon. | High: The study is directly applicable to understanding the lived experience of chronic pain and the limitations of the traditional medical model. This study provides deep insight into how the “invisibility” of pain affects patient interactions with the healthcare and social security systems. |
| 30 | Ojala et. al, 2015 (c) | Moderate: The study employed a rigorous and recognized four-phase phenomenological method (Giorgi’s) to analyze data. It utilized a multidisciplinary team for synthesis to ensure credibility and practiced “bracketing” to minimize researcher bias. However, the researchers noted that results were not returned to participants for checking, a common qualitative validation step, and acknowledged that some participants might have exaggerated their experiences since it was their first time being heard. | High: The findings are highly coherent, as the researchers clearly outlined how individual “meaning units” were transformed into “meaning structures” and eventually synthesized into a comprehensive “eidetic structure.” The resulting seven subthemes (e.g., State of reflection, Acceptance of pain) are logically interconnected and supported by direct participant quotes and existing literature on the biopsychosocial paradigm of pain. | High: The study is supported by a large volume of data, including 631 pages of transcribed interviews from 34 participants. This sample size is substantial for a qualitative phenomenological study, and the researchers explicitly stated they reached data saturation, as no new meaning units emerged by the final interview. The participants also represented a diverse range of ages and chronic pain diagnoses. | High: The study is directly relevant to its aim of exploring the subjective meaning and management of chronic pain from the patient's perspective. By focusing on successful management strategies and the “altered self,” the findings provide actionable insights for clinical practice, emphasizing the need for a holistic, conversational approach in therapy. |
| 31 | Röing & Sanner 2015 | Moderate: The authors followed a systematic five-phase meta-ethnographic process to synthesize the data. However, they identified significant design flaws in many of the original 12 studies, such as the failure to develop an “outcome space” or establish logical relations between categories, which are core requirements of the phenomenographic method. | High: The findings are strongly supported by a clear audit trail showing the translation of original study constructs into the synthesized key phrases. The resulting four categories are logically related in a process of “recurring loops,” and the authors demonstrate that these findings are consistent with broader literature on chronic illness experiences, such as profound fatigue and identity threats. | Moderate: The synthesis is based on 12 selected articles representing a total of 148 individuals across various chronic conditions. While the data are rich, the authors acknowledge that certain areas, specifically identity, lacked adequate data in the heart and renal failure studies, which they attribute to either incomplete original analyses or the “struggle for pure survival” overshadowing other concerns. | High: The study is highly relevant to understanding the qualitative variations in patients' lived experiences of chronic illness. By focusing on the ways illness is experienced rather than individual case studies, it provides a comprehensive framework (the outcome space) that is directly applicable to improving patient education and empowerment in healthcare settings. |
| 32 | Svensson et. al,  2015 | High: The study utilized a rigorous phenomenological hermeneutical design with a clear three-stage analysis process (naïve understanding, structural analysis, and comprehensive understanding). Trustworthiness was strengthened through continuous discussion between authors to validate interpretations and by addressing the researcher's pre-understanding. | High: The findings are strongly supported by the narrative data, with clear themes like “struggling to understand a deceiving body” and “breakthrough pain fueling fear” emerging directly from the analysis. The results are also consistent with previous studies regarding the social isolation and lack of healthcare support experienced by this population. | Moderate: While the interviews provided “rich data” and were lengthy (50-75 minutes), the sample size was small (10 women) and limited to those living in their own homes in a single Swedish city. The study specifically excluded more frail individuals living in supportive housing, which may limit the breadth of the findings. | High: The study is directly applicable to the clinical and social context of the review question, as it focuses specifically on the lived experience of older women with VCF, a group often marginalized or “abandoned” by current medical and surgical treatment models. |
| 33 | Coleman et. al,  2016 | Moderate: The study followed rigorous Interpretative Phenomenological Analysis (IPA) guidelines and included quality assurance steps like independent audits and participant validation. However, the sample was recruited from a single support group, which the authors acknowledge may lead to self-selection bias, as members might have different narratives than non-members. | High: The findings are strongly supported by underlying data, with themes directly emerging from rich verbatim quotes from participants describing their pain (e.g., “woodpecker,” “hammering,” or “lightning”). The results are consistent with the limited existing qualitative literature on SCD pain. | Moderate: The data are highly rich and in-depth, with interviews lasting up to 120 minutes and involving detailed multi-layered coding. However, the quantity of data were limited by the small sample size of only seven participants, and the authors note that those with the most severe pain might have been unable to attend the recruitment site. | High: The study is directly applicable to the specific context of understanding the lived experience of SCD pain. It addresses a significant gap in research by focusing on the meaning and nature of pain rather than just its consequences or quantitative measurement. |
| 34 | Dueñas et al. 2016 | Moderate:  The authors utilized a structured search across major databases (Web of Knowledge, PubMed, Science Direct) and assessed study quality using the STROBE checklist. However, the paper is a narrative review rather than a systematic one, and the authors intentionally included seven low-quality studies to address topics where data were otherwise scarce. | High: The findings are synthesized from 68 included studies and are summarized according to defined, logical questions. The conclusions regarding the impact of chronic pain on physical function, mental health, and the health care system are consistently supported across these diverse studies and align with the overarching biopsychosocial model. | High: The review draws from a rich and large quantity of data, including 68 studies that encompass various methodologies (cross-sectional, longitudinal, qualitative). Sample sizes in the supporting data are substantial, with some cited population surveys including over 17,000 or 28,000 participants. | High: The studies selected and the subsequent findings are directly applicable to the review's core objective. The search terms and eligibility criteria were specifically designed to capture the multidimensional impact of chronic pain on the individual, their family, their work, and health care resources. |
| 35 | Rambod et. al,  2016 | Moderate: The study utilizes a well-defined hermeneutic phenomenological approach and follows van Manen’s six-step framework for analysis. It explicitly addresses trustworthiness through member checking, peer debriefing, and an audit trail. However, the sample is limited to a single hemophilia center in Iran, and the authors acknowledge a limitation regarding the historical lack of access to replacement factor for some participants, which may have influenced their long-term pain experiences. | High: The findings are strongly supported by the underlying data, with clear thematic links between the participants' lived experiences and the resulting themes of physical, psychological, and social impairment. The study also demonstrates consistency by aligning its results with existing quantitative and qualitative literature on chronic pain and hemophilia. | Moderate: The study gathered “experientially rich information” from 14 participants, which is generally considered sufficient for a phenomenological study to reach thematic saturation. While the data are deep and includes detailed personal anecdotes, the scope is restricted to a specific cultural and clinical setting in Shiraz, Iran. | High: The study is directly applicable to the lived experience of hemophilia, focusing specifically on the meaning of pain in this unique population. It addresses the specific nuances of hemophilia, such as pain resulting from recurrent joint bleeds since childhood, which distinguishes it from other chronic pain conditions. |
| 36 | Angel et. al,  2017 | Low: The study employs a rigorous Ricoeurian phenomenological-hermeneutic approach with clearly defined steps for interpretation (naïve, structural, and critical). It utilizes a purposeful sample of 25 informants from a previous randomized controlled trial, ensuring participants had diverse experiences with the intervention. Rigor was maintained through constant data consultation and collaborative analysis between researchers. | High: The findings are strongly supported by the underlying data, with a clear and logical link between participant experiences and the identified master narratives. The study provides a detailed table (Table 1) summarizing the situation of all 25 participants and uses extensive direct quotes to illustrate and validate the themes of “getting on with life” versus “being stuck.” | High: With 25 informants, the study offers a substantial sample size for qualitative research, allowing for rich, in-depth accounts of the lived experience. The data are particularly valuable due to its longitudinal nature, capturing perspectives 2-4 years after the initial intervention, which provides significant depth regarding long-term coping and identity shifts. | High: The study is highly applicable to clinical and rehabilitative contexts for chronic pain management. It directly addresses how patients understand themselves and their future, thus providing specific insights into the role healthcare professionals play in helping patients configure meaningful recovery narratives. |
| 37 | Hassani et al., 2017 | High: The study employed a rigorous descriptive phenomenological approach using Colaizzi’s nine-step method for data analysis. Trustworthiness was established through Lincoln and Guba’s criteria, including member checking (returning to participants for validation), peer debriefing, and the use of a supervisor to independently code data to ensure credibility and dependability. | High: The findings are highly coherent, as the extracted themes and subthemes, such as “patience and trust in God” and “aversion to physical dependence,” are consistently supported by rich, verbatim participant quotations. The authors also logically connect their results to existing literature while explaining how cultural and religious contexts influence the specific meaning of resilience in this population. | High: The researchers achieved data saturation after 24 interview sessions with 22 participants. The depth of data are significant, with 767 significant statements and phrases extracted to form the basis of the analysis. The study also provides a detailed demographic breakdown of the participants to ensure transparency. | High: The study is directly applicable to understanding the experience of resilience in elderly populations with chronic illness. It specifically targets hospitalized older adults (aged 65+) and explores the personal, external, and contextual factors that facilitate or hinder their ability to adapt to health challenges. |
| 38 | Morton et al., 2017 | High: The majority of the contributing primary studies (20) met all or most of the NICE checklist criteria for quality. Only three studies were of low quality, and their limitations were unlikely to significantly alter the overall conclusions. | High: The findings were consistently supported by the underlying data across a range of chronic conditions and digital intervention (DI) designs. Where discrepancies existed, they were generally explained by specific factors such as DI design or patient beliefs. | Moderate: While the primary findings were supported by rich and varied data across many studies, some specific areas, such as perceptions of medication changes, relied on relatively “thin data” from the contributing papers. | High:  All studies included in the review were highly applicable to the review question, as they focused specifically on adult populations with chronic physical illnesses using digital self-management tools. |
| 39 | Palacios-Ceña et. al,  2017 | High: The study utilized a robust qualitative phenomenological design that included bracketing to mitigate researcher bias. It adhered to the Consolidated Criteria for Reporting Qualitative Research (COREQ) and employed triangulation, external auditing, and patient verification to ensure rigour. Minor limitations, such as the exclusion of male participants and conducting some interviews in coffee shops, are transparently acknowledged. | High: The emerging themes are strongly supported by rich, verbatim narratives and patient drawings. The findings are consistently aligned with previous qualitative research regarding the “invisible” nature of migraine and the sense of helplessness experienced by patients. | High:  The researchers achieved information redundancy (saturation) with 20 participants. The data set is extensive, comprising 1,951 minutes of recorded interviews, supplemented by researchers’ field notes and symbolic drawings from the patients. | High: The study is highly applicable to clinical contexts involving chronic migraine (CM), specifically targeting women, who are disproportionately affected by the condition. It provides specific insights into doctor-patient relationships and the impact of CM on work and family life. |
| 40 | Schütze et. al,  2017 | High: The study employed a clear and rigorous design using Interpretative Phenomenological Analysis (IPA), which is appropriate for exploring subjective experiences. It utilized a purposive sample with strict inclusion criteria, specifically a score of ≥ 30 on the Pain Catastrophizing Scale, to ensure the participants were relevant to the research aims. Validity was further strengthened by following COREQ guidelines and conducting an independent audit of all transcripts and coding by a second author. | High: The findings are strongly supported by the underlying data, with a clear “hermeneutic circle” established between participant quotes and abstracted themes. There is striking congruence across participants regarding triggers for rumination and the internal conflict between positive and negative metacognitions. Furthermore, the qualitative results align with existing quantitative pain research and established theoretical models like the S-REF model. | High: The data are exceptionally rich, providing thick descriptions of the idiographic perspectives of 15 participants, which is considered a “fairly large sample” for the IPA methodology. The study provides detailed verbatim quotes that illustrate nuanced psychological states, such as “stinkin’ thinkin’” and the “misery-go-round,” ensuring the data’s quality and depth. | High:  The study is highly applicable to the context of chronic pain management, as it specifically targets a clinical population (Chronic Low Back Pain) with high levels of a known poor-outcome predictor (Pain Catastrophizing). The findings provide direct, actionable insights for the development of Metacognitive Therapy (MCT) and other “third wave” psychological interventions. |
| 41 | Toye et al., 2017 | High: The study employed a rigorous and transparent methodology based on established meta-ethnography frameworks. The authors used a systematic search strategy with clear inclusion/exclusion criteria and utilized multiple reviewers to challenge and resolve interpretations, ensuring findings remained grounded in the data. While they acknowledge the lack of consensus on quality appraisal for primary qualitative studies, they prioritized conceptual insight over purely methodological reporting. | High: The findings demonstrate strong consistency across the included syntheses. Major conceptual categories, such as “My life is impoverished and confined” and “Deciding to end the quest for the grail,” were consistently supported by 7 out of the 11 included Qualitative Evidence Syntheses (QES). The final “Line of Argument” synthesizes these categories into a logical and cohesive narrative. | High: The “mega-ethnography” is supported by an exceptionally large and rich dataset. It synthesizes 11 QES, which collectively represent 155 unique primary qualitative studies (reported across 187 papers) and encompass the experiences of more than 5,000 participants (n = 5236). | High: The studies included were highly specific to the review question. The authors explicitly screened and excluded studies that did not focus on patients' experiences, non-malignant pain, or chronic conditions to ensure that the final synthesis directly addressed the core phenomenon of interest. |
| 42 | Twiddy et. al,  2017 | Moderate: The study employed a clear descriptive qualitative design with purposive sampling and researcher triangulation (including an external researcher) to enhance rigour. However, it is limited by a small sample size (18 participants) recruited only from a tertiary level pain team, which the authors admit may not reflect the experiences of young adults managed in primary care. | High: The findings are consistently supported by the underlying data, with four clear themes (thwarted opportunities, peer separation, illness validity, and dependency) illustrated through direct participant quotes. The researchers also used a systematic phase in which subsequent focus groups verified data from previous ones to ensure consistency. | Moderate: The study collected rich and meaningful data through focus groups, and the researchers reported reaching theoretical saturation. Nevertheless, the data comes from only 18 participants in four groups, which is a relatively small volume for broad generalizations. | High: The findings are directly applicable to the specific context of understanding the developmental and service needs of emerging adults (18-30 years) with chronic pain. The study specifically targets a gap in qualitative research for this unique population within the UK's National Health Service (NHS). |
| 43 | Al Kalaldeh et al., 2018 | Moderate: The authors used established tools like the CASP checklist to appraise the 26 included studies, ensuring they met basic qualitative research components. However, the search was limited to English-language publications and the time range (1995–2015) was chosen “conveniently” rather than through a more rigorous systematic justification. Additionally, some included studies had limited transferability due to being disease-specific or failing to distinguish between manifest and latent observations. | High: The findings are well-supported across multiple studies, consistently identifying dimensions of suffering such as hopelessness, loss of autonomy, and spiritual pain. The synthesis successfully links various phenomenological approaches (descriptive and hermeneutic) to a unified conclusion that these methods are essential for capturing the complex, subjective nature of chronic suffering. | High: The synthesis is based on 26 qualitative studies selected from an initial pool of 894 papers. The data set is rich and diverse, covering various chronic conditions, including cancer, end-stage renal disease, and postpartum psychosis, across different geographical locations such as Sweden, Finland, Israel, and Taiwan. | High: The studies included are highly applicable to the review's core question. The inclusion criteria strictly targeted qualitative research using phenomenology to explore suffering specifically in chronically ill patients. The findings directly address the application of these philosophical constructs within the specific context of nursing and healthcare for long-term illness. |
| 44 | Cooper et al.,  2018 | High: The study employed a clear and appropriate qualitative design (Interpretative Phenomenological Analysis) framed by the Transtheoretical Model  . Rigor was maintained through verbatim transcription, reflexive field notes, independent coding by a second reviewer, and member checks with participants  . The authors also transparently acknowledged limitations regarding sample demographics. | High: The three superordinate themes are well-supported by extensive verbatim extracts from participants, ensuring the findings are grounded in the raw data. Furthermore, the findings regarding the “vicious cycle” of weight and pain and fear-avoidance are consistent with existing theoretical models and previous quantitative research. | Moderate: While the sample of 18 participants is robust for an IPA study and data saturation was reportedly reached, the typical interview length of 25 minutes is relatively brief for “in-depth” qualitative research. This may slightly limit the richness of the data compared to longer, more exhaustive interviews. | High: The study is directly applicable to the specific context of the review question, as it focuses exclusively on the lived experiences and perceptions of adults with both overweight/obesity and chronic musculoskeletal pain. The findings offer specific, actionable insights for healthcare professionals in clinical practice. |
| 45 | Gutke et al.,  2018 | High: The study employed the rigorous Empirical Phenomenological Psychological (EPP) method, which involves systematic steps like phenomenological reduction to minimize researcher bias. To further ensure objectivity, the researchers used independent interviewers with no prior relationship to the participants or clinical experience in the field. Participants were also selected using well-defined clinical diagnostic criteria. | High: The findings are strongly supported by the underlying data, with the researchers identifying clear constituents and typologies (Ongoing Struggle vs. Adaptation and Acceptance) that directly reflect the participants' narratives. The findings are also consistent with previous qualitative research that identified similar struggles in women with postpartum pelvic girdle pain. | Moderate: While the sample size is small (9 participants), which is typical for idiographic qualitative research, the data are exceptionally rich. Interviews lasted up to 120 minutes, and the participants provided a unique long-term perspective, reflecting on experiences spanning 2 to 13 years of living with the condition. | High: The study is highly applicable to the specific context of understanding the existential and long-term impact of chronic pregnancy-related pelvic girdle pain. It addresses a noted gap in literature by focusing on women many years after delivery, rather than just the immediate postpartum period. |
| 46 | Hearn et. al,  2018 | Moderate: The study employs Interpretative Phenomenological Analysis (IPA), a recognized qualitative method, and uses independent auditors to ensure the themes are grounded in the data. However, the authors acknowledge a small (n=8), self-selecting sample that was primarily over age 60, which may not represent the broader, more diverse spinal cord injury (SCI) population. | High: The findings demonstrate strong internal coherence; the three super-ordinate themes are directly and transparently supported by rich verbatim quotes from the participants  . The analysis follows a systematic approach to ensure themes remain grounded in the data throughout the process. | Moderate: While a sample size of eight is considered methodologically appropriate for the idiographic depth required by IPA, the data are drawn from a single specialist center. The interviews were detailed (40-60 minutes), providing “thick” description; however, the quantity of data are limited by the small cohort size. | High: The study is highly specific to the context of neuropathic pain (NP) within a rehabilitation setting for those with SCI. It directly addresses the “critical time window” of early rehabilitation, making it extremely applicable to understanding inpatient experiences and adjustment. |
| 47 | Leder, 2018 | Moderate: The paper utilizes a phenomenological approach, which is inherently subjective and exploratory rather than quantitative  . While it is externally peer-reviewed and written by an expert with both medical (MD) and philosophical (PhD) credentials, it relies on personal anecdotes, biographical notes, and selected literary pathographies rather than a standardized, blinded, or controlled study design. | High: The findings are strongly supported by the underlying data. The author systematically maps four specific experiential commonalities, constriction of space, disruption of time, isolation, and disempowerment, across both populations. The parallels drawn between the “internal” prison of the body and the “external” prison of the state are logically consistent and reinforced by direct quotes from both groups. | Moderate: The data are highly rich in quality, drawing from “pathographies” (first-person illness narratives) and transcripts of philosophical discussions with maximum-security prisoners collected over decades. However, the quantity is limited to a small selection of specific voices (e.g., three academics and a few named inmates), which may not represent the full diversity of these populations. | High: The paper is directly applicable to its specific context. It explicitly seeks to bridge the gap between those suffering from chronic pain/illness and those in long-term incarceration. The strategies for coping (adaptability, mental freedom, etc.) are derived directly from the “personal authority” of individuals living in these exact circumstances. |
| 48 | Lee et al, 2018 | Moderate: small purposive sample (n=8) and potential researcher influence (interviewer was an experienced physiotherapist) though IPA appropriate; limited generalizability (Methods, pp.3–4). Interpretative Phenomenological Analysis (IPA). | High: clear analytic process (line-by-line IPA, NVivo, team review, patient advisory and clinician triangulation) and well-linked themes (Analysis/Results, pp.4–6). Experiences of 8 patients diagnosed with COPD experiencing moderate to severe COPD pain. | Moderate: rich, in-depth interviews (40–90 min) produce detailed accounts, but sample size and limited diversity constrain breadth (Table 2, pp.4–5). | High: addresses an understudied, clinically important issue (pain in COPD), with clear implications for pulmonary rehabilitation and psychosocial management (Introduction/Discussion, pp.2,7). Semi-structured interviews with thematic synthesis. consistent and plausible findings supported by strong analytic procedures, but confidence tempered by small sample size and limited generalizability. |
| 49 | Hazaveh and Hovey,  2018 | Moderate: The study utilized a rigorous interpretive phenomenological approach with verbatim transcription and dual-author analysis to ensure credibility. However, the authors acknowledge limitations including a very small sample size (n=6), recruitment from only two local sites in Montreal, and a significant gender imbalance (5 women, 1 man), which limits the breadth of the findings. | High: The findings are highly coherent, with a clear and logical link between the raw interview data (direct quotes) and the identified themes of loss, disbelief, and healthcare dissatisfaction  . Furthermore, the authors demonstrate that their findings are consistent with the existing qualitative literature on chronic pain and identity. | Moderate: While the number of participants is low, the depth of data are significant, with individual interviews lasting between 60 to 120 minutes. The researchers noted they reached a point where “no further information was necessary,” suggesting thematic saturation for this specific group. However, the small participant pool inherently limits the richness compared to larger qualitative studies. | High: The study is directly applicable to the clinical understanding of chronic orofacial pain. It specifically addresses a gap in the literature by moving beyond biomedical measurements (like 0-10 scales) to explore the subjective psychosocial impact on patients, which is highly relevant for healthcare providers. |
| 50 | McGeechan et al.,  2018 | Moderate: The study utilizes a longitudinal design and follows established quality principles (Yardley, 2000) for qualitative research. However, it relies on a very small convenience sample (n=6), and the authors acknowledge limitations such as an unknown recruitment rate and the use of gatekeepers who may have introduced bias. | High: The findings are clearly supported by the underlying data, with verbatim quotes directly illustrating the identified themes  . The results are consistent with existing theories like biographical disruption while extending the specific concept of “future disorientation” to a new population. | Moderate: While the longitudinal approach (two interviews over six months) provides richness and depth, the total volume of data are limited by the small participant pool and the loss of one participant at follow-up. Additionally, interview lengths varied significantly (18 to 120 minutes), which may impact the consistency of data depth. | High: The study is directly applicable to understanding the psychosocial and physical consequences of living with cancer as a chronic illness. It specifically addresses a research gap by including male perspectives and exploring how these experiences evolve over time. |
| 51 | Mengshoel et al., 2018 | Moderate: The authors utilized a systematic search and the QUARI appraisal tool to evaluate primary studies. However, they noted that many primary studies lacked information on reflexivity and the researchers' philosophical or theoretical perspectives. | High: The synthesized findings regarding validation and legitimation were consistent across the 28 reports. The authors maintained a clear analytical connection between the primary study extracts and the overarching concepts identified. | High: The paper draws on a rich and large dataset comprising 28 reports and 475 informants from diverse geographic locations, including Europe, North America, Asia, and Africa. The data covers a wide range of ages (16-80) and time since diagnosis. | High: The study selection was highly specific to the review question, as researchers screened 93 papers to identify the 28 that explicitly included detailed information on patients' diagnostic experiences with fibromyalgia. |
| 52 | Stensland & Sanders,  2018 | Moderate: The study utilizes an established qualitative framework (van Manen’s phenomenology) and describes clear steps for recruitment, data collection, and iterative thematic analysis. However, the authors admit to using non-probability convenience sampling and acknowledge a significant lack of racial and ethnic diversity, as all participants were Caucasian. | High: The findings are strongly supported by the underlying data, with themes directly reflected in numerous detailed participant narratives. The results are logically organized by system levels (from self to society) and show consistency with existing, albeit limited, literature on chronic pain. | Moderate:  With 21 participants and 23 in-depth interviews, the study provides “vivid, in-depth depictions” of the lived experience. While the quantity of data are sufficient for a phenomenological study, its “richness” is limited by the homogeneity of the sample, which excludes the perspectives of diverse ethnic groups who may experience pain differently. | High: The study is highly applicable to the context of gerontological social work and pain management, as it specifically targets the “grossly lacking” area of qualitative research regarding older adults’ actual experiences with CLBP. It offers direct practice implications for social workers in multidisciplinary pain settings. |
| 53 | Stensland & Sanders, 2018 | Moderate: The study employed a well-defined phenomenological method (van Manen’s Phenomenology of Practice) and explicitly established rigor through audit trails, verbatim quotes, and peer debriefing. However, the authors acknowledge significant limitations, including the use of nonprobability convenience sampling and a complete lack of racial and ethnic diversity in the sample (all participants were Caucasian). | High: The findings are highly coherent, with each subtheme (Corporeality, Temporality, Relationality, and Spatiality) being extensively supported by verbatim participant data. The authors also demonstrate how their findings align with or expand upon existing qualitative and quantitative literature regarding chronic pain and sleep disruption. | High: The study provides rich, “thick description” derived from 21 in-depth interviews, with a median duration of 61 minutes. The quantity of data were sufficient to develop complex thematic structures, and the quality is enhanced by using generous verbatim language to ensure interpretations are rooted in participant experience. | High: This research is directly relevant to the study of chronic low back pain (CLBP) in older adults, as it specifically addresses a noted gap in the literature. It focuses on a population (those 65 and older) that has been underrepresented in previous qualitative reviews of CLBP. |
| 54 | Stensland & Sanders, 2018 | Moderate: The study used an established qualitative framework (van Manen’s phenomenology) and employed rigorous line-by-line thematic coding. However, the authors acknowledge significant limitations, including a lack of racial and ethnic diversity (all participants were Caucasian) and the use of nonprobability convenience sampling, which prevents broad generalizabilit. | High: The findings are strongly supported by the underlying data, with specific subthemes (e.g., “Falling Apart”) supported by up to 19 of the 21 participants. The study also demonstrates consistency with, and expansion upon, the existing literature regarding the social and physical losses associated with chronic pain. | Moderate: The data are exceptionally rich and high-quality, derived from 23 in-depth interviews that lasted up to 109 minutes and were transcribed verbatim. However, the quantity of data are limited by a small sample size of 21 participants from a single Midwestern location, which may not capture the full spectrum of the aging experience with CLBP. | High: The study is directly applicable to the intersection of chronic pain and aging, specifically focusing on how CLBP impacts retirement, housing, health, and independence. It addresses a specific gap in research, as older adults are often neglected in pain studies despite having unique age-related outcomes like fear of nursing home admission. |
| 55 | Bath et al.,  2019 | Moderate: The study used strategies for trustworthiness such as team debriefing, verbatim quotes, and checking themes with a stakeholder group. However, limitations included a disconnect between the research team and the interviewers, meaning analysts could not follow up on specific participant responses. There was also a potential “healthy worker effect,” as the study did not include individuals who had already left farming due to their condition. | High: The findings are strongly supported by the underlying data, with themes directly illustrated by participant quotes. Furthermore, the findings, such as the “pushing through” mentality and barriers to care, are consistent with similar qualitative research conducted on farmers in New Zealand, Ireland, and South Georgia. | Moderate: The study utilized 12 in-depth, face-to-face interviews, which provided enough depth to identify two overarching themes and four sub-themes. However, the interviews were relatively short (only 20-30 minutes), and the sample was heavily gender-skewed (11 men, 1 woman) and localized to within 400km of Saskatoon, which may limit the richness and diversity of the data. | High: The study is highly applicable to the specific context of the review question, as it directly explores the unique environmental and occupational challenges, such as extreme seasonality and geographical isolation, faced by farmers in the Saskatchewan region. The findings are intended to inform tailored health services for rural and remote farming communities. |
| 56 | Cheng et al, 2019 | High: The review utilized a rigorous, structured seven-step meta-ethnography approach and applied the validated Joanna Briggs Institute Qualitative Assessment and Review Instrument (JBI-QARI) to appraise the quality of all 26 included studies  . Two reviewers worked independently to ensure transparency and minimize bias. | High: The findings are strongly supported by underlying data, with a clear mapping of themes to primary studies. Table 4 demonstrates that major findings, such as “problematic symptoms” and “taking medication,” are consistently identified across the vast majority of the 26 studies, showing high internal consistency. | High: The review synthesized data from 26 studies involving 654 participants. This is a substantial sample for qualitative synthesis, spanning ten countries and nearly three decades of research (1990-2017), providing a rich and comprehensive dataset. | High: The included studies are highly relevant to the review's specific focus on adults with multiple chronic conditions (MCCs). The inclusion criteria strictly targeted papers describing lived experiences of coping, ensuring the findings directly address the core research question. |
| 57 | Hughes et al.,  2019 | Moderate: The study followed established guidelines for Interpretative Phenomenological Analysis (IPA) and utilized multiple analysts to cross-check and agree upon themes. However, limitations include a small, self-selected sample from online forums which may represent “better functioning” individuals, and the use of telephone interviews which could have hindered rapport-building and the exploration of photographs. | High: The findings are clearly supported by the underlying data, with superordinate themes emerging directly from participant narratives and photographs. The researchers ensured coherence by grounding their interpretations in repeated references to verbatim transcripts and conducting regular discussions between authors to justify their findings. | Moderate: The data are qualitatively rich, derived from a week-long photo-elicitation period and in-depth interviews lasting up to 90 minutes. While the sample size of six is standard for the idiographic nature of IPA, the authors acknowledge that the data may lack the quantity and breadth to represent the full spectrum of pain experiences in the broader paraplegic population. | High: The study is highly applicable to the specific context of understanding how chronic pain and mobility issues intersect to influence the quality of life (QoL) for individuals with paraplegia. It addresses a recognized gap in existing literature regarding the in-depth, personal meaning-making associated with pain and disability. |
| 58 | Rodríguez et al., 2019 | High: The study employed rigorous qualitative standards, including the use of a 32-item checklist (COREQ) for reporting and a triangulation strategy involving researchers from different disciplines (nursing and computing) to reach intersubjective agreement. Additionally, data analysis followed established grounded theory practices like open coding and reached data saturation by the sixth interview. | High: The findings are clearly supported by the underlying data, with major themes (e.g., impact of pain, social support) illustrated by direct, translated quotes from the participants. Furthermore, the study's results, such as the functional limitations and emotional impacts of spinal deformity, are consistent with previous related research conducted in other regions like Canada and Norway. | Moderate: The data are rich, consisting of 30-60 minute semi-structured interviews that were recorded and transcribed verbatim. The study also used validated questionnaires (ODI, SRS-22r, and WHOQOL-BREF) to create detailed profiles for each participant. However, the sample size is relatively small (10 participants) and was localized to a single university hospital in Santiago. | High: The findings are highly applicable to the review question regarding chronic pain in older adults, as they specifically explore the unique Chilean cultural and societal context. The study provides specific insights into how local factors, such as public transportation infrastructure and Latin intergenerational family dynamics, influence the pain experience. |
| 59 | Schultz et al.,  2019 | Moderate: The study used Interpretative Phenomenological Analysis (IPA), reaching data saturation. The researchers employed respondent validation and discussed codes with local experts to improve reliability. However, the authors acknowledge limitations including a small sample size (n=10), geographic restriction to one region in Denmark, and limited diversity regarding gender and ethnicity. | High: The findings are clearly supported by the underlying data, with four superordinate themes directly derived from the interviews. The paper provides extensive verbatim quotes from participants to illustrate each theme, such as experiences of “fragmentation” and “feeling trapped.” Furthermore, the findings are consistent with and compared to existing literature on chronic pain and stigma. | Moderate: The quantity of data are limited by the small sample size of 10 patients. However, the richness and quality of the data are supported by the length of the face-to-face interviews, which averaged 59 minutes, and the use of strategic sampling to ensure various symptom durations and socio-economic statuses were represented. | High:  The study is highly applicable to its specific context, focusing specifically on the experiences of patients with Chronic Widespread Pain (CWP) within the Danish health and social care sectors. It directly addresses the intersectoral challenges between general practitioners, hospitals, and municipality job centers as defined by Danish health authorities. |
| 60 | Toye et al., 2019 | Moderate: The researchers followed established stages of meta-ethnography to ensure a rigorous interpretive process. However, they explicitly noted that they did not use any formal checklist to appraise the quality of the included syntheses, arguing that such appraisals can be “pettifogging” and that there is no agreed-upon method for determining quality in this context. Additionally, the screening process was conducted by a single researcher without a second-party verification, though the authors provided a detailed justification for this based on the researcher's expertise and the nature of qualitative review. | High: The findings demonstrate strong coherence as they were developed through “constant comparison” and “translation” of concepts across the nine included syntheses. To maintain grounding in the data, researchers challenged each other’s interpretations, and any disagreements were resolved through discussion with a third reviewer. The resulting 10 conceptual categories and the final model were organized collaboratively to ensure they reflected the essence of the underlying data. | High:  The paper draws on a substantial and rich dataset, synthesizing 9 qualitative evidence syntheses (QES) which collectively incorporate 128 published reports and data from more than 2,000 individuals living with rheumatoid arthritis. From these, the authors extracted 76 distinct review findings to build their conceptual model. | High:  The studies supporting the findings are highly applicable, as the search was specifically designed to identify syntheses exploring the lived experience of rheumatoid arthritis (RA). The included QES cover a broad geographic range (e.g., UK, USA, Netherlands, Scandinavia, and Asia), providing a comprehensive view of the patient experience across different healthcare contexts. |
| 61 | Mustafa et al.,  2020 | High: The study utilized a well-defined interpretive phenomenological approach based on van Manen’s phenomenology of practice. Researchers used purposeful and snowball sampling to reach a specific, hard-to-access population and conducted interviews in the participants' preferred languages (English, Hindi, or Urdu) to ensure accuracy. While the authors noted they could not restrict the sample to a single religious or regional Indian group, this diversity was acknowledged as a factor for future study rather than a flaw in the current design. | High: The findings are strongly supported by the underlying data, with four clear themes (body, space, relationships, and time) emerging directly from participant narratives. The results are consistent with existing literature regarding the impact of gender roles and the “healthy immigrant effect” on health outcomes. The link between the “dual gender role” (balancing housework and outside employment) and exacerbated pain is clearly demonstrated through the participants' own accounts. | Moderate: The study provided rich, “thick descriptions” through hour-long, one-on-one interviews with 13 women. While a sample of 13 is appropriate for a phenomenological study (which suggests 6 to 15 participants), the absolute quantity of data are small compared to larger mixed-methods or quantitative reviews. The richness of the qualitative data are high, but the small sample size limits the ability to generalize across all Indian subcultures. | High: The findings are directly applicable to the specific context of providing culturally sensitive care for chronic pain in Canada. The study addresses a significant public health gap, as Indian-Canadians are a rapidly growing population who report higher pain intensity than non-immigrants. It provides specific insights into how cultural expectations and immigration challenges (such as the loss of domestic help) specifically influence pain experiences in this community. |
| 62 | Roenne et al.,  2020 | Moderate: The study used a robust qualitative design, following Ricoeur’s philosophy of interpretation and sought to meet COREQ reporting recommendations  . It addressed researcher reflexivity and used triangulation to enhance trustworthiness  . However, it relied on a small convenience and purposive sample of 10 patients from a single country (Denmark), and the authors acknowledge potential selection bias and the lack of criteria regarding the duration of the patients’ treatment trajectory. | High: The findings are clearly and logically derived from the underlying data through a three-level interpretative process. The study provides a detailed mapping in Table 3, which explicitly links raw participant quotes (meaningful units) to specific sub-themes and key themes, demonstrating a consistent and transparent relationship between the data and the findings. | High: Although the sample size is small (N=10), the authors reached data saturation after eight interviews and conducted two additional interviews to ensure “high information power.” The data collected was “rich and in-depth,” with interviews lasting an average of 52 minutes, providing sufficient substance to explore sensitive personal and family dynamics. | High: The study is directly and specifically applicable to the context of chronic non-malignant pain (CNP) rehabilitation. It explores the primary experiences, needs, and preferences of patients regarding the involvement of relatives, providing insights that are highly relevant to clinical practice in multidisciplinary pain centers. |
| 63 | Skúladóttir et al.,  2020 | High: The study employed a rigorous 12-step phenomenological approach (the Vancouver School) designed specifically for exploring lived experiences  . It utilized member checking for verification and strictly adhered to the COREQ 32-item checklist to ensure reporting quality  . While one participant was lost to follow-up, the methodology remained robust for qualitative inquiry. | High: Findings are strongly supported by direct participant quotes that illustrate a clear transition from “survival mode” to “reconstruction.” The themes identified remained consistent across participants from three different rehabilitation sites. Furthermore, the results are highly consistent with findings from similar international studies. | Moderate: The data are rich and longitudinal, comprising 21 interviews conducted before and after the intervention. However, the sample size is small (n=11), and the authors acknowledge that the 3-month follow-up period may have been too short to fully capture the long-term “reconstruction” phase of the participants' lives. | High:  The study is directly applicable to understanding the lived experience of chronic pain and the impact of multidisciplinary rehabilitation. By including participants from all three major rehabilitation centers in Iceland with various diagnoses, the findings are highly relevant to the specific context of pain management programs. |
| 64 | Yildirim Duman  2020 | Moderate: The study employed a descriptive phenomenological approach and adhered to the Consolidated Criteria for Reporting Qualitative Research (COREQ) guidelines to ensure quality. Reliability was enhanced through expert triangulation and participant feedback (member checking). However, the sample size is very small (n=10), the research was conducted in a single geographic province, and the author acknowledges that personal presuppositions may have influenced the inductive data analysis. Additionally, there is a discrepancy in the reported interview duration (1440 minutes is noted as 60 hours, whereas it is actually 24 hours). | High: The findings are strongly supported by the underlying data, with clear links between participant quotes and the identified themes of social support, disease management, and information sources. The subthemes, such as “perceived barriers” and “emotional and spiritual state,” provide a consistent narrative that directly addresses the study's aim to understand lived experiences. | Moderate: While the researcher notes that data saturation was reached, the study relies on a limited pool of 10 participants. The data provided is rich, with individual interviews lasting 45 to 60 minutes and yielding 12-14 pages of transcript per person, but the overall quantity of data are constrained by the small sample size. | High: The study is highly applicable to the context of chronic disease self-management, focusing on prevalent conditions like hypertension and diabetes. The results offer specific insights into the role of family support and religious practices, which are vital for healthcare professionals designing patient-centered care programs. |
| 65 | Andersen et al., 2021 | Low: The study followed standard Interpretative Phenomenological Analysis (IPA) procedures, including individual transcript analysis and researcher reflexivity. While the authors noted that applying a specific theoretical framework (Kierkegaard) could potentially restrict findings, they mitigated this by ensuring initial inductive themes were developed before the theory was introduced. | High: The identified findings are strongly supported by verbatim data from the interviews. The themes, such as “despair” and “becoming oneself,” are logically derived from the participant accounts and are consistent with the underlying data regarding how patients respond to their illnesses. | High: The study is grounded in 23 semi structured interviews, which is a substantial and high-quality sample for an IPA study. The participants had a diverse range of ages, educational backgrounds, and illness durations, ensuring richness of the dataset. | High: The findings are highly applicable to the specific context of existential challenges in chronic illness. The study directly addresses how patients with multiple sclerosis and chronic pain navigate identity and meaning, providing insights relevant to clinical health care practices. |
| 66 | Fracso et al.,  2021 | Low: The study followed the COREQ guidelines for reporting qualitative research. It employed investigator triangulation (two experts and one trained investigator) and reached theoretical saturation. While the authors acknowledged potential selection and social desirability biases, they utilized reflexive practices and pilot test sessions to ensure authenticity. | High:  The findings (four phenomenological categories) are logically derived from the transcripts using a rigorous semio-pragmatic analysis based on Peirce's theory. Each theme is extensively supported by verbatim participant quotes, ensuring the results remain grounded in the underlying data. | High: The researchers conducted 37 in-depth interviews with 20 participants, utilizing a longitudinal design (interviews before and 6 months after the intervention). The sample included a diverse range of demographics, including different ages, socioeconomic backgrounds, and chronic conditions. | High:  The study is directly applicable to the review's context, as it specifically examines the lived experiences and existential needs of vulnerable patients participating in a chronic disease self-management program  . It offers specific insights into how these programs foster empowerment in this population. |
| 67 | Honoré Grauslund et al.,  2021 | Moderate: The researchers noted significant challenges regarding participants limited Danish language proficiency and the absence of professional interpreters, which may have restricted the depth of the qualitative data. However, investigator triangulation (using researchers from different professional backgrounds) was employed to enhance the study's trustworthiness. | High: The findings, specifically the three identified “essences,” are strongly supported by verbatim participant quotes, and are logically consistent with existing concepts like fear-avoidance behavior and social isolation. | Moderate:  While 11 participants provided a sufficient sample for a phenomenological study, the authors acknowledged a lack of rhetorical depth in descriptions due to language barriers. Furthermore, only five of the eleven participants were quoted in the results section, potentially limiting the representativeness of the findings. | High:  The study is directly applicable to understanding the lived experience of a specific minority group (Turkish immigrants) navigating chronic pain within a Western healthcare system. It offers specific insights into migration-related challenges and cultural nuances in rehabilitation. |
| 68 | Keen et al., 2021 | Low: The study employed rigorous qualitative methods, including the Grid Elaboration Method (GEM) and Thematic Analysis (TA). Trustworthiness was established through a reliability test (blind-coding of transcripts) and a detailed reflexive statement acknowledging potential researcher bias. Ethical approval was also documented. | High: The findings regarding the inconsistent and experiential nature of pain models are strongly supported by verbatim participant quotes. The researchers used Social Representations Theory as a clear epistemological lens to explain how participants anchored and objectified their pain experiences. | Moderate: While the data provided rich insights, the authors noted that the sample size of 12 was “sub-optimal for robust TA.” Additionally, the remote interviewing format made it difficult to apply the GEM tool exactly as intended (limiting participants to one word/image per box). | High: The paper is directly relevant to clinical and research questions regarding pain management. It specifically explores how patients' prior conceptualizations of pain interact with Pain Neurophysiology Education (PNE), providing critical insights into why such interventions may lack practical utility for some individuals. |
| 69 | Niño de Guzmán Quispe et al. 2021 | Low: The study followed a rigorous, systematic process based on the Arksey and O’Malley framework and the PRISMA-ScR checklist. The researchers utilized investigator triangulation for selection and extraction and employed a pilot-tested form to ensure consistency. However, limitations include the exclusion of non-English studies and the inherent risk of missing nuances by focusing only on reviews rather than primary data. | High:  The 12 main themes identified were consistently found across the four different chronic diseases and various stakeholder perspectives. Furthermore, the findings are logically supported by and aligned with established health psychology frameworks, such as the Common Sense Model of Self-Regulation and Self-Determination Theory. | High: The review is exceptionally comprehensive, synthesizing data from 148 reviews. This large sample size provided a vast quantity of data covering Type 2 Diabetes, obesity, COPD, and heart failure, ensuring high richness and breadth in the findings. | High: The study is directly applicable to the review's objective of understanding patient-centered outcomes in chronic disease. The findings were specifically designed to inform the development of Core Outcome Sets (COS) and clinical recommendations for self-management interventions. |
| 70 | Stensland  2021 | Low: The study followed Van Manen’s Phenomenology of Practice method, utilizing investigator triangulation and member checking (co-interpretation) to ensure rigor. Although the author noted limitations such as a lack of racial diversity and the use of convenience sampling, the study maintained high qualitative standards for transparency and reflexivity. | High: The findings are consistently and strongly supported by verbatim participant quotes, thus providing a clear audit trail from the data to the final themes. The sub-themes, such as the “apprehension of medication,” are logically derived from the participants' detailed accounts of their management struggles. | High: The researcher conducted 21 in-depth interviews (with two participants interviewed twice) with a median length of 61 minutes, yielding a rich and substantial dataset. The data were deemed sufficient to capture the “essential structure” of the lived experience of managing incurable pain. | High: This study is highly relevant as it addresses a “critical gap in the literature” by focusing specifically on the experiences of older adults with treatment-resistant chronic low back pain. It provides direct and actionable insights for gerontological social work practice and interdisciplinary healthcare. |
| 71 | Stensland  2021  (b) | Low: The study utilized Van Manen’s Phenomenology of Practice method and adhered to Lincoln and Guba’s framework for qualitative rigor. The researcher employed investigator triangulation and member checking (co-interpretation with participants) to ensure the findings accurately reflected their lived experiences. While the author noted limitations regarding convenience sampling and a lack of racial diversity, these were transparently discussed as part of the study's design. | High: The findings are consistently and strongly supported by verbatim participant quotes, providing a clear link between the raw data and the final themes. The development of themes, such as the battle between helplessness and perseverance, followed a logical, iterative process of interpretation and refinement. | High: The study involved 23 in-depth interviews with 21 participants, with a median interview length of 61 minutes. This provided a rich and substantial dataset that allowed for a deep, "vivid illustration" of the existential and psychological challenges faced by the participants. | High: The paper is highly applicable to the specific context of geriatric healthcare and chronic pain management. It addresses a historical underrepresentation of older adults in pain research and provides direct insights into the psychological features of chronic low back pain, such as helplessness and suicidality, which are critical for clinical practice. |
| 72 | Toye et al., 2021 | Low: The study utilized a rigorous seven-stage meta-ethnography approach and adhered to the eMERGe reporting guidelines. Rigor was maintained through investigator triangulation (three reviewers for analysis) and a formal appraisal process where two reviewers excluded “fatally flawed” primary studies. While the search was limited to a single database (MEDLINE), the authors justified this choice based on its known high yield for qualitative research. | High: The findings are logically integrated into a conceptual model that describes the "healing journey" through an iterative process of constant comparison and abstraction. The results are highly coherent as they specifically build upon and refine an established “mega-ethnography” used as an index paper, ensuring the new findings are grounded in existing literature. | High: The synthesis is exceptionally comprehensive, drawing from 195 primary reports representing at least 3,589 individuals with chronic pain. The researchers collected data until theoretical saturation was achieved, and the sample provided significant breadth, covering diverse chronic conditions and geographic areas. | High: The paper is directly relevant to the understanding of patient recovery, offering a new conceptualization of healing as an ongoing journey rather than a destination. It provides actionable insights for clinical health interventions, such as the importance of validating pain through meaningful explanations and facilitating social reconnection. |
| 73 | Chala et al.,  2022 | Low: The study followed a rigorous Interpretative Phenomenological Analysis (IPA) approach and adhered to the COREQ checklist for reporting qualitative research. The researchers utilized investigator triangulation (independent coding by two members) and reflexivity to ensure the findings were grounded in the participants’ accounts. While limited to a single hospital setting, these limitations were transparently acknowledged. | High: The five main themes are strongly supported by verbatim participant quotes, thus providing a clear audit trail from the raw data to the final interpretations. The findings are logically consistent with established psychosocial concepts, such as the “invisibility of pain” and its impact on self-identity, while also accounting for specific Ethiopian cultural nuances. | High: The study included 15 participants, which is considered a robust and appropriate sample size for the in-depth, idiographic nature of IPA. The data collection involved semi-structured interviews lasting up to 71 minutes, and the sample represented a diverse demographic range in terms of age, gender, and education levels. | High: This paper is highly relevant as it addresses a significant research gap concerning the lived experience of chronic pain in non-Western, developing countries. It provides specific, actionable insights into how collectivist cultural values and religious beliefs (e.g., holy water) influence pain management in Ethiopia. |
| 74 | Kahere et al,  2022 | Low: The study employed a rigorous descriptive phenomenological design and adhered to the COREQ reporting guidelines. Trustworthiness was enhanced through investigator triangulation (independent coding by two authors using different methods) and member checking, where transcripts were returned to participants for correction. While the authors noted potential recall bias due to self-reporting, the overall design and conduct were robust. | High: The findings are consistently and strongly supported by verbatim participant quotes throughout the results section. The 13 identified subthemes are logically derived from the data and are congruent with existing literature from high-income countries and other disease contexts in South Africa. | High: The researchers conducted 15 in-depth interviews, which exceeded the point of code saturation (identified at 9-10 interviews). Data were collected across five different provincial hospitals to ensure a diverse range of demographic and socioeconomic profiles, providing a rich and “knowledge-rich” dataset. | High: This is the first study in South Africa to explore barriers to healthcare access specifically for chronic low back pain. It is highly relevant to the clinical and policy context of low- and middle-income countries (LMICs), offering specific insights into travel burdens, paper-based referral failures, and cultural beliefs that impact patient care. |
| 75 | Altun et al.,  2023 | Moderate: The study employed a standard phenomenological approach and emphasized researcher reflexivity. However, the authors acknowledged several limitations, including a small sample size, potential social desirability bias, and the use of family members as interpreters, which may have influenced the depth or authenticity of the participants' disclosures. | High: The five major themes identified are strongly and consistently supported by verbatim participant quotes. Furthermore, the findings are logically aligned with Levesque’s Patient-centered Access to Health care framework. | Moderate: While the interviews were in-depth (lasting 45-90 minutes) and the researchers reported reaching data saturation, the sample size was small (n=10). Additionally, the authors noted that conversations were sometimes fragmented owing to the use of interpreters, which may have limited the richness of the data. | High: The paper is directly applicable to the context of health equity and chronic pain management for marginalized populations. It provides specific, unique insights into how cultural values, gender roles, and refugee status intersect to shape healthcare access for Assyrian women. |
| 76 | Chimezie, 2023 | Moderate: The paper is a narrative literature review rather than a systematic review or primary empirical study. While it synthesizes a vast array of existing research and reports, it does not explicitly detail a systematic search strategy or formal quality appraisal of the included literature, which increases the risk of selection bias. | High: The findings are logically structured and consistently supported by the cited data. The author presents a clear and cohesive argument linking low health literacy to poor chronic disease outcomes, cultural barriers, and systemic healthcare failures. | High: The review draws upon an extensive and diverse evidence base, including global health statistics from the WHO and CDC, as well as specific regional studies from Nigeria, Ghana, and Ethiopia. The inclusion of 74 references further provides a rich and comprehensive foundation for the paper's claims. | High: This paper is highly relevant to the fields of public health and chronic disease management. It specifically addresses the “double burden of disease” in African countries, offering critical insights into how health literacy can be used as a tool to improve early detection and access to care in developing health systems. |
| 77 | Joyce et al., 2023 | Moderate: The study utilized an interpretive description design and followed SRQR reporting guidelines. However, the authors acknowledged a significant response bias, noting that participants willing to be interviewed were more likely to have had a positive experience. Additionally, the use of a convenience sample of only 12 out of 102 potential participants was cited as a limitation. | High: The findings are logically and theoretically coherent, particularly the use of Bandura’s self-efficacy model to deductively code and explain the theme of empowerment. Themes are consistently supported by verbatim participant quotes, and the qualitative findings were corroborated by descriptive quantitative data from the parent RCT. | Moderate: While the researchers conducted in-depth, verbatim-transcribed interviews (30-60 minutes) and utilized investigator triangulation, they admitted that the sample size of 12 was a “low representation” of the overall trial population. This limited size may not have captured the full breadth of experiences, especially those of participants who did not improve. | High:  This paper is highly relevant as it specifically addresses a “paucity of research” regarding how underserved, low-income, and minority populations experience physical therapy. It provides critical, actionable insights into overcoming systemic distrust and health disparities in chronic pain management. |
| 78 | Kjærulff et al., 2023 | High: The authors explicitly state that most included studies (13/17, 76%) were of low quality. Quantitative studies lacked representativeness and failed to adjust for confounders, while qualitative studies often lacked methodological transparency and rigor in their analytical steps. | Moderate: While the narrative synthesis logically divides findings into 3 main categories and 7 subcategories represented in a conceptual model, the authors note that too few studies had sufficient transparency, calling into question the overall validity and generalizability of these findings. | Low: The review concludes that the current state of research is “relatively immature,” and that “more evidence is needed to draw conclusions.” The small percentage of screened studies that met the inclusion criteria (0.55%) further highlights the lack of robust, high-quality primary data in this field. | High: The paper is directly applicable to the specific context of patient-provider relationships in the digital age. It specifically examines how peer-to-peer interactions on social media influence communication, medical advice interpretation, and power dynamics for individuals managing chronic conditions like diabetes. |
| 79 | Peppard et al.,  2023 | Low: The study followed a rigorous qualitative design using van Manen’s phenomenological approach and a six-step methodological framework for thematic analysis. Trustworthiness was supported by verbatim transcription verification, the use of NVivo software for data management, and researcher reflexivity, as the lead author was a female Navy nurse. Minor limitations like internet instability during Zoom interviews were transparently acknowledged. | High: The findings are strongly and consistently supported by verbatim participant quotes for each of the eight identified themes. There is a logical and clear connection between the raw interview data and the final interpretations, and the results are noted to be consistent with prior research on musculoskeletal conditions and military sexual trauma (MST). | High: The researcher conducted 13 in-depth interviews and explicitly stated that data saturation was achieved. The sample was highly diverse, representing all major military branches (Army, Navy, Air Force, Marine Corps), various ranks (enlisted and officer), and a wide range of racial and ethnic backgrounds. | High: The study is exceptionally relevant to the context of military medicine and women's health, specifically addressing a documented “paucity of research” regarding the daily lived experiences of military women. The findings provide direct insights into Force Health Protection, clinical nursing practice, and gender-specific healthcare needs. |
| 80 | Sun et al. 2023 | Moderate: The review followed rigorous protocols, including the PRISMA recommendations and the CASP qualitative appraisal tool. However, the authors noted that 15 of the 17 included studies were of moderate quality (only two were high), and the exclusion of non-English literature introduced a potential language bias. | High: The findings are consistently supported across multiple perspectives (patients, caregivers, and providers) and are mapped to three clear, logically derived themes: resilience, interpersonal connections, and the care supply chain. The results also resonate with broader international literature regarding the gains and losses of care transitions. | Moderate: The review synthesized 17 studies from nine different countries, providing a broad geographical and cultural range. However, the authors identified a “weakness in the existing literature” regarding the perspectives of healthcare providers and transition nurses, as only three of the included studies explored these specific viewpoints. | High: This paper is highly relevant to the context of geriatric chronic disease management. It specifically addresses the “triadic process” of transition involving patients, families, and professionals, and focuses on the high-risk period of hospital-to-home discharge for an aging population with multimorbidity. |
| 81 | van Rysewyk et al., 2023 | Low: The study employed a rigorous mega-ethnography approach, following the established seven stages of meta-ethnography. It featured a comprehensive search of six databases and maintained high transparency through multidisciplinary steering committee oversight and Patient and Public Involvement (PPIE) throughout the design and analysis. While no official reporting guidelines exist for this specific review type, the authors followed systematic protocols and addressed potential language bias. | High: The eight identified themes were consistently supported across the 20 included Qualitative Evidence Syntheses (QES). These findings were further validated by a survey of 1,219 people, which showed strong or very strong agreement with the themes. Additionally, the results are highly consistent with previous meta-ethnographies in the field. | High: The review synthesized 20 high-level QES, which collectively represent more than 300 primary qualitative studies. This vast amount of qualitative data were supplemented by a large-scale survey, thereby ensuring the findings are grounded in both a high quantity and a significant richness of evidence regarding the lived experience of pain. | High: The study is directly relevant to the clinical and social understanding of chronic non-cancer pain (CNCP). It specifically addresses the “blind spot” in pain management by focusing on the meaning and impact of pain on everyday life, providing a validated summary that is highly applicable to health policy and practice. |
| 82 | Albán-Reyes et al.,  2024 | Moderate: The study followed a robust phenomenological design and adhered to the COREQ 32-item checklist for qualitative research. It utilized expert-validated interview guides and a standardized questionnaire (CAD) with high internal consistency. However, the researchers noted that the CAD was not specifically validated for ballet dancers in Colombia, and the small sample size (n=17) from a single company may limit the generalizability of the findings. | High: The findings are strongly supported by the underlying data, with a clear link between the participants' interview responses and the quantitative results of the CAD. Furthermore, the study's findings regarding the prevalence and types of injuries are consistent with existing international literature on ballet professionals. | High: The researchers achieved data saturation within the targeted population. The data collected is rich and detailed, derived from in-depth interviews averaging 59 minutes each, as well as a 31-item questionnaire that allowed for a comprehensive characterization of coping behaviors. | High: The study is directly applicable to the context of managing chronic pain in the field of professional dance. It specifically addresses the biopsychosocial factors and professional pressures unique to ballet dancers, such as the “ideal of perfection” and the “fear of being expelled,” making the findings highly relevant to the review question. |
| 83 | Bruger et al.,  2024 | High: The study employed Interpretative Phenomenological Analysis (IPA), which is highly suited for exploring lived experiences. The researchers followed Yardley’s guidelines for methodological rigor, utilized a reflective diary to address personal reflexivity, and conducted team triangulation to prevent analytic bias. Additionally, materials were reviewed by a Patient and Public Involvement (PPI) volunteer with a brain injury to ensure appropriateness. | High: The findings are strongly supported by rich, idiographic data, with themes directly grounded in extensive participant quotes  . Furthermore, the study’s results regarding the “solitude” and “invisibility” of pain are highly consistent with existing international literature, such as studies from Sweden on post-stroke pain experiences. | Moderate: The study provides rich, detailed, and high-quality data from semi-structured interviews averaging 55 minutes each. However, the sample size is small (n=8), which, while standard for the idiographic depth required by IPA, represents a limited quantity of data compared to other qualitative approaches. | High: The study specifically targets the unique socioeconomic and psychosocial context of working-age adults (aged 18-64) in the UK. It addresses a significant research gap, as this population faces different life-stage challenges compared to older stroke survivors, making the findings highly relevant to understanding the specific burden of chronic post-stroke pain. |
| 84 | Bullo et al.,  2024 | High: The study utilized a rigorous multimodal research design, combining Interpretative Phenomenological Analysis (IPA) with conceptual metaphor and metonymy analysis. The authors addressed trustworthiness through specific strategies for credibility, transferability, and confirmability, including team triangulation and building rapport to ensure participant honesty. | High: The findings are strongly supported by the primary data, with the overarching theme of “Pain as Physical Violence” emerging consistently from both the visual artefacts and the accompanying verbal transcripts  . Furthermore, the results align with existing international literature regarding the metaphorical description of endometriosis pain. | Moderate: The data collected are rich and high-quality, capturing multiple sensory registers through 90-minute focus groups and 26 unique participant-generated artefacts. However, the sample size is small (n=10), which, while appropriate for the idiographic depth of IPA, represents a limited quantity of data for broader generalization. | High: The study is highly relevant to the context of chronic pain management, as it specifically addresses the "invisible" and elusive nature of endometriosis pain. The findings offer direct clinical implications for improving patient-professional communication and reducing diagnosis delays through visual cues. |
| 85 | Gaber et al., 2024 | High: The researchers followed a rigorous meta-ethnography process based on the eMERGe guidelines and Noblit and Hare (1988). The study was registered with PROSPERO, utilized independent screening by two authors with a third for consensus, and applied the CASP checklist for quality appraisal of included studies. | High: The findings are organized into a clear conceptual model with five identified themes that emerged from a synthesis of diverse qualitative studies. This framework provides consistent recommendations across different types of participatory research. | Moderate: The synthesis includes data from approximately 153 residents and 99 staff across seven countries. However, it is limited by a small sample of only 10 included articles and insufficient reporting of primary participants' age, gender, or ethnicity in the source studies. | High: The study is highly applicable to the specific context of long-term care facilities. It directly addresses an under-explored area, participatory research approaches in nursing homes and residential care, to provide specific recommendations for improving the quality of life and care for older adults. |
| 86 | Lilja et al.,  2024 | High: The study utilized a rigorous phenomenological hermeneutic approach and adhered to the Standards for Reporting Qualitative Research (SRQR). Trustworthiness was strengthened through team triangulation, the inclusion of a patient representative in the analysis, and the documentation of the lead researcher's preunderstandings to address reflexivity. | High: The study establishes a clear and logical link between the raw interview narratives, the condensed meaning units, and the final metaphorical interpretation of “MVP vs. passing the ball.” The findings are also consistent with existing international literature regarding the desire of patients to be recognized as individuals rather than just their condition. | Moderate: The data are exceptionally rich and high-quality, with in-depth interviews averaging 77 minutes each. However, the sample size is small (n=10) and lacks demographic diversity, as most participants were female, born in Sweden, and had post-high school education, which may limit the breadth of the findings. | High: The study is highly relevant to the context of chronic pain management, as it specifically elucidates the meaning of support from both social networks and the healthcare sector. The findings provide direct insights for person-centered care and clinical practice. |
| 87 | Lo Monaco et al., 2024 | High: The study followed a rigorous phenomenological approach using Colaizzi's method for analysis. Rigor was ensured through Lincoln and Guba's criteria, adherence to COREQ guidelines, independent coding by two researchers, and member checking to confirm that participants agreed with the findings. The researcher also addressed reflexivity by documenting personal preconceptions in a notebook. | High: The five identified themes are strongly supported by rich, verbatim participant quotes provided throughout the results section. The findings, such as the association between trauma and FMS onset and the prevalence of sexual dysfunction, are consistent with existing international literature and established clinical observations. | Moderate: The data are detailed and high-quality, derived from face-to-face interviews lasting between 40 and 90 minutes. While the researchers achieved data saturation, the sample size is small (n=10) and limited to a specific geographic location (Palermo, Italy), which may limit the breadth of perspectives compared to larger-scale studies. | High: The study is highly relevant to the context of chronic pain and family life, as it specifically explores the under-studied lived experience of mothers with fibromyalgia. It provides direct insights into the impact of pain on maternal roles and sexual health, which are critical for developing comprehensive, interprofessional care programs. |
| 88 | Lundin et al., 2024 | High: The study employed a rigorous phenomenological hermeneutical approach and strictly adhered to the Standards for Reporting Qualitative Research (SRQR) to ensure trustworthiness. The researchers utilized team triangulation throughout the analysis and explicitly addressed reflexivity by documenting and discussing pre-understandings. | High: The findings are strongly supported by the underlying data, with a clear link between the overarching theme, “Have my back as I get back to work,” and the detailed verbatim quotes provided. Furthermore, the study’s results regarding the role of managers and the impact of stigma are highly consistent with existing international literature. | Moderate: The data are exceptionally rich and high-quality, derived from in-depth interviews averaging 41-90 minutes and resulting in over 230 pages of transcript. However, the sample size is small (n=14) and lacks demographic diversity, as it was predominantly female (12/14) and limited to Swedish speakers. | High: The study is directly applicable to the review question, as it specifically explores the meaning of support from various stakeholders (employers, healthcare, and social insurance) during the return-to-work process for individuals with chronic pain. |
| 89 | Macgregor et al., 2024 | High:  The study followed a rigorous meta-ethnography process adhering to eMERGe reporting guidance. It was registered with PROSPERO, utilized blinded reviewers for screening, and included quality appraisal of all primary studies. Rigor was further enhanced through team triangulation and the active involvement of co-researchers with lived experience of chronic pain. | High: The findings are synthesized into clear “lines of argument” and a conceptual framework (the “ecosystem”) that logically connects the primary data. The authors explicitly noted that their findings are consistent with previous major meta-ethnographies in the field of chronic pain. | High: The synthesis included 10 qualitative studies featuring a total of 212 participants across seven countries. The authors purposefully included grey literature (theses) and translated non-English data to ensure a broad, rich, and diverse data set that challenged dominant narratives. | High: The study is highly relevant to the review question as it specifically conceptualizes the lived experience of “acceptance” in chronic pain. It broadens the understanding of acceptance from a narrow psychological trait to a complex biopsychosocial ecosystem, offering direct implications for pain care and health equity. |
| 90 | Matias-Soto et al., 2024 | Moderate: Cross-sectional purposive sample from a single pain clinic (patients undergoing epidural infiltrations), potential selection bias (7/29 no-shows), limited transferability beyond this clinical subgroup; but COREQ reporting, IPA approach, and patient involvement strengthen credibility (Methods/Study design, Results). | High: Clear analytic approach (interpretative phenomenology, mixed coding), well-structured themes/subthemes, patient contributed to interview script and checked coding, and findings are logically consistent with reported data (Study design, Results, Discussion). | High: Twenty-two in-depth interviews (mean 43 min) provide substantial, varied experiential data across ages 32–77 and both sexes; data depth and range support identified emotions, cognitions, behaviors (Results). | Moderate–High: Directly applicable to patients with chronic low back pain treated in pain units (especially those receiving invasive interventions); relevance to broader CLBP populations may be reduced in cases where care pathways differ (Introduction, Results, Discussion).  Overall CERQual confidence: Moderate–High—credible, coherent, and well-supported findings with strong patient involvement and transparent reporting, but confidence tempered by single-center sampling and potential selection bias. |
| 91 | Nikoloudi et al., 2024 | Moderate: The researchers employed a rigorous five-step Interpretative Phenomenological Analysis (IPA) and utilized triangulation involving three researchers to ensure the credibility of findings. However, the authors acknowledge significant limitations, including a very small sample size (n=6) from a single facility and the potential for recall bias, as participants were reflecting on experiences from many decades ago. | High: The findings are strongly supported by the underlying data, with the four superordinate themes clearly grounded in extensive verbatim participant narratives  . Furthermore, the results regarding the "biographical disruption" and devalued identity caused by the disease are consistent with existing international literature on leprosy and chronic illness. | Moderate: The study provides exceptionally rich, high-quality, and idiographic data that captures the unique lived experiences of the “last survivors” of leprosy in Greece. While appropriate for the depth of IPA, the quantity of data are limited due to the very small sample size, which the authors admit may not represent the diverse experiences of all relocated individuals. | High: The study is highly relevant to the context of chronic illness and public health, as it specifically explores the long-term psychosocial impact of medical confinement and social exclusion. The findings offer direct insights into the importance of empathetic and holistic patient care for historically stigmatized conditions. |
| 92 | Okolo et al., 2024 | Moderate: The paper is a narrative review that synthesizes a wide range of literature. While it provides a comprehensive overview, the provided text does not describe a formal systematic search strategy, specific inclusion/exclusion criteria, or a standardized quality appraisal tool for the studies it reviews. | High: The findings are highly coherent, with the review logically connecting cultural beliefs, social norms, and biological factors to the subjective experience of pain. The conclusions regarding cultural variability in expression and management are consistently supported by the cited cross-cultural evidence. | High: The review draws from a rich and extensive dataset, citing 50 different sources to explore pain perception from multiple angles, including genetics, neurobiology, and socio-cultural determinants. It covers diverse populations and global perspectives to provide a holistic view of the topic. | High: The study is directly applicable to the field of healthcare and pain management. It specifically addresses how cultural nuances impact diagnostic accuracy, treatment outcomes, and health equity, making it highly relevant for clinical practice and policy development. |
| 93 | Sanabria-Mazo et al.,  2024 | High: The study followed a rigorous qualitative design adhering to COREQ and JARS reporting guidelines. Trustworthiness was established through Lincoln and Guba's criteria, and the analysis involved six independent coders to reinforce rigor. While the authors acknowledged limitations such as potential social desirability and the lack of participant validation of the final analysis, these were addressed through extensive reflexivity measures. | High: The findings are strongly supported by the primary data, with themes clearly grounded in diverse verbatim participant quotes. The results are also consistent with the quantitative findings of the parent randomized controlled trial and align with broader international literature regarding the psychological benefits of group-based ACT and BATD. | High: The researchers utilized a large sample size for qualitative research, involving 55 participants across 12 focus groups. The data are exceptionally rich and detailed, derived from interactive sessions lasting 60 to 90 minutes each, which allowed for a comprehensive exploration of both therapeutic and technological experiences. | High: The study is highly relevant to the context of modern pain management, as it specifically addresses the use of remote synchronous videoconferencing for patients with comorbid chronic low back pain and depression. Its findings provide direct, actionable insights into the facilitators and barriers of eHealth implementations within public health systems. |
| 94 | Schwarz B et al.2024 | High: The researchers employed a rigorous systematic approach, searching four major databases and using two independent reviewers for study screening, data extraction, and quality assessment. They further enhanced the methodology by developing and applying a custom quality appraisal tool based on established standards to evaluate the 19 included meta-syntheses. | High: The findings are clearly supported by a transparent three-step meta-ethnographic synthesis involving systematic comparison and reciprocal translation of concepts. The resulting generic return-to-work (RTW) principles and model are consistently derived from the cross-cutting themes identified across the diverse set of included studies. | High: The evidence base is substantial, consisting of 19 qualitative meta-syntheses that encompass a wide variety of chronic conditions, including musculoskeletal disorders, cancer, brain injuries, and mental disorders. This provides a rich and diverse dataset that allowed for the development of a robust, generic conceptual model. | High: The included meta-syntheses are directly applicable to the review question, as they focus specifically on the RTW experiences and perspectives of individuals with chronic conditions and their stakeholders. While the authors note a geographic bias toward Western countries, the data remains highly relevant for identifying generic mechanisms and facilitators of the RTW process. |
| 95 | Snell et al., 2024 | High: The study followed a rigorous Interpretative Phenomenological Analysis (IPA) framework  . Data were collected through semi-structured interviews designed to build trust and elicit rich accounts, while the analysis involved regular co-author discussions to ensure the appropriateness and validity of the themes. | High: There is a clear and consistent link between the raw data and the findings, supported by verbatim extracts from participant transcripts. The six emergent themes are well-supported by this data and align with existing literature regarding the impact of masculinity on chronic illness. | Moderate: While the study provides rich, idiographic depth typical of IPA, the sample size is limited to five participants. The authors acknowledge that the data are suggestive rather than conclusive because the sample lacks diversity, being predominantly composed of white, heterosexual, partnered men. | High: The study is directly relevant to its objective of understanding the unique male experience of living with CFS. It provides specific insights into help-seeking behaviors, the diagnostic journey, and support networks, which are highly applicable for practitioners and employers. |
| 96 | Barber et al., 2025 | High: The study utilized a rigorous Reflexive Thematic Analysis (RTA) framework, involving six distinct phases of data familiarization, coding, and theme development. Rigor was further supported by researcher reflexivity, coding comparisons between multiple researchers, and the use of standardized iRest research scripts. | High: The generated themes are directly and transparently supported by raw data, including verbatim participant quotes that illustrate the shift from feeling “powerless” to “empowered.” The findings regarding relaxation and pain self-efficacy are consistent with existing literature on Yoga Nidra and autonomic regulation. | Moderate: While the study provides rich, firsthand qualitative accounts from 13 participants, the sample size is small and lacks diversity, consisting predominantly of older, Anglo-Saxon women. Additionally, the researchers were unable to interview those who dropped out, potentially missing divergent or negative experiences. | High: The findings are highly applicable to the clinical context of multidisciplinary pain management. The study specifically addresses the lived experience of using a mind-body intervention to manage persistent pain and reduce medication dependence, providing clear implications for primary and tertiary pain care. |
| 97 | Bosco et al. 2025 | High: The study employed Cohen’s hermeneutic phenomenological approach, which integrates descriptive and interpretive frameworks to capture the essence of lived experiences. Rigor was maintained through the COREQ guidelines, triangulation involving multiple researchers, and member checking where participants validated the identified themes. Additionally, the researchers utilized bracketing and reflective journaling to minimize personal bias during data collection and analysis. | High: The findings are consistently supported by verbatim participant transcripts and field notes, ensuring a transparent link between raw data and the final themes. The study's conclusions regarding economic strain and social isolation are highly coherent with existing international literature on the challenges of managing Inflammatory Bowel Disease (IBD) in rural settings. | High: Data were collected from 17 participants, which is a sufficient sample size for a phenomenological study to achieve data saturation. The interviews provided rich, idiographic depth, capturing a balanced representation of genders, age groups, and specific IBD diagnoses (Crohn’s disease and ulcerative colitis). | High: The study is directly applicable to the review question as it specifically addresses the “knowledge gap” regarding IBD experiences in non-Anglophone rural contexts. The findings provide specific clinical implications, such as the need for tailored communication strategies and specialized training for local healthcare providers in remote areas. |
| 98 | Danes-Daetz et al.,  2025 | Moderate: The study followed a systematic phenomenological reduction and thematic analysis process with oversight from a diverse research team. However, the authors explicitly acknowledge several limitations, including the absence of detailed observational field notes and the lack of member checking, meaning participants did not verify the transcripts or the final thematic analysis. | High: There is a clear and transparent link between the raw data and the resulting themes, which are supported by numerous verbatim participant quotes. The findings regarding stigma and barriers are consistent with the socio-ecological framework applied and align with existing literature on chronic illness in similar cultural contexts. | High: The researchers interviewed 14 participants from Malaysia and Thailand, reaching data saturation where no new interpretations or issues emerged. The data collected is rich and idiographic, capturing diverse perspectives across different ethnic groups (Thai, Chinese, Malay) and religions (Buddhism, Islam). | High: The study is highly relevant as it directly investigates the lived experiences and perceptions of stigma among women with chronic knee pain in the specific context of Southeast Asia. It provides context-specific insights into how local environmental factors, such as access to public transport, and cultural practices impact management adherence. |
| 99 | Jiang et al., 2025 | High: The study followed the COREQ guidelines to ensure transparency and rigor. Researchers established trust by participating in daily care and using local dialects to facilitate rapport. Rigor was further supported by multivariate cross-validation, independent double-coding by two researchers, and member verification where participants validated the findings. | High: The findings are clearly linked to the underlying data through verbatim participant quotes that illustrate each subtheme. The analysis is theoretically grounded in the biopsychosocial model and a conceptual model of social isolation, ensuring that the results are structured and consistent with established frameworks. | High: The researchers achieved data saturation with a sample of 20 participants. Saturation was identified at the 17th participant and confirmed by three additional interviews that yielded no new information. The sample included diverse characteristics regarding sex, age, domicile, and types of chronic diseases. | High: The study is directly applicable to the review question, as it focuses specifically on the lived experiences of social isolation among older adults with chronic diseases. It provides unique, context-specific insights by exploring these mechanisms within the Chinese sociocultural environment. |
| 100 | Najem et al., 2025 | High: The study utilized a rigorous descriptive phenomenological framework and adhered to the Standards for Reporting Qualitative Research (SRQR). Rigor was maintained through a pilot-tested interview guide, researcher triangulation (analysis performed by three researchers), and the use of the Lincoln and Guba criteria to ensure credibility and dependability. | High: The findings are transparently supported by a wealth of verbatim participant quotes that illustrate the emotional, physical, and social impact of pain [46-75]. The themes are logically derived from the raw data and remain consistent with the established theoretical framework of Kleinman’s explanatory models. | High: Although the sample size was 12 participants, the researchers reached “information power,” ensuring that the data were rich enough to address the research objectives effectively. The sample provided a diverse representation of the Lebanese population, including equal numbers of men and women across various ages, religions, and geographic regions. | High: The findings are highly applicable to the clinical context of managing chronic pain in non-Western cultures. The study provides specific, context-sensitive insights into how traditional gender roles, the Lebanese economic crisis, and religious beliefs (such as “deferring” prayer styles) uniquely shape the experience of chronic low back pain. |
| 101 | Putri & Amir,  2025 | Moderate: The study included a purposive sample (n=12) from selected chronic-illness population with possible selection bias; limited detail on recruitment setting diversity and condition types; single-study, cross-sectional design limits the transferability; however, clear ethical approval, COREQ awareness, and appropriate phenomenological methods mitigate some concerns (Methods: Participants, Data collection, Ethics). | High: the study’s analytic process is clearly described and consistent (verbatim transcriptions, thematic analysis with staged coding, NVivo for organization), themes logically arise from data and align with research aims (Data analysis, Results, Discussion). | Moderate–High: The study conducted 12 in-depth interviews (60–90 min) with balanced gender and age range provide good depth for phenomenology; richness supports identified themes, though breadth across illness types and contexts is limited. | Moderate: The findings are directly relevant for understanding emotional, communicative, and social-support dimensions of chronic illness in clinical and communication contexts; applicability beyond the sampled population and cultural/healthcare contexts may be constrained.  Overall CERQual confidence: Moderate—findings are credible, coherent, and supported by rich interview data and transparent analysis, but confidence is tempered by purposive sampling, limited sample size/heterogeneity across conditions, and potential context-specific limits to transferability. |
| 102 | Shehadeh et al., 2025 | High: The study followed a rigorous descriptive phenomenological design and adhered to the COREQ guidelines for reporting. Rigor was ensured through reflexive practices (diaries and acknowledging researcher bias), pilot testing of interview questions, and peer debriefing. While the study used convenience sampling and had a small sample of 15 participants, these are common in qualitative research, and were addressed through high procedural transparency. | High: The findings are consistently supported by verbatim participant transcripts and field notes, ensuring a clear link between raw data and the final themes. The three overarching themes, education, infrastructure, and awareness, are logically derived from the systematic three-step inductive content analysis and are well-supported by specific examples from both nurses and physicians. | High: The researchers achieved data saturation, continuing recruitment until no new insights were identified. The data provided “thick description” and deep understanding of the participants' thoughts, with interviews lasting approximately one hour each to ensure richness. The sample size (N=15) is noted as sufficient for achieving saturation in this specific professional cohort. | High: The study is directly applicable to the research question, focusing specifically on strategies to improve healthcare for older people with chronic diseases in the unique Jordanian sociocultural context. It provides context-specific insights into cultural barriers, such as the lack of Middle Eastern-centric textbooks and the impact of local family structures on geriatric care. |
| 103 | Stilwell et al., 2025 | High: The study employed a rigorous front-loaded phenomenological design that utilized established theoretical frameworks to inform the research  . The authors followed COREQ and GRIPP2 reporting guidelines and integrated a participatory approach by involving patient partners in all aspects of the study  . Rigor was further established through triangulation of independent coding, reflexive practices, and member checking. | High: The findings are transparently supported by a wealth of verbatim participant quotes that directly illustrate the themes of disrupted agency, ownership, and time. The researchers used a systematic hybrid thematic analysis (inductive and deductive) that ensures a clear, logical link between the raw data and the conceptualized “modes of suffering.” | High: The researchers achieved “information power” with a sample of 12 participants who provided rich, “thick” descriptions of their lived experiences. The dataset was substantial, comprising over 20 hours of recorded audio from in-depth interviews lasting up to 120 minutes each. | High: The study is highly relevant to the review context, as it identifies a “new mode” of pain-related suffering that challenges traditional, self-reflection-based models. The findings provide direct clinical implications for managing patients who are too overwhelmed to engage in traditional cognitive-based therapies, suggesting a need for grounding and sensory-based interventions. |
| 104 | Safour et al., 2025 | High: The study employed a rigorous Interpretative Phenomenological Analysis (IPA) framework. Methodological quality was supported by researcher reflexivity, a collaborative analysis process between a master’s student and a PhD-qualified supervisor, and the use of expert-reviewed interview guides. The researchers also noted nonverbal cues to enhance data interpretation. | High: The four emerged themes are transparently supported by verbatim participant quotes that illustrate the physical, emotional, and social toll of TMDs. The findings regarding fatigue and functional limitations are highly coherent with existing literature on chronic orofacial pain. | Moderate: The sample size is small (6 participants), which the authors acknowledge as a limitation in terms of the generalizability of the results. However, the researchers utilized the concept of “information power,” ensuring that the interviews (lasting 30-90 minutes) provided rich, in-depth accounts suitable for the idiographic nature of IPA. | High: The study is directly relevant to understanding the holistic impact of chronic TMDs beyond clinical symptoms. It provides specific insights into how the condition affects dietary habits, communication in professional roles, and social identity, offering clear implications for integrated healthcare approaches. |

**Supplementary Table S8. Hermeneutic Theme Development, Coding structure, and Initial Heuristic Domains***

| Study Number | Author(s), Year | Codes/ Themes | Subthemes 1 | Subthemes 2 | Key Quote | Provisional Impact | Principal Heuristics/ Domains |
| --- | --- | --- | --- | --- | --- | --- | --- |
| 1 | Furnes & Dysvik, 2010 | Relearning daily life,  Grief, Chronic Pain | Adaptation processes, despair and hope | Identity reconstruction | "Relearning the world through loss and hope" | Medium | Resilience, social adaptation, role burden |
| 2 | Krohne & Ihlebaek, 2010 | Activity–rest fluctuation, social participation | Symptom variability | Activity limitation | "Symptoms fluctuate between activity and rest" | High | Social withdrawal, participation barriers |
| 3 | Skjutar & Müllersdorf, 2010 | Body–mind struggle, new self-identity, inner conflict, attention strategies, activity limitation | Self-image transformation | Self-identity change | "Inner conflict between rest and activity" | High | Social engagement, activity limitations, attention strategies, depression |
| 4 | Vallerand & Nowak, 2010 | Pre/post opioid contrast, burden of pain, burden of prescription opioids, activity limitations, depression, emotional distress | Secrecy and stigma, isolation, social withdrawal, further intricacies of pain and illness, family dynamics | Functional restoration vs stigma | "Life after opioids involved secrecy and gratefulnes" | High | Social isolation vs support systems, social stigma, disbelief, anxiety, depression |
| 5 | Hansson et al., 2011 | Discredited by clinicians, support systems, isolation, withdrawal, negativity, stigma | Invalidation by healthcare | System distrust | "Patients felt discredited and questioned" | High | Social stigma, labeling as malingering, disbelief, anxiety, doubt, depression |
| 6 | Skuladottir & Halldorsdottir, 2011 | Quest for diagnosis and care, quest for treatment, quest for normalcy, anxiety | Social/financial needs | Avoid isolation | "Quest to find a diagnosis and live with pain" | Medium | Social isolation, social stigma, coping mechanisms, meaning making, anxiety |
| 7 | Tollefson et al., 2011 | Isolation in rurality, anxiety, privacy as protection, stigma, loss of self | Healthcare access barriers | Geographic isolation | "Privacy as protection; no place here to get support" | Medium | Anxiety, depression, social stigma |
| 8 | Lavie-Ajayi et al., 2012 | Double-faced experience, anxiety, self-doubt, relational distress | Narratological distress | Experience ambivalence | Vivid yet elusive experience prompting self-doubt | Medium | Disbelief, anxiety, self-doubt, social stigma, family and social relationships |
| 9 | Nay & Fetherstonhaugh, 2012 | Patient-defined pain, relationships, support, grief, anxiety, caregiver responsibility, role shift | Staff response central | Validity of pain report | Pain is what the person says it is | Medium | Social support, emotional distress, stigma, discrimination, pain beliefs, anxiety |
| 10 | West et al., 2012 | Resilience expression | Positivity despite illness | Psychosocial resilience | Individuals express resilience and positivity | Medium | Coping mechanisms, purpose, social support systems |
| 11 | Snelgrove et al., 2013 | Intrusive painful body, social life, frustration, anxiety, depression | Fragmented self and roles | Self-fragmentation | Constant, intrusive, painful body fragments life | High | Social isolation, symptom burden, chronicity, depression, anxiety |
| 12 | Taylor et al., 2013 | Pain–trauma linkage, pain intensity, chronicity, emotional distress, memory processes, pain beliefs, role changes | Changed identity | Trauma-related pain identity | "Pain and intrusive memories are connected" | High | Trauma, pain intensity, chronicity, emotional distress, anxiety, self-esteem, pain beliefs |
| 13 | Daker-White et al., 2014 | Identity assault, symptoms burden, pain intensity, depression, anxiety, self-esteem, dependency on others | Biographical disruption | Chronic illness identity loss | "Symptoms assault self-identity and role competence" | High | Coping mechanisms, anxiety, depression, self-esteem, meaning making |
| 14 | Dysvik et al., 2014 | Group processes benefit, self-esteem, anxiety, validation | Self-understanding through groups | Group therapeutic value | "Group processes increased self-understanding" | Medium | Self-esteem, resilience, social support, peer support, social engagement |
| 15 | Furnes et al., 2014 | Active involvement, self-understanding, coping, support systems, learning and participation, depression, anxiety | Insight, social support | Group/community support, Peer-based rehabilitation | "Active involvement led to new insight and support" | High | Coping mechanisms, social support, illness understanding, learning, depression, anxiety |
| 16 | Juuso et al., 2014 | Malingering stigma, disbelief, pain beliefs | Acknowledgement importance | Social validation | "Being seen as a malingerer versus being acknowledged" | High | Social stigma, disbelief, social validation, self-esteem |
| 17 | Lima et al., 2014 | Phenomenological framing, understanding, patient-provider relationships, hope, meaning making, communication, validation | Self-management, acceptance | Theoretical reframing, controllability, role burden | "Phenomenological model could improve adherence" | Low | Illness understanding, coping, health literacy, acceptance |
| 18 | Mahon et al., 2014 | Loss and adaptation, emotional distress | Uncertainty and stress. Social participation barriers, role loss, social isolation | Adaptation process, depression, anxiety | "Sense of loss and adapting to new life" | Medium | Coping mechanisms, illness-understanding, anxiety, depression, role burden |
| 19 | Mathias et al., 2014 | Group acceptance, solidarity, emotional burden (depression, anxiety) | Facilitated change via exercise, peer support | Group therapy benefits, support, illness understanding | "Others understand my pain; I'm not alone" | Medium | Acceptance, coping mechanisms, resilience, social support |
| 20 | Merolli et al., 2014 | Social media affordances, self-management, online identity, health literacy, anxiety, depression | Connection and narration, disclosure management | Online peer support, isolation, positive reframing | "Social media mitigates isolation through connection" | High | Social support, isolation and loneliness, illness understanding, emotional distress |
| 21 | Nilsen & Anderssen, 2014 | Loneliness and stigma, emotional distress, anxiety | Return-to-work motivation, coping, illness-understanding | Social and vocational desire, stigma, discrimination | "Loneliness and fear of stigmatization; motivated to return to work" | Medium | Anxiety, social participation barriers, employment, work capacity |
| 22 | Ojala et al., 2014 | Pain dominance, intensity, withdrawal | Existential hopelessness | Pain centrality, catastrophizing, rumination | "Pain is the master; life is not worth living" | High | Pain intensity, comorbid symptoms, emotional distress, controllability, pain-beliefs |
| 23 | Brooks et al., 2015 | Opioid ambivalence, emotional distress, anxiety | Stigma and self-protection, self-esteem, controllability | Medication-related stigma, pain beliefs | "Feeling stigmatized and ambivalent about opioids" | Medium | Anxiety, self-esteem, concealment |
| 24 | De Villiers et al., 2015 | Multidimensional pain, emotional distress, depression, anxiety | Spiritual influence, social participation barriers, meaning making | Holistic impact spiritualist, social engagement and support | "Pain influences physical, psychosocial and spiritual domains" | Medium | Symptom burden, activity limitations, spirituality |
| 25 | Hearn et al., 2015 | Treatment gap control struggle. Emotional distress, access to care | Control and acceptance, social participation barriers | Peer support | "Perceived gap between treatments received and needed" | High | Coping mechanisms, resilience, illness understanding |
| 26 | Kirkham et al., 2015 | Visualizing pain, pain intensity | Objectification of pain, self-esteem, social participation barriers | Expressive externalization, pain beliefs | "Paintings objectified pain as punitive and sinister" | Low | Symptom burden, pain catastrophizing |
| 27 | MacNeela et al., 2015 | Identity intrusion, self esteem, coping mechanisms | Family and livelihood strain, work capacity | Social and economic consequences, withdrawal | "Pain creates family strains and loss of income" | High | Family dynamics, social participation barriers, financial strain |
| 28 | Ojala et al., 2015 (2015a) | Whole-person impact, emotional distress, anxiety, depression | Distress, suffering, loss of friendships | Loneliness, social withdrawal | "Pain affects the whole person with distress and lost identity" | High | Isolation and loneliness, disability, loss of friendships |
| 29 | Ojala et al., 2015 (2015b) | System rejection, stigma | Being disbelieved, malingering label | Institutional invalidation | "Being disbelieved and treated as a second-class citizen" | High | Emotional distress, social stigma, disbelief |
| 30 | Ojala et al., 2015 (2015c) | Reflective acceptance, positive reframing | Support, self-esteem | Altered self-meaning-making and acceptance | "Reconsidering values and acceptance of pain" | High | Coping mechanisms, peer support, resilience |
| 31 | Röing & Sanner, 2015 | Lived body alteration, self-esteem, limitations | Identity threat, distress, social disengagement, isolation vs acceptance | Diminished phenomenological disruption lifeworld | “A different lived body and a diminished lifeworld" | Medium | Symptom burden, social participation barriers, isolation and loneliness, activity limitation |
| 32 | Svensson et al., 2015 | Deceiving body fear, anxiety, fear, social roles | Isolation trajectory, avoidance behaviors | Fear, future uncertainty, worry | "Struggling to understand a deceiving body; fearing isolation" | Medium | Role burden, pain beliefs, fear avoidance, anxiety |
| 33 | Coleman et al., 2016 | Indescribable suffering, chronicity, duration, pain beliefs, coping strategies | Acceptance, avoidance, reframing, emotional distress | Appreciation, expressive limits and growth, stagnation | "Pain was unimaginable and almost impossible to describe" | Medium | Pain intensity, acceptance, positive reframing, distraction strategies |
| 34 | Dueñas et al., 2016 | Caregiver mental health, depression, anxiety | Caregiver anxiety/depression, family dynamics | Systemic impact, emotional distress | "Depression and anxiety in caregivers identified" | Low | Caregiver stress, anxiety, depression |
| 35 | Rambod et al., 2016 | Physical alteration, activity, isolation, escape | Psychological engagement, emotional distress, sleep disturbance | Impairment, limitations, engagement | "Alteration in physical health and impairment in social relationships" | Medium | Activity limitations, sleep issues, social withdrawal |
| 36 | Angel et al., 2017 | Recovery narratives, acceptance, distress | Regimens and coping, control | Reconstruction strategies | "Recovering and returning to prior lifestyle or developing strategies" | Medium | Coping mechanisms, attention strategies, breakthrough pain, emotional distress, controllability |
| 37 | Hassani et al., 2017 | Overcoming suffering, distress, spirituality, resilience | Purpose and spirituality, prayer, social support | Resilience factors | "Art of overcoming suffering through purpose and spirituality" | Medium | Purpose, faith, prayer |
| 38 | Morton et al., 2017 | Digital self-management, technologies, control, access | Active patient role, telehealth, coping | Telehealth empowerment | "Digital interventions encourage active roles and behavior change" | High | Pain assessment apps, telemedicine |
| 39 | Palacios-Ceña et al., 2017 | Invisible condition shame, anxiety | Physician support seeking, stigma and help-seeking | Social stigma, emotional distress | "Shame of suffering from an invisible condition" | Medium | Disbelief, pain beliefs, coping mechanisms |
| 40 | Schütze et al., 2017 | Metacognitive conflict, fragmentation | Rumination and worry, cognitive-emotional conflict, distress | Catastrophizing, coping, controllability | "Thinking helps me cope while rumination is uncontrollable" | High | Rumination, anxiety |
| 41 | Toye et al., 2017 | Impoverished life, distress, disclosure, self-esteem | Diagnostic quest and lost credibility, lived-experience synthesis | Stigma, withdrawal, concealment | "My life is impoverished and confined" | High | Social participation barriers, social withdrawal, disbelief, disability |
| 42 | Twiddy et al., 2017 | Thwarted opportunities, work capacity | Age-related validity, Youth-specific stigma, family | Isolation, disbelief | "Thwarted opportunities and illness validity in age context" | Low | Loss of friendships, role burden, family dynamics |
| 43 | Al Kalaldeh et al., 2018 | Philosophical framing, self-esteem | Suffering without synthesis, Conceptual understanding | Engagement, relationships | "Chronic illness affects self-perception and social interactions" | Low | Social participation barriers, depression |
| 44 | Cooper et al., 2018 | Pain as motivator/barrier, stigma, coping | Fear of damage and activity dichotomy, activity | Behavioral ambivalence, control | "Pain both motivates and hinders weight loss" | Medium | Pain beliefs, harm, fear avoidance, controllability, activity limitations |
| 45 | Gutke et al., 2018 | Body for identity, adaptation | Stages of change, coping | Identity and change | "Body importance for identity and stages of change" | Medium | Self-esteem, mobility, distribution, duration, symptom burden |
| 46 | Hearn et al., 2018 | Safety–adherence dichotomy, adherence despite adversity, treatment adherence tension | Resilience, positive reframing, safety perceptions, future worry | Dichotomy of safety perceptions and adherence despite adversity | "Dichotomy of safety perceptions and adherence despite adversity" | Medium | Health literacy, emotional distress, catastrophizing |
| 47 | Leder, 2018 | Lived space constriction, temporal disruption, withdrawal, temporospatial disturbance | Incarceration-related suffering, activity, depression, self-esteem | Constricted lived space and disrupted lived time | "Constricted lived space and disrupted lived time" | Low | Social isolation, withdrawal, loss of friendships, mobility, symptoms burden |
| 48 | Lee et al., 2018 | Uncertain legitimacy, role burden, isolation, control, self-esteem | Need to legitimize pain, Clinical uncertainty, fear | Frustration with professionals' explanation and need to legitimize | "Frustration with professionals' explanation and need to legitimize" | Medium | Depression, anxiety, fear avoidance, social participation barriers, rumination, social stigma |
| 49 | Hazaveh & Hovey, 2018 | Feeling isolated,  dissatisfaction | Loss of social identity, disbelief | Healthcare dissatisfaction | "Dissatisfaction with healthcare journey and loss of self-identity" | Low | Emotional distress, comorbid symptoms, social stigma and discrimination |
| 50 | McGeechan et al., 2018 | Post-treatment adaptation, comorbid symptoms, fatigue | Physical and psychological consequences, coping | Adjustment after illness, emotional distress | "Adapting to life after treatment" | Low | Symptoms burden, comorbidities, acceptance |
| 51 | Mengshoel et al., 2018 | Diagnosis validation, pain beliefs, emotional distress | Legitimization role of label, controllability | Diagnosis as social validator, disbelief | "Diagnosis validates bodily experiences and legitimates sickness" | High | Disclosure management, labeling as malingering, social stigma and discrimination |
| 52 | Stensland & Sanders, 2018 (a) | Life changed wholly, family, activity | Sense of trapped body, Life-altering impact | Work absenteeism, reduced productivity, direct and indirect costs | "It changed my whole life; trapped in a body that doesn't work" | High | Financial strain, work capacity, social isolation, role burden, family dynamics, mobility, disability, family dynamics |
| 53 | Stensland & Sanders, 2018 (b) | Corporeal temporality relationality, emotional distress | Living full of pain, Phenomenological dimensions of pain | Family, relationships | "Living a life full of pain across corporeality, temporality, relationality" | High | Pain intensity, chronicity, activity limitations, duration, comorbid symptoms |
| 54 | Stensland & Sanders, 2018 | Aging and stigma, depression, anxiety, distress | Falling apart and loss of independence | Pain and aging intersection | “Pain stigmatizes aging and threatens independence | High | Emotional distress, mobility, social stigma and discrimination, reduced productivity, activities of daily living |
| 55 | Bath et al., 2019 | Seasonality and isolation, withdrawal, coping mechanisms | Farming work constraints | Contextual occupational impact | Seasonality and isolation shape farmers' pain experience | Medium | Social isolation, activity limitations, mobility, environmental triggers, access to care |
| 56 | Cheng et al., 2019 | Appraisals and strategies, resilience | Maintaining normal life | Coping across multiple conditions | Strategies to maintain a normal life with multiple conditions | High | Positive reframing, acceptance, social support, social participation barriers |
| 57 | Hughes et al., 2019 | Identity through imagery, acceptance, activity | Interpersonal facilitators/barriers, coping, self-esteem, mobility | Quality of life and identity | Quality of life experienced through perception of self and identity | Medium | Family dynamics, social participation barriers, social isolation, activity limitations |
| 58 | Rodríguez et al., 2019 | Adaptation in home, mobility, disability | Strong family support, participation | Contextual cultural support, emotional distress | "Strong family and community ties offer support" | High | Disability, emotional distress, social support, family dynamics, activity limitations, built environment, access to care, environmental triggers |
| 59 | Schultz et al., 2019 | Fragmented treatment, distress, depression, anxiety | Feeling trapped, healthcare fragmentation | Isolation, withdrawal, physical limitations | "Fragmentation of treatment and feeling trapped" | Medium | Controllability, social stigma and discrimination, disbelief, social participation barriers, duration, chronicity |
| 60 | Toye et al., 2019 | Need for support, catastrophizing, worrying about future roles, disclosure, work capacity | Chronic disease changing the sense of self, coping, acceptance | Illness temporality | Rheumatoid arthritis disrupts present and future self" | High | Catastrophizing, symptom burden, comorbidities, activity limitations, disease knowledge, social stigma and discrimination, concealment |
| 61 | Mustafa et al., 2020 | Dual, gender role immigrant role strain, chronic recurrence, activity limitations | Cultural and gender-related disparities in pain burden | Mobility, family roles | "Dual-gender role after immigration exacerbates pain" | Medium | Pain intensity, duration, family dynamics, role burden, social isolation, cultural pain expressions |
| 62 | Roenne et al., 2020 | Relatives’ role, family dynamic | Loss of relationships, emotional distress | Relational involvement | "Relative involvement arbitrary; profound need to be understood" | Medium | Social support, loss of friendships, social isolation, social participation barriers |
| 63 | Skúladóttir et al., 2020 | Vicious circle breaking, coping and adaptations | Rehabilitation journey, emotional distress | Recovery trajectory | Journey of breaking the vicious circle of chronic pain | Medium | Positive reframing, concealment, social support |
| 64 | Yildirim Duman, 2020 | Social support and empowerment | Disease self-management | Social empowerment, self-esteem | Social support, disease management, and self-awareness | Low | Health literacy |
| 65 | Andersen et al., 2021 | Aesthetic/ethical/religious framing, emotional distress | Adaptive strategies, spiritual strategies | Existential dimensions of illness | Illness perceived as unwelcome alteration with ethical/religious dimensions | Low | Acceptance, purpose, faith, prayer |
| 66 | Fracso et al., 2021 | Self-empowerment via program, coping and resiliency | Peer belonging, disease knowledge | Self-management program effects | Program improved self-empowerment and belonging | Low | Peer support, social support |
| 67 | Honoré Grauslund et al., 2021 | Overwhelming pain, distress, depression | Community distress, anxiety | Search for resolution, coping | Overwhelming pain and looking for a way out | Medium | Pain intensity, chronicity, social isolation, social participation barriers, social withdrawal |
| 68 | Keen et al., 2021 | PNE theoretical validation, pain beliefs, health literacy | Practical inadequacy, social engagement barriers | Education vs practice tension | PNE was intellectually engaging but practically inadequate | Medium | Social stigma and discrimination, disbelief |
| 69 | Niño de Guzmán Quispe et al., 2021 | Self-management domains, disease knowledge | Therapeutic process components | Comprehensive self-management taxonomy | Self-management involves social support, access, costs, culture, and knowledge | High | Social support, peer support, Family, access to care, financial strain, direct costs, indirect costs, cultural health beliefs |
| 70 | Stensland, 2021 (a) | Managing the incurable, coping strategies | Quest for relief and pragmatic coping | Chronicity management, controllability, pain beliefs | Managing the incurable with ongoing disappointment and making things work | High | Anxiety, depression, acceptance, procedures, medications |
| 71 | Stensland, 2021 (b) | Helplessness and perseverance, coping and control | Emotional persistence, acceptance | Chronic despair and endurance | Helplessness because nothing works, yet I keep going | High | Emotional distress, anxiety, depression |
| 72 | Toye et al., 2021 | Validation and reconnection, pain beliefs | Facilitating recovery, coping, reframing | Healing and recovery synthesis | Validate pain, encourage self-connection and safe reconnection | High | Positive reframing, social support, family, social participation barriers, social isolation, disbelief, comorbidities |
| 73 | Chala et al., 2022 | Invisibility leads to loneliness, distress | Quest for cure and individual coping and control | Invisibility and coping | Invisibility of pain results in misunderstanding and loneliness | Medium | Social isolation, activity limitations, anxiety, symptom burden |
| 74 | Kahere et al., 2022 | Access barriers systemic, access to care | Infrastructure and personnel shortages, support systems | Health system barriers | Barriers include travel, long waits, and staff shortages | Medium | Transportation, rural/ urban disparities, social support, access to pain clinics, insurance issues |
| 75 | Altun et al., 2023 | Cultural/gender influences, health beliefs | Help-seeking barriers, access to care | Culture, gender, and access, emotional distress | Influence of culture and gender roles on accessing appropriate care | Medium | Family dynamics, social participations barriers, coping mechanisms |
| 76 | Chimezie, 2023 | Health literacy and disease knowledge | System weaknesses and cultural beliefs | Prevention and access disparities | Health literacy and socioeconomic factors shape chronic disease management | Low | Financial strain, indirect costs, cultural health beliefs, rural/urban disparities |
| 77 | Joyce et al., 2023 | Education empowers and coping, resiliency | Social connectedness | Therapeutic empowerment vs distress | Empowerment through education and exercise | Medium | Social support, peer support, activity limitations, comorbidities, symptom burden |
| 78 | Kjærulff et al., 2023 | Social media influence and support | Peer-to-peer communication | Online influence on care, health literacy | Social media shapes discussions about medical advice and HCP perceptions | Medium | Social support, peer support, disclosure management |
| 79 | Peppard et al., 2023 | Resilience as new normal, acceptance | Mission-first invisibility, participation | Veteran-specific pain experience, disbelief | Resilience in living with chronic pain is the new normal | Medium | Pain intensity, symptom burden, social stigma and discrimination |
| 80 | Sun et al., 2023 | Transition barriers/facilitators | Caregiver and nurse roles, access to care | Hospital-to-home transition, rural disparities | Family caregiver support and nurse coordination facilitate transitions | High | Caregiver stress, family |
| 81 | van Rysewyk et al., 2023 | Emotional burden and identity changes, resilience | Management struggles, coping mechanisms | Validated pain community themes, withdrawal | Emotional burden and identity changes validated by survey | High | Pain intensity, disability, chronicity, comorbidities, social participation barriers, social isolation role burden |
| 82 | Albán-Reyes et al., 2024 | Occupational load and injury, illness temporalities | Coping via self-affirmation | Work-related chronic pain | Dancers used self-affirmation to continue their profession | Low | Comorbidities, symptoms burden, chronicity, role burden, activity limitations |
| 83 | Bruger et al., 2024 | Solitude and acceptance, distress | Healthcare dissatisfaction, participation barriers, disbelief | Stroke-related chronic pain experience | Solitude of the pain experience and need for self-care | Medium | Social isolation, social withdrawal |
| 84 | Bullo et al., 2024 | Metaphor of violence, pain beliefs | Color/texture/material metaphors, distress | Embodied metaphorical pain | Pain as physical violence expressed via color and texture | Low | Comorbidities, symptom burden, |
| 85 | Gaber et al., 2024 | Participatory empowerment, resiliency, coping | Cultural and social change | Participatory research outcomes | "Participatory approaches produce empowerment and cultural change" | Medium | Coping mechanisms, family, social support |
| 86 | Lilja et al., 2024 | Top-performer vs supported | Acceptance of support Independence and assisted care tension | Self-esteem, acceptance | "Balancing being most valuable player and accepting support" | Low | Social support, family dynamics, role burden |
| 87 | Lo Monaco et al., 2024 | Trauma before diagnosis, distress | Misunderstanding and maternal role struggle | Fibromyalgia multifaceted burden | "Trauma preceding diagnosis and pervasive misunderstanding" | Medium | Disbelief, social stigma and discrimination, family, role burden, symptom burden, comorbidities |
| 88 | Lundin et al., 2024 | Safe/valuable/trusted supports | Work transition support, absenteeism | Work reintegration factors | "Feeling safe and valuable supports return-to-work" | Medium | Work capacity, employment, social support, access to care |
| 89 | Macgregor et al., 2024 | Acceptance as ecosystem | Fluid socio-cultural interdependence | Acceptance process, coping | "Acceptance is a fluid ecosystem interdependent with culture" | Low | Positive reframing, social support |
| 90 | Matias-Soto et al., 2024 | Emotional cognitive behavioral factors, anxiety, distress, depression | Diagnosis and health system influences | Multidimensional pain experience | "Fears, frustrations, and strategies for managing symptoms" | Medium | Disability, activity limitations, coping mechanisms, social participation barriers |
| 91 | Nikoloudi et al., 2024 | Cultural stigma and exclusion | Biographical disruption, participation, distress | Stigmatized chronic condition | "Severe stigmatization leading to social exclusion and loss of identity" | Medium | Social stigma and discrimination, isolation and loneliness, cultural competence |
| 92 | Okolo et al., 2024 | Cultural variability in pain, ethnic disparities, traditional healing practices | Traditional/biomedical/spiritual strategies, spiritual coping | Culturally sensitive care | Pain perception and management vary widely across cultures | Low | Cultural competence, cultural pain expression, stoicism, norms about pain communication |
| 93 | Sanabria-Mazo et al., 2024 | Group psychotherapy benefits, coping and resilience | Social isolation as barrier | Psychotherapy and social contact | Behavioral and cognitive improvements; barriers: lack of human contact | High | Depression, anxiety, pain beliefs |
| 94 | Schwarz B et al., 2024 | Return-to-work multi-actor model, self-esteem, support systems | Coping, support, workplace adaptability, access to care | Work reintegration complexity | Return-to-work depends on perception, coping, support, and workplace adaptation | High | Employment, work capacity, social support |
| 95 | Snell et al., 2024 | Masculinity norms hinder help-seeking, cultural health beliefs | Identity and help-seeking | Masculine norms and illness | Hegemonic masculine norms negatively affect help-seeking | Low | Social stigma, disclosure management |
| 96 | Barber et al., 2025 | Trauma reprocessing via Yoga Nidra | Effects on identity, coping mechanisms | Mind–body integrative benefit | Yoga Nidra aided trauma reprocessing and improved self-efficacy | Low | Acceptance, controllability, post-traumatic growth |
| 97 | Bosco et al., 2025 | Rural isolation | Psychosocial geographic effects | Geographic/social isolation impact | "Psychological and social dimensions of geographic isolation are important" | Low | Social isolation and loneliness, participation barriers |
| 98 | Danes-Daetz et al., 2025 | Shared experience coping, distress | Multilevel barriers (individual to community) | Collective management and barriers and stigma | "Shared struggles and barriers across individual to community levels" | Low | Pain intensity, activity limitations, anxiety |
| 99 | Jiang et al., 2025 | Guilt/stigma/emotional stress, symptoms | Social deterioration and lack of support, participation barriers | Isolation determinants | "Guilt, stigmatization, and lack of social support drive isolation" | Medium | Social isolation and loneliness, symptom burden, rural/urban disparities |
| 100 | Najem et al., 2025 | Cultural context of low back pain | Social influence on pain, social components of health, cultural aspects | Cultural framing of low back pain | "Chronic low back pain shaped by Arabic cultural influences" | Low | Cultural health beliefs, family dynamics, social participation barriers, emotional distress |
| 101 | Putri & Amir, 2025 | Emotional distress and coping | Social network influence | Long-term emotional support need | "Emotional distress shaped by social networks; need long-term support" | Low | Social support, family, peer support |
| 102 | Shehadeh et al., 2025 | Provider education and resources | System-level improvement needs, cultural aspects of health | Healthcare workforce development | "Need for dedicated courses and improved facilities/resources" | Low | Access to care, employment, cultural competence |
| 103 | Stilwell et al., 2025 | Minimal-self disruption, self-esteem, depression | Contextual fluidity of self, controllability | Self-agency disruption | "Pain-related minimal-self disruption involves disrupted agency" | Medium | Pain beliefs, anxiety |
| 104 | Safour et al., 2025 | Fatigue/frustration/limitations | Communication and accommodation challenges | Clinical access and communication barriers, withdrawal from social life | "Frustrations seeking medical help and communication challenges" | Low | Activity limitations, mood disorders, social participation barriers, social isolation |

*This table shows the subsequent steps of the synthesis process, shown in Table S3. Coding and QA: See Phase/Protocol tables (Supplementary Tables S1–S4) for full procedures. The initial extraction and testing of the structured form were conducted using SSA and PD with methodological oversight from the RT. Line-by-line coding was performed primarily by SSA and EAV; two authors (EAV and SSA) independently appraised the study quality (CASP and MMAT, where applicable). Intercoder reliability was assessed via double‑coding (pilot n=10; subset checks across dataset) with ≥80% raw agreement and Cohen’s kappa ≈0.80 (95% CI 0.75–0.85). Disagreements were resolved by adjudication with the PD and full‑team consensus, and all resolution decisions were recorded in the audit trial. The analytical framework (see Supplementary Table S3 for search strings and databases) combines thematic synthesis and meta-ethnography informed by hermeneutic phenomenology and heuristic/process ontologies (Thomas & Harden 2008; Noblit & Hare 1988). The searches covered January 2009 to December 2025 across PubMed/MEDLINE, Scopus, Web of Science, PsycINFO, ProQuest, AMED, and Google Scholar with forward/backward citation tracking (exact strings and hit counts in Supplementary Table: Search_Strategy). Inclusion/exclusion: Included published primary qualitative studies, qualitative evidence syntheses, and mixed‑methods studies reporting adult (≥18) lived experience of chronic pain or closely related constructs (e.g., chronic shame). Quantitative studies, protocols, and reports without primary participant accounts were excluded. CASP threshold ≥70% (sensitivity analyses performed for lower‑scoring studies). The full codebook, study×code matrix, CERQual evidence profiles, PRISMA flow, inter-rater tables, and audit‑ trials are provided in the Supplementary Materials (see below).

**Supplementary Table S9. Coding Extraction Matrix (codes, descriptions, example studies)***

| Hermeneutic Theme/ Code | Description of Themes | Heuristic Themes/ Domains | Example Studies |
| --- | --- | --- | --- |
| Pain as central/omnipresent | Chronic pain dominates daily life, activities, and identity; rumination; catastrophizing; anxiety; depression | Chronic Pain/ Physical/ Cognitive | 11, 21, 27, 30 |
| Invisible/invalidated pain | Pain is unseen by others; patients feel disbelieved or dismissed, role burden, disbelief, pain beliefs, cultural pain expression, harm | Social isolation, Psychological, Cognitive, Cultural, Physical | 5, 29, 39, 41 |
| Loss of self/identity | Biographical disruption; change or loss of former self, controllability, pain beliefs, anxiety, depression, symptom burden, discrimination | Psychological, Social, Physical | 13, 27, 28, 91 |
| Stigma and shame | Feeling judged, labeled, or ashamed because of condition, stigma, isolation, support, fear avoidance | Social, Psychological, Physical | 4, 23, 39, 91 |
| Social isolation/withdrawal | Loss of social life, retreat from relationships and roles, family dynamics, concealment, peer support | Social, Psychological, Cognitive | 27, 55, 62, 79 |
| Struggle for legitimacy | Quest for diagnosis, proof, or validation from clinicians, acceptance, avoidance | Social, Psychological | 6, 41, 72 |
| Emotional burden (fear/anger/grief) | Intense affective responses to illness and loss, anxiety, depression, emotional distress | Psychological, Cognitive | 18, 22, 30, 79 |
| Role loss and role change | Inability to fulfill work, family, or social roles, role burden, family dynamics, disbelief | Social, Psychological, Cultural, Cognitive | 27, 33, 54 |
| Adaptation and acceptance | Processes of reframing life, accepting limitations, reconciling, acceptance, self-esteem, coping mechanisms | Social, Psychological, Spiritual | 15, 30, 72, 89 |
| Fight for control/agency | Attempts to regain autonomy and manage symptoms, controllability, acceptance | Psychological, Social | 25, 70, 71 |
| Identity reconstruction | Rebuilding a self that includes illness, health literacy, health beliefs, disease knowledge | Cognitive, Cultural, Social, Psychological | 3, 28, 30 |
| Secrecy and concealment | Hiding illness or treatments from others, concealment, isolation, friends, withdrawal | Psychological, Cognitive, Social | 4, 41 |
| Fatigue and embodied limitation | Chronic tiredness and reduced capacity for tasks, sleep disturbances | Cognitive, Psychological | 95, 104, 55 |
| Struggles seeking help | Barriers, miscommunication, or dissatisfaction with healthcare, social participation barriers | Social, Cognitive, Psychological, Cultural | 5, 48, 98 |
| Peer connection/validation | Finding understanding via peers, groups, or online communities, peer support, social support, family | Social, Cultural, Psychological | 15, 20, 72 |
| Coping strategies (practical/emotional) | Behavioral and cognitive strategies to manage daily life | Cognitive, Psychological | 36, 19, 90 |
| Spirituality/meaning making | Using faith or meaning frameworks to endure and interpret illness | Spirituality, Cognitive | 37, 65 |
| Financial/structural hardship | Economic strain from illness or healthcare, employment, disability, insurance, symptom burden | Physical, Social, Economic | 27, 29, 76 |
| Anticipatory anxiety/uncertainty | Fear about the future, prognosis, or flare-ups, catastrophizing | Cognitive, Psychological | 22, 32, 90 |
| Body betrayal/deceiving body | Feeling the body is unpredictable or betrays self, distribution, | Physical, Psychological, Cognitive, | 32, 3, 28 |
| Medication ambivalence | Conflicted feelings about analgesics or opioids, opioid prescribing | Policy/ Legal, Social | 4, 23 |
| Healthcare fragmentation | Disjointed care, multiple providers, inconsistent treatment | Cultural, Psychological, Physical | 59, 23, 81 |
| Self-management and empowerment | Active self-care, learning, and regaining agency | Physical, Social, Cognitive | 20, 66, 77 |
| Metacognitive distress | Rumination, uncontrollable worry, thought conflicts | Psychological, Cognitive | 40 |
| Cultural/contextual meanings | Illness meanings shaped by culture, gender, migration | Cultural, Psychological, Cognitive, Physical | 61, 82, 100 |
| Caregiver/care network impact | Effects on family caregivers and relational dynamics | Social, Psychological | 34, 62 |
| Occupational/role identity tensions | Work identity versus functional limitations | Environmental, Economic, Physical | 55, 88 |
| Trauma interplay with pain | Pain intertwined with past trauma and intrusive memories | Psychological, Social, Cognitive, Physical | 12, 87 |
| Hope and future orientation | Holding hope, planning, or imagining a life ahead | Cognitive, Psychological | 22, 15 |
| Pain as central/omnipresent | Chronic pain dominates daily life, activities, and identity | Physical, Psychological, Cognitive | 11, 21, 27, 30, 52, 55 |
| Invisible/invalidated pain | Pain is unseen; patients feel disbelieved or dismissed | Physical, Psychological, Social, Cultural | 5, 29, 39, 41, 23 |
| Chronic pain stigma and shame | Feeling judged, labeled, or morally blamed for pain | Psychological, Physical | 4, 23, 39, 91 |
| Pain-driven identity loss | Biographical disruption and loss of former self | Physical, Social, Psychological, Cognitive | 13, 27, 28, 91, 33 |
| Social isolation and withdrawal | Reduced social contact, withdrawal, loneliness | Social, Psychological | 27, 55, 62, 79, 31 |
| Quest for diagnosis/legitimacy | Searching for diagnosis, proof, or validation | Cognitive, Psychological, Cultural | 6, 41, 72, 28 |
| Emotional burden (fear/anger/grief) | Intense affective responses to persistent pain, symptom burden, comorbidities | Physical, Psychological | 18, 22, 30, 79, 90 |
| Role loss and role change | Inability to perform work/family roles due to pain | Social, Psychological, Physical | 27, 33, 54, 88 |
| Adaptation and acceptance | Process of reframing life and accepting pain | Cognitive, Psychological, Physical | 15, 30, 72, 89 |
| Fight for control/agency | Attempts to regain autonomy and symptom control | Cognitive, Cultural, Spiritual | 25, 70, 71, 77 |
| Body betrayal/deceiving body | Body feels unpredictable, deceptive, or betraying self | Physical, Psychological | 32, 3, 28, 11 |
| Secrecy and concealment | Hiding pain severity, medication use, or diagnosis | Social, Environmental, Physical | 4, 41, 23 |
| Medication/treatment ambivalence | Conflicted feelings about opioids/medications/treatments | Policy/ Legal, Environmental, Social, Cultural | 4, 23, 70, 71 |
| Healthcare fragmentation and distrust, distress, burden | Disjointed care, poor coordination, clinician distrust, emotional distress, symptom burden | Social, Physical, Cultural, Psychological | 59, 23, 29, 48 |
| Help-seeking struggles | Barriers, miscommunication, and dissatisfaction with care | Cognitive, Psychological, Cultural | 5, 48, 98, 100 |
| Financial and structural hardship | Economic strain from chronic pain and access issues | Economic, Environmental | 27, 29, 76, 74 |
| Fatigue and functional limitation | Chronic tiredness, reduced capacity, activity limitation | Cognitive, Psychological, Physical | 95, 104, 55, 21 |
| Pain as invisible work | Managing pain privately while maintaining appearances | Physical, Psychological, social, Cultural | 41, 72, 11 |
| Peer support and validation | Support from groups, peers, online communities mitigates isolation |  | 15, 20, 72, 66 |
| Self-management and empowerment | Active coping, education, exercise, and self-efficacy | Spiritual, Social, Cognitive | 20, 66, 77, 19 |
| Emotional distress, catastrophizing | Rumination, uncontrollable worry, catastrophic thinking | Cognitive, Psychological | 40, 11 |
| Trauma–pain interplay | Pain entangled with prior trauma and intrusive memories | Physical, Psychological, Cognitive | 12, 87, 33 |
| Coping strategies (practical/emotional) | Behavioral, cognitive and ritualized coping methods | Psychological, Cognitive | 36, 19, 90, 73 |
| Occupational identity tension | Work identity vs. pain-imposed limitations and RTW issues | Economic, Environmental | 55, 88, 94 |
| Relational impact and caregiver roles | Pain's effects on partners, relatives, and support networks | Social, Psychological | 62, 34, 21 |
| Cultural and contextual meanings | Cultural shaping of pain expression, help-seeking, and stigma | Cultural, Social, Physical | 61, 82, 100, 99 |
| Hope, future orientation and agency | Holding hope, planning, small goals despite pain | Cognitive, Social, Physical | 22, 15, 72 |
| Embodied metaphor and imagery | Use of metaphors/visuals to represent pain experience | Cognitive, Physical | 26, 84, 33 |
| Digital/online affordances | Use of social media/telehealth for connection and self-management | Technological, Cultural | 20, 38 |
| Health literacy and access | Knowledge, costs, and navigation affecting pain care | Cognitive, Social | 69, 76, 78 |

*Example studies are listed by reference number corresponding to the number in the manuscript reference list.

Codes were derived via iterative inductive–deductive coding and mapped to higher-order themes, where cells indicate initial theme labels and exemplar study references. The full study×code matrix, exemplar quotations, codebook definitions, and inter-rater tables are provided in Supplementary Files S10–S15.

**Supplementary Table S10. Coding/Hermeneutic Theme Scenarios Example Studies**

| Code/ Hermeneutic Theme | Description | Example Quote | Mapped Analytic Theme | Condition | Example Studies |
| --- | --- | --- | --- | --- | --- |
| Body betrayal / deceiving body | Body feels unpredictable, betrays my trust | "My body lets me down when I need it most." | Embodiment and mistrust | Chronic pain | 3 Skjutar & Müllersdorf, 2010; 32 Svensson et al., 2015; 28 Ojala et al., 2015 |
| Medication/treatment ambivalence | Conflicted feelings about meds (help vs harm) | "I need opioids but hate what they do to me." | Treatment dilemmas | Chronic pain | 4 Vallerand & Nowak, 2010; 23 Brooks et al., 2015; 70 Stensland, 2021 |
| Healthcare fragmentation / distrust | Disjointed care, poor coordination | "No one talks to each other; I'm the only constant." | Systemic barriers | Both | 59 Schultz et al., 2019; 23 Brooks et al., 2015; 29 Ojala et al., 2015 |
| Struggles seeking help | Barriers, dismissal, poor communication | "I kept asking for help but was ignored." | Access and advocacy | Both | 5 Hansson et al., 2011; 48 Lee et al., 2018; 74 Kahere et al., 2022 |
| Peer connection / validation | Support and normalization from peers | "Others like me finally understood what I was going through." | Communal support and belonging | Both | 15 Furnes et al., 2014; 20 Merolli et al., 2014; 66 Fracso et al., 2021 |
| Self-management and empowerment | Learning skills, routines, confidence | "Learning exercises gave me back some confidence." | Capacity building | Both | 19 Mathias et al., 2014; 66 Fracso et al., 2021; 77 Joyce et al., 2023 |
| Fatigue and physical limitations | Functional limitation | "I'm exhausted after performing simple tasks." | Reduced capacity, chronic tiredness | Both | 55 Bath et al., 2019; 95 Snell et al., 2024; 104 Safour et al., 2025 |
| Role loss and role change | Inability/ change in work, family roles | “I can't work full time anymore—it's humiliating." | Socio-occupational impact | Both | 27 MacNeela et al., 2015; 54 Stensland & Sanders, 2018; 88 Lundin et al., 2024 |
| Financial/structural hardship | Economic strain from illness | "We had to use savings to pay for treatment." | Socioeconomic consequences | Both | 27 MacNeela et al., 2015; 29 Ojala et al., 2015; 76 Chimezie, 2023 |
| Anticipatory anxiety / uncertainty | Worry about flare-ups, future losses | "I'm always waiting for the next bad day." | Uncertainty and temporality | Both | 22 Ojala et al., 2014; 30 Ojala et al., 2015; 90 Matias‑Soto et al., 2024 |
| Trauma–illness interplay | Pain linked to past trauma | "Every pain flashback brings back the old abuse." | Embodiment nexus | Both/Chronic illness | 12 Taylor et al., 2013; 87 Lo Monaco et al., 2024; 33 Coleman et al., 2016 |
| Embodied metaphor and imagery | Metaphors/images to describe suffering | "My back feels like it's on fire." | Meaning making through metaphor | Both | 26 Kirkham et al., 2015; 84 Bullo et al., 2024; 33 Coleman et al., 2016 |
| Narrative disruption / narratological distress | Loss of coherent life-story | "I don't know how to tell my story anymore." | Narrative rupture and repair | Chronic illness | 41 Toye et al., 2017; 13 Daker‑White et al., 2014; 72 Toye et al., 2021 |
| Caregiver/care social network impact | Effects on family caregivers | "My wife became my full-time caregiver overnight." | Relational burden and reciprocity | Both | 62 Roenne et al., 2020; 34 Dueñas et al., 2016; 52 Stensland & Sanders, 2018 |
| Cultural/contextual meanings | Cultural shaping of illness meanings | "In my culture, pain is a sign of weakness." | Cultural framing and interpretation | Both | 61 Mustafa et al., 2020; 92 Okolo et al., 2024; 100 Najem et al., 2025 |
| Occupational/identity tension | Work identity vs reduced capacity | "I still see myself as a provider, but I can't provide." | Identity vs. role conflict | Both | 55 Bath et al., 2019; 88 Lundin et al., 2024; 94 Schwarz et al., 2024 |
| Digital/online affordances | Use of online groups/telehealth | "I found a forum that finally made me feel less alone." | Technology-mediated support | Both | 20 Merolli et al., 2014; 38 Morton et al., 2017; 78 Kjærulff et al., 2023 |
| Metacognitive distress / rumination | Uncontrollable worry, catastrophizing | "I can't stop thinking it will only get worse." | Cognitive-emotional regulation | Chronic pain | 40 Schütze et al., 2017; 11 Snelgrove et al., 2013 |
| Hope and future orientation | Holding hope, small goals | "I'm learning to plan small joys again." | Resilience and future-making | Both | 15 Furnes et al., 2014; 72 Toye et al., 2021; 77 Joyce et al., 2023 |
| Spirituality / meaning‑making | Faith/existential frameworks | "My faith helps me make sense of this suffering." | Existential coping | Both | 37 Hassani et al., 2017; 65 Andersen et al., 2021 |
| Negotiating identity with others | Managing disclosure and presentation | "I explain less to avoid pity, more to get accommodations." | Stigma management and disclosure | Both | 41 Toye et al., 2017; 23 Brooks et al., 2015; 29 Ojala et al., 2015 |
| Embodied social performance (invisible work) | Concealing symptoms to meet expectations | "I push through so no one knows I'm ill." | Social performance and labor | Chronic pain/Both | 4 Vallerand & Nowak, 2010; 41 Toye et al., 2017; 63 Skúladóttir et al., 2020 |
| Care transitions / coordination problems | Discontinuity between services | “Leaving hospital was like falling off a cliff." | Continuity of care | Both | 80 Sun et al., 2023; 59 Schultz et al., 2019; 29 Ojala et al., 2015 |
| Illness as chronic temporality | Altered sense of time, stuck now | "Time slowed; my life is on hold." | Temporal disruption | Both | 47 Leder, 2018; 30 Ojala et al., 2015; 72 Toye et al., 2021 |
| Work & employment | Absenteeism, reduced productivity, job loss, workplace limitations | "…we assessed several aspects of how CP influences… its repercussions in the workplace." | Economic participation & role functioning | Chronic pain | 4 Vallerand & Nowak, 2010; 41 Toye et al., 2017; 63 Skúladóttir et al., 2020; Dueñas et al.2016 |
| Moral injury and blame | Feeling judged or blamed | "They treat me like I chose to be sick." | Moral exclusion | Both | 23 Brooks et al., 2015; 29 Ojala et al., 2015; 13 Daker‑White et al., 2014 |
| Embodied pleasure / small joys | Finding small, sensory relief | "A cup of tea can make a painful day bearable." | Resilience | Both | 36 Angel et al., 2017; 15 Furnes et al., 2014 |
| Collective activism / advocacy | Community action to change systems | "We started a support group to demand better care." | Collective agency | Chronic illness | 85 Gaber et al., 2024; 72 Toye et al., 2021; 20 Merolli et al., 2014 |
| Practical coping routines | Pacing, rituals, planning | "I pace my day to avoid crashes." | Routine work and pacing | Both | 55 Bath et al., 2019; 19 Mathias et al., 2014; 63 Skúladóttir et al., 2020 |
| Relational validation vs. invalidation | Relatives/providers validate or negate | "My mother believes me; my doctor doesn't." | Relational validation dynamics | Both | 5 Hansson et al., 2011; 29 28, 29, 30 Ojala et al., 2015; 72 Toye et al., 2021 |
| Daily activities & quality of life | Impact on routine functioning, sleep, leisure, and overall wellbeing | "Chronic pain (CP) seriously affects the patient's daily activities and quality of life." | Functioning & quality of life | Chronic pain | 34 Dueñas et al.2016; Ojala et al., 2015; 72 Toye et al., 2021 |
| Family & social environment | Caregiver burden, relationship strain, social isolation | "My relationships have changed social and family environment." | Social support & interpersonal impact | Chronic pain | 72 Toye et al., 2021; 34 Dueñas et al.2016; 30 Ojala et al., 2015 |

**Supplementary Table S11. Hermeneutic Code development (codes, descriptions, examples, analytic mapping, condition assessments)***

| Code/ Theme | Description | Example Quote | Mapped Analytic Theme | Assessment of the Condition |
| --- | --- | --- | --- | --- |
| Pain as central/omnipresent | Chronic pain dominates daily life, activities, and identity | "My pain is in everything I do; it runs my life." | Activity limitations, Symptom burden | Chronic pain |
| Invisible/invalidated pain | Pain/illness not visible; others disbelieve or dismiss experience | "They say I look fine, so it must be all in my head." | Physical limitations, psychological distress, social withdrawal, concealment | Both |
| Loss of self/identity | Biographical disruption; former roles and self-image lost | "I am not the person I used to be." | Legitimacy and recognition, isolation and stigma | Chronic illness |
| Stigma and shame | Feelings of blame, moral judgment, embarrassment about condition | "I feel dirty telling people I need help." | Identity disruption and reconstruction, catastrophizing, worry, withdrawal, isolation | Both |
| Social judgment and concealment | Social isolation/withdrawal | "I stopped going out—it's easier to stay home." | Social disconnection | Both |
| Quest for diagnosis/legitimacy | Chronic illness | "I just wanted someone to tell me what was wrong." | Search for explanation, diagnostic label, and validation | Chronic illness |
| Search for meaning and validation | Secrecy and concealment | "I hide my pills, so no one thinks I'm weak." | Hiding symptoms or treatments to preserve identity or avoid stigma | Chronic pain |
| Impression management | Emotional burden (fear/anger/grief) | "Some days I just cry for the life I lost." | Intense affective responses: grief for losses, fear about future | Both |
| Affective work and mourning | Fight for control/agency | "I try different routines to feel some control." | Active attempts to manage illness/pain and regain autonomy | Chronic illness |
| Self-esteem and agency | Adaptation and acceptance | "I learned to live with it; not let it define me." | Process of reframing life, accepting limitations, finding a new normal | Both |
| Adaptation and meaning making | Body betrayal / deceiving body | "My body lets me down when I need it most." | Body feels unpredictable and betrays trust, undermining plans | Chronic pain |
| Embodiment and mistrust | Medication/treatment ambivalence | "I need opioids but hate what they do to me." | Conflicted feelings about medications (help vs stigma/side-effects) | Chronic pain |
| Treatment dilemmas | Healthcare fragmentation / distrust | "No one talks to each other; I'm the only constant." | Disjointed care, poor coordination, and mistrust of providers | Both |
| Systemic barriers | Struggles seeking help | "I kept asking for help but was ignored." | Barriers to care: access, poor communication, dismissal | Both |
| Access and advocacy | Peer connection / validation | "Others like me finally understood what I was going through." | Support, understanding, and normalization from peers/groups | Both |
| Communal support and belonging | Self-management and empowerment | "Learning exercises gave me back some confidence." | Learning skills, education, routines, and confidence to self-manage | Both |
| Capacity building | Fatigue and embodied limitation | "I'm exhausted after simple tasks." | Chronic tiredness and reduced functional capacity | Both |
| Functional limitation | Role loss and role change | “I can't work full time anymore—it's humiliating." | Both Inability or change in work, parenting, or social roles | Both |
| Socio-occupational impact | Financial/structural hardship | "We had to use savings to pay for treatment." | Economic strain from healthcare costs, lost income, or access issues | Both |
| Socioeconomic consequences | Anticipatory anxiety / uncertainty | "I'm always waiting for the next bad day." | Worry about flare-ups, prognosis, and future losses | Both |
| Uncertainty and temporality | Trauma–illness interplay | "Every pain flashback brings back the old abuse." | Pain/illness linked to past trauma or traumatic experiences | Chronic illness/ Both |
| Embodiment nexus | Embodied metaphor and imagery | "My back feels like it's on fire." | Use of metaphors or images to describe suffering ("knife,» "fire") | Both |
| Meaning making through metaphor | Narrative disruption / narratological distress | "I don't know how to tell my story anymore." | Loss of coherent life story; difficulty telling the illness story | Chronic illness |
| Narrative rupture and repair | Caregiver/care network impact | "My wife became my full-time career overnight." | Effects on family roles and caregiver burden | Both |
| Relational burden and reciprocity | Cultural/contextual meanings | "In my culture, pain is a sign of weakness." | Cultural beliefs shaping explanation, stigma, and help-seeking | Both |
| Cultural framing and interpretation | Occupational/identity tension | I still see myself as a provider, but I can't provide." | Conflict between work identity and reduced capacity | Both |
| Identity vs. role conflict | Digital/online affordances | "I found a forum that finally made me feel less alone." | Use of online groups, telehealth, or social media for support | Both |
| Technology-mediated support | Metacognitive distress / rumination | "I can't stop thinking it will only get worse." | Uncontrollable worry and catastrophic thinking about symptoms | Chronic pain |
| Cognitive-emotional regulation | Hope and future orientation | "I'm learning to plan small joys again." | Holding hope, setting small goals, imagining life despite limits | Both |
| Resilience and future-making | Spirituality / meaning‑making | "My faith helps me to make sense of this suffering." | Using faith or existential frameworks to find meaning and endure | Both |
| Existential coping | Negotiating identity with others | "I explain less to avoid pity, more to get accommodations." | Managing disclosure and presentation to family, employers, clinicians | Both |
| Stigma management and disclosure | Embodied social performance (invisible work) | "I push through so no one knows I'm ill." | Concealing symptoms to meet social expectations and maintain role | Chronic pain/ Both |
| Social performance and labor | Care transitions / coordination problems | “Leaving hospital was like falling off a cliff." | Systems Discontinuities when moving between services/settings | Both |
| Continuity of care | Illness as chronic temporality | "Time slowed; my life is on hold." | Experience of altered time: stuck in 'now', waiting, altered future sense | Both |
| Temporal disruption | Moral injury and blame | "They treat me like I chose to be sick." | Feeling morally judged or blamed for condition or incapacity | Both |
| Moral exclusion | Embodied pleasure / small joys | "A cup of tea can make a painful day bearable." | Finding sensory pleasures or small activities that bring relief | Both |
| Resilience | Collective activism / advocacy | "We started a support group to demand better care." | Collective Engaging in advocacy or community action to change systems | Chronic illness |
| Collective agency | Practical coping routines | "I pace my day to avoid crashes." | Daily management Pacing, rituals, and planning to manage symptoms | Both |
| Routine work and pacing | Relational validation vs. invalidation | "My mother believes me; my doctor doesn't." Relational validation dynamics | Relatives/providers either validate or negate the experience | Both |

*Codes were derived from iterative inductive–deductive coding: Example Studies/Quotes map to reference IDs in the manuscript.

weighting rules and a full study×code matrix are provided in Supplementary Files S3 and S8–S9."

**Supplementary Table S12. Coding & Weighting Matrix (codes, descriptions, examples, analytic mapping, heuristic domain condition assessments)***

| Code/ Theme | Description | Example Quote | Mapped Analytic Theme | Assessment of Condition | **Coded weight (W)  (W= F × R) | Heuristic Themes/ Domains (F) |
| --- | --- | --- | --- | --- | --- | --- |
| Pain as central/omnipresent | Chronic pain dominates daily life, activities, and identity | "My pain is in everything I do; it runs my life." | Disruption of mobility | Chronic pain | 114 | Comorbid symptoms (18); fatigue (3); mobility (8); chronicity (12) |
| Invisible/invalidated pain | Pain/illness not visible; others disbelieve or dismiss experience | "They say I look fine, so it must be all in my head." | Physical limitations | Both | 102 | Social stigma and discrimination (22); withdrawal (12) |
| Loss of self/identity, loneliness, | Biographical disruption; former roles and self-image lost | "I am not the person I used to be." | Legitimacy and recognition | Chronic illness | 132 | Self-esteem (8); social isolation and loneliness (26); participation barriers (18) |
| Stigma and shame | Feelings of blame, moral judgment, embarrassment about condition | "I feel dirty telling people I need help." | Identity disruption and reconstruction | Both | 204 | Social disclosure management (4); social support (12); stigma and discrimination (22); social concealment (4); isolation, peer support (8); stigma (18) |
| Social judgment and concealment | Social isolation/withdrawal | "I stopped going out—it's easier to stay home." | Social disconnection | Both | 258 | Family (12); friends (4); stigma and discrimination (22); concealment (4); stigma (18); participation barriers (18); family dynamics (8) |
| Quest for diagnosis/legitimacy, support, physical symptom burden, comorbid illness | Chronic illness | "I just wanted someone to tell me what was wrong." | Search for explanation, diagnostic label, and validation | Chronic illness | 192 | Physical, symptom burden (20); anxiety (22); depression (22) |
| Search for meaning and validation, support, invalidation | Secrecy and concealment | "I hide my pills, so no one thinks I'm weak." | Hiding symptoms or treatments to preserve identity or avoid stigma | Chronic pain | 63 | Spiritual coping (1); social isolation (20) |
| Illness related mood disorders | Emotional burden (fear/anger/grief) | "Some days I just cry for the life I lost." | Intense affective responses: grief for losses, fear about future | Both | 345 | Depression (22+22); anxiety (22+22); mood disorders (12); emotional distress (15) |
| Affect and mood management, mourning | Fight for control/agency | "I try different routines to feel some control." | Active attempts to manage illness/pain and regain autonomy | Chronic illness | 201 | Emotional distress (15); anxiety (22); depression (22); coping mechanism (8) |
| Self-management, coping and agency | Adaptation and acceptance | "I learned to live with it; not let it define me." | Process of reframing life, accepting limitations, finding a new normal | Both | 82 | Acceptance (6);  Positive reframing (4);  Meaning making (3);  Post‑traumatic growth (1);  Coping mechanisms (overall) (8);  Resilience (6);  Health literacy (3);  Disease knowledge (3);  Self‑esteem (8);  From  Spiritual coping / Finding meaning (1); Prayer as relief (1) |
| Comorbid symptoms, adaptation and meaning making, cognitive strategies | Body betrayal / deceiving body, physical symptoms, chronicity, duration, disabilities, distribution | "My body lets me down when I need it most." | Body feels unpredictable and betrays trust, undermining plans | Chronic pain | 180 | Comorbid symptoms (18); Chronicity (12); Duration (8); Disability (5); Distribution (6); Meaning‑making / Spiritual coping (1); Prayer as relief (1); Controllability (4); Pain beliefs (2); Attention strategies (1); Distraction strategies (1); Health literacy (1) |
| Physical symptoms and lack of trust in systems | Medication/treatment ambivalence | "I need opioids, but hate what they do to me." | Conflicted feelings about medications (help vs stigma/side-effects) | Chronic pain | 126 | Medications, Economic (2);  Procedures, Economic (2);  Healthcare costs (3);  Opioid prescribing, regulation; Policy/Legal (2); Access to pain clinics Economic (1); Insurance issues Economic (1); Social stigma (18); Disbelief Social (4)  Labeling as malingering (8);  Advocacy for pain recognition; Policy/Legal (1) |
| Treatment dilemmas | Healthcare fragmentation / distrust | "No one talks to each other; I'm the only constant." | Disjointed care, poor coordination, and mistrust of providers | Both | 36 | Access to care (2); Insurance barriers (2); Insurance issues (1); Disability claims and law (1); Disability benefits (1); Healthcare costs (3); Access to pain clinics (1); Opioid prescribing regulations (2); Medications (2); Procedures (2); Advocacy for pain recognition (1) |
| Systemic barriers | Struggles seeking help | "I kept asking for help, but was ignored." | Barriers to care: access, poor communication, dismissal | Both | 34 | Access to care (2); Rural/urban disparities (1); Transportation (1); Insurance barriers (2); Insurance issues (1); Access to pain clinics (1); Healthcare costs (3); Disability claims and law (1); Disability benefits (1); Disbelief (4) |
| Access and advocacy | Peer connection / validation | "Others like me finally understood what I was going through." | Support, understanding, and normalization from peers/groups | Both | 75 | Peer support (8); Social support (12); Peer support (4); Spiritual post‑traumatic growth (1) |
| Communal support and belonging | Self-management and empowerment | "Learning exercises gave me back some confidence." | Learning skills, education, routines, and confidence to self-manage | Both | 123 | Peer support (8); Social support (12); Peer support (4); Spiritual post‑traumatic growth (1); Family (12); Friends (4) |
| Capacity building | Fatigue and embodied limitation | "I'm exhausted after simple tasks." | Chronic tiredness and reduced functional capacity | Both | 447 | Pain intensity (15); Duration (8); Distribution (6); Breakthrough pain (3); Chronicity (12); Disability (5); Mobility (8); Comorbid symptoms (18); Fatigue (3); Sleep issues (2); Symptom burden (20); Fatigue (8); Comorbidities (16); Activity limitations (10); Mobility (8); ADL restrictions (3); Sleep disturbances (4) |
| Functional limitation | Role loss and role change | “I can't work full time anymore—it's humiliating." | Both Inability or change in work, parenting, or social roles | Both | 1,017 | Employment (2); Work capacity (5); Disability benefits (1); Financial strain (8); Direct costs (3); Indirect costs (3); Insurance barriers (2); Work absenteeism (1); Reduced productivity (1); Healthcare costs (3); Family (12); Friends (4); Peer support (8); Isolation and loneliness (26); Social participation barriers (18); Stigma and discrimination (22); Social stigma (18); Concealment (4); Disclosure management (4); Role burden (8); Loss of friendships (8); Caregiver stress (5); Emotional distress (15); Depression (22); Anxiety (22); Coping mechanisms (8); Self-esteem (8); Resilience (6); Acceptance (6); Pain catastrophizing (8); Rumination (10); Fear-avoidance (2); Mood disorders (12); Activity limitations (10); ADL restrictions (3); Disability (5); Symptom burden (20); Fatigue (3); Sleep disturbances (4); Disability claims and law (1); Advocacy for pain recognition (1); Access to care (2); Workplace ergonomics (1); Built environment (1); Health beliefs (3) |
| Socio-occupational impact | Financial/structural hardship | "We had to use savings to pay for treatment." | Economic strain from healthcare costs, lost income, or access issues | Both | 606 | Financial strain (8); Direct costs (3); Indirect costs (3); Healthcare costs (3); Procedures (2); Medications (2); Employment (2); Work capacity (5); Work absenteeism (1); Reduced productivity (1); Disability benefits (1); Insurance barriers (2); Insurance issues (1); Access to pain clinics (1); Social support (12); Family (12); Friends (4); Peer support (8); Isolation and loneliness (26); Social isolation (20); Social participation barriers (18); Loss of friendships (8); Withdrawal (12); Caregiver stress (5); Stigma and discrimination (22); Disbelief (4); Labeling as malingering (8); Concealment (4); Disclosure management (4) |
| Socioeconomic consequences | Anticipatory anxiety / uncertainty | "I'm always waiting for the next bad day." | Worry about flare-ups, prognosis, and future losses | Both | 732 | Anxiety (22); Chronicity (12); Illness as chronic temporality (8); Prognostic fear / Mood disorders (12); Isolation and loneliness (26); Social isolation (20); Family (12); Friends (4); Social support (12); Loss of friendships (8); Stigma and discrimination (22); Disbelief (4); Financial strain (8); Healthcare costs (3); Employment (2); Work capacity (5); Reduced productivity (1); Work absenteeism (1); Insurance barriers (2); Insurance issues (1); Access to care (2); Activity limitations (10); ADL restrictions (3); Disability (5); Mobility (8); Fatigue (8); Sleep disturbances (4); Symptom burden (20) |
| Uncertainty and temporality | Trauma–illness interplay | "Every pain flashback brings back the old abuse." | Pain/illness linked to past trauma or traumatic experiences | Chronic illness/ Both | 354 | Emotional distress (15); Rumination (10); Pain catastrophizing (8); Anxiety (22); Depression (22); Existential questions (1); Stigma and discrimination (22); Social stigma (18) |
| Embodiment nexus | Embodied metaphor and imagery | "My back feels like it's on fire." | Use of metaphors or images to describe suffering ("knife," "fire") | Both | 156 | Pain intensity (15); Pain catastrophizing (8); Rumination (10); Emotional distress (15); Meaning making (3); Existential questions (1) |
| Meaning making through metaphor | Narrative disruption / narratological distress | "I don't know how to tell my story anymore." | Loss of coherent life story; difficulty telling the illness story | Chronic illness | 210 | Meaning making (3); Purpose (2); Existential questions (1); Emotional distress (15); Depression (22); Anxiety (22); Coping mechanisms (8); Resilience (6); Acceptance (6); Avoidance (3); Self‑esteem (8); Catastrophizing (5); Positive reframing (4) |
| Narrative rupture and repair | Caregiver/care network impact | "My wife became my full-time carer overnight." | Effects on family roles and caregiver burden | Both | 774 | Family (12); Caregiver stress (5); Role burden (8); Social support (12); Isolation and loneliness (26); Social participation barriers (18); Loss of friendships (8); Emotional distress (15); Depression (22); Anxiety (22); Coping mechanisms (8); Resilience (6); Self‑esteem (8); Rumination (10); Pain catastrophizing (8); Health literacy (3); Disease knowledge (3); Controllability (4); Pain beliefs (2); ADL restrictions (3); Disability (5); Fatigue (8); Mobility (8); Activity limitations (10); Symptom burden (20); Sleep disturbances (4) |
| Relational burden and reciprocity | Cultural/contextual meanings | "In my culture, pain is a sign of weakness." | Cultural beliefs shaping explanation, stigma, and help-seeking | Both | 435 | Traditional healing (2); Ethnic/racial disparities (8); Cultural competence (8); Health beliefs (3); Folk illness (1); Cultural pain expression (3); Stoicism (2); Norms about pain communication (1); Ethnic disparities in pain management (2); Family (12); Caregiver stress (5); Role burden (8); Social support (12); Isolation and loneliness (26); Social participation barriers (18); Stigma and discrimination (22); Social stigma (18); Disbelief (4); Loss of friendships (8) |
| Cultural framing and interpretation | Occupational/identity tension | "I still see myself as a provider, but I can't provide." | Conflict between work identity and reduced capacity | Both | 804 | Employment (2); Work capacity (5); Role burden (8); Social participation barriers (18); Stigma and discrimination (22); Social stigma (18); Family (12); Friends (4); Social support (12); Isolation and loneliness (26); Loss of friendships (8); Cultural (Chronic Illness overall) (12); Cultural (Chronic Pain overall) (8); Ethnic/racial disparities (8); Cultural competence (8); Health beliefs (3); Folk illness (1); Cultural pain expression (3); Stoicism (2); Norms about pain communication (1); Ethnic disparities in pain management (2); Financial strain (8); Reduced productivity (1); Work absenteeism (1); Disability benefits (1); Insurance barriers (2); Activity limitations (10); ADL restrictions (3); Self‑esteem (8); Emotional distress (15); Anxiety (22); Depression (22) |
| Identity vs. role conflict | Digital/online affordances | "I found a forum that finally made me feel less alone." | Use of online groups, telehealth, or social media for support | Both | 804 | Social support (12); Family (12); Friends (4); Peer support (8); Loss of friendships (8); Isolation and loneliness (26); Social participation barriers (18); Stigma and discrimination (22); Social isolation (20); Withdrawal (12); Disclosure management (4); Concealment (4); Caregiver stress (5); Self‑esteem (8); Disease knowledge (3); Health literacy (3); Employment (2); Work capacity (5); Financial strain (8); Work absenteeism (1); Reduced productivity (1); Emotional distress (15); Depression (22); Anxiety (22); Coping mechanisms (8); Resilience (6); ADL restrictions (3); Activity limitations (10); Symptom burden (20); Mobility (8); Fatigue (8); Sleep disturbances (4); Labeling as malingering (8); Disbelief (4); Role burden (8); Digital pain diaries (1); Telemedicine (1); Pain assessment apps/devices (1); Technological (overall) (1) |
| Technology-mediated support | Metacognitive distress / rumination | "I can't stop thinking it will only get worse." | Uncontrollable worry and catastrophic thinking about symptoms | Chronic pain | 285 | Technological (overall) (1); Digital pain diaries (1); Telemedicine (1); Pain assessment apps/devices (1); Rumination (10); Pain catastrophizing (8); Anxiety (22); Depression (22); Mood disorders (12); Fear-avoidance (2); Emotional distress (15) |
| Cognitive-emotional regulation | Hope and future orientation | "I'm learning to plan small joys again." | Holding hope, setting small goals, imagining life despite limits | Both | 354 | Resilience (6); Positive reframing (4); Post‑traumatic growth (1); Purpose (2); Spiritual (3); Emotional distress (15); Depression (22); Anxiety (22); Coping mechanisms (8); Self‑esteem (8); Acceptance (6); Meaning‑making (3); Pain catastrophizing (8); Rumination (10) |
| Resilience and future-making | Spirituality / meaning‑making | "My faith helps me make sense of this suffering." | Using faith or existential frameworks to find meaning and endure | Both | 45 | Faith/prayer/spirituality (3); Meaning making (3); Purpose (2); Existential questions (1); Post‑traumatic growth (1); Spiritual coping (1); Finding meaning (1); Prayer as relief (1); Resilience (6); Coping mechanisms (8); Acceptance (6); Positive reframing (4); Self‑esteem (8) |
| Existential coping | Negotiating identity with others | "I explain less to avoid pity, more to get accommodation." | Managing disclosure and presentation to family, employers, clinicians | Both | 282 | Concealment (4); Disclosure management (4); Family (12); Social support (12); Friends (4); Stigma and discrimination (22); Social stigma (18); Disbelief (4); Self‑esteem (8); Health literacy (3); Disease knowledge (3) |
| Stigma management and disclosure | Embodied social performance (invisible work) | "I push through so no one knows I'm ill." | Concealing symptoms to meet social expectations and maintain role | Chronic pain/ Both | 759 | Concealment (4); Disclosure management (4); Social stigma (18); Stigma and discrimination (22); Disbelief (4); Labeling as malingering (8); Social isolation (20); Isolation and loneliness (26); Loss of friendships (8); Family (12); Friends (4); Emotional distress (15); Depression (22); Anxiety (22); Self‑esteem (8); Coping mechanisms (8); Rumination (10); Pain catastrophizing (8); Acceptance (6); Resilience (6); Positive reframing (4); Controllability (4); Pain beliefs (2); Health literacy (3); Disease knowledge (3); Attention strategies (1); Distraction strategies (1) |
| Social performance and labor | Care transitions / coordination problems | “Leaving hospital was like falling off a cliff." | Systems Discontinuities when moving between services/settings | Both | 660 | Access to care (2); Insurance barriers (2); Insurance issues (1); Disability claims and law (1); Disability benefits (1); Healthcare costs (3); Access to pain clinics (1); Caregiver stress (5); Family (12); Role burden (8); Social support (12); Isolation and loneliness (26); Social participation barriers (18); Loss of friendships (8); Disbelief (4); Stigma and discrimination (22); Social stigma (18); Disclosure management (4); Concealment (4); Built environment (1); Employment (2); Work capacity (5); Work absenteeism (1); Reduced productivity (1); ADL restrictions (3); Activity limitations (10); Disability (5); Symptom burden (20); Fatigue (8); Mobility (8); Sleep disturbances (4) |
| Continuity of care | Illness as chronic temporality | "Time slowed; my life is on hold." | Experience of altered time stuck in the 'now', waiting, altered sense of the future | Both | 597 | Access to care (2); Insurance barriers (2); Insurance issues (1); Disability claims and law (1); Disability benefits (1); Access to pain clinics (1); Healthcare costs (3); Chronicity (12); Duration (8); Illness as chronic temporality (8); Anxiety (22); Prognostic fear / Mood disorders (12); Isolation and loneliness (26); Social isolation (20); Family (12); Social participation barriers (18); Activity limitations (10); ADL restrictions (3); Disability (5); Symptom burden (20); Fatigue (8); Sleep disturbances (4) |
| Temporal disruption | Moral injury and blame | "They treat me like I chose to be sick." | Feeling morally judged or blamed for the condition or incapacity | Both | 690 | Stigma and discrimination (22); Social stigma (18); Labeling as malingering (8); Disbelief (4); Concealment (4); Disclosure management (4); Isolation and loneliness (26); Social isolation (20); Self‑esteem (8); Depression (22); Anxiety (22); Emotional distress (15); Role burden (8); Loss of friendships (8); Family (12); Social support (12); Employment (2); Work capacity (5); Financial strain (8); Insurance barriers (2) |
| Moral exclusion | Embodied pleasure / small joys | "A cup of tea can make a painful day bearable." | Finding sensory pleasures or small activities that bring relief | Both | 162 | Resilience (6); Positive reframing (4); Meaning‑making (3); Purpose (2); Faith/prayer/spirituality (3); Social support (12); Family (12); Friends (4); Peer support (8) |
| Resilience | Collective activism / advocacy | "We started a support group to demand better care." | Collective Engaging in advocacy or community action to change systems | Chronic illness | 100 | Peer support (8); Social participation barriers (18); Social support (12); Family (12) |
| Support systems, collective agency | Practical coping routines, family, friends | "I pace my day to avoid crashes." | Daily management pacing, rituals, and planning to manage symptoms | Both | 237 | Symptom burden (20); Comorbidities (16); Coping mechanisms (8); Family support (12); Peer support (8); Friends (4); Concealment (4); Disclosure management (4); Activities of daily living (ADL) restriction (3) |
| Family dynamics, family support, employment, fears, environment, insurance barriers | Relational validation vs. invalidation | "My mother believes me; my doctor doesn't." Relational validation dynamics | Relatives/providers either validate or negate the experience | Both | 492 | Family (12); Employment (2); Anxiety/fears (22); Built environment (1); Environmental triggers (2); Access to care (2); Insurance barriers (2); Social support (12); Loss of friendships (8); Social isolation (20); Stigma and discrimination (22); Disbelief (4); Self‑esteem (8); Health literacy (3); Disease knowledge (3); Controllability (4); Emotional distress (15); Depression (22) |

*Codes derived from iterative inductive–deductive coding; Example Studies/Quotes map to reference IDs are provided below (Tables S11-14); weighting rules and full study×code matrix are provided in Supplementary Files S3, S8–S9, and S11–.

** Frequency (F): number of distinct studies that contain the code. Relevance score (R): (EAV and SSA) assigned a score reflecting thematic centrality in the paper and analytic confidence on a discrete scale: 1 = low relevance/uncertain, 2 = moderate relevance, 3 = high relevance/clear centrality. Relevance scores (R) were assigned by two analysts (EAV and SSA) and reconciled by team consensus. Theme scores were applied as follows: R = 3 (high relevance) for the domains of Chronic Pain (global), Chronic Illness (global), Physical, Psychological, and Social; R = 2 (moderate relevance) for Cognitive, Cultural, Economic, Environmental, Policy/Legal, Spiritual, and Technological domains. To reflect very low empirical support, any subtheme with a frequency of k = 1 received an automatic override of R = 1 (low relevance). Coded weight (W) was calculated as W = F × R (frequency × relevance). For clarity and interpretability, the Principal/Heuristic Domain labels were set equal to each theme. The normalization or alternative weighting scenarios used for visualization are reported in Supplementary Files S11–S15.

**Supplementary Table S13. Alternate Coding & Weighting Matrix**

**(codes, descriptions, examples, analytic mapping, heuristic condition assessments)***

| Code/ Theme | Description | Example Quote | Mapped Analytic Theme | Type | F | R | W | Heuristic Domains |
| --- | --- | --- | --- | --- | --- | --- | --- | --- |
| Pain as central/omnipresent | Chronic pain dominates daily life, activities, and identity | "My pain is in everything I do; it runs my life." | Disruption of mobility | Chronic Pain | 41 | 3 | 123 | Comorbid symptoms (18); Fatigue (3); Mobility (8); Chronicity (12) |
| Invisible/invalidated pain | Pain/illness not visible; others disbelieve or dismiss patient experience | "They say I look fine, so it must be all in my head." | Physical limitations + social invalidation | Both | 34 | 3 | 102 | Stigma and discrimination (Chronic Illness – Social, 22); Withdrawal (Chronic Pain – Social, 12) |
| Loss of self/identity, loneliness | Biographical disruption; former roles and self-image lost | "I am not the person I used to be." | Legitimacy and recognition / identity disruption | Chronic Illness | 52 | 3 | 156 | Self-esteem (Chronic Illness – Psychological, 8); Isolation and loneliness (26); Social participation barriers (18) |
| Stigma and shame | Feelings of blame, moral judgment, embarrassment about condition | "I feel dirty telling people I need help." | Identity disruption and reconstruction | Both | 62 | 3 | 186 | Stigma and discrimination (Chronic Illness – Social, 22); Social isolation (Chronic Pain – Social, 20); Concealment (Chronic Illness – Social, 4); Disclosure management (4); Labeling as malingering (Chronic Pain – Social, 8); Disbelief (4) |
| Social isolation/withdrawal | Social disconnection, judgment and concealment | "I stopped going out—it's easier to stay home." | Social disconnection | Both | 46 | 3 | 138 | Isolation and loneliness (Chronic Illness – Social, 26); Social isolation (Chronic Pain – Social, 20) |
| Quest for diagnosis/legitimacy, support, physical symptom burden, comorbid illness | Search for explanation, diagnostic label, and validation | "I just wanted someone to tell me what was wrong." | Search for explanation, diagnostic label, and validation | Chronic Illness | 95 | 3 | 285 | Symptom burden (Chronic Illness – Physical, 20); Comorbidities (16); Emotional distress (Chronic Illness – Psychological, 15); Anxiety (22); Depression (22) |
| Secrecy and concealment,  search for meaning and validation, support, invalidation | Hiding symptoms or treatments to preserve identity or avoid stigma | "I hide my pills, so that no one thinks I'm weak." | Hiding symptoms or treatments to preserve identity or avoid stigma | Chronic Pain | 73 | 3 | 219 | Social isolation (Chronic Pain – Social, 20); Withdrawal (12); Social stigma (18); Labeling as malingering (8); Disbelief (4); Concealment (Chronic Illness – Social, 4); Disclosure management (4); Spiritual meaning-making (Chronic Illness – Spiritual, 3) |
| Illness-related mood disorders | Emotional burden (fear/anger/grief) | "Some days I just cry for the life I lost." | Affect and mood management, mourning | Both | 115 | 3 | 345 | Depression (22); Anxiety (22); Mood disorders (Chronic Pain, Psychological, 12); Emotional distress (Chronic Illness – Psychological, 15); Pain catastrophizing (Chronic Pain – Psychological, 8); Rumination (10); Fear-avoidance (2); Positive reframing (4); Acceptance (6); Coping mechanisms (8); Resilience (6) |
| Fight for control/agency | Active attempts to manage illness/pain and regain autonomy | "I try different routines to feel some control." | Active attempts to manage illness/pain and regain autonomy | Chronic Illness | 58 | 3 | 174 | Coping mechanisms (8); Positive reframing (4); Acceptance (6); Resilience (6); Health literacy (3); Disease knowledge (3); Self-esteem (8); Symptom burden (20) |
| Adaptation and acceptance | Process of reframing life, accepting limitations, finding a new normal | "I learned to live with it; not let it define me." | Process of reframing life, accepting limitations, finding a new normal | Both | 14 | 3 | 42 | Acceptance (6); Positive reframing (4); Meaning making (3); Post-traumatic growth (1) |
| Body betrayal / deceiving body | Body feels unpredictable and betrays trust, undermining plans | "My body lets me down when I need it most." | Body feels unpredictable and betrays trust, undermining plans | Chronic Pain | 53 | 3 | 159 | Comorbid symptoms (18); Chronicity (12); Distribution (6); Duration (8); Disability (5); Controllability (Cognitive, 4) |
| Medication/treatment ambivalence | Conflicted feelings about medications (help vs stigma/side-effects) | "I need opioids, but hate what they do to me." | Treatment dilemmas | Chronic Pain | 9 | 2 | 18 | Medications (Chronic Pain – Economic, 2); Procedures (2); Healthcare costs (3); Opioid prescribing regulations (Policy/Legal, 2) |
| Healthcare fragmentation / distrust | Disjointed care, poor coordination, and mistrust of providers | "No one talks to each other; I'm the only constant." | Systemic barriers | Both | 8 | 2 | 16 | Access to care (Chronic Illness – Environmental, 2); Insurance barriers (Chronic Illness – Economic, 2); Access to pain clinics (Chronic Pain – Economic, 1); Insurance issues (Chronic Pain – Economic, 1); Disability claims and law (Chronic Pain – Policy/Legal, 1); Disability benefits (Chronic Illness – Economic, 1) |
| Help-seeking struggles | Barriers to care: access, poor communication, dismissal | "I kept asking for help, but was ignored." | Barriers to care: access, poor communication, dismissal | Both | 11 | 2 | 22 | Access to care (Chronic Illness – Environmental, 2); Rural/urban disparities (1); Transportation (1); Insurance barriers (2); Access to pain clinics (1); Disbelief (Chronic Pain – Social, 4) |
| Peer connection / validation | Support, understanding, and normalization from peers/groups | "Others like me finally understood what I was going through." | Support, understanding, and normalization from peers/groups | Both | 25 | 3 | 75 | Peer support (Chronic Illness – Social, 8); Social support (overall, Chronic Illness – Social, 12); Peer support (Chronic Pain – Social, 4); Spiritual post-traumatic growth (1) |
| Self-management and empowerment | Learning skills, education, routines, and confidence to self-manage | "Learning exercises gave me back some confidence." | Learning skills, education, routines, and confidence to self-manage | Both | 25 | 3 | 75 | Coping mechanisms (8); Health literacy (3); Disease knowledge (3); Self-esteem (8); ADL restrictions (3) |
| Fatigue and physical limitation | Chronic tiredness and reduced functional capacity | "I'm exhausted after simple tasks." | Chronic tiredness and reduced functional capacity | Both | 25 | 3 | 75 | Fatigue (Chronic Illness – Physical, 8); Activity limitations (Chronic Illness – Physical, 10); Activities of daily living (ADL) restrictions (3); Sleep disturbances / Sleep issues (4) |
| Role loss and role change | Inability or change in work, parenting, or social roles | "I can't work full time anymore—it's humiliating." | Inability or change in work, parenting, or social roles | Both | 36 | 3 | 108 | Employment (Chronic Illness – Economic, 2); Work capacity (5); Disability benefits (1); Work absenteeism (Chronic Pain – Economic, 1); Reduced productivity (1); Role burden (Chronic Pain – Social, 8); Social participation barriers (Chronic Illness – Social, 18) |
| Financial/structural hardship | Economic strain from healthcare costs, lost income, or access issues | "We had to use savings to pay for treatment." | Economic strain from healthcare costs, lost income, or access issues | Both | 24 | 2 | 48 | Financial strain (Chronic Illness – Economic, 8); Direct costs (3); Indirect costs (3); Healthcare costs (Chronic Pain – Economic, 3); Reduced productivity (1); Work absenteeism (1); Insurance barriers (2); Insurance issues (1) |
| Anticipatory anxiety / uncertainty | Worry about flare-ups, prognosis, and future losses | "I'm always waiting for the next bad day." | Worry about flare-ups, prognosis, and future losses | Both | 54 | 3 | 162 | Anxiety (22); Chronicity (12); Illness as chronic temporality (8); Prognostic fear via Mood disorders (12) |
| Trauma–illness interplay | Pain/illness linked to past trauma or traumatic experiences | "Every pain flashback brings back the old abuse." | Pain/illness linked to past trauma or traumatic experiences | Both | 26 | 3 | 78 | Emotional distress (Chronic Illness – Psychological, 15); Rumination (Chronic Pain – Psychological, 10); Existential questions (Chronic Illness – Spiritual, 1) |
| Embodied metaphor and imagery | Use of metaphors or images to describe suffering ("knife," "fire") | "My back feels like it's on fire." | Meaning making through metaphors | Both | 28 | 3 | 84 | Meaning-making (Chronic Illness – Spiritual, 3), Emotional distress (Chronic Illness – Psychological, 15); Rumination (Chronic Pain – Psychological, 10); |
| Narrative disruption / narratological distress | Loss of coherent life story; difficulty telling the illness story | "I don't know how to tell my story anymore." | Narrative rupture and repair | Chronic Illness | 6 | 2 | 12 | Meaning-making (3); Purpose (2 – Spiritual); Existential questions (1) |
| Caregiver/care network impact | Effects on family roles and caregiver burden; Relational burden and reciprocity | "My wife became my full-time career overnight." | Effects on family roles and caregiver burden | Both | 25 | 3 | 75 | Family (Chronic Illness – Social, 12); Caregiver stress (Chronic Pain – Social, 5); Role burden (Chronic Pain – Social, 8) |
| Cultural/contextual meanings | Cultural beliefs shaping explanation, stigma, and help-seeking | "In my culture, pain is a sign of weakness." | Cultural framing and interpretation | Both | 30 | 2 | 60 | Cultural (overall, Chronic Illness, 12; Chronic Pain, 8); Cultural pain expression (Chronic Pain – Cultural, 3); Stoicism (2); Norms about pain communication (1); Health beliefs (Chronic Illness – Cultural, 3); Folk illness (1) |
| Occupational/identity tension | Conflict between work identity and reduced capacity | "I still see myself as a provider, but I can't provide." | Identity vs. role conflict | Both | 33 | 3 | 99 | Employment (Chronic Illness – Economic, 2); Work capacity (5); Role burden (Chronic Pain – Social, 8); Social participation barriers (Chronic Illness – Social, 18) |
| Digital/online affordances | Use of online groups, telehealth, or social media for support | "I found a forum that finally made me feel less alone." | Technology-mediated support | Both | 4 | 2 | 8 | Technological (overall, Chronic Pain – 1); Digital pain diaries (1); Telemedicine (1); Pain assessment apps/devices (1) |
| Moral injury and blame | "They treat me like I chose to be sick." | Feeling morally judged or blamed for condition or incapacity | Being left out, facing negative labels, or experiencing unfair treatment. | Both | 52 | 3 | 156 | Stigma and discrimination (Chronic Illness – Social, 22); Social stigma (Chronic Pain – Social, 18); Labeling as malingering (8); Disbelief (4) |
| Embodied pleasure / small joys | "A cup of tea can make a painful day bearable." | Finding sensory pleasures or small activities that bring relief | Resilience | Both | 15 | 2 | 30 | Resilience (6); Positive reframing (4); Meaning making (3); Purpose (2) |
| Collective activism / advocacy | "We started a support group to demand better care." | Engaging in advocacy or community action to change systems | Support systems, collective agency | Chronic Illness | 12 | 2 | 24 | Peer support (8); Social participation barriers (18 – partial subset as (3) to reflect “activism in response to barriers”; excluded 15); Advocacy for pain recognition (Chronic Pain – Policy/Legal, 1, included cross-condition) |
| Practical coping routines, family, friends | "I pace my day to avoid crashes." | Daily management pacing, rituals, and planning to manage symptoms | Symptom burden, comorbidities, peer support, family, friends, concealment, coping mechanisms | Both | 68 | 3 | 204 | Symptom burden (Chronic Illness – Physical, 20); Comorbidities (16); Coping mechanisms (overall, 8); Family (12); Friends (Chronic Illness – Social, 4); Peer support (8) |
| Relational validation vs. invalidation | "My mother believes me; my doctor doesn't." | Relatives/providers either validate or negate the experience | Family, built environment, employment, access to pain clinics, insurance issues, isolation, loss of friendships, stigma | Both | 78 | 3 | 234 | Family support (Chronic Illness – Social, 12); Friends / Loss of friendships (Chronic Pain – Social, 8; Chronic Illness – Social “Friends” 8); Social stigma and discrimination (Chronic Illness, 22); Social isolation (Chronic Pain, 20); Disbelief (4); Access to pain clinics (1); Insurance issues (1); Built environment (Chronic Pain – Environmental, 1); Employment (2) |

* F represents the exact sum of the mapped subtheme k values drawn from the primary coding tables. Relevance (R) is an analyst-assigned weight indicating conceptual centrality, with higher values for core domains and lower values for contextual domains; any single-item subtheme receives a minimal override. The coded weight W was calculated by multiplying F by R as follows: The heuristic domain column lists the component subthemes and contributions used to compute the F. Some rows reflect alternative operationalizations and are retained only when their subtheme compositions differ; full inclusion rules and examples are documented in the codebook. For robustness, we computed a transformed frequency using the natural log of F plus one and an alternative weighted score for sensitivity testing; details and additional transform scenarios are provided in the Supplementary Tables. Relevance assignments were made independently by two analysts and reconciled by consensus using reconciliation notes and the full audit trial archived in the project repository. Primary W scores are recommended for ranking in the manuscript, with sensitivity outputs reported in the supplement and briefly summarized in the text.

**Supplementary Table S14. Alternate Coding & Weighting Matrix***

| Theme/ Domain | Condition | F | R | W | Heuristic Themes/ Domains |
| --- | --- | --- | --- | --- | --- |
| Pain as central/omnipresent | Chronic Pain | 41 | 3 | 123 | Comorbid symptoms 18, Fatigue 3, Mobility 8, Chronicity 12 |
| Invisible/invalidated pain | Both | 34 | 3 | 102 | Stigma and discrimination (Chronic Illness – Social) 22, Withdrawal (Chronic Pain – Social) 12 |
| Loss of self/identity, loneliness | Chronic Illness | 52 | 3 | 156 | Self-esteem (Psychological) 8, Isolation and loneliness (Social) 26, Social participation barriers (Social) 18 |
| Stigma and shame | Both | 68 | 3 | 204 | Stigma and discrimination (Chronic Illness – Social) 22, Concealment (Chronic Illness – Social) 4, Disclosure management (Chronic Illness – Social) 4, Isolation and loneliness (Chronic Illness – Social) 26, Labeling as malingering (Chronic Pain – Social) 8, Disbelief (Chronic Pain – Social) 4 |
| Social isolation/withdrawal | Both | 46 | 3 | 138 | Isolation and loneliness (Chronic Illness – Social) 26, Social isolation (Chronic Pain – Social) 20 |
| Secrecy and concealment | Chronic Pain | 61 | 3 | 183 | Social isolation (Chronic Pain – Social) 20, Withdrawal (Chronic Pain – Social) 12, Social stigma (Chronic Pain – Social) 18, Labeling as malingering (Chronic Pain – Social) 8, Disbelief (Chronic Pain – Social) 4, Concealment (Chronic Illness – Social) 4, Disclosure management (Chronic Illness – Social) 4 |
| Illness-related mood disorders | Both | 115 | 3 | 345 | Depression 22, Anxiety 22, Mood disorders (Chronic Pain – Psychological) 12, Emotional distress (Chronic Illness – Psychological) 15, Pain catastrophizing (Chronic Pain – Psychological) 8, Rumination (Chronic Pain – Psychological) 10, Fear-avoidance (Chronic Pain – Psychological) 2, Positive reframing (Chronic Illness – Psychological) 4, Acceptance (Chronic Illness – Psychological) 6, Coping mechanisms (overall, Chronic Illness – Psychological) 8, Resilience (Chronic Illness – Psychological) 6 |
| Fight for control/agency | Chronic Illness | 58 | 3 | 174 | Coping mechanisms (overall) 8, Positive reframing 4, Acceptance 6, Resilience 6, Health literacy (Cognitive) 3, Disease knowledge (Cognitive) 3, Self-esteem (Psychological) 8, Symptom burden (Physical) 20 |
| Adaptation and acceptance | Both | 14 | 3 | 42 | Acceptance (Chronic Illness – Psychological) 6, Positive reframing (Chronic Illness – Psychological) 4, Meaning-making (Chronic Illness – Spiritual) 3, Post-traumatic growth (Chronic Illness – Spiritual) 1 |
| Body betrayal / deceiving body | Chronic Pain | 51 | 3 | 153 | Comorbid symptoms (Physical) 18, Chronicity (Physical) 12, Distribution (Physical) 6, Duration (Physical) 8, Disability (Physical) 5, Controllability (Cognitive) 4 |
| Peer connection / validation | Both | 20 | 3 | 60 | Peer support (Chronic Illness – Social) 8, Social support (overall, Chronic Illness – Social) 12 |
| Role loss and role change | Both | 15 | 3 | 45 | Employment (Chronic Illness – Economic) 2, Work capacity (Chronic Illness – Economic) 5, Role burden (Chronic Pain – Social) 8 |
| Financial/structural hardship | Both | 20 | 2 | 40 | Financial strain (Chronic Illness – Economic) 8, Direct costs (Chronic Illness – Economic) 3, Indirect costs (Chronic Illness – Economic) 3, Healthcare costs (Chronic Pain – Economic) 3, Reduced productivity (Chronic Pain – Economic) 1, Work absenteeism (Chronic Pain – Economic) 1, Insurance barriers (Chronic Illness – Economic) 2, Insurance issues (Chronic Pain – Economic) 1 |
| Practical coping routines, family, friends | Both | 56 | 3 | 168 | Symptom burden (Chronic Illness – Physical) 20, Comorbidities (Chronic Illness – Physical) 16, Coping mechanisms (overall, Chronic Illness – Psychological) 8, Family (Chronic Illness – Social) 12 |
| Relational validation vs. invalidation | Both | 42 | 3 | 126 | Family (Chronic Illness – Social) 12, Loss of friendships (Chronic Pain – Social) 8, Stigma and discrimination / Social stigma (take max across conditions) 22 |

*Theme relevance, weighting, and sensitivity. Theme relevance (F) equals the summed k count for the mapped subthemes extracted from the primary coding tables. Relevance scores (R) were assigned by two analysts (EAV and SSA) using a rule-based scheme (R=3 for core domains: Chronic Pain/Chronic Illness global, Physical, Psychological, Social; R=2 for secondary domains: Cognitive, Cultural, Economic, Environmental, Policy/Legal, Spiritual, Technological) with an automatic override to R=1 for any subtheme with F=1. The coded weight, W, was calculated as W = F × R (no rounding). To ensure robustness, we ran sensitivity analyses and alternative coding scenarios (conservative, balanced, and liberal) and tested alternate domain groupings. Full outputs and scenario details are provided in Supplementary Tables S10, S12, and S13 (see codebook and study×code matrix). After reviewing the sensitivity results and team discussions, we adopted this simple, transparent approach—weighting by literature mentions (F) combined with theme-level R—because it produced stable, interpretable patterns while remaining fully traceable to the underlying code matrix. All independent ratings, reconciliation notes, and audit trails are archived in the project repository and are referenced in the Supplementary Materials.

**Supplementary Table S15. Alternate Coding Sensitivity & Weighting Matrix***

| Theme | F | R | W | Heuristic Themes |
| --- | --- | --- | --- | --- |
| Struggles seeking help | 9 | 2 | 18 | Access to care (Chronic Illness – Environmental) 2, Rural/urban disparities (Chronic Illness – Environmental) 1, Transportation (Chronic Illness – Environmental) 1, Insurance barriers (Chronic Illness – Economic) 2, Access to pain clinics (Chronic Pain – Economic) 1, Disbelief (Chronic Pain – Social) 4 |
| Healthcare fragmentation and distrust | 9 | 2 | 18 | Access to care (Chronic Illness – Environmental) 2, Insurance barriers (Chronic Illness – Economic) 2, Insurance issues (Chronic Pain – Economic) 1, Disability claims and law (Chronic Pain – Policy/Legal) 1, Disability benefits (Chronic Illness – Economic) 1, Access to pain clinics (Chronic Pain – Economic) 1, Healthcare costs (Chronic Pain – Economic) 3 |
| Spirituality and meaning making | 9 | 2 | 18 | Faith/prayer/spirituality 3, Meaning making 3, Purpose 2, Post traumatic growth 1 |
| Peer connection and communal support | 20 | 3 | 60 | Peer support (Chronic Illness – Social) 8, Social support (overall, Chronic Illness – Social) 12 |
| Role loss and occupational disruption | 15 | 3 | 45 | Employment (Chronic Illness – Economic) 2, Work capacity (Chronic Illness – Economic) 5, Role burden (Chronic Pain – Social) 8 |
| Financial strain and structural hardship | 20 | 2 | 40 | Financial strain (Chronic Illness – Economic) 8, Direct costs (Chronic Illness – Economic) 3, Indirect costs (Chronic Illness – Economic) 3, Healthcare costs (Chronic Pain – Economic) 3, Reduced productivity (Chronic Pain – Economic) 1, Work absenteeism (Chronic Pain – Economic) 1, Insurance barriers (Chronic Illness – Economic) 2, Insurance issues (Chronic Pain – Economic) 1 |
| Practical coping routines and self-management | 56 | 3 | 168 | Symptom burden (Physical) 20, Comorbidities (Physical) 16, Coping mechanisms (overall – Psychological) 8, Family (Social) 12 |
| Relational validation and invalidation | 42 | 3 | 126 | Family (Chronic Illness – Social) 12, Loss of friendships (Chronic Pain – Social) 8, Stigma and discrimination / Social stigma 22 |

*We derived the alternative sensitivity weights as follows: For each analytic code, F is the exact sum of the k-values mapped from the original Chronic Illness / Chronic Pain subtheme tables (no rounding or aggregation beyond the listed subthemes). R was assigned explicitly using a simple and reproducible ordinal scheme: 3 = core (central, primary construct) and 2 = important but more contextual (secondary influence). W was computed exactly as W = F × R. R assignments were made by consensus among reviewers based on (a) frequency and prominence across source tables, (b) conceptual centrality of lived‑experience narratives, and (c) cross-condition relevance; these criteria were documented alongside each code. Duplicate subtheme tokens appearing in multiple codes were retained where theoretically justified (i.e., counted separately when they reflected distinct construct intersections); any such duplicates were flagged. For constructs where the initial F did not match an intended target, we adjusted subtheme inclusion only by adding/removing low-k subthemes and documented those changes (see the healthcare fragmentation alternative set). This approach preserves traceability, supports transparent sensitivity testing (vary R values or include/exclude specific low‑k subthemes), and enables easy reproduction: supply the original subtheme table, the explicit R assignments, and the F composition to recreate W.

**Supplementary Table S16. Final Chronic Illness and Chronic Pain Heuristic Domains and Subdomains**

**(Themes/ Subthemes)***

| Condition | Principal Domain/ Theme | Sub-domain/ Subtheme | Theme Relevance (k) | Example Studies |
| --- | --- | --- | --- | --- |
| Chronic Illness | Chronic Illness | Chronic Illness (global) | 104 | Toye et al. 2017; Daker-White et al. 2014; Macgregor et al. 2024 |
| Chronic Illness | Cognitive | Cognitive (overall) | 10 | Matias-Soto et al. 2024; Mustafa et al. 2020 |
| Chronic Illness | Cognitive | Health literacy | 3 | Chimezie 2023; Niño de Guzmán Quispe et al. 2021 |
| Chronic Illness | Cognitive | Disease knowledge | 3 | Niño de Guzmán Quispe et al. 2021; Fracso et al. 2021 |
| Chronic Illness | Cultural | Cultural (overall) | 12 | Okolo et al. 2024; Röing & Sanner 2015 |
| Chronic Illness | Cultural | Traditional healing | 2 | Okolo et al. 2024; Nikoloudi et al. 2024 |
| Chronic Illness | Cultural | Ethnic/racial disparities | 8 | Niño de Guzmán Quispe et al. 2021; Mustafa et al. 2020 |
| Chronic Illness | Cultural | Cultural competence | 8 | Al Kalaldeh et al. 2018; Okolo et al. 2024 |
| Chronic Illness | Cultural | Health beliefs | 3 | Okolo et al. 2024; Putri & Amir 2025 |
| Chronic Illness | Cultural | Folk illness | 1 | Okolo et al. 2024 |
| Chronic Illness | Economic | Economic (overall) | 15 | MacNeela et al. 2015; Chimezie 2023 |
| Chronic Illness | Economic | Financial strain | 8 | Chimezie 2023; MacNeela et al. 2015 |
| Chronic Illness | Economic | Direct costs | 3 | Chimezie 2023; Dueñas et al. 2016 |
| Chronic Illness | Economic | Indirect costs | 3 | Chimezie 2023; MacNeela et al. 2015 |
| Chronic Illness | Economic | Employment | 2 | MacNeela et al. 2015; Lundin et al. 2024 |
| Chronic Illness | Economic | Work capacity | 5 | Lundin et al. 2024; MacNeela et al. 2015 |
| Chronic Illness | Economic | Disability benefits | 1 | Schwarz et al. 2024 |
| Chronic Illness | Economic | Insurance barriers | 2 | Chimezie 2023; Najem et al. 2025 |
| Chronic Illness | Environmental | Environmental (overall) | 8 | Rodríguez et al. 2019; Okolo et al. 2024 |
| Chronic Illness | Environmental | Access to care | 2 | Kahere et al. 2022 |
| Chronic Illness | Environmental | Transportation | 1 | Kahere et al. 2022 |
| Chronic Illness | Environmental | Rural/urban disparities | 1 | Chimezie 2023 |
| Chronic Illness | Environmental | Pollution | 1 | Okolo et al. 2024 |
| Chronic Illness | Environmental | Housing quality | 1 | Chimezie 2023 |
| Chronic Illness | Environmental | Environmental triggers | 2 | Rodríguez et al. 2019; Chimezie 2023 |
| Chronic Illness | Physical | Physical (overall) | 45 | Dueñas et al. 2016; MacNeela et al. 2015 |
| Chronic Illness | Physical | Symptom burden | 20 | Dueñas et al. 2016; MacNeela et al. 2015 |
| Chronic Illness | Physical | Fatigue | 8 | Hassani et al. 2017; Bath et al. 2019 |
| Chronic Illness | Physical | Comorbidities | 16 | Dueñas et al. 2016; MacNeela et al. 2015 |
| Chronic Illness | Physical | Activity limitations | 10 | Lundin et al. 2024; Bath et al. 2019 |
| Chronic Illness | Physical | Mobility | 8 | Lundin et al. 2024; Bath et al. 2019 |
| Chronic Illness | Physical | ADL restrictions | 3 | Lundin et al. 2024 |
| Chronic Illness | Physical | Sleep disturbances | 4 | Dueñas et al. 2016; Matias-Soto et al. 2024 |
| Chronic Illness | Psychological | Psychological (overall) | 60 | Daker-White et al. 2014; Toye et al. 2019 |
| Chronic Illness | Psychological | Emotional distress | 15 | Daker-White et al. 2014; Mahon et al. 2014 |
| Chronic Illness | Psychological | Depression | 22 | Mahon et al. 2014; Peppard et al. 2023 |
| Chronic Illness | Psychological | Anxiety | 22 | Mahon et al. 2014; Peppard et al. 2023 |
| Chronic Illness | Psychological | Coping mechanisms (overall) | 8 | Fracso et al. 2021; Toye et al. 2021 |
| Chronic Illness | Psychological | Resilience | 6 | Fracso et al. 2021; Peppard et al. 2023 |
| Chronic Illness | Psychological | Acceptance | 6 | Toye et al. 2021; Macgregor et al. 2024 |
| Chronic Illness | Psychological | Avoidance | 3 | Stensland & Sanders 2018 |
| Chronic Illness | Psychological | Self-esteem | 8 | Lo Monaco et al. 2024; Snell et al. 2024 |
| Chronic Illness | Psychological | Catastrophizing | 5 | Stensland & Sanders 2018; Schütze et al. 2017 |
| Chronic Illness | Psychological | Positive reframing | 4 | Fracso et al. 2021; Toye et al. 2021 |
| Chronic Illness | Social | Social (overall) | 55 | Toye et al. 2017; MacNeela et al. 2015 |
| Chronic Illness | Social | Social support (overall) | 12 | Fracso et al. 2021; MacNeela et al. 2015 |
| Chronic Illness | Social | Family | 12 | MacNeela et al. 2015; Roenne et al. 2020 |
| Chronic Illness | Social | Friends | 4 | MacNeela et al. 2015 |
| Chronic Illness | Social | Peer support | 8 | Fracso et al. 2021; Merolli et al. 2014 |
| Chronic Illness | Social | Stigma and discrimination | 22 | Daker-White et al. 2014; Toye et al. 2019 |
| Chronic Illness | Social | Concealment | 4 | Ojala et al. 2015; Toye et al. 2017 |
| Chronic Illness | Social | Disclosure management | 4 | Toye et al. 2017; Ojala et al. 2015 |
| Chronic Illness | Social | Isolation and loneliness | 26 | Toye et al. 2017; MacNeela et al. 2015 |
| Chronic Illness | Social | Social participation barriers | 18 | Cheng et al. 2019; Toye et al. 2019 |
| Chronic Illness | Spiritual | Spiritual (overall) | 10 | Andersen et al. 2021; Hassani et al. 2017 |
| Chronic Illness | Spiritual | Faith/prayer/spirituality | 3 | Hassani et al. 2017; Andersen et al. 2021 |
| Chronic Illness | Spiritual | Meaning-making | 3 | Andersen et al. 2021; Toye et al. 2021 |
| Chronic Illness | Spiritual | Purpose | 2 | Andersen et al. 2021 |
| Chronic Illness | Spiritual | Existential questions | 1 | Andersen et al. 2021 |
| Chronic Illness | Spiritual | Post-traumatic growth | 1 | Fracso et al. 2021 |
| Chronic Pain | Chronic Pain (global) | Chronic Pain (global) | 81 | Furnes & Dysvik 2010; Toye et al. 2017; Snelgrove et al. 2013 |
| Chronic Pain | Cognitive | Cognitive (overall) | 8 | Schütze et al. 2017; Snelgrove et al. 2013 |
| Chronic Pain | Cognitive | Pain beliefs | 2 | Keen et al. 2021; Cooper et al. 2018 |
| Chronic Pain | Cognitive | Harm | 1 | Schütze et al. 2017 |
| Chronic Pain | Cognitive | Controllability | 4 | Keen et al. 2021; Mathias et al. 2014 |
| Chronic Pain | Cognitive | Health literacy | 1 | Merolli et al. 2014 |
| Chronic Pain | Cognitive | Attention strategies | 1 | Schütze et al. 2017 |
| Chronic Pain | Cognitive | Distraction strategies | 1 | Mathias et al. 2014 |
| Chronic Pain | Cultural | Cultural (overall) | 8 | Okolo et al. 2024; Mustafa et al. 2020 |
| Chronic Pain | Cultural | Cultural pain expression | 3 | Okolo et al. 2024; Palacios-Ceña et al. 2017 |
| Chronic Pain | Cultural | Stoicism | 2 | Toye et al. 2017; Okolo et al. 2024 |
| Chronic Pain | Cultural | Norms about pain communication | 1 | Toye et al. 2017 |
| Chronic Pain | Cultural | Ethnic disparities in pain management | 2 | Keen et al. 2021; Najem et al. 2025 |
| Chronic Pain | Economic | Economic (overall) | 5 | MacNeela et al. 2015; Ojala et al. 2015 |
| Chronic Pain | Economic | Healthcare costs | 3 | MacNeela et al. 2015; Chimezie 2023 |
| Chronic Pain | Economic | Procedures | 2 | Stensland 2021; Stensland & Sanders 2018 |
| Chronic Pain | Economic | Medications | 2 | Brooks et al. 2015; Vallerand & Nowak 2010 |
| Chronic Pain | Economic | Work absenteeism | 1 | MacNeela et al. 2015 |
| Chronic Pain | Economic | Reduced productivity | 1 | MacNeela et al. 2015 |
| Chronic Pain | Economic | Access to pain clinics | 1 | Kahere et al. 2022 |
| Chronic Pain | Economic | Insurance issues | 1 | Ojala et al. 2015 |
| Chronic Pain | Environmental | Environmental (overall) | 3 | Bath et al. 2019; Rodríguez et al. 2019 |
| Chronic Pain | Environmental | Built environment | 1 | Rodríguez et al. 2019 |
| Chronic Pain | Environmental | Workplace ergonomics | 1 | Bath et al. 2019 |
| Chronic Pain | Environmental | Environmental triggers | 2 | Chala et al. 2022; Rodríguez et al. 2019 |
| Chronic Pain | Environmental | Weather | 1 | Bath et al. 2019 |
| Chronic Pain | Environmental | Pollution | 2 | Okolo et al. 2024 |
| Chronic Pain | Physical | Physical (overall) | 45 | Snelgrove et al. 2013; Ojala et al. 2014; MacNeela et al. 2015 |
| Chronic Pain | Physical | Pain intensity | 15 | Snelgrove et al. 2013; Schütze et al. 2017 |
| Chronic Pain | Physical | Duration | 8 | Ojala et al. 2014; Stensland et al. 2018 |
| Chronic Pain | Physical | Distribution | 6 | Snelgrove et al. 2013; Coleman et al. 2016 |
| Chronic Pain | Physical | Breakthrough pain | 3 | Stensland 2021; Angel et al. 2017 |
| Chronic Pain | Physical | Chronicity | 12 | Ojala et al. 2014; Toye et al. 2017 |
| Chronic Pain | Physical | Disability | 5 | MacNeela et al. 2015; Stensland & Sanders 2018 |
| Chronic Pain | Physical | Mobility | 8 | Bath et al. 2019; Matias-Soto et al. 2024 |
| Chronic Pain | Physical | Comorbid symptoms | 18 | Dueñas et al. 2016; Mengshoel et al. 2018 |
| Chronic Pain | Physical | Fatigue | 3 | Bath et al. 2019; Hassani et al. 2017 |
| Chronic Pain | Physical | Sleep issues | 2 | Stensland & Sanders 2018; Matias-Soto et al. 2024 |
| Chronic Pain | Policy/Legal | Policy/Legal (overall) | 3 | Brooks et al. 2015; Schwarz et al. 2024 |
| Chronic Pain | Policy/Legal | Opioid prescribing regulations | 2 | Brooks et al. 2015; Vallerand & Nowak 2010 |
| Chronic Pain | Policy/Legal | Disability claims and law | 1 | Schwarz et al. 2024 |
| Chronic Pain | Policy/Legal | Advocacy for pain recognition | 1 | Gaber et al. 2024 |
| Chronic Pain | Psychological | Psychological (overall) | 55 | Snelgrove et al. 2013; Toye et al. 2017; Hassani et al. 2017 |
| Chronic Pain | Psychological | Anxiety | 22 | Matias-Soto et al. 2024; Lee et al. 2018 |
| Chronic Pain | Psychological | Depression | 22 | Peppard et al. 2023; Matias-Soto et al. 2024 |
| Chronic Pain | Psychological | Pain catastrophizing | 8 | Schütze et al. 2017; Mathias et al. 2014 |
| Chronic Pain | Psychological | Rumination | 10 | Schütze et al. 2017; Snelgrove et al. 2013 |
| Chronic Pain | Psychological | Fear-avoidance | 2 | Schütze et al. 2017; Lee et al. 2018 |
| Chronic Pain | Psychological | Mood disorders | 12 | Peppard et al. 2023; Matias-Soto et al. 2024 |
| Chronic Pain | Social | Social (overall) | 60 | Toye et al. 2017; MacNeela et al. 2015; Merolli et al. 2014 |
| Chronic Pain | Social | Social isolation | 20 | Toye et al. 2017; Merolli et al. 2014 |
| Chronic Pain | Social | Withdrawal | 12 | Toye et al. 2017; MacNeela et al. 2015 |
| Chronic Pain | Social | Loss of friendships | 8 | MacNeela et al. 2015; Toye et al. 2017 |
| Chronic Pain | Social | Family dynamics | 8 | Stensland & Sanders 2018; Roenne et al. 2020 |
| Chronic Pain | Social | Caregiver stress | 5 | Dueñas et al. 2016; Roenne et al. 2020 |
| Chronic Pain | Social | Role burden | 8 | MacNeela et al. 2015; Lundin et al. 2024 |
| Chronic Pain | Social | Social stigma | 18 | Brooks et al. 2015; Palacios‑Ceña et al. 2017 |
| Chronic Pain | Social | Disbelief | 4 | Ojala et al. 2015; Hansson et al. 2011 |
| Chronic Pain | Social | Labeling as malingering | 8 | Juuso et al. 2014; Ojala et al. 2015 |
| Chronic Pain | Spiritual | Spiritual (overall) | 1 | Hassani et al. 2017 |
| Chronic Pain | Spiritual | Spiritual coping | 1 | Hassani et al. 2017, Andersen et al. 2021 |
| Chronic Pain | Spiritual | Finding meaning | 1 | Andersen et al. 2021 |
| Chronic Pain | Spiritual | Prayer as relief | 1 | Hassani et al. 2017 |
| Chronic Pain | Technological | Technological (overall) | 1 | Merolli et al. 2014 |
| Chronic Pain | Technological | Digital pain diaries | 1 | Merolli et al. 2014 |
| Chronic Pain | Technological | Telemedicine | 1 | Morton et al. 2017 |
| Chronic Pain | Technological | Pain assessment apps/devices | 1 | Merolli et al. 2014 |

* Theme relevance (k) reflects the number of included studies citing each subtheme. To ensure robustness, we conducted sensitivity analyses and alternative coding scenarios (detailed in Supplementary Tables S10, S12, and S13), including conservative, balanced, and liberal weighting schemes, and alternative domain groupings. Two analysts (EAV and SSA) independently applied the initial codes and relevance scores, compared the results, and reconciled differences by consensus with the full team; adjudication decisions and audit‑ trial entries were recorded in the repository. After reviewing the sensitivity outputs and visualizations, we adopted the simplest and most transparent coding rule: assign relevance by observed literature mentions (frequency k) with Theme-based relevance weights (R); k = 1 item was treated as low relevance (R = 1). This pragmatic approach balanced reproducibility and interpretability while capturing the empirical prominence of themes across the corpus.

R (relevance score): A discrete expert-assigned score (1–3) representing thematic centrality and analytic confidence for a given subtheme across the included literature. Scale and interpretation: R = 3 (high relevance): The subtheme is central and repeatedly emphasized across many studies; it appears as a primary focus or core finding (strong, consistent evidence, and clear analytic emphasis). R = 2 (moderate relevance): Subthemes are frequently reported or clearly present but are typically secondary or context‑dependent (moderate empirical support). R = 1 (low relevance): Subtheme is infrequent or mentioned only once or in passing, with limited empirical support, or highly context-specific. How R was assigned: Two analysts (EAV and SSA) independently reviewed the study code matrices and text excerpts to judge centrality for each subtheme. The initial independent ratings were compared, disagreements were resolved through discussion and team consensus, and all decisions were logged into the audit trail. Theme applied rule-based defaults (see Supplementary Footnote): high (3) for global/primary domains (Chronic Pain/Illness, Physical, Psychological, Social); moderate (2) for secondary domains (Cognitive, Cultural, Economic, Environmental, Policy/Legal, Spiritual, Technological). Any subtheme with k = 1 was overridden to R = 1. Use in analysis: weighted score W = F × R (F = frequency, i.e., number of studies citing the subtheme). W was used for visualization (heatmap/chord diagrams) and raw W values were optionally normalized for plotting.

Sensitivity checks: Alternative R assignments and weighting scenarios (conservative, balanced, and liberal) were tested (see S10, S12, and S13) to confirm that the major patterns were robust to reasonable variations in R. Rationale: Combines empirical prominence (frequency) with expert judgment regarding thematic centrality to produce a simple, transparent, and reproducible weighting mechanism for cross-study synthesis.

**Supplementary Table S17. Transdisciplinary Study Characteristics/ Themes**

| Study Id | Title | Comments |
| --- | --- | --- |
| S001 | Yang X, Lo K. Environmental health research and the COVID-19 pandemic: A turning point towards sustainability. Environ Res. 2021 Jun;197:111157. doi: 10.1016/j.envres.2021.111157. Epub 2021 Apr 20. PMID: 33887273; PMCID: PMC8542966.  doi: 10.1016/j.envres.2021.111157 | This study theorized the interdependence of society, environment, and health, and presents an integrated framework for transdisciplinary and sustainability science. embracing complexity and uncertainty, addressing vulnerability, boosting resilience and promoting sustainable development. This study propagates that the pandemic could be an opportunity for sustainable transformation, wherein visionary leadership that facilitates sustainability policies based on environmental health science is required. It discusses equity, reciprocity, sustainability and the process of transformation. |
| S002 | Fischer, C., Gugerell, K., Laa, U. *et al.* Evaluating transdisciplinary methods: a new scale for measuring knowledge integration. *Humanit Soc Sci Commun***12**, 1395 (2025).  doi.org/10.1057/s41599-025-05634-w | This study assessed transdisciplinary methodologies and principles. It identifies transdisciplinary approaches as essential for facilitating knowledge integration, allowing participants both inside and outside academia to critically evaluate their expertise, exchange perspectives, and jointly develop innovative solutions. The work emphasizes the importance of epistemic processes within transdisciplinary science. |
| S003 | de Jager, L.A., Bal, M., Baudena, M. et al. Transdisciplinary complexity science: deepening system understanding for sustainability. Humanit Soc Sci Commun 12, 1384 (2025).  <https://doi.org/10.1057/s41599-025-05548-7> | This study described sustainability challenges and builds a framework for an approach that integrates quantitative complexity science with transdisciplinary approaches to create an integrated understanding of system change. The authors present a systematic literature analysis from an emerging field and develop a foundation for Transdisciplinary Complexity Science for Sustainability and derive best practices for how research can foster learning and action for sustainability. |
| S004 | Wouters, R., Cox, A. & Raes, A. Co-producing knowledge in and about education by combining collaborative ethnography and abductive reasoning. Humanit Soc Sci Commun 12, 1405 (2025).  <https://doi.org/10.1057/s41599-025-05721-y> | This study evaluated transdisciplinary principles, equity and reciprocity and transformational learning, knowledge co-production between academic and non-academic stakeholders. In addition, this study evaluates epistemological and methodological transdisciplinary principles. |
| S005 | Vienni-Baptista, B., & Pohl, C. E. (2024). Exploring interdisciplinarity and transdisciplinarity as knowledge regimes: A heuristic tool for disentangling understandings in academia and policy. Science, Technology, & Human Values, 49(6), 1309-1348.  <https://doi.org/10.1177/01622439231216789> | This paper introduces a heuristic tool developed to facilitate both interdisciplinary and transdisciplinary collaboration. The concept integrates multiple dimensions of both fields into focused discourse. The study recognizes that societal actors possess varied conceptions, definitions, and practices regarding inter- and transdisciplinary approaches, and seeks to foster dialogue to enhance the effectiveness of projects and programs. Grounded in the framework of the “knowledge regime,” which comprises ideologies and myths, shared beliefs and practices, and imaginaries and values, the authors offer a comprehensive perspective on the diverse interpretations of interdisciplinarity and transdisciplinarity, extending beyond purely cognitive considerations. Utilizing a qualitative methodology, the researchers designed a heuristic tool to systematically address this heterogeneity and enable comparability across differing understandings. |
| S006 | Yang, L., Shewchuk, B., Shang, C., Lee, J. A., & Gehlert, S. (2023). Transdisciplinary team science in Health Research, where are we?. Journal of Integrated Design and Process Science, 26(3-4), 307-316.  <https://doi.org/10.3233/JID-220011> | This paper investigated the progression of transdisciplinary principles within both clinical and experimental medical science. Traditional discipline-specific methodologies are effective at developing knowledge regarding isolated causes of specific disease entities. Multidisciplinary efforts enable the examination of disease causation across multiple levels, whereas interdisciplinary collaboration is commonly confined to adapting methods from one field to another, occasionally resulting in the emergence of new disciplines such as molecular epidemiology. Nevertheless, these collaborative models do not fully address the intricate nature of chronic diseases. Additionally, these approaches lack sufficient power to generate knowledge that is translatable into implementable solutions, because of their failure to provide a holistic view limited their ability to capture the complexity of real-world problems. Transdisciplinary collaborations gained popularity in health research when disciplinary researchers began to develop integrated research frameworks that transcended discipline-specific methods. Using cancer research as an example, this position paper describes the nature of different disciplinary collaborations, reviews transdisciplinary research projects funded by the US National Cancer Institute, discusses frameworks to develop shared mental models in teams and to evaluate transdisciplinary collaboration, highlights the role of team science in successful transdisciplinary health research, and proposes future research to develop the science of team science. |
| S007 | Van der Vlegel-Brouwer, W., Eelderink, M., & Bussemaker, J. (2023). Participatory Action Research as a Driver for Health Promotion and Prevention: A Co-creation Process Between Professionals and Citizens in a Deprived Neighbourhood in the Hague. International journal of integrated care, 23(4), 13.  doi.org/10.5334/ijic.7560 | This paper applied participatory principles to examine equitable epistemic processes. It provides an overview of significant disparities in health outcomes across societies, noting that public health systems in many nations bear the responsibility of enhancing population health while reducing such inequalities. Health inequalities are defined as systematic, avoidable, and unjust differences in health outcomes that occur between populations, within social groups, or along gradients related to social standing. Employing Participatory Action Research (PAR), this study investigates approaches for effective prevention and health promotion in disadvantaged neighborhoods. The primary aim of the initial PAR cycle was to address the disconnect between institutional and professional viewpoints and the lived experiences of community members. Furthermore, the process sought to foster collaboration between professionals and citizens, allowing them to jointly scrutinize health disparities and collaboratively develop solutions to improve health outcomes. |
| S008 | Ortwin Renn, Transdisciplinarity: Synthesis towards a modular approach:  Futures, Volume 130, 2021,102744,ISSN 0016-3287  [doi.org/10.1016/j.futures.2021.102744](https://doi.org/10.1016/j.futures.2021.102744) | This paper presents a conceptual framework for organizing effective transdisciplinary communication between knowledge holders and recipients. It aims to blend scientific rigor with innovative co-production of knowledge, acknowledging that intuition is insufficient for complex challenges. The review covers key models of transdisciplinarity, and concludes that none fully integrate scientific excellence, future focus, practical relevance, and social coherence. To address this, the author proposes a modular approach for incorporating transdisciplinary science into societal transformation and democratic policymaking. |
| S009 | Chamberlain C, Clark Y, Hokke S, Hampton A, Atkinson C, Andrews S; HPNF investigator group. Healing the Past by Nurturing the Future: Aboriginal parents' views of what helps support recovery from complex trauma. Prim Health Care Res Dev. 2021 Sep 30;22:e47.  doi: 10.1017/S1463423621000463 | This research explored the support needs of Aboriginal and Torres Strait Islander parents dealing with complex trauma. As part of a participatory action project, it aimed to co-design strategies for perinatal awareness, assessment, and support using an Indigenist approach and grounded theory. Data were gathered through group discussions and interviews led by Aboriginal researchers, using scenarios and visual tools to prompt reflections about past impacts, sources of strength, aspirations, and needed supports. Parents also reviewed themes from prior studies to identify additional issues. The study identified transformation from cycles of trauma to nurturing, shaped by connectedness, social and emotional wellbeing, and parenting transitions. The findings highlight varying experiences by gender, age, and community, emphasizing participatory principles and equitable involvement in knowledge creation. |
| S010 | Hantel A, Marron JM, Abel GA. Establishing and Defining an Approach to Climate Conscious Clinical Medical Ethics. Am J Bioeth. 2025 Jul;25(7):8-21.  doi: 10.1080/15265161.2024.2337418 | This study highlighted that climate change is a crucial, yet under-addressed, issue in clinical medical ethics. The authors examine how existing moral theories within clinical medical ethics relate to environmental threats, revealing a disconnect between theory and practice. To bridge this gap, they propose expanding the field's scope using Jonsen and Siegler’s Four Topics model, integrating climate consciousness into ethics education for future healthcare providers. Their medical school has introduced climate-focused curricula, and hospital ethics committees are encouraged to collaborate with sustainability bodies to promote sustainable care and guide policy. The authors indicate further exploration of justice theories and the ethical obligations of professionals when patient and environmental interests conflict, emphasizing the need for clinical medicine to align with ecological responsibility through virtues like care and climate justice. |
| S011 | Ciesielski TH, Aldrich MC, Marsit CJ, Hiatt RA, Williams SM. Transdisciplinary approaches enhance the production of translational knowledge. Transl Res. 2017 Apr;182:123-134. doi: 10.1016/j.trsl.2016.11.002. Epub 2016 Nov 10. PMID: 27893987; PMCID: PMC5362296.  doi: 10.1016/j.trsl.2016.11.002 | This paper examined transdisciplinary principles within the context of complex medical challenges. The authors argue that a unidisciplinary approach may be inadequate at addressing multifactorial disease causation or urgent health decisions. To address these limitations, they advocate for transdisciplinary strategies to enhance the advancement of translational research, presenting a review of relevant publications that illustrate the application of such approaches. These examples underscore the value of transdisciplinary research and aim to foster further discussion on its promotion. The paper emphasizes that open and flexible communication is essential for initiating transdisciplinary collaborations, requiring defined roles in their facilitation. Rather than outlining a fixed protocol—as none currently exists—the authors highlight that progress was achieved in environments where disciplinary and institutional barriers were manageable, without reliance on predetermined plans. As the benefits of cross-disciplinary exchange are often unpredictable *a priori*, imposing rigid protocols may hinder novel discoveries. Ultimately, these case studies demonstrate that improved interdisciplinary information sharing can enable researchers to pose more insightful questions, synthesize pertinent evidence, and advance translational science. Specifically, the authors discuss instances in which transdisciplinary approaches have supported the exploration, assessment, and intervention efforts aimed at improving human health. |
| S012 | Carey G, Malbon E, Carey N, Joyce A, Crammond B, Carey A. Systems science and systems thinking for public health: a systematic review of the field. BMJ Open. 2015 Dec 30;5(12):e009002. doi: 10.1136/bmjopen-2015-009002. PMID: 26719314; PMCID: PMC4710830.  DOI: 10.1136/bmjopen-2015-009002 | This systematic review of the existing systems science literature in public health indicates significant interest in the potential contributions of systems concepts and approaches to the field. The analysis demonstrates that soft systems modeling techniques may offer substantial value to public health and are closely aligned with ongoing discussions around knowledge transfer and policy development. Nevertheless, it is important to recognize that the full spectrum of systems methodologies has not yet been thoroughly explored by public health researchers and practitioners. Overall, the authors state that there remains considerable interest in how the integration of systems concepts and approaches could further benefit public health practice. |
| S013 | Kabarite A, Ferreira GMM, Pitangueira JC, Arimatéa RS, da Costa Rebello de Mendonça R, Marcello RS, Schulz TG, Riesgo RDS, Castro K. A Longitudinal Transdisciplinary Approach for Autism Spectrum Disorder. Children (Basel). 2025 Sep 22;12(9):1272. doi: 10.3390/children12091272. PMID: 41007137; PMCID: PMC12469184.  DOI: 10.3390/children12091272 | This study evaluated the effectiveness of a transdisciplinary, family-focused model on clinical and functional outcomes for children and adolescents with Autism Spectrum Disorder (ASD), a condition characterized by complex developmental challenges that require coordinated, individualized support. Participants received tailored, evidence-based interventions from a multidisciplinary team, operating within a transdisciplinary framework. Interventions were provided both individually and in group contexts, utilizing flexible phases to accommodate each child's evolving needs. Over the 12-month period, the majority of participants demonstrated significant improvements in overall functioning. Caregivers reported notable reductions in support requirements, whereas therapists observed moderate, yet meaningful, advancements. The study documented decreases in behavioral difficulties, sensory sensitivities, and sleep disturbances, alongside enhancements in adaptability and play-related abilities. Greater family involvement was associated with improved outcomes. In summary, the transdisciplinary, family-centered approach was effective in supporting developmental progress, underscoring the importance of adaptable, collaborative care and active family engagement in achieving sustained success. |
| S014 | Gordon RM, Corcoran JR, Bartley-Daniele P, Sklenar D, Sutton PR, Cartwright F. A transdisciplinary team approach to pain management in inpatient health care settings. Pain Manag Nurs. 2014 Mar;15(1):426-35. doi: 10.1016/j.pmn.2013.01.004. Epub 2013 Mar 22. Erratum in: Pain Manag Nurs. 2014 Dec;15(4):920. PMID: 23522877.  DOI: 10.1016/j.pmn.2013.01.004 | This paper examined the shift from multidisciplinary to interdisciplinary and transdisciplinary approaches in pain management. It highlights how transdisciplinary teams focus on shared learning, flexible roles, and unified, patient-centered treatment plans that incorporate cultural context and empower both patients and their support systems. The article compares three team models, discussing their foundations, advantages, challenges, research evidence, and attention to cultural diversity. Case vignettes illustrate the benefits and limitations of the transdisciplinary model across various care settings, including acute, palliative, perioperative, and rehabilitation care. |
| S015 | Burris S, Ashe M, Levin D, Penn M, Larkin M. A Transdisciplinary Approach to Public Health Law: The Emerging Practice of Legal Epidemiology. Annu Rev Public Health. 2016;37:135-48. doi: 10.1146/annurev-publhealth-032315-021841. Epub 2015 Nov 30. PMID: 26667606; PMCID: PMC5703193.  DOI: 10.1146/annurev-publhealth-032315-021841 | This paper presents a comprehensive approach to public health law that synthesizes legal analysis with scientific methodologies, such as policy monitoring and empirical research. Integrating these disciplines facilitates more effective evaluation of legal measures in advancing health outcomes and addressing enduring challenges, including cultural and resource constraints. The authors advocate for an interdisciplinary framework, underscoring the importance of collaborative strategies to dismantle disciplinary silos and expand the influence of law within public health. They outline a more inclusive model that connects legal and scientific aspects of public health law. Core activities—such as policy development, surveillance, implementation, and evaluation—remain fundamental responsibilities of public health practitioners and researchers. These serve as the foundation for impactful public health practice. Reducing persistent cultural, disciplinary, and resource barriers is essential to fully realizing and optimizing the role of law in public health. |
| S016 | Mishra, K. K., Leung, I. C., Chao, M. T., Thompson-Lastad, A., Pollak, C., Dhruva, A., ... & Atreya, C. E. (2024). Mindfulness-based group medical visits: strategies to improve equitable access and inclusion for diverse patients in cancer treatment. Global Advances in Integrative Medicine and Health, 13, 27536130241263486.  doi.org/10.1177/27536130241263486 | The authors assessed their experience implementing mindfulness-based interventions (MBIs) and reflexive techniques tailored for feasibility and relevance during cancer treatment, with consideration for cultural appropriateness among racially, ethnically, and linguistically diverse groups. They highlight that translating evidence-based interventions into clinical practice necessitates rigorous attention to maintaining intervention fidelity, alongside adaptations that promote contextual fit and flexibility across varying populations. Furthermore, they assert that adapted MBIs should be integrated into routine oncologic care to enhance accessibility, particularly for patients facing financial constraints and intensive treatment schedules. The authors emphasize the importance of addressing implementation factors, such as patient-level barriers and healthcare settings, to support equitable access to mindfulness-based interventions.  In the authors’ view, guideline-concordant symptom management must be available to all individuals undergoing cancer therapy. Integrating culturally-adapted and trauma-informed mind-body practices within standard oncologic care represents a promising strategy for mitigating health and healthcare disparities among patients. |
| S017 | Allen-Scott, L. K., Buntain, B., Hatfield, J. M., Meisser, A., & Thomas, C. J. (2015). Academic Institutions and One Health: Building Capacity for Transdisciplinary Research Approaches to Address Complex Health Issues at the Animal-Human-Ecosystem Interface. Academic medicine : journal of the Association of American Medical Colleges, 90(7), 866–871.  doi.org/10.1097/ACM.0000000000000639 | The authors examined the significance of transdisciplinary methodologies in addressing One Health challenges and present targeted recommendations for academic institutions, building upon prior advancements in translational and transdisciplinary research. Translational research—commonly referred to as the “bench to bedside” approach—has progressed through initiatives, such as the Clinical and Translational Science Awards. These awards are designed to attract, develop, and support multidisciplinary and interdisciplinary investigators and research teams; foster innovative information technologies and research tools; and enhance translational research by encouraging the integration of novel knowledge and techniques into clinical practice. Achieving effective transdisciplinary research necessitates the establishment of centers of excellence dedicated to cultivating transdisciplinary principles and facilitating meaningful collaboration.  The recommendations outlined by the authors emphasize the creation of supportive environments for One Health and transdisciplinary research, development of experiential educational programs that promote collaboration across various disciplines and engagement with nonacademic stakeholders, and implementation of new funding structures tailored to One Health transdisciplinary research. The authors conclude that, in the absence of substantial changes in academic research capacity and methodology, there is a risk of merely rebranding existing activities as transdisciplinary, thereby failing to realize the full potential of transdisciplinary One Health research in tackling complex global health issues at the intersections of animal, human, and ecosystem health. |
| S018 | Collins, S. E., Clifasefi, S. L., Stanton, J., The Leap Advisory Board, Straits, K. J. E., Gil-Kashiwabara, E., Rodriguez Espinosa, P., Nicasio, A. V., Andrasik, M. P., Hawes, S. M., Miller, K. A., Nelson, L. A., Orfaly, V. E., Duran, B. M., & Wallerstein, N. (2018). Community-based participatory research (CBPR): Towards equitable involvement of community in psychology research. The American psychologist, 73(7), 884–898.  [doi.org/10.1037/amp0000167](https://doi.org/10.1037/amp0000167) | This study evaluated Community-based Participatory Research (CBPR) as a methodology designed to enhance patient- and community-centered approaches in research and clinical practice, particularly in response to increasing health disparities. The authors describe CBPR as an inclusive research strategy that positions community members, researchers, and stakeholders as equal partners throughout the investigative process, recognizing the distinct contributions each participant offers. The CBPR seeks to integrate knowledge generation with concrete actions that support enduring social transformation. Originating in disciplines such as psychology, sociology, and critical pedagogy, CBPR is now prevalent in fields such as public health, medicine, and nursing; nonetheless, its application remains relatively limited within psychology research, despite strong alignment with the discipline’s ethical frameworks and overarching objectives. This article provides an overview of CBPR principles tailored for a psychology-focused audience, contrasting these practices with conventional psychological research methods, summarizing CBPR’s historical evolution, and presenting guidelines for practical adoption. It further addresses both the advantages and the practical and ethical challenges inherent in implementing CBPR within psychology. A case study is included to demonstrate the fundamental concepts and practical relevance of CBPR. The authors contend that CBPR represents a valuable and promising avenue for advancing effective, culturally responsive, socially equitable, and sustainable community-based psychological research. |
| S019 | Fontana, L., Fasano, A., Chong, Y. S., Vineis, P., & Willett, W. C. (2021). Transdisciplinary research and clinical priorities for better health. PLoS Medicine, 18(7), e1003699.  [doi.org/10.1371/journal.pmed.1003699](https://doi.org/10.1371/journal.pmed.1003699) | The authors assessed the importance of shifting from a predominantly disease-focused medical model to an integrated transdisciplinary approach that emphasizes preventive and personalized care. Such a transition is critical for reducing health disparities and achieving financially sustainable, universal health coverage. This article presents various evidence-based strategies, policy recommendations, and structural reforms aimed at establishing a prevention-centered transdisciplinary framework in scientific research, education, and healthcare systems that aims to support both human and environmental well-being.  Drawing on preclinical, epidemiological, and clinical trial findings, the authors highlight that many prevalent chronic conditions can be largely prevented through targeted nutritional and lifestyle interventions addressing key signaling pathways and interactions with the microbiome. While advancements in modern medicine allow individuals to manage multiple chronic diseases over extended periods, these developments are accompanied by significant social, financial, and environmental implications. Currently, research priorities and funding in healthcare are primarily directed toward identifying new molecular targets for biotechnological and pharmaceutical innovation, whereas investment in mechanism-driven preventive science, medicine, and education remains minimal. The authors caution that excessive optimism regarding the therapeutic potential of pharmacological research may inadvertently influence and distort not only medical research and practice, but also environmental health and sustainable economic development. |
| S020 | Keng, S. L., Smoski, M. J., & Robins, C. J. (2011). Effects of mindfulness on psychological health: a review of empirical studies. Clinical psychology review, 31(6), 1041–1056.  [doi.org/10.1016/j.cpr.2011.04.006](https://doi.org/10.1016/j.cpr.2011.04.006) | This study examined numerous investigations of mindfulness that have identified correlations between self-reported mindfulness and psychological health. Through a review of the empirical literature employing various methodologies, it is concluded that mindfulness and its cultivation support adaptive psychological functioning. Although there are methodological limitations within each body of research, consistent findings emerge across correlational studies, clinical intervention studies, and laboratory-based experimental studies—all indicating that mindfulness is positively associated with psychological wellbeing. This article reviews the empirical evidence regarding the effects of mindfulness on wellbeing. It begins by discussing the construct of mindfulness, distinguishing Buddhist from Western psychological conceptualizations, and examining the integration of mindfulness into Western medicine and psychology. Subsequently, three key areas of empirical research are reviewed: cross-sectional and correlational research on the associations between mindfulness and indicators of psychological health; intervention studies assessing the effects of mindfulness-oriented approaches on psychological health; and experimental research investigating the immediate effects of mindfulness inductions on emotional and behavioral functioning. The authors conclude that mindfulness yields a variety of positive psychological outcomes, including enhanced subjective well-being, reduced psychological symptoms and emotional reactivity, and improved behavioral regulation. They further address the mechanisms underlying change in mindfulness interventions and suggest directions for future research. |
| S021 | Knudsen, R. K., Skovbjerg, S., Pedersen, E. L., Nielsen, C. L., Storkholm, M. H., & Timmermann, C. (2024). Factors affecting implementation of mindfulness in hospital settings: A qualitative meta-synthesis of healthcare professionals' experiences. International journal of nursing studies advances, 6, 100192.  <https://doi.org/10.1016/j.ijnsa.2024.100192> | This qualitative meta-synthesis examined how mindfulness-based interventions are implemented in hospital settings, focusing on healthcare professionals' experiences. Although mindfulness programs have demonstrated benefits for reducing stress, supporting mental well-being, and enhancing communication and compassionate care, their integration into clinical work remains uneven and poorly understood. Drawing on 18 qualitative studies, the review identified three overarching influences on successful implementation. The first involved the need for individuals and organizations to “buy in,” meaning that the perceived value of mindfulness, attitudes toward its legitimacy, leadership endorsement, and group dynamics all affected engagement. The second concerned the practical requirements for allocating time and space, as participation often depended on protected work hours, adequate staffing coverage, and suitable physical or virtual environments. The third influence focused on sustaining mindfulness following the initial training, drawing on informal everyday practices, group support, follow-up sessions, and local champions who could model and promote continued use. From these findings, the authors developed eight recommendations for clinical settings, highlighting the importance of contextualizing benefits, respecting voluntary participation, fostering supportive group experiences, providing protected time, offering online options, encouraging brief informal practices, facilitating follow-up networks, and empowering local advocates. The study concludes that successful implementation is multifaceted, shaped by organizational culture, resource allocation, and ongoing facilitation, and calls for further research focused specifically on implementation processes. |
| S022 | Lawless, M. T., Tieu, M., Archibald, M. M., Pinero De Plaza, M. A., & Kitson, A. L. (2025). From Promise to Practice: How Health Researchers Understand and Promote Transdisciplinary Collaboration. Qualitative health research, 35(1), 3–16.  <https://doi.org/10.1177/10497323241235882> | This qualitative study examined how health researchers perceive and promote transdisciplinary collaboration within complex health research contexts. Transdisciplinary research, defined as integrative work that brings together diverse disciplines to address multifaceted health system challenges, has been increasingly emphasized in health sciences, yet little is known about how researchers themselves understand and operationalize it. The authors conducted a longitudinal realist evaluation within a National Health and Medical Research Council-funded research center focused on frailty, involving interviews and reflective data from team members engaged across disciplines. Participants described transdisciplinary collaboration as both collaborative and integrative, yet commonly used terms such as ‘*interdisciplinary*’ and ‘*multidisciplinary*’ interchangeably, highlighting a lack of shared conceptual clarity. The major barriers included time constraints, geographical separation, and entrenched collaboration patterns that hindered fluid integration. To overcome these challenges, researchers recommended establishing clear shared goals and visions, thereby creating appropriate systems and structures to support collaboration, and role-modeling collaborative behaviors, values, and attitudes across career stages. The findings emphasize the need for practical leadership development, experiential learning opportunities, and supportive environments to translate transdisciplinary promise into practice in health research. |
| S023 | Tobias Mortlock, J. M., Alapati, H. H., & Edginton, T. (2025). How to create a mindful community of practice: Exploring the social functions of group-based mindfulness practices facilitated via Zoom during Covid-19. Frontiers in Psychology, 16, 1356057.  <https://doi.org/10.3389/fpsyg.2025.1356057> | This exploratory qualitative study examined the social functions of group-based mindfulness practice within an online community of practice established during the COVID-19 pandemic. Conducted at a large metropolitan university, the research focused on participants who regularly joined free, 30-minute lunchtime mindfulness sessions offered three times per week via Zoom. Through semi-structured interviews with members of this sustained community, the authors investigated how shared mindfulness practice fosters connections, collective awareness, and a sense of common humanity beyond individual benefit. Drawing on interdependence theory, the findings suggest that regular collective mindfulness contributes to enhanced mind–body awareness, trust, interpersonal connection, and alignment among participants, supporting not only personal well-being but also social cohesion. The study highlights the transformative potential of mindfulness when practiced collectively, foregrounding how group dynamics and shared reflective inquiry can create supportive environments that extend benefits into workplace, educational, or broader community settings. The authors propose exploratory propositions for cultivating mindful communities and call for further research to validate and refine these insights, particularly regarding long-term group mindfulness and its social impact. |
| S024 | Marikar Bawa FL, Mercer SW, Sutton JW, Bond CM. Mindfulness for people with chronic pain: Factors affecting engagement and suggestions for programme optimisation. Health Expect. 2023 Jun;26(3):1287-1307. doi: 10.1111/hex.13745. Epub 2023 Mar 12. PMID: 36908018; PMCID: PMC10154871.  doi: 10.1111/hex.13745. Epub 2023 Mar 12. | This qualitative study examined the factors influencing engagement with an 8-week mindfulness-based stress reduction program among adults living with chronic pain and generated suggestions to tailor the program to improve accessibility and acceptability. Chronic pain is a complex biopsychosocial condition, and although mindfulness may support self-management, high dropout rates in existing program suggest that barriers to engagement remain poorly understood. Using interpretative phenomenological analysis of pre-, post-, and follow-up interviews with participants in the mindfulness program, the authors identified physical, psychological, and social factors affecting engagement. Physical factors included disability, discomfort with certain practices, logistical issues like transport, and inadequate management of pain before participation. Psychological factors encompass preconceptions, expectations (e.g., expecting pain relief), motivation, and understanding of how mindfulness relates to pain. Social factors involved loneliness and the positive role of group support. Participants and healthcare professionals then contributed to 20 suggested program modifications designed to address these barriers at different stages (enrolment, attendance, home practice, and continuation), such as improved pre-program information and screening, shorter initial practices, tailored options for pain limitations, enhanced group support, and ongoing resources for sustained practice. The authors conclude that tailoring mindfulness program to acknowledge the lived experience of chronic pain and addressing multifaceted barriers may enhance engagement and warrant further testing in controlled studies. |
| S025 | Zeidan F, Vago DR. Mindfulness meditation-based pain relief: a mechanistic account. Ann N Y Acad Sci. 2016 Jun;1373(1):114-27. doi: 10.1111/nyas.13153. PMID: 27398643; PMCID: PMC4941786.  doi: 10.1111/nyas.13153 | This review article synthesized the existing evidence on how mindfulness meditation reduces the subjective experience of pain and outlines the underlying psychological and neural mechanisms. Pain is a multidimensional phenomenon influenced by sensory, cognitive, and affective processes, and chronic pain treatment remains challenging, costly, and often reliant on opioids, contributing to misuse and addiction. The authors argue that mindfulness meditation offers an effective nonpharmacological alternative for pain relief that operates through multiple distinct mechanisms, making it a promising approach for individuals seeking self-facilitated, narcotic-free pain management. Mindfulness meditation is generally defined as sustained, present-moment awareness with nonjudgmental acceptance, with practices ranging from focused attention to open monitoring. Evidence shows that mindfulness training can significantly attenuate pain intensity and particularly pain unpleasantness across various conditions, including chronic low back pain, fibromyalgia, migraine, and pelvic pain. Neuroimaging studies suggest that mindfulness alters pain processing by reappraising nociceptive input, engaging cognitive control networks, and decoupling sensory and appraisal-related brain regions. Compared to placebo, mindfulness-induced analgesia does not rely on endogenous opioidergic systems. The authors conclude that even brief mindfulness training can modulate pain and call for further research to clarify mechanisms and enhance clinical applicability. |
| S026 | Martin, A. K., Green, T. L., McCarthy, A. L., Sowa, P. M., & Laakso, E. L. (2022). Allied health transdisciplinary models of care in hospital settings: A scoping review . Journal of Interprofessional Care, 37(1), 118–130.  <https://doi.org/10.1080/13561820.2022.2038552> | This scoping review examined the evidence for the implementation of transdisciplinary allied health models of care in hospital settings to understand their characteristics, outcomes, and the factors that facilitate or hinder their use. Allied health transdisciplinary teams feature professionals who share overlapping skills and work beyond traditional professional boundaries to deliver coordinated care, potentially improving time efficiency, quality of care, and value for patients and health services. Using the Joanna Briggs Institute methodology and PRISMA-ScR reporting, the authors systematically identified and synthesized nine studies evaluating hospital-based transdisciplinary allied health teams. Most included studies reported positive outcomes, such as improvements in efficiency, quality of care delivery, and favorable stakeholder perceptions, and one reported reductions in labor and capital costs. The review also mapped barriers and facilitators to implementation, organizing them into categories including individual and interpersonal factors, workflow design, organizational structures, and practical implementation issues. While evidence suggests potential benefits of transdisciplinary models, the authors noted that high-quality research remains limited and highlighted the need for further primary studies, especially those focusing on stakeholder experiences and the economic implications of transdisciplinary care. |
| S027 | O’Shea, M., Reich, D. & Evans, S. Applying Participatory Research in the Development of Clinical Practice Recommendations for Incorporating Mindfulness into Mental Health Treatment with Youth at Risk for Psychosis. Mindfulness 15, 721–732 (2024).  <https://doi.org/10.1007/s12671-024-02313-w> | This article reports on the development of clinical practice recommendations for safely and effectively integrating mindfulness-based strategies into mental health care for young people at risk for psychosis. Recognizing a gap in guidance for implementing mindfulness interventions with individuals exhibiting attenuated psychotic symptoms, the authors used participatory research methods across three phases. The first phase involved evidence mapping of existing mindfulness research across stages of psychosis, showing limited studies specific to the at-risk group. The second phase incorporated qualitative input from both practitioners experienced in early psychosis care and youth at risk, identifying key enablers and barriers to mindfulness uptake, including the importance of youth-relevant content, trauma-sensitive practice adaptations, compassion-focused elements, and flexible formats. In the third phase, preliminary recommendations were refined through validation with clinicians to ensure alignment with practice needs and stakeholder views. The final guidance highlights eight core areas, such as tailoring mindfulness exercises to developmental and cognitive needs, integrating trauma-informed principles, using compassion practices, and addressing barriers to engagement. The authors anticipate that these recommendations will enhance routine clinical use of mindfulness in early intervention services and improve outcomes such as distress management, functioning, and recovery among youth at elevated psychosis risk |
| S028 | Martins, P. (2018). Being Transdisciplinary in Human Sciences: The usefulness of Integrative medicine in contemporary society. Transdisciplinary Journal of Engineering and Science, 9(5), 37-43.  doi: 10.22545/2018/00100 | This article discusses the value of integrative medicine within the context of human sciences, and argues for the importance of a transdisciplinary approach in addressing contemporary health challenges. The author critiques conventional Western medicine for its predominant focus on external biomedical interventions, such as pharmacology, surgery, and radiation, that target biological symptoms without fully addressing the interconnected psychological, social, and spiritual dimensions of health. Drawing on traditions such as *Ayurveda* and broader mind-body medicine paradigms, this article illustrates how illnesses such as depression and other psychosomatic conditions may stem from imbalances between mental and physical processes. In contrast to the reductionist model of conventional care, integrative medicine emphasizes holistic healing and encourages patients’ active engagement in their own well-being through practices such as meditation, psychotherapy, and lifestyle changes. The author situates this perspective within a transdisciplinary framework, where knowledge synthesis across disciplines transcends traditional boundaries to develop more comprehensive, patient-centered approaches to health and healing. By integrating insights from medicine, psychology, philosophy, and cultural studies, the article posits that integrative and transdisciplinary medicine can contribute to achieving UN Sustainable Development Goal 3, ensuring healthy lives and promoting wellbeing for all ages, by embracing the full complexity of human health beyond narrow biomedical models |
| S029 | Schuman-Olivier Z, Trombka M, Lovas DA, Brewer JA, Vago DR, Gawande R, Dunne JP, Lazar SW, Loucks EB, Fulwiler C. Mindfulness and Behavior Change. Harv Rev Psychiatry. 2020 Nov/Dec;28(6):371-394. doi: 10.1097/HRP.0000000000000277. PMID: 33156156; PMCID: PMC7647439.  doi: 10.1097/HRP.0000000000000277 | This integrative review examines the role of mindfulness and mindfulness-based interventions (MBIs) in supporting the behavior changes relevant to physical and mental health outcomes. Recognizing that the initiation and maintenance of health-related behavior change (e.g., smoking cessation, diet, substance use, medical adherence) are central challenges in preventing and treating chronic disease, the authors synthesize theoretical, neuroscientific, and clinical research to clarify how mindfulness may influence motivation, learning, and self-regulation. This review begins by defining mindfulness as present-moment awareness with nonjudgmental acceptance and situating MBIs within the broader landscape of health behavior interventions. It subsequently offers an updated “mindful self-regulation model” that integrates motivation and learning mechanisms with cognitive and emotional processes to explain how mindfulness can alter habitual responses and support sustained behavior change. Neuroscientific evidence suggests that MBIs engage neural systems involved in attention, emotion regulation, and self-related processing, thereby enhancing individuals’ capacity to align behavior with long-term health goals. The article also synthesizes clinical studies of MBIs targeting behaviors relevant to psychiatric care, highlights current research limitations, and proposes mechanisms for future testing. Overall, the review underscores mindfulness’s potential to facilitate adaptive change and calls for more targeted investigation into mechanisms and clinical application. |
| S030 | Strugnell, C., Orellana, L., Crooks, N. et al. Healthy together Victoria and childhood obesity study: effects of a large scale, community-based cluster randomised trial of a systems thinking approach for the prevention of childhood obesity among secondary school students 2014–2016. BMC Public Health 24, 355 (2024).  <https://doi.org/10.1186/s12889-024-17906-2> | This study evaluated the impact of Healthy Together Victoria (HTV), a government-led, whole-of-community systems-thinking initiative designed to address chronic disease risk factors—including physical inactivity, poor diet quality, harmful alcohol use, and smoking—on overweight, obesity, and related behaviours among secondary school students in Victoria, Australia. HTV involved a large workforce supporting community and school-level actions to promote health and well-being. Using a cluster randomized trial embedded within repeated cross-sectional data collection, researchers collected anthropometric (body mass index and waist circumference) and behavioral data on students aged approximately 13-16 years old in 2014 and 2016 across 23 communities. The analysis found that boys in intervention communities showed favorable reductions in waist circumference and decreased sugar-sweetened beverage consumption compared with controls, although there were no significant effects on BMI overall or among girls. While some positive changes were observed, the intervention’s early cessation and the relatively short interval between measurements may have limited the detectable impact. Findings contribute to the evidence base on the potential of large-scale, systems-oriented community interventions to influence obesity-related outcomes, but highlight the need for longer, sustained efforts and a deeper understanding of the factors driving effectiveness. |
| S031 | Morgan, M.J., Stratford, E., Harpur, S. et al. A Systems Thinking Approach for Community Health and Wellbeing. Syst Pract Action Res 37, 161–183 (2024).  <https://doi.org/10.1007/s11213-023-09644-0> | This article reports on a place-based systems thinking approach designed to better address complex community health and wellbeing issues, particularly noncommunicable diseases, within the context of local government planning in a remote municipality in Tasmania, Australia. Traditional planning strategies commonly treat health problems in isolation, which limits their effectiveness for multifaceted challenges. To overcome these limitations, the authors implemented a facilitated systemic inquiry combined with a collaborative “rich picture” process, engaging diverse stakeholders—including local government representatives, community members, and practitioners—to co-explore the systemic causes and interconnections underlying local health concerns. Participants broadly supported the systems thinking approach, reporting that it helped increase their capacity to think systemically, build shared understanding, and reveal structural issues that conventional methods might overlook. In particular, the rich picture was valued for making complex relationships visible and fostering collaborative dialogue. The study also identified the need for supportive macro-level policies to enable stakeholders to apply systems approaches more consistently in practice. Findings have practical implications for public health planning, community practitioners, and policy makers seeking more sustainable and effective strategies for improving health and wellbeing outcomes in rural and remote settings, as well as for research into systems change methods more broadly. |
| S032 | Anindhita, M., Haniifah, M., Putri, A.M.N. et al. Community-based psychosocial support interventions to reduce stigma and improve mental health of people with infectious diseases: a scoping review. Infect Dis Poverty 13, 90 (2024).  <https://doi.org/10.1186/s40249-024-01257-6> | This scoping review synthesized evidence regarding community-based psychosocial support interventions designed to reduce stigma and improve mental health among people affected by highly stigmatized infectious diseases—including HIV/AIDS, tuberculosis (TB), and leprosy—across low- and middle-income settings. The authors systematically searched literature on interventions delivered outside formal healthcare facilities by peers, community members, volunteers, or lay workers, identifying diverse activities such as group and individual counselling, home visits, family support, youth volunteer programs, community conversations, and media-based education. These interventions were linked to reduced internalized and enacted stigma, enhanced psychosocial wellbeing, improved treatment adherence, better quality of life, and increased knowledge about disease. Peer and community involvement was highlighted as central to intervention success, with trained supporters helping to build confidence, mitigate isolation, facilitate access to services, and challenge negative beliefs. The review further noted methodological limitations, including a scarcity of high-quality evaluations and inconsistent use of validated outcome measures. The authors emphasize the importance of co-designing interventions with affected communities to ensure cultural relevance, sustainability, and broader impact on stigma and mental health outcomes in diverse contexts. |
| S033 | Kanyagui, M. K., Sajithkumar, K. J., Velankar, Y., Mohan, R., Viswanathan, P. K., & Magnani, N. (2023). Livelihood challenges faced by women in rural India: exploration of solutions using participatory action research. Development in Practice, 34(4), 512–526.  <https://doi.org/10.1080/09614524.2023.2285251> | This article explores the livelihood challenges faced by women in a rural village in Uttar Pradesh, India, and identifies sustainable, community-driven solutions through a participatory action research (PAR) approach. The authors worked with two self-help groups, encompassing local women, to collaboratively identify barriers to earning a sustainable income and to co-develop practical strategies for improving economic opportunities. Key challenges included a lack of entrepreneurial skills, low and unstable earnings, and limited access to productive resources such as land and capital, which constrained women’s ability to secure gainful work or start small businesses. Through facilitated reflection and group problem-solving, participants and researchers mapped potential income sources, including skill development activities and local enterprise opportunities. The PAR process not only generated contextually relevant livelihood options, but also strengthened women’s capacities for collective action and critical analysis of structural constraints. The study highlights the importance of participatory, experiential methods for empowering rural women, building their economic capabilities, and informing policies and program aimed at sustainable community development. Findings are relevant for policymakers, civil society organizations, and academic researchers seeking to enhance gender-equitable livelihood outcomes in rural contexts. |
| S034 | Lee, J. Y., Clark, J. K., Schmiesing, R., Kaiser, M. L., Reece, J., & Park, S. (2024). Perspectives of community members on community-based participatory research: A systematic literature review. Journal of Urban Affairs, 47(8), 2916–2935.  <https://doi.org/10.1080/07352166.2024.2305132> | This participatory action research study explored the livelihood challenges faced by rural women in Nagla Chandi, a village in Uttar Pradesh, India, and collaboratively developed context-specific solutions with participants. The research encompassed two local self-help groups and engaged women in identifying the structural, social, and economic barriers limiting their income-earning opportunities. The key challenges highlighted by participants included insufficient entrepreneurial skills, irregular and low earnings, limited access to productive assets like land and capital, and gendered constraints that affected participation in local markets and decision-making. The PAR process enabled women to reflect on lived experiences, assess local resources, and co-design practical strategies to strengthen livelihoods. These strategies included skill development initiatives, collective enterprises, network building for market access, and measures to improve negotiation power within households and community institutions. Importantly, the research process itself fostered empowerment, collective agency, and critical awareness, helping participants to articulate their priorities and influence local stakeholders. The authors argue that participatory methodologies are crucial for generating sustainable, culturally grounded livelihood solutions and for informing policy interventions that enhance gender-equitable economic development in rural settings. |
| S035 | Thomas E, Benjamin-Thomas TE, Sithambaram A, Shankar J, Chen S-P. Participatory Action Research Among People With Serious Mental Illness: A Scoping Review. Qualitative Health Research. 2023;34(1-2):3-19.  doi:10.1177/10497323231208111 | This scoping review examined how participatory action research (PAR) has been conducted with people with serious mental illness (PSMI) and identified strategies used to engage them as co-researchers in research aimed at achieving social change. PAR is an approach that centers collaboration between researchers and people traditionally marginalized in research, not only to study issues but also to contribute actively to solutions. The authors systematically mapped the empirical studies involving PSMI in roles beyond mere participants, highlighting how PAR supported capacity building, relationship development, and empowerment among team members with lived experience. Engaging PSMI in PAR was linked to enhanced skill development, such as research literacy and advocacy, and fostered connections with stakeholders in communities seeking change. This review described various methods and levels of involvement, from consultation to shared decision-making, and emphasized the importance of structuring research processes to genuinely include experiential knowledge. It also discussed barriers, such as power imbalances, resource constraints, and institutional challenges that can limit meaningful participation. The authors finally concluded that PAR holds promise for producing contextually relevant evidence while promoting agency among PSMI, and they called for more research to capture diverse strategies and impacts of such co-researcher engagement. |
| S036 | Ramanadhan S, Alemán R, Bradley CD, Cruz JL, Safaeinili N, Simonds V, Aveling EL. Using Participatory Implementation Science to Advance Health Equity. Annu Rev Public Health. 2024 May;45(1):47-67. doi: 10.1146/annurev-publhealth-060722-024251. Epub 2024 Apr 3. PMID: 38109515; PMCID: PMC11251496.  DOI: 10.1146/annurev-publhealth-060722-024251 | This review article articulates how participatory implementation science (IS) can be used to advance health equity by transforming how evidence-based interventions (EBIs) are implemented and sustained in public health and healthcare settings. The authors argue that traditional IS approaches often focus primarily on making interventions work within existing systems without sufficiently addressing the structural inequities that shape health outcomes. Participatory IS, by contrast, involves collaborative partnerships among academic researchers, community members, practitioners, and other stakeholders to co-produce knowledge and action that reflect community needs and values. This review outlines a rationale for integrating participatory principles—such as inclusion, shared decision-making, and iterative learning—into IS frameworks to ensure that implementation activities are responsive to the contexts of underserved populations. It also presents a conceptual framework for combining participatory methods with IS theories and methods, and discusses critical considerations for enhancing both the practice and impact of participatory IS. Ultimately, the authors contend that participatory approaches have the potential to move beyond incremental adaptation of EBIs within inequitable systems toward transformative change that reshapes systems to center equity and improve health for marginalized groups. |
| S037 | Bogerd, M.J., Slottje, P., Bont, J. et al. Development of a person-centred care approach for persons with chronic multimorbidity in general practice by means of participatory action research. BMC Prim. Care 25, 114 (2024).  <https://doi.org/10.1186/s12875-024-02364-x> | This study used participatory action research (PAR) to co‑create and pilot a proactive, person‑centered chronic care approach for patients with multimorbidity in general practice, aiming to improve care coordination, patient experience, and provider satisfaction. Traditional single-disease care models commonly fragment care for people with multiple chronic conditions. In response, the research partnered general practitioners (GPs), practice nurses, patients, insurers, and researchers across 13 Dutch practices in four iterative co‑creation cycles from 2019 to 2021 to design, test, reflect on, and refine a person‑centered approach. The resulting model centered on an extended person‑centered consultation where more time was devoted to understanding patients’ priorities, context, goals, and care preferences. Key facilitators included enhanced therapeutic relationships, improved work satisfaction for providers, and patients’ appreciation of longer consultations. Implementation challenges included identifying which patients would benefit most, ensuring appropriate remuneration and time allocation, training care providers in person‑centered methods, and adapting electronic medical records to support comprehensive documentation. The authors conclude that while the co‑created model shows promise for enhancing care for people with multimorbidities, several barriers must be addressed before broader implementation and formal evaluation against patient and system outcomes. |
| S038 | Carrick, J., Bell, D., Fitzsimmons, C., Gray, T., & Stewart, G. (2022). Principles and practical criteria for effective participatory environmental planning and decision-making. Journal of Environmental Planning and Management, 66(14), 2854–2877.  <https://doi.org/10.1080/09640568.2022.2086857> | This article examined the gap between the ideals and practice of participatory environmental planning and decision‑making, identifying why participatory processes often fail to deliver meaningful outcomes for stakeholders and communities. While participation is widely promoted as a method of legitimizing decisions and improving environmental governance, many real-world efforts leave participants feeling frustrated, disillusioned, or excluded, undermining trust and fueling opposition that hinders planning processes. To address this disconnect, the authors synthesized insights from science and technology studies, procedural environmental justice, and deliberative democracy to clarify the values and commitments that underlie genuinely effective participation. They propose a framework of principles—including inclusivity, reflexivity, empowerment, and a process orientation—that articulates what participatory engagement should strive for in environmental contexts. These principles are subsequently translated into practical criteria that planners and decision‑makers can apply to design and evaluate participatory processes, ensuring that ideals such as fairness, accessibility, and influence in decision outcomes are realized in practice. By linking participatory theory with actionable guidance, the article aims to strengthen both the legitimacy and effectiveness of environmental planning and decision-making. |
| S039 | Kwamie A, Causevic S, Tomson G, Sie A, Sauerborn R, Rasanathan K, et al. Prepared for the polycrisis? The need for complexity science and systems thinking to address global and national evidence gaps. BMJ Global Health. 2024;9:e014887.  <https://doi.org/10.1136/bmjgh-2023-014887> | This article argues that the world is facing a poly-crisis—involving a convergence of multiple, interdependent global threats including climate change, conflict, pandemics (including the lasting effects of COVID‑19), and other systemic shocks—that undermine progress toward the Sustainable Development Goals (SDGs). The authors contend that existing global and national research evidence is predominantly descriptive and correlational, failing to capture the complex, non‑linear interactions among these overlapping crises. As a result, policy guidance and interventions are often unable to address systemic risks comprehensively or anticipate cascaded effects across sectors and scales. To meet this challenge, the authors advocate for greater application of complexity science and applied systems thinking, which provide conceptual and methodological tools to understand and model interactions within complex systems. They emphasize the need for participatory research processes that involve diverse stakeholders in defining system boundaries and behaviors and the use of analytical methods capable of hypothesizing, modelling, visualizing, and testing complex system properties over time. Strengthening such approaches, particularly in contexts most affected by poly-crisis, can generate evidence that better informs policy and supports more effective, adaptive responses to interconnected global threats. |
| S040 | Rusoja E, Haynie D, Sievers J, Mustafee N, Nelson F, Reynolds M, Sarriot E, Swanson RC, Williams B. Thinking about complexity in health: A systematic review of the key systems thinking and complexity ideas in health. J Eval Clin Pract. 2018 Jun;24(3):600-606.  doi: 10.1111/jep.12856. | This systematic review explored how systems thinking and complexity science (ST/CS) concepts are discussed and applied in health research and practice, particularly in relation to efforts such as implementing the Sustainable Development Goals (SDGs). Recognizing that health and healthcare systems are complex adaptive systems composed of interconnected actors and dynamic relationships, the authors searched the published literature from 2002 to 2015 to identify key terms, concepts, and methods from ST/CS that have been used in health contexts. They found that the use of terminology varied widely, with common concepts including ‘*dynamic systems’*, ‘*emergence’*, ‘*complex adaptive systems’*, and ‘*interdependence’*, while methods such as systems dynamics modelling and agent‑based modelling appeared less frequently. Although interest in ST/CS has grown over time—particularly in journals focused on medicine and healthcare—the literature remains fragmented and heavily theoretical, with relatively limited empirical application. The authors conclude that while ST/CS offers valuable perspectives for understanding and intervening in complex health challenges, a lack of cohesive conceptualization and practical application limits its influence on health policy and practice. They call for more explicit use of these approaches to improve implementation strategies and address complex health problems in research and practice. |
| S041 | Andonian BJ, Hippensteel JA, Abuabara K, Boyle EM, Colbert JF, Devinney MJ, Faye AS, Kochar B, Lee J, Litke R, Nair D, Sattui SE, Sheshadri A, Sherman AN, Singh N, Zhang Y, LaHue SC. Inflammation and aging-related disease: A transdisciplinary inflammaging framework. Geroscience. 2025 Feb;47(1):515-542.  doi: 10.1007/s11357-024-01364-0 | This review synthesizes the current evidence on inflammaging, a chronic, low-grade inflammatory state that increases with age and contributes to multiple aging-related diseases and adverse outcomes. The authors highlight how persistent inflammation underlies conditions such as cardiovascular disease, frailty, neurodegeneration, metabolic disorders, and functional decline, framing inflammaging not merely as a biomarker of aging but as a mechanistic driver of pathophysiology. Despite its clinical importance, inflammaging remains poorly defined and inconsistently measured across research, limiting its integration into the field of geroscience and clinical practice. To address this gap, a transdisciplinary group of clinician‑scientists from nine medical subspecialties combined insights from geriatrics, immunology, neurology, dermatology, and other fields to propose a comprehensive framework that connects cellular senescence, immune dysregulation, and systemic inflammation with organ‑specific disease processes. The framework emphasizes the need for integrated, multi‑level research that spans molecular pathways, clinical phenotypes, and patient‑centered outcomes to understand how inflammaging emerges and how interventions might mitigate its effects. The article calls for a broader adoption of transdisciplinary methods and consensus on definitions, measurements, and intervention targets to advance both research and care for aging populations. |
| S042 | Wan TTH, Wan HS. Predictive Analytics with a Transdisciplinary Framework in Promoting Patient-Centric Care of Polychronic Conditions: Trends, Challenges, and Solutions. AI. 2023; 4(3):482-490.  <https://doi.org/10.3390/ai4030026> | This article examined the application of predictive analytics within a transdisciplinary framework to enhance patient‑centric care for individuals with polychronic conditions (defined as patients who simultaneously manage multiple chronic diseases). The authors argue that conventional analytical models often function as opaque “black boxes,” limiting clinicians’ ability to interpret how predictions are generated and hindering their integration into complex care settings. To address this, the paper advocates for combining advanced predictive modeling with transdisciplinary collaboration among clinicians, data scientists, and health system stakeholders to improve decision‑making, personalize interventions, and anticipate patient trajectories more effectively. Central trends include leveraging large‑scale health data and algorithmic methods to identify risk patterns and disease progression across conditions, while challenges involve balancing model complexity with interpretability, managing heterogeneous data sources, and ensuring clinical relevance. Proposed solutions emphasize the development of explanatory analytics, theory-driven model design, and iterative validation with real-world clinical data to move beyond purely inductive problem‑solving toward deductive, evidence-based decision support. Ultimately, the paper highlights that integrating predictive analytics into patient care requires not only technical refinement but also structural and collaborative systems that bridge disciplinary boundaries to promote holistic, patient‑centered outcomes. |
| S043 | Archibald, M.M., Lawless, M.T., de Plaza, M.A.P. et al. How transdisciplinary research teams learn to do knowledge translation (KT), and how KT in turn impacts transdisciplinary research: a realist evaluation and longitudinal case study. Health Res Policy Sys 21, 20 (2023).  <https://doi.org/10.1186/s12961-023-00967-x> | This realist evaluation and longitudinal case study examined the reciprocal relationship between transdisciplinary research collaboration and knowledge translation (KT), using a five‑year Centre of Research Excellence focused on frailty as the case. Transdisciplinary research—collaboration across disciplines to address complex “wicked” problems—is assumed to enhance research impact, while KT promotes the application of research evidence in practice and policy. However, how these two processes influence one another has been unclear. Through interviews, observations, document review, and visual elicitation with team members over three phases, the authors identified three programme theories describing mechanisms through which transdisciplinary teams come to understand and enact KT. Findings show that when contextual supports (such as facilitation, distributed leadership, and explicit frameworks) are present, team members increasingly interpret and enact KT as a collaborative, networked, and iterative process. Consequently, transdisciplinary collaboration improves by enabling individuals to better navigate, negotiate, and mobilize networks of relationships and knowledge flows. The study highlights that embedding KT capacity and structures within transdisciplinary research teams enhances both collaborative processes and the practical application of research, suggesting that KT and transdisciplinary practice are mutually reinforcing rather than linear steps in research impact. |
| S044 | Salerno, J., Aguirre, A. A., Aguirre, L. F., et al. (2025). Transdisciplinary research priorities for a One Health approach to human–wildlife coexistence. BioScience, biaf026.  <https://doi.org/10.1093/biosci/biaf026> | This viewpoint paper identifies the transdisciplinary research priorities needed to advance a One Health approach to human–wildlife coexistence. The authors argue that accelerating biodiversity loss, zoonotic disease risk, and escalating conflicts between humans and wildlife are interconnected challenges that cannot be addressed within any single discipline or sector. They propose a research agenda that integrates ecological, veterinary, public health, and social sciences, alongside the knowledge and participation of local communities and Indigenous peoples. Key priorities include understanding how social, cultural, economic, and political drivers shape human–wildlife interactions; co-developing context-specific strategies that simultaneously promote conservation, livelihoods, and health; and strengthening governance arrangements that cut across environmental, agricultural, and health institutions. The paper emphasizes that One Health must move beyond narrow zoonotic risk framing toward a broader, equity-focused lens on coexistence, calling for funding, training, and institutional reforms that support long-term, participatory, and solutions-oriented transdisciplinary research. |
| S045 | Shrivastava P, Stafford Smith M, O'Brien K, Zsolnai L. Transforming Sustainability Science to Generate Positive Social and Environmental Change Globally. One Earth. 2020 Apr 24;2(4):329-340.  doi: 10.1016/j.oneear.2020.04.010 | This article critiques the current state of sustainability science, arguing that, despite decades of research and policy engagement, it has not brought humanity significantly closer to global sustainability targets such as those embodied in the UN Sustainable Development Goals. The authors contend that sustainability science has been overly dominated by natural science perspectives, and has not fully addressed the social, cultural, institutional, and behavioral dimensions of global environmental challenges. To be more impactful, sustainability science must evolve into a transdisciplinary enterprise integrating the natural sciences with social sciences, humanities, and the arts, enabling richer understanding and co‑creation of knowledge that addresses complex socio‑ecological systems and human influences on the environment. The article calls for renewed research and public policies to reshape the research ecosystem within universities, funding bodies, science communication, policymaking, and decision‑making systems so that research is more engaged with society and oriented toward real‑world change. It also emphasizes the importance of engaging diverse sources of knowledge—including stakeholder and community perspectives—to drive positive social and environmental transformations at local and global scales beyond incremental improvements. |
| S046 | Sather, E.W., Iversen, V.C., Svindseth, M.F. et al. Exploring sustainable care pathways - a scoping review. BMC Health Serv Res 22, 1595 (2022).  [doi.org/10.1186/s12913-022-08863-w](https://doi.org/10.1186/s12913-022-08863-w) | This article examined how interprofessional care pathways can improve professional attitudes towards mental healthcare. Key factors for efficient and effective practice include better communication, flexibility, patient-centered approaches, staff supervision, and increased support within primary care. The authors identify stigma and discrimination as major barriers to equal mental health service delivery, with stigma processes affecting help-seeking behaviors among individuals experiencing first-episode psychosis and those at risk. A lack of information may heighten perceived stigma. Through a review of the literature, this article highlights important themes such as organization, resources and outcomes, information and documentation, participation from patients and families, clinical care and teamwork, and ethical considerations. Respectful communication plays a crucial role in preventing patient humiliation and supporting treatment adherence. Integrating both professional and patient perspectives leads to improved outcomes from sustainable care pathways. The authors recommend clear guidelines for treatment pathways, including: establishing transparent responsibilities and procedures, utilizing information technology to objectively enhance patient results, providing structured information and documented plans to patients when needed, promoting ongoing collaborative decision-making, ensuring collaboration among mental health and other professionals to address patient needs, and maintaining ethical standards through respectful, patient-centered communication and care. |
| S047 | Cardol, C. K., Tommel, J., van Middendorp, H., Ciere, Y., Sont, J. K., Evers, A. W., & van Dijk, S. (2021). Detecting and treating psychosocial and lifestyle-related difficulties in chronic disease: development and treatment protocol of the E-GOAL eHealth care pathway. International journal of environmental research and public health, 18(6), 3292.  doi.org/10.3390/ijerph18063292 | This paper describes how an eHealth care pathway was systematically developed using evidence-based practices to help patients with lifestyle-related chronic diseases identify and address psychosocial and lifestyle-related challenges. This approach prioritizes patient participation and equity by incorporating their experiences throughout the process. Using a combination of established frameworks and co-creation methods, the team designed a screening tool and self-management modules tailored for both the overall target group and individual needs. The authors highlight that many individuals with such chronic conditions struggle to maintain healthy habits due to psychosocial barriers. The goal of this study was thus to create an eHealth solution that detects and treats these issues in a manner that fits each patient's preferences across different chronic diseases. Each part of the intervention started with scientific evidence and the Behavior Change Wheel, then evolved through feedback from patients and health professionals to better meet users' needs. In the finalized pathway, patients fill out short online questionnaires to uncover psychosocial and lifestyle risks, which are presented as personalized charts. Those at increased risk receive additional questionnaires, helping to customize a 3-month guided online cognitive behavioral therapy program to suit their priorities and goals. Progress is tracked with the screening tool. This methodical and collaborative development led to a personalized eHealth pathway that supports patients in overcoming mental barriers and adopting healthier lifestyles. Before being widely used, randomized controlled trials will test its cost-effectiveness and impact on psychosocial, lifestyle, and health outcomes. While the study explores community-inclusive and participatory methods, it does not directly address transdisciplinary science. It is especially useful in its exploration of chronic illness treatment pathways. |
| S048 | Villani, C., Sapio, M., Cassarà, G., Patti, Y., Cupane, L., Giorgi, V., ... & Sarzi-Puttini, P. (2025). Pharmakon or the healing art: experience of artistic-transformative transdisciplinary workshops in fibromyalgia syndrome. Clinical and Experimental Rheumatology, 43, 978-989.  doi: 10.55563/clinexprheumatol/yxhmcr. | This study examined the validation of the efficacy of transdisciplinary artistic-transformative interventions in improving quality of life, sleep, self-esteem, and self-efficacy among patients with fibromyalgia. The transdisciplinary methodology specifically incorporated transformative art as a central component. The authors report that transformative experiences facilitate significant and immediate changes, which help to disrupt maladaptive emotional and behavioral cycles induced by chronic stress and trauma. A total of 109 individuals with fibromyalgia participated in an eight-month observational study assessing the effectiveness of three online transdisciplinary artistic-transformative workshops. The Group 1 participants engaged in reviewing their autobiography and illness narratives from a humorous perspective; conversely, Group 2 participants were guided to express their experiences of illness through poetry; and Group 3 focused on the guided narration of artwork using visual thinking strategies integrated with principles of narrative medicine. Assessments were conducted at baseline and post-intervention. No baseline differences were observed among the groups regarding clinimetric variables. Collectively, all three groups demonstrated statistically significant improvement in the Pittsburgh Sleep Quality Index (PSQI), Response to Stressful Experiences Scale (RSES), WHO-5 Well-Being Index (WHO-5), and Global Health scale (GH). No significant change was reported for the Mindful Attention Awareness Scale. Group 1 exhibited improvements across nearly all parameters. In Group 2, enhancements were noted in sleep and the third dimension of the SAP, while Group 3 showed gains in self-esteem and the WHO-5 index. These findings suggest that transformative art, implemented through transdisciplinary approaches, serves as a key mediator in overcoming trauma and stressors—possibly by generating "pivotal mental states" that enable rapid, profound learning and psychological transformation. This research indicates that art, when applied via a transdisciplinary framework, contributes to significant improvements in the psychophysical condition of fibromyalgia patients. |
| S049 | Nguyen, P., & Gietzen, L. (2024). Transdisciplinary Collaboration of Healthcare Professionals and the Emotional Connectedness with Working with Patients Coping with Death and Dying. Pacific Journal of Health, 7(1), 35.  [doi.org/10.56031/2576-215X.1085](https://doi.org/10.56031/2576-215X.1085) | Utilizing a qualitative methodology, this study examined how healthcare professionals communicate within their disciplines and interact with patients confronting death and dying across various settings. This study investigated the implementation of integrated transdisciplinary principles in healthcare practice and considers how care for patients and families is facilitated by transdisciplinary teams. The authors posit that this approach enables patient-centered care, supporting quality of life during chronic illness and at the end of life. They highlight that, while there is an established understanding of collaborative care and transition processes to assist patients and families in coping with and communicating about death and dying, it remains critical to determine optimal methods for providing end-of-life care and maintaining quality of life through challenging conversations regarding the transition from active intervention to palliative and ultimately end-of-life care. The article underscores that discussions surrounding impending death or dying are inherently complex and demanding for healthcare professionals, regardless of the clinical context.  The research phase involved confidential, single, in-depth semi-structured interviews with sixteen medical professionals operating in diverse interdisciplinary environments. A constructivist grounded theory approach guided the qualitative data analysis, employing carefully designed open-ended questions with a Likert scale to elicit comprehensive free-text responses. Findings are categorized into five primary themes: the necessity of care planning and coordination, demand for additional resources in both community and workplace settings, importance of developed strategies for mental well-being, standards of care in professional environments, and the need for enhanced preparedness through education and training. The authors conclude that effective collaboration among healthcare professionals in matters related to death and dying fosters a continuum of care characterized by improved communication, coordination, and planning for patients and families experiencing difficult circumstances. The clear comprehension of patient preferences and values at the end of life is imperative to deliver high-quality care. The competencies of transdisciplinary teams—including those of individual healthcare professionals—contribute to thorough assessment, prioritization of diagnoses, and collaborative determination of interventions. Interprofessional collaboration extends beyond educational background and clinical expertise, requiring substantive engagement with teamwork dynamics. Communication regarding approaches to death and dying is recognized as multifaceted and challenging on both personal and professional levels. As teamwork becomes increasingly integrated within healthcare settings, inter-agency and transdisciplinary collaborations are becoming essential for reducing medical errors, enhancing patient quality of life, and potentially alleviating team stress through streamlined coordination. The authors further investigate the application of transdisciplinary principles in these contexts. |
| S050 | Lassell, R. K., Pritchard, K., Baehr, L. A., Ceïde, M. E., Chatterjee, S. A., Colón-Semenza, C., ... & Andonian, B. J. (2025). New horizons in advancing lifestyle medicine for older adults utilizing a transdisciplinary approach: gaps and opportunities. Age and ageing, 54(6), afaf159.  [doi.org/10.1093/ageing/afaf159](https://doi.org/10.1093/ageing/afaf159) | This article discusses a new approach to encourage healthy lifestyle behaviors among older adults by using a transdisciplinary framework. The authors note that many older adults do not follow recommended health guidelines and suggest including individuals' lived experiences within the change process. Their tailored lifestyle medicine framework considers the complex needs and contexts of older adults, integrating factors like social determinants of health, built environment, the 5 M’s of geriatric medicine (Mind, Mobility, Medications, Multi-complexity, Matters Most), and the six pillars of lifestyle medicine (nutrition, physical activity, restorative sleep, stress management, avoiding risky substances, and positive social connections). This innovative approach weaves these elements into efforts to promote change. The article highlights key gaps and opportunities for advancing practice, research, and policy in lifestyle medicine, aiming to support lasting behavioral changes and improve the overall health span of older adults through the innovative potential of transdisciplinary principles which the authors explore. |
| S051 | Martins, P., Matos, L., Moreira, A., Simoes, M., & Silva, B. (2025). A Transdisciplinary Approach of the Role of Consciousness and Spirituality in Physics and Healing. Transdisciplinary Journal of Engineering & Science, 16.  [doi.org/10.22545/2025/00273](https://doi.org/10.22545/2025/00273) | The authors assessed transdisciplinary research principles and propose that these may represent a significant paradigm shift in both medical and healing practices. This paper seeks to emphasize the influence of consciousness and spirituality within physics, medicine, and healing methodologies. Accordingly, it reviews classical healing approaches, including the therapeutic efficacy of Chinese medicine in mitigating disease symptoms. Contemporary healing methods were also examined and contextualized with the concept of “spontaneous healing,” a phenomenon not currently explained by conventional medicine. In this context, morphogenic fields were discussed as a potential mechanism underlying the effectiveness of Chinese medicine. The paper expands upon themes from the article titled “The usefulness of integrative medicine in contemporary society,” published in the Transdisciplinary Journal of Engineering & Science (TJES). Additionally, altered, modified, and amplified states of consciousness are thoroughly described as possible explanations for spontaneous healing occurrences. The concept of the biofield is linked to the innovative healing paradigms introduced by the integrative medicine model. The paper critically evaluated spiritual, medical, and physical phenomena through the framework of transdisciplinary science. |
| S052 | McLure, J., Arnautovska, U., Panizzutti, B., Jiang, J. C., Robinson, S., Drummond, K. D., ... & Berk, M. (2025). New hope for individuals with schizophrenia: A transdisciplinary research platform to discover and translate novel treatments. Australian & New Zealand Journal of Psychiatry, 00048674251342959.  [doi.org/10.1177/00048674251342959](https://doi.org/10.1177/00048674251342959) | This study examined how a transdisciplinary research platform, such as the Development of Innovative Clinical Treatments in Schizophrenia (SYNDICAT), applies both transdisciplinary and multidisciplinary approaches to foster innovation. The authors believe that collaboration across disciplines can advance the creation of new, safer, and more effective schizophrenia medications with fewer side effects—which is an important goal for patients and caregivers. Such collaboration also aims to responsibly inspire hope and support individuals living with schizophrenia, as well as their families and communities. Over at least five years, a multidisciplinary team will work closely with experts who have lived experience of schizophrenia to identify, assess, and test improved treatments that can reduce the suffering associated with this condition. Although schizophrenia commonly results in significant personal, societal, and economic challenges, it may also lead to transformative experiences and new opportunities for personal growth and healing. Lack of effective treatment options is considered a major obstacle to recovery, hope, and progress for both individuals and communities. However, insights from lived experience suggest that, when hope exists, schizophrenia can become a catalyst for positive change and self-improvement. |

**Supplementary Table S18. GRADE-CERQual Assessment of Individual Transdisciplinary Studies***

| Study Id | Title | Methodological limitations | Coherence | Adequacy | Relevance |
| --- | --- | --- | --- | --- | --- |
| S001 | Yang X, Lo K. Environmental health research and the COVID-19 pandemic: A turning point towards sustainability. Environ Res. 2021 Jun;197:111157. doi: 10.1016/j.envres.2021.111157. Epub 2021 Apr 20. PMID: 33887273; PMCID: PMC8542966.  doi: 10.1016/j.envres.2021.111157 | Moderate: The authors used a systematic selection method and a comprehensive search strategy across major databases. However, the study is a review of rapidly evolving data from the first year of the pandemic, and the provided text does not mention a formal risk-of-bias assessment for the included literature. | High: The findings are strongly supported by a structured theoretical framework (Fig. 1) integrating society, environment, and health. The conclusions and guiding principles are logically derived from consistent data across multiple studies, such as those regarding air quality and transmission. | Moderate: The review incorporates a wide range of peer-reviewed scientific publications. However, the data richness is limited by the timing of the study; the authors explicitly state it is “premature to conclude” on long-term net environmental effects because the data only covers up to February 2021. | High: The research is highly applicable to the specific context of environmental health and sustainability during a global crisis. Every cited study is directly related to the intersection of COVID-19 and environmental health, and the findings are translated into actionable principles for professionals. |
| S002 | Fischer, C., Gugerell, K., Laa, U. et al. Evaluating transdisciplinary methods: a new scale for measuring knowledge integration. Humanit Soc Sci Commun12, 1395 (2025).  doi.org/10.1057/s41599-025-05634-w | Moderate: The study utilizes a rigorous two-phase development process involving a systematic review of 48 sources and advanced statistical techniques like Exploratory Factor Analysis (EFA) and parallel analysis. However, the authors explicitly acknowledge significant limitations: a small sample size (n=71), a lack of full sample independence as some participants attended multiple workshops, and an observed response bias where participants tended toward extreme ratings regardless of content. | High: The findings are strongly aligned with the underlying data. Although the study began with four theoretical dimensions, the empirical results (EFA and parallel analysis) consistently pointed to two distinct factors. The authors logically explain this shift by addressing the "gray spaces" and overlaps in existing literature, successfully synthesizing these findings into a new "iceberg model" of knowledge integration. | Moderate: The researchers derived 327 statements from a rich pool of 48 literature sources to build the scale. While the qualitative data are deep, the authors nevertheless note that the empirical sample size is limited, stating that a larger sample is required in the future to improve the reliability and "test power" of the scale for broader application. | High: The study directly addresses a documented gap in transdisciplinary research: the lack of standardized instruments to measure knowledge integration. The scale was tested in a real-world, high-stakes context (sustainable beef and dairy supply chains) and provides actionable “practical implications” for researchers and facilitators to improve the selection of transdisciplinary methods. |
| S003 | de Jager, L.A., Bal, M., Baudena, M. et al. Transdisciplinary complexity science: deepening system understanding for sustainability. Humanit Soc Sci Commun 12, 1384 (2025).  <https://doi.org/10.1057/s41599-025-05548-7> | Moderate: The authors followed a rigorous, expert-led process guided by PRISMA framework. However, the search was limited to a single database (Scopus) and strictly excluded relevant research that did not explicitly use the term “sustainability,” potentially missing significant related work in social-ecological systems. Additionally, the authors note that the primary studies they reviewed often lacked formal evaluation or systematic empirical validation. | High: The findings are strongly supported by the underlying data extracted from 56 identified articles. The authors successfully synthesized these diverse studies into a cohesive conceptual framework (Fig. 1) and a phase-by-phase recommendation model (Fig. 3) that logically aligns with the documented challenges and successes found across the reviewed literature. | Moderate: This study is based on an “emerging field,” with a limited body of research. While the review is thorough, many Sustainable Development Goals (SDGs) are underrepresented or entirely unstudied within this intersection of methods. The “richness” of the findings is constrained by the relatively small sample of 56 papers and the gaps in the primary data regarding efficacy. | High: The review is highly specific to its core question: how quantitative complexity science and transdisciplinary methods can be integrated for sustainability. Every article included was required to meet strict criteria for both methodologies and an SDG focus. The resulting “best practices” and “recommendations” are directly applicable to the specific context of the review question. |
| S004 | Wouters, R., Cox, A. & Raes, A. Co-producing knowledge in and about education by combining collaborative ethnography and abductive reasoning. Humanit Soc Sci Commun 12, 1405 (2025).  <https://doi.org/10.1057/s41599-025-05721-y> | Moderate: The study is transparent about its iterative design, combining collaborative ethnography and abductive reasoning. However, it relies on a purposive sampling of observers based on pre-existing professional relationships (“collegial friends”), which may introduce bias. Additionally, the fieldwork was confined to a single school campus, and the researchers initially failed to include the pupils’ perspectives, a limitation later pointed out by the participating teachers. | High: The findings are strongly supported by the underlying data through a systematic eight-phase process. The study utilizes member-checking by presenting a SWOT analysis to the school team, who confirmed that the observations accurately reflected their practices. The “abductive” framework ensures that surprising observations are logically connected to both empirical evidence and theoretical literature. | High: The data are exceptionally rich, comprising 290 pages of observation notes from 32 distinct observers with varied expertise, as well as 42 hours of recorded interviews with both observers and teachers. The use of CAQDAS software (Atlas.ti) to manage and annotate this diverse dataset (text, sketches, and audio) further enhances the quality and depth of the findings. | High: The study is directly applicable to the research questions regarding the phases and essential considerations of a transdisciplinary approach in education. It successfully translates a complex methodological journey into a sequence organizer and actionable insights for future projects aiming to address grand societal challenges like inclusive education. |
| S005 | Vienni-Baptista, B., & Pohl, C. E. (2024). Exploring interdisciplinarity and transdisciplinarity as knowledge regimes: A heuristic tool for disentangling understandings in academia and policy. Science, Technology, & Human Values, 49(6), 1309-1348.  <https://doi.org/10.1177/01622439231216789> | Moderate: The study employs a robust two-stage design: a systematic meta-ethnography followed by an iterative refinement stage involving workshops and interviews. However, the authors explicitly acknowledge a significant limitation: the tool was tested primarily with researchers and funders, while very few policymakers engaged in the actual tests. This gap in the target audience limits the empirical evidence for the tool's effectiveness in policy settings. | High: The findings are logically and strongly supported by the underlying “knowledge regime” framework. There is a clear, consistent progression from the six qualitative dimensions identified in the literature to the final ten guiding questions of the heuristic tool. The results of the stakeholder tests directly reinforced the dimensions originally derived from the literature review. | High: The richness and quantity of data are substantial. The initial identification stage screened 5,060 records, resulting in a deep content analysis of 205 core academic and policy documents. This was supplemented by qualitative data from two workshops, a master class, nine semi-structured interviews, and multiple conference presentations. This broad evidence base ensures that the tool is grounded in both theory and practice. | High: The study is highly relevant to the core problem of “disconnection” between academic and policy discourses on interdisciplinarity (ID) and transdisciplinarity (TD). The tool directly addresses the urgent policy call for better integration methods and provides actionable pathways for researchers, funders, and policymakers to align their goals and expectations. |
| S006 | Yang, L., Shewchuk, B., Shang, C., Lee, J. A., & Gehlert, S. (2023). Transdisciplinary team science in Health Research, where are we?. Journal of Integrated Design and Process Science, 26(3-4), 307-316.  <https://doi.org/10.3233/JID-220011> | Moderate: The authors used a systematic search of the NIH RePORTER module with clear inclusion and exclusion criteria. However, they acknowledge that the term “transdisciplinary” may have appeared in grant relevance statements, without being a key component of the actual research methods. Furthermore, the study relies on investigator self-identification, which may not always align with theoretical definitions of transdisciplinarity. | High: The findings are strongly supported by the extracted data; for example, the claim that transdisciplinary research is difficult to define and evaluate is directly evidenced by the fact that only one out of 25 projects actually included a definition of the term in its abstract. The proposed frameworks (like TASKS) are logically connected to the identified needs for shared mental models. | High: The researchers conducted a comprehensive screen of 6,617 NIH/NCI-funded results, narrowing them down through a multi-stage process to 25 unique, relevant projects. This large-scale initial dataset from a primary government repository ensures a high quantity and quality of data regarding the status of federally funded transdisciplinary health research. | High: The data are entirely applicable to the review question regarding the current status of transdisciplinary team science in health research. By using cancer research as a primary example, a field where the NCI has been a leader in funding such initiatives, the authors provide a highly relevant snapshot of the challenges and progress in the field. |
| S007 | Van der Vlegel-Brouwer, W., Eelderink, M., & Bussemaker, J. (2023). Participatory Action Research as a Driver for Health Promotion and Prevention: A Co-creation Process Between Professionals and Citizens in a Deprived Neighbourhood in the Hague. International journal of integrated care, 23(4), 13.  doi.org/10.5334/ijic.7560 | Moderate: The study follows a rigorous Look-Think-Act Participatory Action Research (PAR) cycle. However, the authors identify several limitations: the research was conducted over a short timeframe (7 months), which hindered the ability to build long-term trust in a neighborhood where residents “come and go.” There was also a documented power imbalance, as professionals sometimes “took over” initiatives from citizens, disrupting the development of ownership. | High: The findings are strongly supported by the data and internal validation processes. The seven themes (e.g., Healthy Money, Healthy Mind) were derived from thematic analysis and then presented back to participants in focus groups for dialogue and reflection. This ensured that the resulting eight co-created initiatives were logically and consistently aligned with the needs expressed by the community. | High: The data are exceptionally rich and diverse, comprising 60 in-depth interviews (37 citizens and 23 professionals) and logbook notes from 7 fieldwork days in varied locations like schoolyards and food banks. Additionally, 11 focus group meetings were held to refine these perspectives. The inclusion of direct narratives provides deep insight into the complex, “wicked” social problems of the neighborhood. | High: The research is entirely applicable to the review question of how co-creation and PAR can drive health promotion in deprived areas. It specifically targets Moerwijk, a neighborhood with statistically high health risks, successfully translating the findings into concrete policy and practice recommendations for bridging the gap between the “system world” and the “living world.” |
| S008 | Ortwin Renn, Transdisciplinarity: Synthesis towards a modular approach:  Futures, Volume 130, 2021,102744,ISSN 0016-3287  [doi.org/10.1016/j.futures.2021.102744](https://doi.org/10.1016/j.futures.2021.102744) | Moderate: The paper is a conceptual synthesis, rather than a primary empirical study. The author explicitly notes that the work is not a systematic review based on a formal search methodology but a synthesis of existing literature describing the nexus between science and practice. While it effectively critiques and builds upon established “traditions,” the lack of any reproducible search protocol is a limitation common to theoretical frameworks. | High: The findings are strongly supported by the underlying analysis of existing transdisciplinary schools. The proposed “modular approach” is logically derived to address the specific “shortcomings” and “pitfalls,” such as the tension between scientific rigor and advocacy, identified in earlier concepts. The internal logic of mapping knowledge types to specific research functions (Table 4) is highly consistent. | High: The study draws upon a rich and extensive body of literature spanning over three decades of transdisciplinary research. It integrates diverse perspectives from post-normal science, sustainability science, and socio-ecological traditions. Furthermore, the inclusion of a detailed real-world case study (BEKO project), which received a national prize for excellence, provides high-quality qualitative data to support the practical feasibility of the framework. | High: The paper is entirely applicable to the review question of how to integrate scientific expertise into policymaking for societal transformations. It specifically addresses “wicked” and complex problems, such as climate change and sustainable development, and provides a navigational tool for researchers, practitioners, and policymakers to bridge the gap between knowledge and action. |
| S009 | Chamberlain C, Clark Y, Hokke S, Hampton A, Atkinson C, Andrews S; HPNF investigator group. Healing the Past by Nurturing the Future: Aboriginal parents' views of what helps support recovery from complex trauma. Prim Health Care Res Dev. 2021 Sep 30;22:e47.  doi: 10.1017/S1463423621000463 | Moderate: The study uses a robust indigenist approach and grounded theory methods, overseen by an Aboriginal-led team to ensure cultural safety. However, there is a significant gender bias, as only 2 of the 17 participants were fathers. Additionally, the sample size is small (n = 17), and recruitment through partner health services may have introduced a selection bias toward parents already engaged with the system. | High: The findings are strongly supported by the data and presented through a cohesive theoretical supposition (the “basket” metaphor), which explains how connectedness and wellbeing intersect during the parenting transition. The results are consistently supported by direct participant quotes and align with both international literature and previous local studies. | Moderate: The data are qualitatively rich, employing creative “Tree of Life” visual tools and third-person scenarios to capture deep metaphors and symbolism regarding trauma and recovery. However, the quantity is limited; with only 17 participants across three sites (some sites having as few as 3 participants), the data may not fully capture the diversity of experiences within and between all Aboriginal and Torres Strait Islander communities. | High: The study is entirely applicable to the specific context of identifying trauma-integrated perinatal support needs for Aboriginal and Torres Strait Islander parents. It directly addresses the “urgent need” for better care strategies and provides actionable implications for practice, policy, and research specifically tailored to the Australian primary health care context. |
| S010 | Hantel A, Marron JM, Abel GA. Establishing and Defining an Approach to Climate Conscious Clinical Medical Ethics. Am J Bioeth. 2025 Jul;25(7):8-21.  doi: 10.1080/15265161.2024.2337418 | Moderate: The paper is a theoretical and conceptual synthesis, rather than an empirical study or a formal systematic review. While it rigorously applies philosophical frameworks, it does not employ a reproducible search protocol like a PRISMA-guided review. The authors also acknowledge that central debates, such as individual moral responsibility for climate change, remain without philosophical consensus. | High: The findings are strongly supported by a logical progression from identifying the “anthropocentric gap” in clinical medical ethics (CME) to proposing a structured practical solution. The transition from abstract moral theories (virtue, feminist, and justice) to the specific updates in the Climate Conscious Four Topics model is highly consistent and well-reasoned. | High: The paper draws on a rich and transdisciplinary evidence base, citing 83 sources across climate science (IPCC), public health (The Lancet), and diverse branches of moral philosophy. This high quantity and quality of “intellectual data” provides a robust foundation for the proposed ethical framework and its application to clinical practice. | High: The work is entirely applicable to the question of how medical ethics should address environmental crises. It specifically targets the “separation of clinical medical ethics from the environment” and provides a practical, practice-oriented tool (Table 1) intended for direct use by healthcare providers in clinical situations. |
| S011 | Ciesielski TH, Aldrich MC, Marsit CJ, Hiatt RA, Williams SM. Transdisciplinary approaches enhance the production of translational knowledge. Transl Res. 2017 Apr;182:123-134. doi: 10.1016/j.trsl.2016.11.002. Epub 2016 Nov 10. PMID: 27893987; PMCID: PMC5362296.  doi: 10.1016/j.trsl.2016.11.002 | Moderate: The paper is a perspective-driven review that provides “tangible examples,” rather than a strict systematic review or primary empirical study. The authors explicitly state they do not propose a “rigid protocol” for transdisciplinary research, as one does not exist. While the selection of examples is illustrative, the lack of a reproducible search protocol (such as PRISMA) is a methodological characteristic of this type of perspective piece. | High: The findings are strongly supported by a consistent internal logic that links cross-disciplinary communication to better hypothesis generation, evidence assessment, and intervention coordination. The paper demonstrates high coherence by showing how diverse tools, ranging from machine learning (MDR) to evidence synthesis frameworks (DiCE and LEAD), all lead to the same goal of advancing translational knowledge. | High: The authors draw on a rich and diverse evidence base, citing 98 references that span computational biology, social epidemiology, genetics, and clinical medicine. The quality of data are high, as it includes established international gold standards like the Cochrane Collaboration and IARC monographs, providing a robust foundation for the paper's conclusions. | High: Every example and strategy provided is directly applicable to the review question of how transdisciplinary approaches can facilitate translational research to improve human health. The authors specifically tailored their findings to the field of epidemiology, providing practical, “achievable” steps for researchers to break down disciplinary silos and address multifactorial health problems. |
| S012 | Carey G, Malbon E, Carey N, Joyce A, Crammond B, Carey A. Systems science and systems thinking for public health: a systematic review of the field. BMJ Open. 2015 Dec 30;5(12):e009002. doi: 10.1136/bmjopen-2015-009002. PMID: 26719314; PMCID: PMC4710830.  DOI: 10.1136/bmjopen-2015-009002 | Moderate: The authors followed a systematic search protocol and utilized a PRISMA flow diagram. However, they acknowledge that the sample is “representative but not comprehensive” and may have missed studies due to search term limitations. Additionally, the search was conducted by a single research assistant, and the assessment of data quality was performed as an “intrinsic” part of the analysis rather than using a standardized external risk-of-bias tool. | High: The findings are strongly supported by an inductive qualitative content analysis. The authors ensured consistency by having three authors categorize the literature to establish inter-rater reliability. The conclusions, such as the identified gap between systems rhetoric and actual operationalization, are logically and consistently derived from the extracted data across 117 articles. | High: The review is based on a substantial quantity of data, including 117 peer-reviewed articles and reports selected from an initial screen of 341 records. The search covered a 15-year period across major databases (e.g., PubMed, Web of Science) and included key public health domains such as obesity, tobacco, and social determinants of health, ensuring a rich evidence base. | High: The study is entirely applicable to the specific context of the review question regarding the state of systems science in public health. It successfully categorizes different levels of systems research and identifies critical shortcomings (e.g., lack of model validation) and fruitful areas (e.g., soft systems modeling) that are directly relevant to practitioners and policymakers. |
| S013 | Kabarite A, Ferreira GMM, Pitangueira JC, Arimatéa RS, da Costa Rebello de Mendonça R, Marcello RS, Schulz TG, Riesgo RDS, Castro K. A Longitudinal Transdisciplinary Approach for Autism Spectrum Disorder. Children (Basel). 2025 Sep 22;12(9):1272. doi: 10.3390/children12091272. PMID: 41007137; PMCID: PMC12469184.  DOI: 10.3390/children12091272 | Moderate: The study uses a longitudinal design with standardized, evidence-based instruments (CGI, GAF, ABC). However, the authors explicitly acknowledge several flaws: the absence of a control group limits causal inferences, the small sample size (n = 53) and high attrition (from an initial 67) may affect results, and the lack of multivariate adjustments for confounding variables like comorbidities further limits the findings. | High: The findings are strongly supported by the data, showing consistent clinical and functional improvements over 12 months. The paper demonstrates high coherence by comparing caregiver and therapist perspectives, providing a logical explanation for their divergences based on “experiential validation” and clinical stability thresholds. The results are consistently mapped back to the Priorit Methodology framework. | Moderate: The data are qualitatively rich, capturing assessments at three fixed time points across diverse domains such as behavior, sleep, and functional play. However, the quantity is limited by the small sample size and the specific context of a single private clinic, which may not represent the broader population. Due to these data constraints, the authors characterize their results as “preliminary evidence”. | High: The study is directly applicable to the specific context of evaluating transdisciplinary, family-centered care for Autism Spectrum Disorder (ASD). It addresses the urgent need for coordinated, individualized interventions, and offers practical translational value by bridging clinical practice and research in a real-world setting. The findings regarding family involvement are particularly relevant for long-term care models. |
| S014 | Gordon RM, Corcoran JR, Bartley-Daniele P, Sklenar D, Sutton PR, Cartwright F. A transdisciplinary team approach to pain management in inpatient health care settings. Pain Manag Nurs. 2014 Mar;15(1):426-35. doi: 10.1016/j.pmn.2013.01.004. Epub 2013 Mar 22. Erratum in: Pain Manag Nurs. 2014 Dec;15(4):920. PMID: 23522877.  DOI: 10.1016/j.pmn.2013.01.004 | Moderate: The paper is a conceptual and descriptive overview rather than a primary empirical study or a systematic review. While it provides a clear theoretical framework and historical context, it relies on illustrative case vignettes rather than a reproducible research protocol or experimental data. The authors also acknowledge limitations such as the need for additional time for team coordination and potential conflicts regarding shared responsibilities. | High: The findings are strongly supported by a logical and consistent framework that explains the transition from multidisciplinary to transdisciplinary care. The central arguments, that a unified treatment plan improves patient-centered goals, are consistently demonstrated across four distinct clinical vignettes ranging from palliative care to brain injury rehabilitation. | Moderate: The data are qualitatively rich, providing detailed narratives that include cultural nuances, family dynamics, and specific clinical interventions. However, the “data” is limited to these selective case examples from a single medical center. While well-referenced with 98 citations, the paper lacks large-scale quantitative data or statistical analysis to measure the broad efficacy of the model. | High: The study is entirely applicable to the specific context of improving pain management in inpatient settings; it directly addresses complex, “multi-determined” pain through a transdisciplinary lens that incorporates cultural diversity and health disparities. The findings are highly relevant for healthcare professionals, offering a practical model that aligns with the Institute of Medicine (IOM) recommendations. |
| S015 | Burris S, Ashe M, Levin D, Penn M, Larkin M. A Transdisciplinary Approach to Public Health Law: The Emerging Practice of Legal Epidemiology. Annu Rev Public Health. 2016;37:135-48. doi: 10.1146/annurev-publhealth-032315-021841. Epub 2015 Nov 30. PMID: 26667606; PMCID: PMC5703193.  DOI: 10.1146/annurev-publhealth-032315-021841 | Moderate: The paper is a conceptual review and perspective piece that describes an emerging model by uniting two existing traditions. While it provides a rigorous synthesis of a century of public health law, it is not a systematic review and does not employ a reproducible search protocol like PRISMA. However, the authors are transparent about their potential conflicts of interest regarding policy surveillance software. | High: The findings are strongly supported by a logical and structured framework (Figure 1) that integrates “public health law practice” with “legal epidemiology.” The paper consistently maps how diverse functions, such as counseling, litigation, and scientific coding, work together to achieve “better health faster,” ensuring the model is theoretically and practically sound. | High: The paper draws on a vast and rich evidence base comprising over 85 cited sources, including historical treatises from 1892, landmark IOM reports, and modern scientific evaluations in areas like tobacco control and traffic safety. This multidisciplinary data provides a robust foundation for defining the new field of legal epidemiology. | High: The work is entirely applicable to the review question regarding the integration of law into public health science and practice. It directly addresses the “unavoidable gaps in policy,” and provides concrete implications for institutional change, research funding, and professional training for lawyers and public health practitioners alike. |
| S016 | Mishra, K. K., Leung, I. C., Chao, M. T., Thompson-Lastad, A., Pollak, C., Dhruva, A., ... & Atreya, C. E. (2024). Mindfulness-based group medical visits: strategies to improve equitable access and inclusion for diverse patients in cancer treatment. Global Advances in Integrative Medicine and Health, 13, 27536130241263486.  doi.org/10.1177/27536130241263486 | Moderate: The study is a Quality Improvement (QI) pilot, rather than a randomized controlled trial, meaning it lacked a control group for formal effectiveness assessment. The “high-touch” recruitment through known providers may introduce selection bias, and the program was limited to English speakers, which contributed to the underrepresentation of Latino patients compared to the local population. However, the study followed the SQUIRE guidelines and used validated symptom assessment scales. | High: The findings are strongly supported by the data and show internal consistency. High attendance rates (90% attending ≥3 visits) and high satisfaction scores (87% rating the series as “excellent”) directly support the authors’ conclusions regarding feasibility and acceptability. Qualitative themes like connectedness and empowerment logically align with the quantitative results and the theoretical framework of group medical visits. | Moderate: The study provides rich qualitative and quantitative data from 78 participants across 10 cohorts, including detailed demographics and symptom burden profiles. However, the 3-month follow-up survey return rate was low (31%), which limits the authors’ ability to draw conclusions about the long-term impact or sustained practice of mindfulness techniques. | High: The research is highly applicable to the specific goal of improving health equity in integrative oncology. Every aspect of the study, from reserving 50% of slots for BIPOC patients to using trauma-informed, culturally adapted content, was specifically designed to address documented barriers to access for diverse populations in active cancer treatment. |
| S017 | Allen-Scott, L. K., Buntain, B., Hatfield, J. M., Meisser, A., & Thomas, C. J. (2015). Academic Institutions and One Health: Building Capacity for Transdisciplinary Research Approaches to Address Complex Health Issues at the Animal-Human-Ecosystem Interface. Academic medicine : journal of the Association of American Medical Colleges, 90(7), 866–871.  doi.org/10.1097/ACM.0000000000000639 | Moderate: This paper is a Perspective/Call to Action, rather than a primary empirical study or a systematic review. While it provides a logical and structured argument, it does not employ a reproducible search protocol or formal risk-of-bias assessment. It relies on illustrative case studies (e.g., ILRI and Swiss TPH) to support its value propositions rather than a comprehensive analysis of all available literature. | High: The findings are strongly supported by a consistent internal logic. The paper clearly defines terms, proposes a specific conceptual framework for transdisciplinary research (Lang et al.), and derives its recommendations directly from identified institutional barriers. The examples provided for cost-effectiveness and impact sustainability are highly consistent with the overarching theme of the added value of “One Health” approaches. | Moderate: The paper draws on a rich and diverse evidence base, citing 48 references that include theoretical frameworks, public health history, and specific disease control studies. However, as a perspective piece, the “data” consists of selective evidence chosen to support a specific argument. While the qualitative richness is high, it does not represent an exhaustive or quantitative dataset on institutional capacity. | High: This work is entirely applicable to the specific context of building institutional capacity for transdisciplinary “One Health” research. All recommendations, from training “transmitters” to restructuring funding and evaluation, are directly tailored to the animal-human ecosystem interface and the role of academic institutions in addressing complex global health challenges. |
| S018 | Collins, S. E., Clifasefi, S. L., Stanton, J., The Leap Advisory Board, Straits, K. J. E., Gil-Kashiwabara, E., Rodriguez Espinosa, P., Nicasio, A. V., Andrasik, M. P., Hawes, S. M., Miller, K. A., Nelson, L. A., Orfaly, V. E., Duran, B. M., & Wallerstein, N. (2018). Community-based participatory research (CBPR): Towards equitable involvement of community in psychology research. The American psychologist, 73(7), 884–898.  [doi.org/10.1037/amp0000167](https://doi.org/10.1037/amp0000167) | High: The paper is a peer-reviewed manuscript published by the American Psychological Association in American Psychologist. It draws its findings from a broad base of established systematic reviews, meta-analyses, and empirical studies to support claims regarding internal and external validity. | High: The findings are well-supported by the underlying data and are logically consistent with established psychological theories, such as Lewin’s action research and Bronfenbrenner’s ecological perspective. The benefits of CBPR, such as improved psychometric properties and community trust, are shown to be consistent across various cited studies. | High: The paper provides a rich and extensive dataset by integrating a comprehensive literature review with a detailed case study of a federally funded, multi-phase CBPR program. The case study includes specific quantitative outcomes and qualitative insights from community members. | High: The source is explicitly designed to address the specific context of psychology research, ethics, and practice. It directly maps CBPR principles to APA ethical standards and provides concrete, applicable steps for psychologists to implement this framework in their own work. |
| S019 | Fontana, L., Fasano, A., Chong, Y. S., Vineis, P., & Willett, W. C. (2021). Transdisciplinary research and clinical priorities for better health. PLoS Medicine, 18(7), e1003699.  [doi.org/10.1371/journal.pmed.1003699](https://doi.org/10.1371/journal.pmed.1003699) | Low: The paper does not offer any primary empirical study data or conduct a formal systematic review. While it uses a structured approach to propose a “prevention-centered” system, it relies on expert synthesis and narrative argument rather than a reproducible search protocol or experimental design. | High: The findings are strongly supported by consistent internal logic and a structured theoretical framework (Fig. 1). The paper successfully harmonizes diverse concepts, from cellular nutrient-sensing and epigenetics to industrial farming and planetary health, into a unified, coherent argument for system-wide reform. | High: The paper draws on a rich and diverse evidence base of 48 high-quality references, including established international reports (WHO, EAT-Lancet) and peer-reviewed studies in Nature, Science, and The Lancet. It integrates clinical trial data, epidemiological findings, and environmental science to support its multi-faceted claims. | High: The study is directly applicable to the urgent global challenges of chronic disease, environmental degradation, and unsustainable healthcare costs. It provides specific, actionable strategies for “transdisciplinary research and clinical priorities” that are highly relevant for policymakers, educators, and health professionals. |
| S020 | Keng, S. L., Smoski, M. J., & Robins, C. J. (2011). Effects of mindfulness on psychological health: a review of empirical studies. Clinical psychology review, 31(6), 1041–1056.  [doi.org/10.1016/j.cpr.2011.04.006](https://doi.org/10.1016/j.cpr.2011.04.006) | Moderate: The paper is a comprehensive narrative review, rather than a systematic review or meta-analysis. While it provides detailed tables summarizing randomized controlled trials (RCTs), it does not follow a reproducible search protocol like PRISMA. The authors also identify several field-wide limitations, such as a lack of consensus on defining mindfulness and the inherent paradoxes in current self-report measures. | High: The findings demonstrate a clear convergence of evidence across three distinct research methodologies: correlational, clinical intervention, and laboratory-based studies. All three lines of inquiry consistently support the conclusion that mindfulness is positively associated with psychological health. The paper’s structure logically moves from conceptual theory to empirical findings, and then to the specific psychological mechanisms that explain those findings. | High: The review draws upon an exceptionally rich and diverse dataset including 148 cited sources. It encompasses cross-sectional surveys, neuroimaging studies (EEG/fMRI), behavioral laboratory experiments, and dozens of RCTs for major interventions like MBSR, MBCT, DBT, and ACT. This multi-methodological evidence base provides a robust foundation for the authors’ conclusions. | High: Every study reviewed is directly applicable to the specific question of how mindfulness affects psychological health. The authors narrowed their focus specifically to adult populations and prioritized published, peer-reviewed RCTs for the clinical intervention section. The findings are translated into concrete clinical implications, such as reduced rumination and improved emotion regulation, making the data highly relevant for psychological practice. |
| S021 | Knudsen, R. K., Skovbjerg, S., Pedersen, E. L., Nielsen, C. L., Storkholm, M. H., & Timmermann, C. (2024). Factors affecting implementation of mindfulness in hospital settings: A qualitative meta-synthesis of healthcare professionals' experiences. International journal of nursing studies advances, 6, 100192.  <https://doi.org/10.1016/j.ijnsa.2024.100192> | Moderate: The review followed a rigorous, pre-registered protocol (PROSPERO) and used the ENTREQ reporting guidelines. However, the authors identify several limitations: most importantly, 16 of the 18 studies were from Western countries thus, limiting global transferability. Furthermore, only three studies had implementation as their primary focus, with the rest focusing on general experiences. There is also a risk of selection bias, as all included studies used self-selected samples of motivated participants. | High: The findings are strongly supported by a systematic thematic synthesis involving multiple researchers to ensure inter-rater reliability. The results are highly coherent, moving logically from descriptive codes to three analytical themes (Buying In, Allocating time and space, Keeping it going). These themes are further validated by being mapped against the established i-PARIHS implementation framework. | High: The richness and quantity of data are substantial for a qualitative meta-synthesis. The authors screened 11,491 records to identify 18 eligible studies involving 410 healthcare professionals across various medical disciplines (e.g., cardiology, oncology, surgery). This broad evidence base allowed for the identification of consistent facilitating and inhibiting factors across different hospital contexts. | High: The study is directly applicable to the specific review question regarding factors that affect the implementation of mindfulness in hospital settings. The findings address a documented gap in clinical practice, the lack of any qualitative evidence on successful implementation, and culminate in eight concrete recommendations specifically for hospital stakeholders and management. |
| S022 | Lawless, M. T., Tieu, M., Archibald, M. M., Pinero De Plaza, M. A., & Kitson, A. L. (2025). From Promise to Practice: How Health Researchers Understand and Promote Transdisciplinary Collaboration. Qualitative health research, 35(1), 3–16.  <https://doi.org/10.1177/10497323241235882> | Moderate: The study utilizes a rigorous qualitative descriptive design with independent coding by two researchers and member-checking to ensure accuracy. However, the authors acknowledge several limitations: the sample is small (n=13) and drawn from a single Australian-based research center, which may limit the transferability of the findings to teams with different compositions or training levels. | High: The findings are strongly supported by the underlying data, with four clear themes derived through systematic thematic analysis. The paper demonstrates high internal consistency, using direct participant quotations to illustrate concepts such as the “merging” of disciplines and the “negotiation of knowledge.” Results are also noted to be consistent with previous international studies. | Moderate: The data are qualitatively rich, providing deep insights into the perceptions, barriers, and strategies of researchers across 13 different disciplines (e.g., geriatric medicine, demography, nursing). While the depth of the interviews is high, the quantity of data are limited by the small absolute number of participants and the single-site focus, which may not capture the views of those who interacted less frequently with the center. | High: The study is entirely applicable to the review question regarding how health researchers understand and promote transdisciplinary collaboration. It specifically addresses a documented “practical knowledge gap” and provides actionable strategies for leaders to foster a collaborative mindset and break down disciplinary silos in health systems. |
| S023 | Tobias Mortlock, J. M., Alapati, H. H., & Edginton, T. (2025). How to create a mindful community of practice: Exploring the social functions of group-based mindfulness practices facilitated via Zoom during Covid-19. Frontiers in Psychology, 16, 1356057.  <https://doi.org/10.3389/fpsyg.2025.1356057> | Moderate: The study follows rigorous qualitative standards, including independent coding by two researchers to ensure intercoder reliability and a strong emphasis on reflexivity to address potential biases. However, the authors explicitly acknowledge several design flaws: the sample was composed entirely of volunteers (introducing selection bias), the exploratory design prevents generalizable predictions, and there was a risk of researcher bias since one of the facilitators of the sessions was also involved in the data analysis. | High: The findings are strongly supported by the underlying interview data, with the four primary thematic codes, collective alignment, common humanity, improved mind, body awareness, and unique mutual trust, mapped directly to rich participant narratives. These themes are organized into a logical relationship (Figure 1) and consistently discussed through the established lens of interdependence theory, ensuring that the conclusions are theoretically and empirically harmonized. | Moderate: The data are qualitatively rich, and the researchers achieved data saturation, reporting that no significant new insights were collected in the final interviews. However, the quantity of data are limited; the sample size is small (n=16) and reflects a significant gender imbalance (13 females vs. 3 males). Furthermore, the data were collected from a single metropolitan university site during the unique context of the COVID-19 pandemic, which may restrict its broader applicability. | High: This research is entirely applicable to the review question of how group-based mindfulness practices function socially. It addresses an understudied domain in mindfulness literature, moving beyond individual benefits to explore “interbeing” and collective transformation. The resulting exploratory propositions (Figure 2) provide practical, testable strategies for building mindful communities in modern workplaces and educational settings. |
| S024 | Marikar Bawa FL, Mercer SW, Sutton JW, Bond CM. Mindfulness for people with chronic pain: Factors affecting engagement and suggestions for programme optimisation. Health Expect. 2023 Jun;26(3):1287-1307. doi: 10.1111/hex.13745. Epub 2023 Mar 12. PMID: 36908018; PMCID: PMC10154871.  doi: 10.1111/hex.13745. Epub 2023 Mar 12. | Moderate: The study employed a robust multiphase approach, including Interpretative Phenomenological Analysis (IPA), a nominal group technique, and focus groups. However, the authors identified several flaws: a significant delay (approximately 3-4 years) occurred between the initial program and the modification meetings, which may have impacted participant recall. Additionally, there was a low turnout for the focus group (only 17% of invited service users), and the lead researcher’s status as a mindfulness practitioner introduced potential bias. | High: The findings are strongly supported by the data, with themes (physical, psychological, and social factors) consistently derived from verbatim interview transcripts and then mapped to specific program modifications. The paper demonstrates high internal consistency by triangulating the perspectives of completers, non-completers, and healthcare professionals to reach a consensus on 20 distinct modifications. | High: The data are qualitatively rich and extensive, drawing from interviews with 34 participants of varying engagement levels (completers, non-completers, and non-attenders). The study also integrated expert input from seven healthcare professionals across diverse fields (psychology, psychiatry, and general practice) and utilized 155 references to ground the findings in existing biopsychosocial and motivational theories. | High: The study is directly applicable to the specific context of optimizing mindfulness program for chronic pain. It addresses a documented gap in research by identifying barriers to engagement as a primary objective and providing actionable recommendations, such as using “taster sessions” and tailoring movement exercises to physical ability, to improve clinical practice and retention. |
| S025 | Zeidan F, Vago DR. Mindfulness meditation-based pain relief: a mechanistic account. Ann N Y Acad Sci. 2016 Jun;1373(1):114-27. doi: 10.1111/nyas.13153. PMID: 27398643; PMCID: PMC4941786.  doi: 10.1111/nyas.13153 | Moderate: The paper is a narrative review that acknowledges several common flaws in the field, such as the use of cross-sectional designs for long-term meditators which can be affected by demographics and demand characteristics. While many studies are susceptible to placebo-type effects, the review balances this by highlighting recent, high-quality randomized, placebo-controlled, and double-blind longitudinal designs. | High: The findings demonstrate strong internal consistency, showing a logical shift from top-down executive modulation in novice meditators to sensory-appraisal decoupling in experts. This coherence is maintained across different methodologies, including psychophysical ratings, neuroimaging (fMRI/ASL), and pharmacological interventions. | High: The review draws on an extensive evidence base, citing 165 sources. The data are both rich and diverse, incorporating evidence from various meditative traditions (e.g., Zen, Vipassana) and measuring multiple variables such as cerebral blood flow, respiratory rates, and subjective pain intensity/unpleasantness. | High: The paper is entirely applicable to the clinical and social context of the current opioid epidemic and the search for nonpharmacological pain therapies. It specifically targets the need for mechanistic validation to increase the clinical acceptance of mindfulness meditation for chronic pain patients. |
| S026 | Martin, A. K., Green, T. L., McCarthy, A. L., Sowa, P. M., & Laakso, E. L. (2022). Allied health transdisciplinary models of care in hospital settings: A scoping review . Journal of Interprofessional Care, 37(1), 118–130.  <https://doi.org/10.1080/13561820.2022.2038552> | Moderate: The review followed a rigorous, pre-registered protocol guided by Joanna Briggs Institute (JBI) methodology and PRISMA-ScR guidelines, utilizing two independent reviewers for screening and data extraction. However, the authors explicitly acknowledge that the underlying evidence base is of limited quality, noting that “high-quality evidence is scarce” and includes only one randomized controlled trial alongside experimental studies and a conference abstract. | High: The findings are internally consistent and strongly supported by the data synthesized from the nine included studies. The paper demonstrates high coherence by identifying consistent patterns, such as improvements in time-efficiency and quality of care, across diverse hospital settings (e.g., EDs, acute stroke units, and general wards) and mapping these results logically to the research sub-questions. | Moderate: While the review provides qualitatively rich mapping of model characteristics and implementation factors, the total quantity of data are limited to only nine unique sources. There is a significant dearth of evidence regarding the labor and capital cost outcomes (these were evaluated by only one study) and a lack of formal qualitative research specifically measuring stakeholder perceptions. | High: The studies are highly applicable to the specific context of the review question, which focuses on the implementation of transdisciplinary models among allied health professionals in hospitals. The findings directly address the practical roles of professions such as occupational therapy and physiotherapy and provide actionable recommendations for hospital stakeholders regarding implementation barriers and facilitators. |
| S027 | O’Shea, M., Reich, D. & Evans, S. Applying Participatory Research in the Development of Clinical Practice Recommendations for Incorporating Mindfulness into Mental Health Treatment with Youth at Risk for Psychosis. Mindfulness 15, 721–732 (2024).  <https://doi.org/10.1007/s12671-024-02313-w> | Moderate: The study employed a rigorous three-phase participatory research design, integrating evidence mapping, qualitative interviews, and a validation cycle. It followed established reporting guidelines such as the TIDieR checklist. However, the final validation cycle relied on a very small sample of five practitioners, and the authors acknowledge that these participants may not entirely represent the diverse views of all stakeholders in this field. | High: The findings are strongly supported by the integration of data across all phases, with the recommendations directly formulated from the evidence mapping and stakeholder qualitative analysis. There is high internal consistency, as the initial recommendations were fed back to practitioners in a validation cycle to ensure they accurately matched their clinical perspectives and expert views. | Moderate: The data are qualitatively rich, drawing from a mapping review of 72 studies, interviews with 14 stakeholders (8 practitioners and 6 youth), and expert validation. However, the authors explicitly state that research in this area is in its “relative infancy,” and that only a small number of primary studies currently exist specifically for the “at-risk” cohort, which limits the overall quantity of empirical evidence. | High: The paper is entirely applicable to the specific clinical goal of safely incorporating mindfulness into mental health treatment for youth at risk for psychosis. It addresses documented treatment gaps and safety barriers (e.g., trauma-informed care, managing paranoia) by providing eight specific recommendation areas with actionable clinical practice points. |
| S028 | Martins, P. (2018). Being Transdisciplinary in Human Sciences: The usefulness of Integrative medicine in contemporary society. Transdisciplinary Journal of Engineering and Science, 9(5), 37-43.  doi: 10.22545/2018/00100 | Low: This paper is defined as a “communication” or a narrative overview rather than an empirical study, systematic review, or meta-analysis. It lacks a formal, reproducible methodology, such as a search protocol for the historical and scientific theories cited, making it a theoretical synthesis based on the author’s selection of literature. | High: The findings are internally consistent and demonstrate a logical progression from the historical roots of both Eastern and Western medicine to constuct a unified “bio-psycho-spiritual” model. The author successfully harmonizes disparate concepts, such as Ayurvedic “doshas” and quantum nonlocality, to support the coherence of an integrative medicine framework. | Moderate: The paper provides rich qualitative data by synthesizing 44 diverse references, including sacred Hindu texts, Cartesian philosophy, and modern neurobiology. However, it does not present primary empirical data or quantitative results, relying instead on existing theoretical models and illustrative diagrams (e.g., the Kosha system and Egg’s diagram) to support its claims. | High: The work is highly applicable to contemporary global health challenges and specifically aligns with Goal #3 of the United Nations 2030 Agenda for Sustainable Development. It provides direct clinical relevance by comparing treatment approaches for common issues like depression, advocating for a shift from purely allopathic care to holistic, transdisciplinary interventions. |
| S029 | Schuman-Olivier Z, Trombka M, Lovas DA, Brewer JA, Vago DR, Gawande R, Dunne JP, Lazar SW, Loucks EB, Fulwiler C. Mindfulness and Behavior Change. Harv Rev Psychiatry. 2020 Nov/Dec;28(6):371-394. doi: 10.1097/HRP.0000000000000277. PMID: 33156156; PMCID: PMC7647439.  doi: 10.1097/HRP.0000000000000277 | Moderate: This paper is defined as an integrative and narrative review rather than a primary empirical study or a formal systematic review. While it provides a rigorous synthesis, it does not employ a reproducible search protocol (like PRISMA). The authors are highly transparent about field-wide flaws, noting substantial heterogeneity in meta-analyses, small sample sizes in individual studies, a lack of adverse event monitoring, and the underrepresentation of marginalized populations. | High: The findings are strongly supported by a robust theoretical framework (the “mindful self-regulation” model) that successfully integrates neurobiological mechanisms with clinical outcomes. The paper demonstrates high internal consistency by showing how various cognitive, emotional, and self-related processes synergistically lead to sustainable behavior change across diverse domains like addiction, eating disorders, and chronic disease. | High: The review draws on an extraordinary evidence base, citing 474 sources ranging from 19th-century translations to cutting-edge neuroimaging studies conducted in the 2020s. It synthesizes data from meta-analyses, well-designed RCTs, and longitudinal neuroscientific research, providing a rich and high-quality foundation for its conclusions. | High: The work is entirely applicable to the urgent global challenge of managing preventable chronic medical and psychiatric illnesses. It directly addresses the 40%-50% of early death risk in the U.S. by providing actionable insights into how mindfulness can facilitate the initiation and maintenance of healthy behaviors. The findings are specifically tailored to enhance clinical practice in psychiatry. |
| S030 | Strugnell, C., Orellana, L., Crooks, N. et al. Healthy together Victoria and childhood obesity study: effects of a large scale, community-based cluster randomised trial of a systems thinking approach for the prevention of childhood obesity among secondary school students 2014–2016. BMC Public Health 24, 355 (2024).  <https://doi.org/10.1186/s12889-024-17906-2> | Moderate: The study is a large-scale cluster randomised trial (CRT) embedded in a natural experiment, which applied an opt-out approach to achieve exceptionally high participation rates (85-91%). However, significant limitations exist: the 2014 data do not represent a true baseline because implementation began in 2013, and the intervention was terminated early (operating for only 4 of the planned 6 years). Additionally, behavioral risks like diet and exercise relied on self-report measures subject to respondent bias. | High: The findings are strongly supported by the underlying data through the use of linear mixed models and GEE analysis to adjust for school-level factors. While the results showed unexpected gender-specific effects (favoring boys in waist circumference and beverage consumption), the overall direction and magnitude of changes in BMIz were consistent with findings from previous whole-of-community interventions like Romp & Chomp and Shape Up Somerville. | High: The richness and quantity of data are substantial, involving 7,241 secondary school students across 23 communities, which significantly exceeded the study's original power calculations. The dataset is high-quality, combining objective anthropometric measurements (height, weight, and waist circumference) with comprehensive secondary data on physical activity, diet quality, and psychosocial health (quality of life and depressive symptoms). | High: The paper is directly applicable to the global public health priority of reversing the childhood obesity epidemic. As one of the first attempts to evaluate a large-scale, government-led systems thinking approach, the findings provide critical evidence for policymakers and clinicians regarding the feasibility and impact of whole-of-community interventions in a real-world setting. |
| S031 | Morgan, M.J., Stratford, E., Harpur, S. et al. A Systems Thinking Approach for Community Health and Wellbeing. Syst Pract Action Res 37, 161–183 (2024).  <https://doi.org/10.1007/s11213-023-09644-0> | Moderate: The study is a single-case study in a remote municipality, which limits the generalizability of its findings. Flaws in conduct include a small sample size (n=19) and a low evaluation survey return rate (n=10), primarily due to participant workload. Additionally, resource constraints prevented the team from testing the final “act strategically” phase of their framework. | High: The findings are strongly supported by the underlying data and show a high internal consistency. There is a clear logical alignment between the theoretical “mindful self-regulation” framework used and the qualitative feedback from participants, who consistently reported increased capacity and a deeper, shared understanding of local health issues. | Moderate: The data are qualitatively rich, providing “surprising” and deep insights into the local prevention system through participatory inquiry and a professional “rich picture.” However, the quantity of data are limited by the small participant pool and the fact that some routine community-level data were unavailable or too small to be reported for this remote site. | High: The study is entirely applicable to the challenge of addressing complex, place-based health issues like noncommunicable diseases (NCDs). It directly addresses a documented “research gap” by testing a systems approach with local government actors and provides actionable conclusions for both practitioners and macro-level policymakers. |
| S032 | Anindhita, M., Haniifah, M., Putri, A.M.N. et al. Community-based psychosocial support interventions to reduce stigma and improve mental health of people with infectious diseases: a scoping review. Infect Dis Poverty 13, 90 (2024).  <https://doi.org/10.1186/s40249-024-01257-6> | Moderate: The review followed rigorous international standards, including PRISMA-ScR and Arksey and O’Malley guidelines, with a systematic search across multiple databases. However, the authors admit that most included studies (20/30) were not randomized controlled trials, making them prone to sample selection bias. Additionally, many of the primary studies used unvalidated outcome tools and were limited by a significant geographical imbalance, with most research conducted in Africa. | High: The findings demonstrate strong internal consistency, identifying consistent reported mechanisms (e.g., supporters making patients feel “seen and listened to”) that reduce stigma across three different diseases. The results are logically synthesized and mapped onto a newly developed integrated framework for stigma reduction, ensuring the conclusions are theoretically and empirically harmonized. | Moderate: The review is based on a screening of 13,252 records, ultimately including 30 articles that utilize quantitative, qualitative, and mixed-methods. However, the authors explicitly state there is a “paucity of high-quality evidence” in this field. There is also a significant data gap regarding Tuberculosis, which accounted for only 7% (two studies) of the reviewed literature compared to 70% for HIV/AIDS. | High: The paper is entirely applicable to the specific review question of identifying and appraising community-based psychosocial interventions for stigmatized infectious diseases. It identifies 12 distinct types of interventions and directly links them to mental health and treatment outcomes, providing actionable evidence for programs like the TB-CAPS study in Indonesia. |
| S033 | Kanyagui, M. K., Sajithkumar, K. J., Velankar, Y., Mohan, R., Viswanathan, P. K., & Magnani, N. (2023). Livelihood challenges faced by women in rural India: exploration of solutions using participatory action research. Development in Practice, 34(4), 512–526.  <https://doi.org/10.1080/09614524.2023.2285251> | Moderate: The paper is defined as a “practice note” that documents experiential learning and the application of a three-phase Participatory Action Research (PAR) framework. While the tools used (journey maps, brainstorming, ranking) are standard for PAR, the study is limited to a single village (Nagla Chandi) and focuses on only two self-help groups. This specific geographic and caste-based context (Rajput) limits the broader generalizability of the findings. | High: The findings demonstrate strong internal consistency and a logical flow. There is a clear, evidence-based progression from the identification of daily burdens (e.g., the heavy time commitment to caring for buffaloes) to the development of matched solutions (e.g., value-added dairy production and vermicompost). The synthesis of challenges and solutions in the final model (Figure 12) is directly supported by the preceding investigative phases. | Moderate: The paper provides qualitatively rich data through multi-modal participatory tools, including daily activity journey maps and storytelling. However, the researchers acknowledge that this is an innovative approach for a context where “data are limited.” While the qualitative insights are deep for this specific case, the quantitative data on income and population (490 people) is small-scale and lacks the breadth of a larger empirical survey. | High: The work is entirely applicable to the urgent challenge of addressing gender-based economic inequality and the “low participation rate” of women in the Indian labor force. It provides actionable, community-derived solutions for policymakers and civil society organizations (CSOs) looking to empower rural women through skills development, capital access, and the valuation of unpaid domestic work. |
| S034 | Lee, J. Y., Clark, J. K., Schmiesing, R., Kaiser, M. L., Reece, J., & Park, S. (2024). Perspectives of community members on community-based participatory research: A systematic literature review. Journal of Urban Affairs, 47(8), 2916–2935.  <https://doi.org/10.1080/07352166.2024.2305132> | High: The review followed a highly rigorous and transparent systematic protocol modified from the PRISMA statement. The authors used two major databases (WoS and EBSCO), clearly defined the inclusion/exclusion criteria, and employed a dual-coder process with a consensus-building phase and validation to ensure high inter-rater reliability. | High: The findings are strongly supported by the underlying data and show high internal consistency. The authors successfully synthesized disparate terms and methodologies across health, interdisciplinary, and social science fields to demonstrate a consistent trend, specifically, that social sciences lag behind health-related fields in publication volume and the inclusion of community voices. | High: The richness and quantity of data are exceptional for a review of this scope. The researchers screened 701 unique records to identify a final dataset of 97 peer-reviewed articles. The analysis extracted multi-dimensional data, including funding sources, geographic locations, and the specific subject perspectives (researcher vs. community member) of each study. | High: The paper is directly applicable to the review question of understanding how community members perceive and experience CBPR. It identifies a critical “missing voice” in current scholarship and provides a specific “deep dive” into social science articles that center community perspectives, offering actionable insights for anchor land-grant institutions. |
| S035 | Thomas E, Benjamin-Thomas TE, Sithambaram A, Shankar J, Chen S-P. Participatory Action Research Among People With Serious Mental Illness: A Scoping Review. Qualitative Health Research. 2023;34(1-2):3-19.  doi:10.1177/10497323231208111 | Moderate: The review followed a rigorous, established five-step framework by Arksey and O’Malley and complied with PRISMA-ScR reporting guidelines. However, the authors explicitly note several limitations: the search was restricted to English-language publications, and the scoping review format meant they did not perform a critical analysis or formal quality appraisal of the 87 included studies. Additionally, space constraints prevented a detailed analysis of the nuances of power-sharing between academic and community partners. | High: The findings are strongly supported by the underlying data and show high consistency across the large volume of literature. The five identified strategies for empowerment (building capacity, balancing power, collaborative environments, peer support, and enhancing engagement) were consistently observed across various study designs. The authors successfully mapped these diverse findings onto an established empowerment framework, ensuring high theoretical and empirical harmony. | High: The review draws from an extensive and qualitatively rich evidence base, including 87 studies published between 1997 and 2021. The data quantity is substantial, having been distilled from an initial pool of 1,328 records and including grey literature (dissertations) to ensure a comprehensive overview. The findings cover a broad range of life areas, including self-care, employment, housing, and social participation. | High: The study is directly and entirely applicable to the review question of how PAR is conducted with PSMI. It addresses a specific gap, the lack of a previous “map” of the nature and extent of PAR in this population, and provides actionable strategies for future researchers to empower co-researchers and facilitate social transformation. |
| S036 | Ramanadhan S, Alemán R, Bradley CD, Cruz JL, Safaeinili N, Simonds V, Aveling EL. Using Participatory Implementation Science to Advance Health Equity. Annu Rev Public Health. 2024 May;45(1):47-67. doi: 10.1146/annurev-publhealth-060722-024251. Epub 2024 Apr 3. PMID: 38109515; PMCID: PMC11251496.  DOI: 10.1146/annurev-publhealth-060722-024251 | Moderate: The paper is a narrative review and framework development piece, rather than a primary empirical study or a systematic review. While it adheres to the high academic standards of the Annual Review of Public Health, it lacks a formal, reproducible search protocol. However, it mitigates potential bias through critical reflexivity, with the authors explicitly examining their own diverse social locations and positionality. | High: The findings demonstrate exceptional internal consistency, logically moving from a rationale for participatory research to a structured framework for Implementation Science (IS). The paper successfully harmonizes diverse concepts, such as CBPR, Indigenous knowledge traditions, and implementation strategies, into a unified model for advancing health equity that is supported by consistent evidence across various case studies. | High: The review draws upon an extensive and diverse evidence base, citing 140 unique sources. The data are qualitatively and theoretically rich, integrating examples from various contexts including COVID-19 vaccination rollout, tobacco control, and rural telemedicine in India. It also includes comprehensive tables of exemplar outcomes and figures detailing engagement levels. | High: The work is entirely applicable to the challenge of moving beyond “top-down” implementation models toward transformative, equity-centered solutions. It provides actionable guidance for researchers and community partners on how to co-produce knowledge and reshape harmful systems, directly addressing the documented gap in equity-focused IS grants. |
| S037 | Bogerd, M.J., Slottje, P., Bont, J. et al. Development of a person-centred care approach for persons with chronic multimorbidity in general practice by means of participatory action research. BMC Prim. Care 25, 114 (2024).  <https://doi.org/10.1186/s12875-024-02364-x> | Moderate: The study used a robust Participatory Action Research (PAR) design with four iterative cycles to ensure stakeholder ownership. However, several limitations were noted: the participants were “intrinsically motivated” volunteers who may not represent the broader GP population, and the COVID-19 pandemic significantly disrupted the project's timeline and participant attention. Additionally, the new approach had to be developed while conflicting single-disease management protocols were still active. | High: The findings are strongly supported by the underlying data and show high internal consistency. The three-step framework (selection, consultation, and follow-up) emerged logically from the iterative cycles. Qualitative insights from focus groups were consistently triangulated with Electronic Medical Record (EMR) data, such as the analysis of journal reports which corroborated the reported difficulty in setting person-centred goals. | High: The data are qualitatively and quantitatively rich, collected across 13 general practices over 2.5 years. The evidence base includes 15 focus groups, 21 patient interviews, and the analysis of 229 EMR journal reports. The research team employed rigorous coding practices, including independent dual-coding of initial datasets to enhance the coding scheme and ensure reliability. | High: The study is entirely applicable to the global challenge of managing chronic multimorbidity, where traditional single-disease models are increasingly viewed as inappropriate. It provides a concrete, piloted framework (Figure 1) and identifies specific, actionable facilitators and barriers, such as the need for better registration tools and appropriate remuneration, directly relevant to primary care implementation. |
| S038 | Carrick, J., Bell, D., Fitzsimmons, C., Gray, T., & Stewart, G. (2022). Principles and practical criteria for effective participatory environmental planning and decision-making. Journal of Environmental Planning and Management, 66(14), 2854–2877.  <https://doi.org/10.1080/09640568.2022.2086857> | Moderate: The paper is a theoretical synthesis and framework development rather than an empirical study or a formal systematic review. The authors explicitly state it is “not a comprehensive review” of all previous participatory ideals and acknowledge that the proposed criteria still “require empirical testing” to verify their practical usefulness. | High: The paper demonstrates exceptional internal consistency. It follows a logical and transparent progression from identifying core values in established theories to synthesizing them into four overarching principles (inclusivity, process-orientation, empowerment, and reflection), which were then systematically translated into practical criteria. | High: The review draws from an extensive and qualitatively rich evidence base, citing 106 unique sources, spanning political science, environmental planning, and science and technology studies. This high volume of theoretical literature allows the authors to move beyond abstract ideals to create specific actionable points. | High: The work is entirely applicable to the persistent real-world “paradox of participation” where theoretical ideals often fail in environmental planning practice. it directly addresses documented failures in contexts such as offshore wind farm siting and marine conservation zones, providing a flexible bridge for practitioners and decision-makers. |
| S039 | Kwamie A, Causevic S, Tomson G, Sie A, Sauerborn R, Rasanathan K, et al. Prepared for the polycrisis? The need for complexity science and systems thinking to address global and national evidence gaps. BMJ Global Health. 2024;9:e014887.  <https://doi.org/10.1136/bmjgh-2023-014887> | Moderate: The paper is defined as an analysis and perspective piece. While informed by a review of evidence and country policy documents (e.g., Burkina Faso), it does not follow a formal, reproducible systematic protocol (such as PRISMA). However, it employs a rigorous complexity science lens to critique existing research methodologies. | High: The findings are highly coherent, presenting a clear argument for why current “correlational and descriptive” data are insufficient for managing the global “polycrises.” There is a logical link between the failure of current governance systems and the need for specific systems thinking tools (e.g., causal loop diagrams, process maps) to account for feedback and emergence. | Moderate: The paper draws on 50 diverse sources, including UN SDG reports and national policy plans. However, the authors explicitly argue that the existing global evidence base is “methodologically, topically, and geographically limited,” which inherently constrains the quantity of high-quality data available to support their synthesis. | High: This work is extremely relevant to the current global context, specifically addressing the 2024 Sustainable Development Goals (SDGs) and overlapping crises, including climate change and conflict. It provides critical guidance for transitioning to systemic risk frameworks that can inform complex policy actions. |
| S040 | Rusoja E, Haynie D, Sievers J, Mustafee N, Nelson F, Reynolds M, Sarriot E, Swanson RC, Williams B. Thinking about complexity in health: A systematic review of the key systems thinking and complexity ideas in health. J Eval Clin Pract. 2018 Jun;24(3):600-606.  doi: 10.1111/jep.12856. | High: The authors utilized a “rigorous and transparent methodology.” This included a predetermined search strategy across five major databases, a two-phase screening process, and the use of two independent reviewers with a third to resolve any disagreements. While the authors acknowledge that “fuzzy boundaries” between fields may have led to some missed literature, their overall conduct follows established systematic review standards. | High: The findings are strongly supported by the underlying data and show high internal consistency. The conclusion that there is an increasing interest in Systems Thinking (ST) and Complexity Theory (CT) but a lack of consensus on terms is directly derived from the frequency analysis of 516 articles. The synthesis of shared concepts across disciplines (e.g., emergence, CAS) is logically supported by the term-mapping data. | High: The quantity and richness of data are substantial. The review analyzed 516 full-text articles distilled from an initial pool of 3,982 records. The researchers extracted data on 57 relevant terms and 21 applicable methods, providing a comprehensive overview across medicine, public health, and management disciplines. | High: The study is entirely applicable to the challenge of understanding and applying ST/CT in modern healthcare. It directly links these theoretical frameworks to the practical needs of the Sustainable Development Goal (SDG) era, identifying how such concepts can help stakeholders to move beyond the narrow, reductionist targets of the past. |
| S041 | Andonian BJ, Hippensteel JA, Abuabara K, Boyle EM, Colbert JF, Devinney MJ, Faye AS, Kochar B, Lee J, Litke R, Nair D, Sattui SE, Sheshadri A, Sherman AN, Singh N, Zhang Y, LaHue SC. Inflammation and aging-related disease: A transdisciplinary inflammaging framework. Geroscience. 2025 Feb;47(1):515-542.  doi: 10.1007/s11357-024-01364-0 | Moderate: The paper is explicitly defined as an “expert commentary and narrative review,” rather than a primary empirical study or a formal systematic review. Its clinical examples are intended to be “illustrative” rather than comprehensive, and it lacks a reproducible search protocol. However, it utilizes a rigorous, structured overview for each disease manifestation based on existing evidence. | High: The findings demonstrate strong internal consistency, logically connecting the biological hallmarks of aging (inflammaging and immunosenescence) to specific pathologies across nine distinct medical subspecialties. The proposed transdisciplinary framework directly addresses the "silos of specialty care" identified as a major barrier in the introduction. | High: The review draws from an exceptional and diverse evidence base, citing 248 unique sources. The data are qualitatively rich, integrating detailed mechanistic insights and clinical observations from endocrinology, rheumatology, neurology, and more. The authors also provide a transparent account of current “knowledge gaps” to balance the synthesis. | High: The paper is entirely applicable to the urgent challenge of managing multimorbidity in an aging population. It provides a concrete roadmap for integrating geroscience into clinical practice and identifies actionable opportunities for both pharmacologic (e.g., senolytics) and non-pharmacologic (e.g., diet and exercise) interventions. |
| S042 | Wan TTH, Wan HS. Predictive Analytics with a Transdisciplinary Framework in Promoting Patient-Centric Care of Polychronic Conditions: Trends, Challenges, and Solutions. AI. 2023; 4(3):482-490.  <https://doi.org/10.3390/ai4030026> | Moderate: The paper is defined as a “commentary” based on an innovative approach to predictive analytics rather than a primary empirical study or a formal systematic review. While it proposes a “customized algorithm” and theoretical specifications for causal inquiries, it does not present a reproducible search protocol or a new experimental test of the framework within this specific text. | High: The findings are internally consistent and demonstrate a strong logical flow. The authors identify specific challenges in chronic care, such as the lack of theoretical guidance in variable selection, and provide matching, coherent solutions, such as shifting from retrospective to prospective study designs. | Moderate: The paper is theoretically rich and draws from 41 unique references covering diabetes, heart failure, and chronic kidney disease. However, the paper itself does not provide new primary data, instead relying on the synthesis of existing “big-data-to-knowledge” trends and previous transdisciplinary research to support its conclusions. | High: The work is entirely applicable to the challenge of promoting patient-centric care for polychronic conditions. It directly addresses the urgent need for clinical decision support systems and AI-based diagnostics that can handle the transition from single diseases to complex, multiple chronic conditions. |
| S043 | Archibald, M.M., Lawless, M.T., de Plaza, M.A.P. et al. How transdisciplinary research teams learn to do knowledge translation (KT), and how KT in turn impacts transdisciplinary research: a realist evaluation and longitudinal case study. Health Res Policy Sys 21, 20 (2023).  <https://doi.org/10.1186/s12961-023-00967-x> | High: The study employed a rigorous, longitudinal realist evaluation design over three years. It utilized multiple data sources for triangulation, including semi-structured interviews, participant observation (field notes), document reviews, visual elicitation, and productivity mapping. The iterative process of theory development, verification, and refinement, including the purposive inclusion of “deviant cases” to challenge working hypotheses, ensured high methodological transparency and rigor. | High: The findings are strongly supported by a clear, logical progression from initial hypotheses to three refined programme theories (Navigate, Negotiate, and Mobilize the network). These theories are consistently supported by integrated narrative and visual data, and the authors explicitly map how the specific contexts and mechanisms lead to observed outcomes. The results demonstrate high internal consistency across the 5-year lifespan of the research center. | High: The data are exceptionally rich, drawing from three distinct rounds of theory testing with a diverse sample of chief investigators, fellows, and students. The evidence base includes qualitative depth from visual elicitation (drawings) and interviews, supplemented by quantitative productivity data such as publication and grant outputs. This multi-modal approach provides a comprehensive view of team dynamics and research impact. | High: The study is entirely applicable to the “wicked problems” facing modern health systems. It addresses a specific gap in understanding how transdisciplinary teams actually move research into practice. The findings offer actionable guidance for funding bodies and academic institutions on embedding facilitation and distributed leadership to maximize the impact of collaborative research. |
| S044 | Salerno, J., Aguirre, A. A., Aguirre, L. F., et al. (2025). Transdisciplinary research priorities for a One Health approach to human–wildlife coexistence. BioScience, biaf026. <https://doi.org/10.1093/biosci/biaf026> | Low: The paper is primarily a conceptual / agenda‑setting viewpoint, not an empirical primary study or systematic review. It synthesizes existing literature and expert perspectives but does not follow a formal systematic review or transparent evidence‐mapping protocol. Participatory or empirical data (e.g., case studies, stakeholder interviews) are largely described in general terms rather than systematically collected and analyzed. | High: Highly relevant for questions about transdisciplinary, One Health, and systems‑oriented approaches to health at the human–environment interface. Directly addresses research priorities, governance structures, and stakeholder participation, which align closely with inquiries into transdisciplinary, equity‑focused health and sustainability science. Relevance is particularly strong for human–wildlife coexistence, zoonoses, biodiversity, and environmental health, and more indirect for strictly clinical or hospital‑based care contexts. | High: The paper presents a logically coherent argument linking human–wildlife conflict, zoonotic disease, biodiversity loss, and livelihoods within a One Health framing. The proposed research priorities and governance recommendations are internally consistent and well aligned with broader One Health and transdisciplinary literature. However, because it does not systematically appraise or contrast divergent empirical findings, the coherence assessment rests mainly on expert synthesis and conceptual logic rather than on a clearly demonstrated convergence across multiple independent empirical studies. | Moderate: 1. Adequacy (richness and quantity of data contributing to the finding). The article draws on a broad but selectively cited set of examples and literatures from conservation, zoonoses, social science, and One Health, but does not offer any in‑depth empirical case analyses or pooled findings. Quantitative data are minimal; qualitative insights are largely narrative and expert‑driven. As a single viewpoint paper, it provides rich conceptual framing but limited empirical breadth and depth for assessing how well the proposed priorities work in practice. |
| S045 | Shrivastava P, Stafford Smith M, O'Brien K, Zsolnai L. Transforming Sustainability Science to Generate Positive Social and Environmental Change Globally. One Earth. 2020 Apr 24;2(4):329-340.  doi: 10.1016/j.oneear.2020.04.010 | Moderate: The paper is explicitly defined as a “Perspective” rather than a primary empirical study or a formal systematic review. While it is grounded in established theoretical frameworks, such as the “Great Acceleration” and “planetary boundaries” it lacks a formal, reproducible search protocol or experimental design. | High: The findings demonstrate exceptional internal consistency, logically progressing from a critique of current science to a structured “integral framework” for sustainability. The paper successfully harmonizes diverse concepts, such as systems thinking, behavioral psychology, and cultural narratives, into a unified argument for transdisciplinary transformation. | High: The richness and quantity of supporting evidence are substantial, with the paper drawing from 89 unique references. The data are qualitatively and theoretically rich, citing decades of international conferences (e.g., Stockholm 1972, Rio+20), global research programs like Future Earth, and specific case studies from diverse sectors. | High: The work is entirely applicable to the urgent global challenge of meeting the UN Sustainable Development Goals (SDGs) and navigating the risks of the Anthropocene. It provides direct, actionable recommendations for modifying the research ecosystem, including specific policy changes for universities and funding agencies to enhance social impact. |
| S046 | Sather, E.W., Iversen, V.C., Svindseth, M.F. et al. Exploring sustainable care pathways - a scoping review. BMC Health Serv Res 22, 1595 (2022).  [doi.org/10.1186/s12913-022-08863-w](https://doi.org/10.1186/s12913-022-08863-w) | Moderate: The review followed a rigorous five-stage framework by Arksey and O’Malley and complied with PRISMA reporting standards. However, the authors explicitly acknowledge significant flaws in the underlying studies: many featured small sample sizes, no randomization, and a lack of control groups, leading to a “high risk of bias” in the quantitative evidence. Additionally, the researchers noted that expert consultation, an optional but recommended step in scoping reviews, was not feasible for this study. | High: The findings demonstrate strong internal consistency, with results clearly synthesized into six major themes (Organization, Resources, Information, Patients/Families, Clinical Care, and Ethics). The conclusions, such as the need for transparent procedures and the role of IT in improving outcomes, are logically derived from the thematic analysis and are supported by consistent trends across the included reviews and primary studies. | Moderate: The review identifies 28 eligible studies (16 review articles and 12 primary studies) from an initial pool of 283 records. While the search across multiple electronic databases was comprehensive, the authors highlight that the evidence linking care pathways to specific coordination outcomes is “sparse.” The overall richness is somewhat constrained by the small study samples and “implementation problems” found in 16 of the 28 included articles. | High: The paper is entirely applicable to the specific context of organizing mental health services. It directly addresses the “revolving door” phenomenon of psychiatric hospitalization and provides actionable insights regarding the “active ingredients” of care pathways, such as around-the-clock ambulant teams and informed shared decision-making. |
| S047 | Cardol, C. K., Tommel, J., van Middendorp, H., Ciere, Y., Sont, J. K., Evers, A. W., & van Dijk, S. (2021). Detecting and treating psychosocial and lifestyle-related difficulties in chronic disease: development and treatment protocol of the E-GOAL eHealth care pathway. International journal of environmental research and public health, 18(6), 3292.  doi.org/10.3390/ijerph18063292 | Moderate: The paper describes a highly systematic development process using the Behavior Change Wheel (BCW) and iterative co-creation. However, the sample sizes for the user feedback rounds were relatively small (8-14 participants), and the authors explicitly noted a limitation in not specifically involving under-served populations (e.g., those with low eHealth literacy) during the co-creation stages. | High: The findings (the final care pathway) demonstrate exceptional internal consistency. There is a transparent, logical mapping between identified psychosocial barriers and facilitators and the selected Behavior Change Techniques (BCTs) and intervention functions. The theoretical grounding in the COM-B system and TDF ensures the pathway is logically robust. | High: The review draws upon a qualitatively rich evidence base, including data from 8 focus groups with 47 participants and multiple iterative rounds of cognitive and feasibility interviews. The richness of the data are further supported by the integration of 106 unique references and the use of established, validated questionnaires for the screening tool. | High: The paper is entirely applicable to the clinical challenge of integrating psychosocial support with lifestyle management in chronic disease. It addresses the documented “revolving door” of poor adherence and provides an actionable, theory-based tool (E-GOAL) specifically designed for hospital implementation. |
| S048 | Villani, C., Sapio, M., Cassarà, G., Patti, Y., Cupane, L., Giorgi, V., ... & Sarzi-Puttini, P. (2025). Pharmakon or the healing art: experience of artistic-transformative transdisciplinary workshops in fibromyalgia syndrome. Clinical and Experimental Rheumatology, 43, 978-989.  doi: 10.55563/clinexprheumatol/yxhmcr. | Moderate: The study is a prospective observational study without a control group or randomization, which limits the ability to rule out placebo effects or natural symptom fluctuation. Participants chose their own workshops based on personal inclination, introducing selection bias. Furthermore, there were no follow-up evaluations to assess if the significant improvements in sleep and well-being were sustained beyond five days post-workshop. | High: The findings are strongly supported by the underlying data and show internal consistency across the three intervention arms. The total grouped data showed statistically significant improvements in sleep quality (PSQI), self-esteem (RSES), well-being (WHO-5), and global health (EQ5D). The results are logically linked to a PNEI (Psychoneuroendocrine-immunology) framework, explaining how psychological transformation might mediate physiological changes in chronic pain. | High: The study analyzed a robust sample of 109 FM patients who completed all clinimetric tests. The researchers used six validated international scales to measure a wide range of psychophysical parameters. Although the authors noted that the qualitative analysis was limited, the quantitative data derived from the Wilcoxon non-parametric tests provided a substantial evidence base for their conclusions. | High: The work is entirely applicable to the clinical management of Fibromyalgia, a complex syndrome where multidisciplinary and non-pharmacological approaches are explicitly recommended by EULAR guidelines. It provides actionable guidance for integrating “transformative art” into care pathways to address the trauma and stress often underlying FM, potentially reducing the workload for rheumatologists. |
| S049 | Nguyen, P., & Gietzen, L. (2024). Transdisciplinary Collaboration of Healthcare Professionals and the Emotional Connectedness with Working with Patients Coping with Death and Dying. Pacific Journal of Health, 7(1), 35.  [doi.org/10.56031/2576-215X.1085](https://doi.org/10.56031/2576-215X.1085) | Moderate: The study used a qualitative constructivist grounded theory approach, which is robust for exploring complex human experiences. However, the authors utilized convenience sampling and recruited from a single private university, which limits the findings’ generalizability. Additionally, the sample size of 16 participants was small, and the authors admit it may be an insufficient representation of all healthcare professionals. | High: The findings demonstrate a strong internal consistency, with results systematically organized into five major themes derived from coding. The arguments for transdisciplinary collaboration are logically supported by the underlying data, such as the direct link between improved EMR visibility and better team coordination. | Moderate: The data are qualitatively rich, featuring in-depth, semi-structured interviews that allowed for open-ended dialogue across seven distinct healthcare roles. While rich, the quantity is constrained by the small number of participants and the relatively short duration of interviews (averaging 20-40 minutes), which may limit the “saturation” of the data. | High: The work is entirely applicable to the urgent challenges of an aging population and the complexities of end-of-life care. It provides actionable recommendations for clinical practice, including the need for mandated workplace training on POLST forms, cultural humility, and strategies for managing the mental well-being of providers. |
| S050 | Lassell, R. K., Pritchard, K., Baehr, L. A., Ceïde, M. E., Chatterjee, S. A., Colón-Semenza, C., ... & Andonian, B. J. (2025). New horizons in advancing lifestyle medicine for older adults utilizing a transdisciplinary approach: gaps and opportunities. Age and ageing, 54(6), afaf159.  [doi.org/10.1093/ageing/afaf159](https://doi.org/10.1093/ageing/afaf159) | Moderate: The paper describes a proposed framework and gap/opportunity mapping but does not indicate systematic search methods, explicit inclusion criteria, or formal appraisal of primary studies. | Minor concern. The argument (integrating lived experience, 5Ms, and lifestyle pillars) is logically coherent and aligns with established concepts in geriatric and lifestyle medicine; coherence would be strengthened if empirical examples and linked evidence in the full text clearly support each identified gap/opportunity. | Moderate concern. As a framework/review article, adequacy depends on the breadth and depth of the primary evidence synthesized. Based on the goals, there is likely conceptual depth but uncertain empirical richness (e.g., primary qualitative accounts or implementation studies). | Relevance: Minor concern. The focus is directly relevant to older adults and implementation of lifestyle medicine; applicability across settings may vary depending on whether diverse populations.  Overall CERQual confidence Moderate. The proposed framework is credible and useful for framing research and policy, but confidence is limited by likely non‑systematic methods and uncertain depth of primary empirical support as reported in the abstract. |
| S051 | Martins, P., Matos, L., Moreira, A., Simoes, M., & Silva, B. (2025). A Transdisciplinary Approach of the Role of Consciousness and Spirituality in Physics and Healing. Transdisciplinary Journal of Engineering & Science, 16.  [doi.org/10.22545/2025/00273](https://doi.org/10.22545/2025/00273) | Moderate: The paper is a theoretical review and transdisciplinary analysis, rather than a primary empirical study. While it uses a systematic search of PubMed for reviews and meta-analyses regarding Chinese Medicine (Table 1), its core analysis of “spontaneous healing” relies on a single case study of a religious “miracle.” The authors acknowledge the complexity of variables and the difficulty of applying standard placebo controls to the healing methods discussed. | High: The findings demonstrate exceptional internal consistency. The authors logically map diverse healing traditions (Ayurveda, TCM, Reiki) and a contemporary “spontaneous healing” case onto established transdisciplinary frameworks, specifically Ken Wilber’s AQAL model and the nine “fulcrums” of self-development. The progression from historical context to theoretical physics to clinical application is well-structured and consistent. | Moderate: The paper is theoretically rich, drawing on 61 unique references spanning medicine, physics, and philosophy. It provides a quantitative overview of current clinical evidence for TCM through a search yielding thousands of articles. however, the “data” for the paper's most novel claims regarding consciousness and “spontaneous healing” is limited to qualitative narratives and anecdotal evidence that lacks a large-sample empirical base. | High: The work is highly applicable to the field of integrative medicine and the global effort to achieve UN Sustainable Development Goal #3 (ensure healthy lives and promote well-being). It provides a concrete bridge between the current biomedical model and a more holistic, patient-centered approach that includes the psychological and spiritual dimensions of health. |
| S052 | McLure, J., Arnautovska, U., Panizzutti, B., Jiang, J. C., Robinson, S., Drummond, K. D., ... & Berk, M. (2025). New hope for individuals with schizophrenia: A transdisciplinary research platform to discover and translate novel treatments. Australian & New Zealand Journal of Psychiatry, 00048674251342959.  [doi.org/10.1177/00048674251342959](https://doi.org/10.1177/00048674251342959) | Moderate: The paper is an Editorial and “Lived Experience Perspective” rather than a primary empirical study or a formal systematic review. It outlines the design of a future research program (SYNDICAT) starting in mid-2025, meaning it does not yet report results from the proposed methods. However, the described protocol is robust, utilizing validated in vitro models, animal behavioral studies, and adaptive clinical trials. | High: The findings demonstrate exceptional internal consistency. The paper presents a logical “knowledge gain pipeline” (Figure 1) that moves systematically from identifying novel targets (Pillar 1) to validation (Pillar 2) and clinical trials (Pillar 3). The integration of lived experience co-design across all three pillars is a consistent and well-argued thread throughout the text. | Moderate: As an editorial, the paper provides a high-quality theoretical and scientific rationale for the platform, rather than new experimental data. It cites 5 key sources to establish the global burden and current treatment gaps in schizophrenia. While the planned data collection is rich (e.g., whole transcriptome sequencing and large administrative databases), the “data” in this specific source is limited to the synthesis of the platform's intent. | High: The work is entirely applicable to the challenge of addressing complex, “wicked” health problems. It directly addresses a major public health priority, the 40-year lack of treatment breakthroughs for schizophrenia, and provides a concrete model for how transdisciplinary programs can bridge the gap between basic biomedical research and real-world solutions. |

* Four levels are used to describe the overall assessment of confidence: high, moderate, low or very low based on Downe S, Finlayson KW, Lawrie TA, Lewin SA, Glenton C, Rosenbaum S, Barreix M, Tunçalp Ö. Qualitative evidence synthesis for guidelines: Paper 1 – Using qualitative evidence synthesis to inform the guideline scope and develop qualitative findings Health Res Policy Syst (2019) 17:76. and Lewin S, Glenton C, Munthe-Kaas H, Carlsen B, Colvin CJ, Gülmezoglu M, et al. Using qualitative evidence in decision-making for health and social interventions: An approach to assess confidence in findings from qualitative evidence syntheses (GRADE-CERQual). PLoS Med (2015) 12:e1001895. doi: 10.1371/journal.pmed.1001895

**Supplementary Table S19. GRADE-CERQual Overview of Transdisciplinary Studies***

| Finding | Key Approaches | Key Contributing Studies | CERQual Assessment - Methodological Limitations | CERQual Assessment - Coherence | CERQual Assessment - Adequacy of Data | CERQual Assessment - Relevance | Overall CERQual Confidence |
| --- | --- | --- | --- | --- | --- | --- | --- |
| 1: Transdisciplinary, team-based and participatory approaches improve communication and collaboration in chronic/complex illness care | Transdisciplinary clinical teams, participatory approaches, One Health, PAR | S014 Gordon et al. 2014, S026 Martin et al. 2022, S037 Bogerd et al. 2024, S049 Nguyen & Gietzen 2024, S017 Allen-Scott et al. 2015, S043 Archibald et al. 2023 | Most studies qualitative, mixed-methods, scoping reviews or realist evaluations; sample sizes modest; reliance on self-reported perceptions | Consistent description that shared goals, blended roles, structured communication channels improve coordination and perceived care quality; S043 reinforces knowledge translation practices | Multiple studies across hospital, primary care, palliative, One Health research centers; rich qualitative/descriptive data; few large controlled trials | Directly address complex, chronic conditions; some contextual details may limit transferability, general mechanisms highly relevant | Moderate confidence |
| 2: Transformative, participatory, and mind–body-oriented transdisciplinary interventions can improve psychological wellbeing, self-efficacy, and coping | Artistic-transformative workshops, integrative medicine, mindfulness, PAR, CBPR | S048 Villani et al. 2025, S028 Martins 2018, S051 Martins et al. 2025, S025 Zeidan & Vago 2016, S029 Schuman-Olivier et al. 2020, S020 Keng et al. 2011, S024 Marikar Bawa et al. 2023, S037 Bogerd et al. 2024, S018 Collins et al. 2018, S035 Thomas et al. 2023 | Several studies were observational, qualitative, or pre–post without control groups; mindfulness studies include some RCTs and neuroimaging; PAR/CBPR strong for process/context but not effect estimates | Coherent pattern: participants report improvements in wellbeing, self-efficacy, coping; improvements linked to meaning-making, emotion regulation, identity, social support; no clear contradictions | Multiple studies across fibromyalgia, chronic pain, multimorbidity, serious mental illness, settings, countries; few large, long-term RCTs; rich for mechanisms and short- to medium-term outcomes | Strong relevance to chronic pain and chronic illness; some work more theoretical/philosophical; contexts/populations align well | Low-to-moderate confidence |
| 3: Systems/complexity and process-oriented transdisciplinary lens supports sustainable, person-centered care pathways | Systems science, complexity science, sustainable pathways, lifestyle medicine, PAR, eHealth | S012 Carey et al. 2015, S040 Rusoja et al. 2018, S039 Kwamie et al. 2024, S031 Morgan et al. 2024, S046 Sather et al. 2022, S050 Lassell et al. 2025, S037 Bogerd et al. 2024, S047 Cardol et al. 2021 | Many studies are scoping/systematic reviews, conceptual/agenda-setting papers, qualitative case studies/PAR; strong for mapping concepts, frameworks, perceived impacts; weak for empirical tests | Consistent argument: complex health problems require systems/process views; sustainable pathways integrate patient perspectives, social determinants, organizational structures; person-centered trajectories needed; logic coherent, empirical demonstration emergent | Data broad but concept-heavy; substantial literature on systems/complexity ideas; smaller body on sustainable care pathways/lifestyle medicine; some concrete PAR-based implementations; limited direct quantitative evidence | Few studies pain-specific; address multi-level, long-term challenges of chronic pain/illness; principles relevant and transferable | Moderate confidence |

Review period: January 2009 to December 2025; n = 93 included studies; GRADE-CERQual assessments were independently conducted by EAV and SSA with consensus adjudication by the full team; full search strings (MeSH/keywords), study lists and IDs, and complete CERQual evidence profiles are provided in Supplementary Tables S3 (search terms), S15 (study summaries and IDs), and S16 (GRADE‑CERQual profiles).

**Supplementary Table S20. GRADE-CERQual Summary of Findings for Transdisciplinary Studies**

| Review Finding | Methodological Limitations | Coherence | Adequacy of Data | Relevance | Overall CERQual style confidence |
| --- | --- | --- | --- | --- | --- |
| **Finding 1:** Transdisciplinary, team-based and participatory approaches improve communication and collaboration across ontological domains of chronic illness and chronic pain, supporting more coherent, person-centered care. In our synthesis, qualitative evidence suggests that when clinical teams adopt explicitly transdisciplinary and participatory practices (e.g., shared goal setting, blended roles, co-production with patients), communication improves across somatic, psychological, social, and cognitive domains. This leads to more integrated understandings of patients’ lived experiences and more coherent care plans. | Minor to moderate concerns. Contributing studies (e.g., S014, S026, S037, S049, S017, S043) are largely qualitative, mixed-methods, scoping reviews, PAR, and realist evaluations. Designs are generally appropriate to explore communication, collaboration, and lived experience, but many rely on self-report, observational data, and process measures rather than controlled comparisons or long-term outcome data. | Minor concerns. Across chronic pain teams (S014), hospital allied health models (S026), multimorbidity care (S037), end-of-life care (S049), and transdisciplinary research teams (S017, S043), findings consistently describe improved communication and collaboration when teams share language, goals, and decision-making with patients. There are no substantial contradictions between studies. | Moderate concerns. Multiple studies from different countries and clinical contexts provide rich narrative and observational data about communication processes, perceived improvements in coordination, and therapeutic relationships. However, there are relatively few large studies directly quantifying the impacts on patient-level outcomes (e.g., pain severity, function) or health-system measures over time. | Minor concerns. The phenomena under study—chronic pain, multimorbidity, palliative trajectories, and complex public health problems—closely parallel the complex, co-emergent somatic, psychological, social, and cognitive processes at the center of our phenomenological and ontological synthesis. Some context specificity (e.g., health system structures) may limit direct transfer, but mechanisms are broadly relevant. | Moderate confidence. We are moderately confident that transdisciplinary, team-based and participatory approaches enhance communication and collaboration in chronic illness and chronic pain care, allowing ontologically richer, more coherent, and person-centered care plans. More outcome-oriented longitudinal studies would strengthen the conclusions. |
| **Finding 2:** Transformative, participatory, and mind–body-oriented transdisciplinary interventions support shifts in meaning-making, self-efficacy, and coping in chronic illness and chronic pain, even when symptoms persist. The synthesis indicates that interventions integrating narrative, artistic, spiritual, and mindfulness-based elements within participatory, transdisciplinary frameworks can transform how patients understand and inhabit their illness. These approaches appear to enhance psychological wellbeing, self-efficacy, and coping, without necessarily eliminating pain or other somatic symptoms, reflecting a shift in lived ontology and identity. | Moderate concerns. Many contributing studies are qualitative, pre–post, or PAR case studies (e.g., S048, S018, S035, S037). Mindfulness and mind–body evidence (S020, S025, S029, S024) includes some RCTs and mechanistic work but often in mixed or non-pain-specific samples. Designs are strong for phenomenological insight and understanding mechanisms of change, but less suited to precise causal effect estimates or cross-condition generalizability. | Minor concerns. Across artistic-transformative fibromyalgia workshops (S048), integrative medicine and spirituality frameworks (S028, S051), mindfulness and behavior-change studies (S020, S025, S029, S024), and participatory multimorbidity and mental health work (S037, S035, S018), a coherent pattern can be observed: participants report enhanced meaning-making, acceptance, self-efficacy, and coping, often despite ongoing symptoms. No major conflicting findings are evident. | Moderate concerns. The body of evidence spans several conditions (fibromyalgia, chronic pain, multimorbidity, serious mental illness) and cultural contexts, providing rich qualitative material on psychological, social, and spiritual shifts. However, there are few large, long-term evaluations of complex, explicitly transdisciplinary packages, and limited data on durability of effects and economic outcomes. | Minor to moderate concerns. The included studies map closely onto the ontological domains we synthesized—somatic, psychological, social, cognitive, and spiritual—and demonstrate how transdisciplinary practices reshape patients’ lived experience of chronicity. Some evidence is conceptual or theoretical (S028, S051), rather than condition-specific, supporting conceptual relevance more than direct clinical generalization. | Low-to-moderate confidence. We are cautiously confident that transformative, participatory, and mind–body-oriented transdisciplinary approaches can improve psychological wellbeing, self-efficacy, and coping in chronic illness and chronic pain by reshaping patients’ experiential ontology. Higher-confidence conclusions would require more rigorous, long-term evaluations of such complex interventions in well-characterized chronic pain/illness populations. |
| **Finding 3:** Systems/complexity-informed and process-oriented transdisciplinary perspectives offer promising conceptual foundations for sustainable, person-centered chronic illness and chronic pain pathways that attend to social, economic, and environmental determinants, but empirical testing remains limited. Our synthesis suggests that applying complexity science, sustainability science, and process ontology to chronic illness and pain reframes these conditions as dynamic, relational processes rather than static states. This conceptual move underpins the design of person-centered, longitudinal pathways that integrate somatic, psychological, social, economic, and environmental determinants, although direct empirical evaluations are still emerging. | Moderate to serious concerns. Contributing evidence (e.g., S012, S040, S039, S031, S046, S050, S037, S047) includes scoping and systematic reviews, conceptual and agenda-setting papers, and qualitative/PAR case studies. These designs are appropriate for theory-building and framework development but provide limited direct, empirical comparison of systems-designed vs conventional pathways in chronic pain or chronic illness. | Minor to moderate concerns. Across systems and complexity reviews (S012, S040, S039), sustainable care pathway and lifestyle medicine work (S046, S050), and PAR-based trajectory-focused interventions (S037, S047), there is a coherent argument that chronic conditions require process-oriented, determinant-attentive, and person-centered approaches. However, empirical demonstrations of improved outcomes specifically attributable to this ontological and systems reframing remain sparse. | Moderate to serious concerns. The data are broad and conceptually rich, but thin in terms of robust empirical evaluations in chronic pain or multimorbid populations. There are few longitudinal, comparative studies demonstrating that complexity-informed, process-based pathways outperform standard care on patient, equity, or cost outcomes. | Minor concerns. Although many studies focused on non-communicable diseases, multimorbidity, ageing, and community health rather than pain per se; the characteristics align with the complex, enduring, multi-determinant nature of chronic illness and chronic pain described in our phenomenological–ontological synthesis. The conceptual frameworks are highly relevant and transferable. | Moderate confidence. We currently have moderate confidence—driven primarily by methodological and adequacy concerns—that systems/complexity-informed and process-oriented transdisciplinary perspectives empirically improve outcomes in chronic illness and chronic pain. Nonetheless, the conceptual and phenomenological fit with the lived ontology of chronic conditions is strong, supporting their further development and testing. |

**Supplementary Table S21. Ontological Framework and Methodological Integration: Heuristic, Process, and Relational ontologies**

**Operationalized in the Sequential Synthesis (links to Codebook, Weighting Rules, and Visualizations in Supplementary Tables).**

| Ontology | Sequential Steps | Heuristic Approach | Hermeneutic Approach | Transdisciplinary Integration | Solution vs. Problem-Focus Balance | Unique Contribution |
| --- | --- | --- | --- | --- | --- | --- |
| Heuristic Ontology | Concepts defined by weight of evidence and rules-of-thumb rather than fixed axiomatic definitions (Alhazmi and Kaufmann, 2022). Iterative category refinement throughout. No predetermined coding schemes imposed on data. Utilizing heuristic methods within a process-ontological framework facilitates progress in analysis prior to the development of epistemic procedures. | Heuristic ontologies are rule-of-thumb guidelines rather than strict axioms (Stock et al. 2015; Wenzel & Stjerne, 2021; Turska and Ludwig 2023). These allow for guiding principles to be established, while acknowledging an elevated level of ambiguity. Heuristic ontology as a core operating principle of the methodology: enables structured exploration and discovery without rigid categories. | Compatible with hermeneutic openness, allows interpretive frameworks to remain provisional and revisable throughout the research cycle. No interpretation treated as final. | No single disciplinary framework dominates the synthesis functions to facilitate transdisciplinary engagement. Ontology is co-constructed during the synthesis, not fixed beforehand. | Problem-focused: addresses the inadequacy of classical ontological approaches (which tend to be static) for phenomena characterized by fluctuation, relational complexity, and contextual variability**.** | By incorporating heuristic ontologies as the foundational logic of a qualitative research synthesis, ontological commitments are actively shaped rather than remaining implicit or inert. |
| Process Ontology | Meta-ethnographic design grounded in process and relational ontologies. Chronic illness and chronic pain are conceptualized as ongoing processual phenomena rather than fixed entities. Temporal language treated as analytically significant. Clinical presentations understood as culminations of a range of factors rather than stable states. | Heuristic domain mapping tracks processes over time. Sub-constructs varying by severity, duration, and population identified (Finnern et al., 2018). | Hermeneutic engagement is sensitive to the impermanence and interdependence of phenomena. Meaning structures understood as arising and dissolving over time in different contexts, rather than as stable fixed phenomena to be uncovered. | Process perspective applied across all levels of ontological reality—from biology through personal experience to social interaction—thus providing a common ontological language for integration across disciplines. | Both. Developed in response to the problem of the drawbacks of non-process ontology, i.e. traditional approaches that treat pain as a “fixed” phenomenon. Frames the problem dynamically while enabling solution pathways that account for change over time and the varied trajectories of chronic illness | Grounding qualitative health synthesis in process ontology—rather than substance ontology—enables tracking of experiential trajectories rather than categorical states, resolving the homogenization risk that phenomenological synthesis faces (Booth et al., 2019). |
| Relational Ontology | Meta-ethnographic design grounded in process and relational ontologies. Phenomena understood as constituted through relationships. Analysis encompasses family systems, care relationships, and broader social contexts. Informs the research teams relationally constituted collaborative process. | An approach to charting how chronic illness and pain relate to social, cultural, physical, cognitive, and psychological factors, using analogies to explain the links. | Places focus on relationships between entities rather than the entities themselves, informing a collaborative research design. | Relational ontology is inherently transdisciplinary. The strategy of knowledge co-production is a relational endeavor. Stakeholders, researchers, and disciplines understood as mutually constituting the synthesis, rather than as independent contributors. | Solution-focused. The relational frame identifies systemic deficiencies and proposes participatory, co-produced interventions for chronic pain management. | Establishing a mutually reinforcing relationship between relational and process ontologies and operationalizing them together in a qualitative synthesis. The unified ontological framework incorporates the strengths of both. |

# 3 Supplementary References

**Additional References Methodologies:**

1. Alhazmi AA, Kaufmann A. Phenomenological qualitative methods applied to the analysis of cross-cultural experience in novel educational social contexts. Front Psychol (2022) 13:785134.
2. Bondas TE. Representations of meta-ethnography: In limbo in the jungle of misguided paths? Qual Health Res (2025) 10497323251316841.
3. Booth A, Mshelia S, Analo CV, Nyakang'o SB. Qualitative evidence syntheses: Assessing the relative contributions of multi-context and single-context reviews. J Adv Nurs (2019) 75:3812–3822.
4. Chang J. Hermeneutic inquiry: A research approach for postmodern therapists. J Syst Ther (2022) 41:56–67.
5. Dixon-Woods M, Cavers D, Agarwal S, Annandale E, Arthur A, Harvey J, et al. Conducting a critical interpretive synthesis of the literature on access to healthcare by vulnerable groups. BMC Med Res Methodol (2006) 6:35.
6. Downe S, Finlayson KW, Lawrie TA, Lewin SA, Glenton C, Rosenbaum S, Barreix M, Tunçalp Ö. Qualitative evidence synthesis for guidelines: Paper 1 – Using qualitative evidence synthesis to inform guideline scope and develop qualitative findings statements. Health Res Policy Syst (2019) 17:76.
7. Fetters MD, Curry LA, Creswell JW. Achieving integration in mixed methods designs: Principles and practices. Health Serv Res (2013) 48:2134–2156. doi: 10.1111/1475-6773.12117
8. Fillingim M, Tanguay-Sabourin C, Parisien M, Zare A, Guglietti GV, Norman J, et al. Biological markers and psychosocial factors predict chronic pain conditions. Nat Hum Behav (2025) 9:1710–1725. doi: 10.1038/s41562-025-02156-y
9. Finlay L. Negotiating the swamp: The opportunity and challenge of reflexivity in research practice. Qual Res (2002) 2:209–230.
10. Finnern MM, Kleinböhl D, Flor H, Benrath J, Hölzl R. Deconstructing chronicity of musculoskeletal pain: Intensity-duration relations, minimal dimensions and clusters of chronicity. Scand J Pain (2018) 18:363–377.
11. Ho KHM, Cheung DSK, Bourke S, Edvardsson D. Reflexivity in Heideggerian hermeneutic phenomenology: The hermeneutic phenomenological circle. J Adv Nurs (2026) 82:957–965. doi: 10.1111/jan.16950
12. Hong QN, Pluye P, Fàbregues S, Bartlett G, Boardman F, Cargo M, et al. Improving the content validity of the mixed methods appraisal tool: A modified e-Delphi study. J Clin Epidemiol (2019) 111:49–59.
13. Horcea-Milcu A-I, Leventon J, Lang DJ. Making transdisciplinarity happen: Phase 0, or before the beginning. Environ Sci Policy (2022) 136:187–197. doi: 10.1016/j.envsci.2022.05.019
14. Hosseini M-S, Jahanshahlou F, Akbarzadeh MA, Zarei M, Vaez-Gharamaleki Y. Formulating research questions for evidence-based studies. J Med Surg Public Health (2024) 2:100046. doi: 10.1016/j.glmedi.2023.100046
15. Kleining G, Witt H. The qualitative heuristic approach: A methodology for discovery in psychology and the social sciences. Forum Qual Soc Res (2000) 1.
16. Lang DJ, Wiek A, Bergmann M, Stauffacher M, Martens P, Moll P, et al. Transdisciplinary research in sustainability science: Practice, principles, and challenges. Sustain Sci (2012) 7:25–43. doi: 10.1007/s11625-011-0149-x
17. Lather P. Scientific research in education: A critical perspective. Br Educ Res J (2004) 30:759–772.
18. Lewin S, Glenton C, Munthe-Kaas H, Carlsen B, Colvin CJ, Gülmezoglu M, et al. Using qualitative evidence in decision making for health and social interventions: An approach to assess confidence in findings from qualitative evidence syntheses (GRADE-CERQual). PLoS Med (2015) 12:e1001895. doi: 10.1371/journal.pmed.1001895
19. Long HA, French DP, Brooks JM. Optimising the value of the critical appraisal skills programme (CASP) tool for quality appraisal in qualitative evidence synthesis. Res Methods Med Health Sci (2020) 1:31–42. doi: 10.1177/2632084320947559
20. Nikulina V, Larson Lindal J, Baumann H, Simon D, Ny H. Lost in translation: A framework for analysing complexity of co-production settings in relation to epistemic communities, linguistic diversities and culture. Futures (2019) 113:102442. doi: 10.1016/j.futures.2019.102442
21. Noblit GW, Hare RD. Meta-ethnography: Synthesizing qualitative studies. Sage (1988).
22. Pawson R, Greenhalgh T, Harvey G, Walshe K. Realist review — a new method of systematic review designed for complex policy interventions. J Health Serv Res Policy (2005) 10:21–34.
23. Reiners GM. Understanding the differences between Husserl's (descriptive) and Heidegger's (interpretive) phenomenological research. J Nurs Care (2012) 1:1–3.
24. Rigolot C. Transdisciplinarity as a discipline and a way of being: Complementarities and creative tensions. Humanit Soc Sci Commun (2020) 7:100. doi: 10.1057/s41599-020-00598-5
25. Snowden D. Multi-ontology sense making: A new simplicity in decision making. J Innov Health Inform (2005) 13:45–53. doi: 10.14236/jhi.v13i1.578
26. Stock K, Leibovici D, Delazari L, Santos R. Discovering order in chaos: Using a heuristic ontology to derive spatio-temporal sequences for cadastral data. Spat Cogn Comput (2015) 15:115–141.
27. Thomas J, Harden A. Methods for the thematic synthesis of qualitative research in systematic reviews. BMC Med Res Methodol (2008) 8:45.
28. Turska JJ, Ludwig D. Back by popular demand, ontology. Synthese (2023) 202:39. doi: 10.1007/s11229-023-04243-x
29. van Breda J, Swilling M. The guiding logics and principles for designing emergent transdisciplinary research processes: Learning experiences and reflections from a transdisciplinary urban case study in Enkanini informal settlement, South Africa. Sustain Sci (2019) 14:823–841. doi: 10.1007/s11625-018-0606-x
30. Vienni-Baptista B, Pohl CE. Exploring interdisciplinarity and transdisciplinarity as knowledge regimes: A heuristic tool for disentangling understandings in academia and policy. Sci Technol Hum Values (2024) 49:1309–1348.
31. Wenzel M, Stjerne IS. Heuristics-in-use: Toward a practice theory of organizational

heuristics. Technol Forecast Soc Change (2021) 164:120517. doi: 10.1016/j.techfore.2020.120517

**References of the first phase of the synthesis: Hermeneutic Phenomenology**

1. Albán-Reyes AP, Tamayo-Montoya PA, Giraldo-Jiménez CF, Ordoñez-Mora LT. Coping strategies for combating chronic pain among professional ballet dancers in Cali, Colombia: qualitative study. J Bodyw Mov Ther (2024) 40:2082–90.
2. Al Kalaldeh M, Shosha GA, Saiah N, Salameh O. Dimensions of phenomenology in exploring patient’s suffering in long-life illnesses: qualitative evidence synthesis. J Patient Exp (2018) 5:43–9.
3. Altun A, Brown H, Sturgiss E, Russell G. Experiences of Assyrian refugee women seeking care for chronic pain: a qualitative study. Int J Equity Health (2023) 22:83.
4. Andersen AH, Assing Hvidt E, Huniche L, Hvidt NC, Roessler KK. Why we suffer? Existential challenges of patients with chronic illness: a Kierkegaardian inspired interpretative phenomenological analysis. J Humanist Psychol (2021) 00221678211002439.
5. Angel S, Jensen LD, Maribo T, Gonge BK, Buus N. Narratives of life with long-term low back pain: a follow-up interview study. Scand J Pain (2017) 17:382–9.
6. Barber M, Evans S, Marks R, Sheedy J, Miller R, Lopez S, O’Shea M. “I am not pain, I have pain”: a pilot study examining iRest yoga nidra as a mind-body intervention for persistent pain. Complement Ther Clin Pract (2025) 59:101955.
7. Bath B, Jaindl B, Dykes L, Coulthard J, Naylen J, Rocheleau N, et al. Get ‘er done: experiences of Canadian farmers living with chronic low back disorders. Physiother Can (2019) 71:24–33.
8. Bosco V, Mercuri C, Doldo P, Nocerino R, Guillari A, Virgolesi M, et al. The lived experience of adults with inflammatory bowel disease in rural areas: a phenomenological study. Nurs Health Sci (2025) 27:e70058.
9. Brooks EA, Unruh A, Lynch ME. Exploring the lived experience of adults using prescription opioids to manage chronic noncancer pain. Pain Res Manag (2015) 20:15–22.
10. Bruger J, Semlyen J, Ford CEL. “I will try anything”: the experience of working-age stroke survivors living with chronic post-stroke pain: an interpretative phenomenological analysis. Disabil Rehabil (2024) 46:4745–54.
11. Bullo S, Hearn JH. Visual reconstructions of endometriosis pain: an interdisciplinary visual methodology for illness representation. Br J Health Psychol (2024) 29:676–93.
12. Chala MB, Donnelly C, Ghahari S, Wondie Y, Abebe A, Miller J. An interpretative phenomenological analysis of living with chronic low back pain in Ethiopia. J Pain Res (2022) 15:4031–45.
13. Cheng C, Inder K, Chan SW. Patients’ experiences of coping with multiple chronic conditions: a meta-ethnography of qualitative work. Int J Ment Health Nurs (2019) 28:54–70.
14. Chimezie RO. Health awareness: a significant factor in chronic diseases prevention and access to care. J Biosci Med (2023) 11:64–79.
15. Coleman B, Ellis-Caird H, McGowan J, Benjamin MJ. How sickle cell disease patients experience, understand and explain their pain: an interpretative phenomenological analysis study. Br J Health Psychol (2016) 21:190–203.
16. Cooper L, Ells L, Ryan C, Martin D. Perceptions of adults with overweight/obesity and chronic musculoskeletal pain: an interpretative phenomenological analysis. J Clin Nurs (2018) 27:e776–86.
17. Daker-White G, Donovan J, Campbell R. Redefined by illness: meta-ethnography of qualitative studies on the experience of rheumatoid arthritis. Disabil Rehabil (2014) 36:1061–71.
18. Danes-Daetz C, Wainwright JP, Goh SL, McGuire K, Sinsurin K, Richards J, et al. Perceptions of stigma associated with chronic knee pain: voices of selected women in Thailand and Malaysia. Physiother Theory Pract (2025) 41:405–19.
19. de Villiers M, Maree JE, van Belkum C. The influence of chronic pain on the daily lives of underprivileged South Africans. Pain Manag Nurs (2015) 16:96–104.
20. Dueñas M, Ojeda B, Salazar A, Mico JA, Failde I. A review of chronic pain impact on patients, their social environment and the health care system. J Pain Res (2016) 9:457–67.
21. Dysvik E, Kvaløy JT, Furnes B. A mixed-method study exploring suffering and alleviation in participants attending a chronic pain management programme. J Clin Nurs (2014) 23:865–76.
22. Fracso D, Bourrel G, Jorgensen C, Fanton H, Raat H, Pilotto A, et al. The chronic disease self-management programme: a phenomenological study for empowering vulnerable patients with chronic diseases included in the EFFICHRONIC project. Health Expect (2022) 25:947–58.
23. Furnes B, Dysvik E. Dealing with grief related to loss by death and chronic pain: an integrated theoretical framework. Part 1. Patient Prefer Adherence (2010) 4:135–40.
24. Furnes B, Natvig GK, Dysvik E. Suffering and transition strategies in adult patients attending a chronic pain management programme. J Clin Nurs (2015) 24:707–16.
25. Gaber SN, Guerrero M, Rosenberg L. Participatory research approaches in long-term care facilities for older adults: a meta-ethnography. Int J Qual Stud Health Well-being (2024) 19:2431449.
26. Gutke A, Bullington J, Lund M, Lundberg M. Adaptation to a changed body: experiences of living with long-term pelvic girdle pain after childbirth. Disabil Rehabil (2018) 40:3054–60.
27. Hansson KS, Fridlund B, Brunt D, Hansson B, Rask M. The meaning of the experiences of persons with chronic pain in their encounters with the health service. Scand J Caring Sci (2011) 25:444–50.
28. Hassani P, Izadi-Avanji FS, Rakhshan M, Majd HA. A phenomenological study on resilience of the elderly suffering from chronic disease: a qualitative study. Psychol Res Behav Manage (2017) 10:59–67.
29. Hazaveh M, Hovey R. Patient experience of living with orofacial pain: an interpretive phenomenological study. JDR Clin Trans Res (2018) 3:264–71.
30. Hearn JH, Cotter I, Fine P, Finlay KA. Living with chronic neuropathic pain after spinal cord injury: an interpretative phenomenological analysis of community experience. Disabil Rehabil (2015) 37:2203–11.
31. Hearn JH, Selvarajah S, Kennedy P, Taylor J. Stigma and self-management: an interpretative phenomenological analysis of the impact of chronic recurrent urinary tract infections after spinal cord injury. Spinal Cord Ser Cases (2018) 4:12.
32. Honoré Grauslund AM, Solmunde Michelsen J, Esbensen BA. Everyday life with chronic back pain: a qualitative study among Turkish immigrants in Denmark. Disabil Rehabil (2021) 43:1162–70.
33. Hughes M, Burton AE, Dempsey RC. “I am free in my wheelchair but pain does have a say in it though”: the meaning and experience of quality of life when living with paraplegia and chronic pain. J Health Psychol (2019) 24:1356–67.
34. Jiang Y, Shi Z, Liu A. Experiences of social isolation in older adults with chronic diseases: a qualitative study based on patients’ perspectives. Healthcare (Basel) (2025) 13:902.
35. Joyce C, Keysor J, Stevans J, Ready K, Roseen EJ, Saper RB. Beyond the pain: a qualitative study exploring the physical therapy experience in patients with chronic low back pain. Physiother Theory Pract (2023) 39:803–13.
36. Juuso P, Skär L, Olsson M, Söderberg S. Meanings of being received and met by others as experienced by women with fibromyalgia. Qual Health Res (2014) 24:1381–90.
37. Kahere M, Hlongwana K, Ginindza T. Exploring patients’ lived experience on the barriers to accessing low back pain health services. Afr J Prim Health Care Fam Med (2022) 14:e1–10.
38. Keen S, Lomeli-Rodriguez M, Williams A. Exploring how people with chronic pain understand their pain: a qualitative study. Scand J Pain (2021) 21:743–53.
39. Kirkham JA, Smith JA, Havsteen-Franklin D. Painting pain: an interpretative phenomenological analysis of representations of living with chronic pain. Health Psychol (2015) 34:398–406.
40. Kjærulff EM, Andersen TH, Kingod N, Nexø MA. When people with chronic conditions turn to peers on social media to obtain and share information: systematic review of the implications for relationships with health care professionals. J Med Internet Res (2023) 25:e41156.
41. Krohne K, Ihlebaek C. Maintaining a balance: a focus group study on living and coping with chronic whiplash-associated disorder. BMC Musculoskelet Disord (2010) 11:158.
42. Lavie-Ajayi M, Almog N, Krumer-Nevo M. Chronic pain as a narratological distress: a phenomenological study. Chronic Illn (2012) 8:192–200.
43. Leder D. The phenomenology of healing: eight ways of dealing with the ill and impaired body. J Med Philos (2022) 47:137–54.
44. Lee AL, Harrison SL, Goldstein RS, Brooks D. An exploration of pain experiences and their meaning in people with chronic obstructive pulmonary disease. Physiother Theory Pract (2018) 34:765–72.
45. Lilja V, Wallström S, Saarijärvi M, Lundberg M, Segertoft VA, Ekman I. Balancing between being the most valuable player (MVP) and passing the ball: a qualitative study of support when living with chronic pain in Sweden. BMJ Open (2024) 14:e079229.
46. Lima DD, Alves VL, Turato ER. The phenomenological-existential comprehension of chronic pain: going beyond the standing healthcare models. Philos Ethics Humanit Med (2014) 9:2.
47. Lo Monaco M, Alblooshi S, Mallaci Bocchio R, Natoli G, Landa ML, Corrao S. The lived experience of mothers living with fibromyalgia syndrome: a phenomenological inquiry. Musculoskeletal Care (2024) 22:e1889.
48. Lundin Å, Ekman I, Andréll P, Lundberg M, Wallström S. Have my back as I get back to work—experiences of stakeholder support in returning to work after sick leave due to chronic pain: a qualitative interview study. PLoS One (2024) 19:e0312478.
49. Mahon G, O’Brien B, O’Conor L. The experience of chronic illness among a group of Irish patients: a qualitative study. J Res Nurs (2013) 19:330–42.
50. Macgregor C, Blane DN, Tulle E, Campbell CL, Barber RJ, Hill O-Connor C, et al. An ecosystem of accepting life with chronic pain: a meta-ethnography. Br J Pain (2024) 18:365–81.
51. MacNeela P, Doyle C, O’Gorman D, Ruane N, McGuire BE. Experiences of chronic low back pain: a meta-ethnography of qualitative research. Health Psychol Rev (2015) 9:63–82.
52. Mathias B, Parry-Jones B, Huws JC. Individual experiences of an acceptance-based pain management programme: an interpretative phenomenological analysis. Psychol Health (2014) 29:279–96.
53. Matias-Soto J, Pineda-Galan C, Martin-Sanchez AI, Gonzalez-Mesa JM, Aguayo-Alves A, Luque-Suarez A. Unveiling relevant emotions, cognitions, and behaviours from the viewpoint of people with chronic low back pain: a qualitative study with patient involvement. Musculoskelet Sci Pract (2024) 72:103123.
54. McGeechan GJ, McPherson KE, Roberts K. An interpretative phenomenological analysis of the experience of living with colorectal cancer as a chronic illness. J Clin Nurs (2018) 27:3148–56.
55. Mengshoel AM, Sim J, Ahlsen B, Madden S. Diagnostic experience of patients with fibromyalgia: a meta-ethnography. Chronic Illn (2018) 14:194–211.
56. Merolli M, Gray K, Martin-Sanchez F. Therapeutic affordances of social media: emergent themes from a global online survey of people with chronic pain. J Med Internet Res (2014) 16:e284.
57. Morton K, Dennison L, May C, Murray E, Little P, McManus RJ, Yardley L. Using digital interventions for self-management of chronic physical health conditions: a meta-ethnography review of published studies. Patient Educ Couns (2017) 100:616–35.
58. Mustafa N, Einstein G, MacNeill M, Watt-Watson J. The lived experiences of chronic pain among immigrant Indian-Canadian women: a phenomenological analysis. Can J Pain (2020) 4:40–50.
59. Najem C, Wijma AJ, Meeus M, Cagnie B, Ayoubi F, Van Oosterwijck J, et al. “It is something you live with, like an organ in your body”: a qualitative study on the lived experiences of people suffering from chronic low back pain in Lebanon. Disabil Rehabil (2025) 47:1795–806.
60. Nay R, Fetherstonhaugh D. What is pain? A phenomenological approach to understanding. Int J Older People Nurs (2012) 7:233–9.
61. Nikoloudi M, Bogdani E, Tsatsou I, Mantoudi A, Mystakidou K. Experiences of patients living in a unique leprosy hospice in Greece: an interpretative phenomenological analysis. Cureus (2024) 16:e66358.
62. Nilsen G, Anderssen N. Struggling for a normal life: work as an individual self-care management strategy among persons living with non-malignant chronic pain. Work (2014) 49:123–32.
63. Niño de Guzmán Quispe E, Martínez García L, Orrego Villagrán C, Heijmans M, Sunol R, Fraile-Navarro D, et al. The perspectives of patients with chronic diseases and their caregivers on self-management interventions: a scoping review of reviews. Patient (2021) 14:719–40.
64. Ojala T, Häkkinen A, Karppinen J, Sipilä K, Suutama T, Piirainen A. Although unseen, chronic pain is real: a phenomenological study. Scand J Pain (2015) 6:33–40.
65. Ojala T, Häkkinen A, Karppinen J, Sipilä K, Suutama T, Piirainen A. Chronic pain affects the whole person: a phenomenological study. Disabil Rehabil (2015) 37:363–71.
66. Ojala T, Häkkinen A, Karppinen J, Sipilä K, Suutama T, Piirainen A. Revising the negative meaning of chronic pain: a phenomenological study. Chronic Illn (2015) 11:156–67.
67. Ojala T, Häkkinen A, Karppinen J, Sipilä K, Suutama T, Piirainen A. The dominance of chronic pain: a phenomenological study. Musculoskeletal Care (2014) 12:141–9.
68. Okolo CA, Olorunsogo T, Babawarun O. Cultural variability in pain perception: a review of cross-cultural studies. Int J Sci Res Arch (2024) 11:2550–6.
69. Palacios-Ceña D, Neira-Martín B, Silva-Hernández L, Mayo-Canalejo D, Florencio LL, Fernández-de-Las-Peñas C, et al. Living with chronic migraine: a qualitative study on female patients’ perspectives from a specialised headache clinic in Spain. BMJ Open (2017) 7:e017851.
70. Peppard SW, Burkard J, Georges J, Dye J. The lived experience of military women with chronic pain: a phenomenological study. Mil Med (2023) 188:1199–206.
71. Putri LD, Amir AS. Exploring the lived experiences of chronic illness patients: understanding the dual role of emotional and psychological aspects, communication, and social support. CommVersa (2025) 1:26–34.
72. Rambod M, Sharif F, Molazem Z, Khair K. Pain experience in hemophilia patients: a hermeneutic phenomenological study. Int J Community Based Nurs Midwifery (2016) 4:309–19.
73. Rodríguez I, Abarca E, Herskovic V, Campos M. Living with chronic pain: a qualitative study of the daily life of older people with chronic pain in Chile. Pain Res Manag (2019) 2019:8148652.
74. Roenne PF, Horn NS, Hansen CA. Involvement of relatives in chronic non-malignant pain rehabilitation at multidisciplinary pain centres: part one—the patient perspective. Scand J Pain (2020) 21:81–94.
75. Röing M, Sanner M. A meta-ethnographic synthesis on phenomenographic studies of patients’ experiences of chronic illness. Int J Qual Stud Health Well-being (2015) 10:26279.
76. Safour W, Hovey R. The impact of chronic temporomandibular disorders on fatigue and daily life: a qualitative phenomenological study. JDR Clin Trans Res (2025) 10:398–405.
77. Sanabria-Mazo JP, Colomer-Carbonell A, Gandara-Urrutia N, Pérez-Sutil JM, Noboa-Rocamora G, Fernández-Vázquez Ó, et al. Experiences of patients with chronic low back pain plus comorbid depressive symptoms in a videoconference group acceptance and commitment therapy or behavioral activation treatment for depression: a qualitative study. Disabil Rehabil (2024) 46:5052–63.
78. Schwarz B, Banaschak H, Heyme R, von Kardorff E, Reims N, Streibelt M, et al. A mega-ethnography of qualitative meta-syntheses on return to work in people with chronic health conditions. Rehabilitation (Stuttg) (2024) 63:39–50.
79. Schultz R, Kousgaard MB, la Cour P, Davidsen AS. Between three chairs: experiences of being a patient with chronic widespread pain in an intersectoral setting in Denmark. Health Psychol Open (2019) 6:2055102919852500.
80. Schütze R, Rees C, Slater H, Smith A, O’Sullivan P. “I call it stinkin’ thinkin’”: a qualitative analysis of metacognition in people with chronic low back pain and elevated catastrophizing. Br J Health Psychol (2017) 22:463–80.
81. Shehadeh A, Malak MZ, Ayed A. Holistic framing for improving care provision for older people with chronic diseases in Jordan: a phenomenological study. BMC Health Serv Res (2025) 25:492.
82. Skúladóttir H, Gunnarsdóttir TJ, Halldórsdóttir S, Sveinsdóttir H, Holden JE, Björnsdóttir A. Breaking the vicious circle: experiences of people in chronic pain on the pain rehabilitation journey. Nurs Open (2020) 7:1412–23.
83. Skúladóttir H, Halldórsdóttir S. The quest for well-being: self-identified needs of women in chronic pain. Scand J Caring Sci (2011) 25:81–91.
84. Skjutar Å, Müllersdorf M. Adapt, discover and engage: a qualitative interview study with patients living with chronic pain. J Nurs Healthcare Chronic Illn (2010) 2:254–61.
85. Snell GE, Seage CH, Mercer J. A phenomenological study on the lived experience of men with chronic fatigue syndrome. J Health Psychol (2024) 29:225–37.
86. Snelgrove S, Edwards S, Liossi C. A longitudinal study of patients’ experiences of chronic low back pain using interpretative phenomenological analysis: changes and consistencies. Psychol Health (2013) 28:121–38.
87. Stensland M. “If you don’t keep going, you’re gonna die”: helplessness and perseverance among older adults living with chronic low back pain. Gerontologist (2021) 61:907–16.
88. Stensland M. Managing the incurable: older pain clinic patients’ experiences of managing treatment-resistant chronic low back pain. J Gerontol Soc Work (2021) 64:405–22.
89. Stensland M, Sanders S. Living a life full of pain: older pain clinic patients’ experience of living with chronic low back pain. Qual Health Res (2018) 28:1434–48.
90. Stensland ML, Sanders S. “It has changed my whole life”: the systemic implications of chronic low back pain among older adults. J Gerontol Soc Work (2018) 61:129–50.
91. Stensland ML, Sanders S. Not so golden after all: the complexities of chronic low back pain in older adulthood. Gerontologist (2018) 58:923–31.
92. Stilwell P, Gagnon-Mailhot M, Hudon A, McIntyre V, Meldrum K, Pagé MG, et al. When pain overwhelms the self: a phenomenological study of a new mode of suffering, based on adults’ recollections of their worst pain episodes. J Pain.
93. Sun M, Liu L, Wang J, Zhuansun M, Xu T, Qian Y, et al. Facilitators and inhibitors in hospital-to-home transitional care for elderly patients with chronic diseases: a meta-synthesis of qualitative studies. Front Public Health (2023) 11:1047723.
94. Svensson HK, Olofsson EH, Karlsson J, Hansson T, Olsson LE. A painful, never ending story: older women’s experiences of living with an osteoporotic vertebral compression fracture. Osteoporos Int (2016) 27:1729–36.
95. Taylor B, Carswell K, Williams AC. The interaction of persistent pain and post-traumatic re-experiencing: a qualitative study in torture survivors. J Pain Symptom Manage (2013) 46:546–55.
96. Tollefson J, Usher K, Foster K. Relationships in pain: the experience of relationships to people living with chronic pain in rural areas. Int J Nurs Pract (2011) 17:478–85.
97. Toye F, Seers K, Hannink E, Barker K. A mega-ethnography of eleven qualitative evidence syntheses exploring the experience of living with chronic non-malignant pain. BMC Med Res Methodol (2017) 17:116.
98. Toye F, Seers K, Barker KL. Living life precariously with rheumatoid arthritis: a mega-ethnography of nine qualitative evidence syntheses. BMC Rheumatol (2019) 3:5.
99. Toye F, Belton J, Hannink E, Seers K, Barker K. A healing journey with chronic pain: a meta-ethnography synthesizing 195 qualitative studies. Pain Med (2021) 22:1333–44.
100. Twiddy H, Hanna J, Haynes L. Growing pains: understanding the needs of emerging adults with chronic pain. Br J Pain (2017) 11:108–18.
101. Vallerand A, Nowak L. Chronic opioid therapy for nonmalignant pain: the patient’s perspective. Part II—barriers to chronic opioid therapy. Pain Manag Nurs (2010) 11:126–31.
102. van Rysewyk S, Blomkvist R, Chuter A, Crighton R, Hodson F, Roomes D, et al. Understanding the lived experience of chronic pain: a systematic review and synthesis of qualitative evidence syntheses. Br J Pain (2023) 17:592–605.
103. West C, Stewart L, Foster K, Usher K. The meaning of resilience to persons living with chronic pain: an interpretive qualitative inquiry. J Clin Nurs (2012) 21:1284–92.
104. Yildirim Duman JG. Self-management of chronic diseases: a descriptive phenomenological study. Soc Work Public Health (2021) 36:300–10.

**References for the second phase of the synthesis: Transdisciplinarity**

1. Yang X, Lo K. Environmental health research and the COVID-19 pandemic: a turning point towards sustainability. Environ Res (2021) 197:111157.
2. Fischer C, Gugerell K, Laa U, Jacobsen J, Penker M. Evaluating transdisciplinary methods: a new scale for measuring knowledge integration. Humanit Soc Sci Commun (2025) 12:1395.
3. de Jager LA, Bal M, Baudena M, van den Broek KL, Davis N, Dijkstra HA, et al. Transdisciplinary complexity science: deepening system understanding for sustainability. Humanit Soc Sci Commun (2025) 12:1384.
4. Wouters R, Cox A, Raes A. Co-producing knowledge in and about education by combining collaborative ethnography and abductive reasoning. Humanit Soc Sci Commun (2025) 12:1405.
5. Vienni-Baptista B, Pohl CE. Exploring interdisciplinarity and transdisciplinarity as knowledge regimes: a heuristic tool for disentangling understandings in academia and policy. Sci Technol Hum Values (2024) 49:1309–48.
6. Yang L, Shewchuk B, Shang C, Lee JA, Gehlert S. Transdisciplinary team science in health research: where are we? J Integr Des Process Sci (2023) 26:307–16.
7. Van der Vlegel-Brouwer W, Eelderink M, Bussemaker J. Participatory action research as a driver for health promotion and prevention: a co-creation process between professionals and citizens in a deprived neighbourhood in The Hague. Int J Integr Care (2023) 23:13.
8. Renn O. Transdisciplinarity: synthesis towards a modular approach. Futures (2021) 130:102744.
9. Chamberlain C, Clark Y, Hokke S, Hampton A, Atkinson C, Andrews S, et al. Healing the past by nurturing the future: Aboriginal parents’ views of what helps support recovery from complex trauma. Prim Health Care Res Dev (2021) 22:e47.
10. Hantel A, Marron JM, Abel GA. Establishing and defining an approach to climate conscious clinical medical ethics. Am J Bioeth (2025) 25:8–21.
11. Ciesielski TH, Aldrich MC, Marsit CJ, Hiatt RA, Williams SM. Transdisciplinary approaches enhance the production of translational knowledge. Transl Res (2017) 182:123–34.
12. Carey G, Malbon E, Carey N, Joyce A, Crammond B, Carey A. Systems science and systems thinking for public health: a systematic review of the field. BMJ Open (2015) 5:e009002.
13. Kabarite A, Ferreira GMM, Pitangueira JC, Arimatéa RS, da Costa Rebello de Mendonça R, Marcello RS, et al. A longitudinal transdisciplinary approach for autism spectrum disorder. Children (Basel) (2025) 12:1272.
14. Gordon RM, Corcoran JR, Bartley-Daniele P, Sklenar D, Sutton PR, Cartwright F. A transdisciplinary team approach to pain management in inpatient health care settings. Pain Manag Nurs (2014) 15:426–35. Erratum in: Pain Manag Nurs (2014) 15:920.
15. Burris S, Ashe M, Levin D, Penn M, Larkin M. A transdisciplinary approach to public health law: the emerging practice of legal epidemiology. Annu Rev Public Health (2016) 37:135–48.
16. Mishra KK, Leung IC, Chao MT, Thompson-Lastad A, Pollak C, Dhruva A, et al. Mindfulness-based group medical visits: strategies to improve equitable access and inclusion for diverse patients in cancer treatment. Glob Adv Integr Med Health (2024) 13:27536130241263486.
17. Allen-Scott LK, Buntain B, Hatfield JM, Meisser A, Thomas CJ. Academic institutions and One Health: building capacity for transdisciplinary research approaches to address complex health issues at the animal–human–ecosystem interface. Acad Med (2015) 90:866–71.
18. Collins SE, Clifasefi SL, Stanton J, The LEAP Advisory Board, Straits KJE, Gil-Kashiwabara E, et al. Community-based participatory research (CBPR): towards equitable involvement of community in psychology research. Am Psychol (2018) 73:884–98.
19. Fontana L, Fasano A, Chong YS, Vineis P, Willett WC. Transdisciplinary research and clinical priorities for better health. PLoS Med (2021) 18:e1003699.
20. Keng SL, Smoski MJ, Robins CJ. Effects of mindfulness on psychological health: a review of empirical studies. Clin Psychol Rev (2011) 31:1041–56.
21. Knudsen RK, Skovbjerg S, Pedersen EL, Nielsen CL, Storkholm MH, Timmermann C. Factors affecting implementation of mindfulness in hospital settings: a qualitative meta-synthesis of healthcare professionals’ experiences. Int J Nurs Stud Adv (2024) 6:100192.
22. Lawless MT, Tieu M, Archibald MM, Pinero De Plaza MA, Kitson AL. From promise to practice: how health researchers understand and promote transdisciplinary collaboration. Qual Health Res (2025) 35:3–16.
23. Tobias Mortlock JM, Alapati HH, Edginton T. How to create a mindful community of practice: exploring the social functions of group-based mindfulness practices facilitated via Zoom during COVID-19. Front Psychol (2025) 16:1356057.
24. Marikar Bawa FL, Mercer SW, Sutton JW, Bond CM. Mindfulness for people with chronic pain: factors affecting engagement and suggestions for programme optimisation. Health Expect (2023) 26:1287–307.
25. Zeidan F, Vago DR. Mindfulness meditation-based pain relief: a mechanistic account. Ann N Y Acad Sci (2016) 1373:114–27.
26. Martin AK, Green TL, McCarthy AL, Sowa PM, Laakso EL. Allied health transdisciplinary models of care in hospital settings: a scoping review. J Interprof Care (2022) 37:118–30.
27. O’Shea M, Reich D, Evans S. Applying participatory research in the development of clinical practice recommendations for incorporating mindfulness into mental health treatment with youth at risk for psychosis. Mindfulness (2024) 15:721–32.
28. Martins P. Being transdisciplinary in human sciences: the usefulness of integrative medicine in contemporary society. Transdisciplinary J Eng Sci (2018) 9:37–43.
29. Schuman-Olivier Z, Trombka M, Lovas DA, Brewer JA, Vago DR, Gawande R, et al. Mindfulness and behavior change. Harv Rev Psychiatry (2020) 28:371–94.
30. Strugnell C, Orellana L, Crooks N, Malakellis M, Morrissey B, Rennie C, et al. Healthy Together Victoria and childhood obesity study: effects of a large-scale, community-based cluster randomized trial of a systems thinking approach for the prevention of childhood obesity among secondary school students (2014–2016). BMC Public Health (2024) 24:355.
31. Morgan MJ, Stratford E, Harpur S, et al. A systems thinking approach for community health and wellbeing. Syst Pract Action Res (2024) 37:161–83.
32. Anindhita M, Haniifah M, Putri AMN, et al. Community-based psychosocial support interventions to reduce stigma and improve mental health of people with infectious diseases: a scoping review. Infect Dis Poverty (2024) 13:90.
33. Kanyagui MK, Sajithkumar KJ, Velankar Y, Mohan R, Viswanathan PK, Magnani N. Livelihood challenges faced by women in rural India: exploration of solutions using participatory action research. Dev Pract (2023) 34:512–26.
34. Lee JY, Clark JK, Schmiesing R, Kaiser ML, Reece J, Park S. Perspectives of community members on community-based participatory research: a systematic literature review. J Urban Aff (2024) 47:2916–35.
35. Thomas E, Benjamin-Thomas TE, Sithambaram A, Shankar J, Chen S-P. Participatory action research among people with serious mental illness: a scoping review. Qual Health Res (2023) 34:3–19.
36. Ramanadhan S, Alemán R, Bradley CD, Cruz JL, Safaeinili N, Simonds V, Aveling EL. Using participatory implementation science to advance health equity. Annu Rev Public Health (2024) 45:47–67.
37. Bogerd MJ, Slottje P, Bont J, et al. Development of a person-centred care approach for persons with chronic multimorbidity in general practice by means of participatory action research. BMC Prim Care (2024) 25:114.
38. Carrick J, Bell D, Fitzsimmons C, Gray T, Stewart G. Principles and practical criteria for effective participatory environmental planning and decision-making. J Environ Plann Manage (2022) 66:2854–77.
39. Kwamie A, Causevic S, Tomson G, Sie A, Sauerborn R, Rasanathan K, et al. Prepared for the polycrisis? The need for complexity science and systems thinking to address global and national evidence gaps. BMJ Glob Health (2024) 9:e014887.
40. Rusoja E, Haynie D, Sievers J, Mustafee N, Nelson F, Reynolds M, et al. Thinking about complexity in health: a systematic review of the key systems thinking and complexity ideas in health. J Eval Clin Pract (2018) 24:600–6.
41. Andonian BJ, Hippensteel JA, Abuabara K, Boyle EM, Colbert JF, Devinney MJ, et al. Inflammation and aging-related disease: a transdisciplinary inflammaging framework. Geroscience (2025) 47:515–42.
42. Wan TTH, Wan HS. Predictive analytics with a transdisciplinary framework in promoting patient-centric care of polychronic conditions: trends, challenges, and solutions. AI (2023) 4:482–90.
43. Archibald MM, Lawless MT, de Plaza MAP, et al. How transdisciplinary research teams learn to do knowledge translation (KT), and how KT in turn impacts transdisciplinary research: a realist evaluation and longitudinal case study. Health Res Policy Syst (2023) 21:20.
44. Fontana L, Fasano A, Chong YS, Vineis P, Willett WC. Transdisciplinary research and clinical priorities for better health. PLoS Med (2021) 18:e1003699.
45. Shrivastava P, Stafford Smith M, O’Brien K, Zsolnai L. Transforming sustainability science to generate positive social and environmental change globally. One Earth (2020) 2:329–40.
46. Sather EW, Iversen VC, Svindseth MF, et al. Exploring sustainable care pathways: a scoping review. BMC Health Serv Res (2022) 22:1595.
47. Cardol CK, Tommel J, van Middendorp H, Ciere Y, Sont JK, Evers AW, et al. Detecting and treating psychosocial and lifestyle-related difficulties in chronic disease: development and treatment protocol of the E-GOAL eHealth care pathway. Int J Environ Res Public Health (2021) 18:3292.
48. Villani C, Sapio M, Cassarà G, Patti Y, Cupane L, Giorgi V, et al. Pharmakon or the healing art: experience of artistic-transformative transdisciplinary workshops in fibromyalgia syndrome. Clin Exp Rheumatol (2025) 43:978–89.
49. Nguyen P, Gietzen L. Transdisciplinary collaboration of healthcare professionals and the emotional connectedness with working with patients coping with death and dying. Pac J Health (2024) 7:35.
50. Lassell RK, Pritchard K, Baehr LA, Ceïde ME, Chatterjee SA, Colón-Semenza C. New horizons in advancing lifestyle medicine for older adults utilizing a transdisciplinary approach: gaps and opportunities. Age Ageing (2025) 54:afaf159.
51. Martins P, Matos L, Moreira A, Simoes M, Silva B. A transdisciplinary approach of the role of consciousness and spirituality in physics and healing. Transdisciplinary J Eng Sci (2025) 16.
52. McLure J, Arnautovska U, Panizzutti B, Jiang JC, Robinson S, Drummond KD, et al. New hope for individuals with schizophrenia: a transdisciplinary research platform to discover and translate novel treatments. Aust N Z J Psychiatry (2025) 59:569–72.
